# Synthesis of Cyclohepta[*b*]indoles by (4 + 3) Cycloaddition of 2-Vinylindoles or 4*H*-Furo[3,2-*b*]indoles with Oxyallyl Cations

**DOI:** 10.1021/acs.joc.9b03117

**Published:** 2020-01-24

**Authors:** Valentina Pirovano, Elisa Brambilla, Andrea Moretti, Silvia Rizzato, Giorgio Abbiati, Donatella Nava, Elisabetta Rossi

**Affiliations:** † Dipartimento di Scienze Farmaceutiche−Sezione di Chimica Generale e Organica “A. Marchesini”, Università degli Studi di Milano, Via Venezian 21, 20133 Milano, Italy; ‡ Dipartimento di Chimica, Università degli Studi di Milano, Via Golgi 19, 20133 Milano, Italy

## Abstract

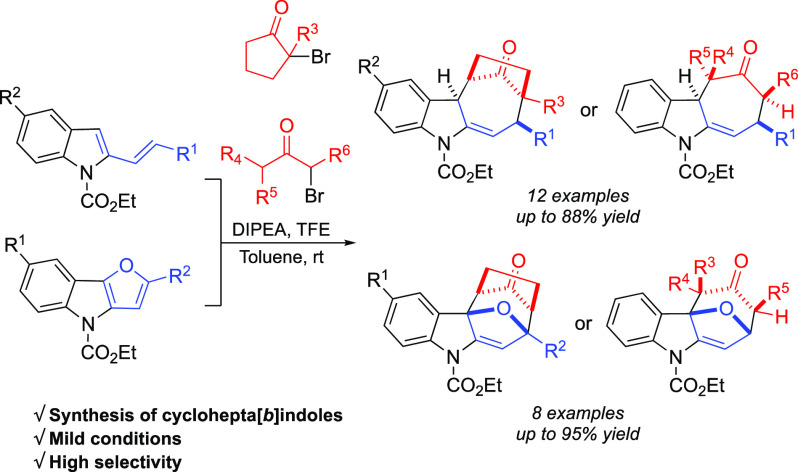

The synthesis of cyclohepta[*b*]indole derivatives through the dearomative (4 + 3) cycloaddition reaction of 2-vinylindoles or 4*H*-furo[3,2-*b*]indoles with in situ generated oxyallyl cations is reported. Oxyallyl cations are generated from α-bromoketones in the presence of a base and a perfluorinated solvent. Cyclohepta[*b*]indole scaffolds are obtained under mild reaction conditions, in the absence of expensive catalysts, starting from simple reagents, in good to excellent yields and with complete diasteroselectivity. Preliminary expansion of the scope to 3-vinylindoles and to aza-oxyallyl cations is reported.

## Introduction

**Figure 1:**
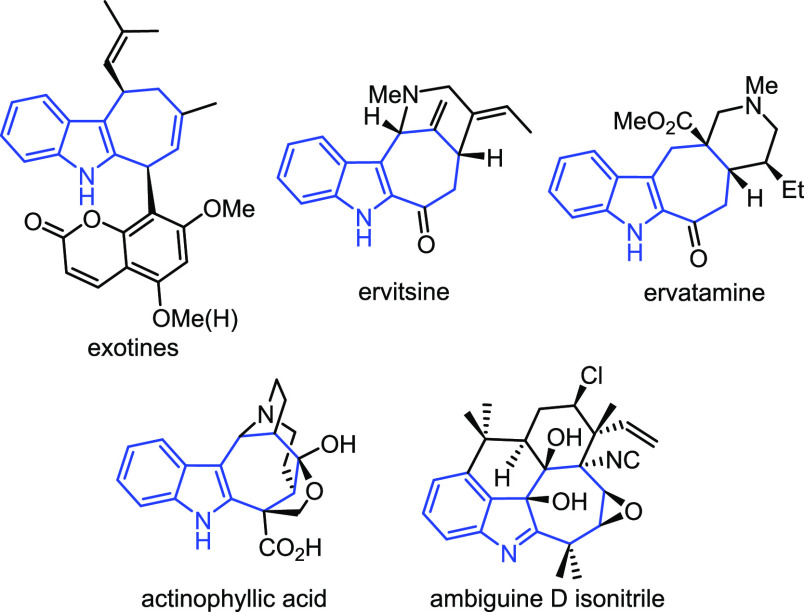
Natural products containing the cyclohepta[*b*]indoles scaffold.

The cyclohepta[*b*]indole is the core privileged structure of a variety of natural as well as non-natural compounds having different degrees of structural complexity in addition to a great variety of biological activities. Gaich and Stempel have recently organized all of these features in an exhaustive review.^1^ In particular, they describe the structural geography of different families of cyclohepta[*b*]indoles alkaloids ranging from the simplest exotines and ervitsine–ervatamine alkaloids to the more complex actinophyllic acid and ambiguines (Figure 1).

**Figure 2:**
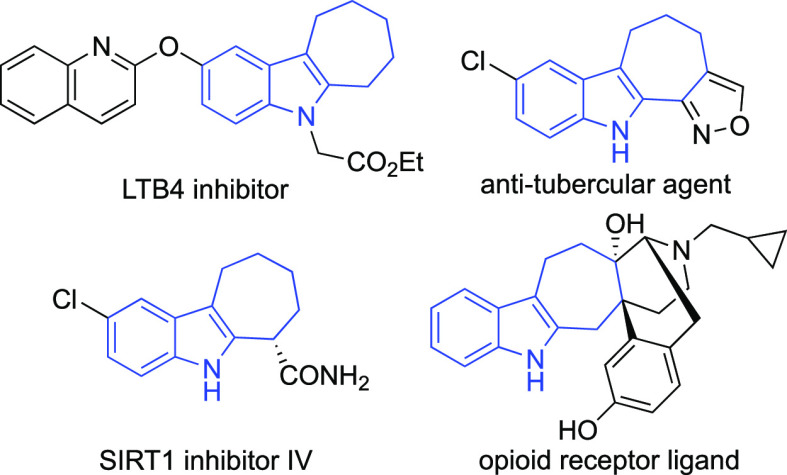
Non-natural cyclohepta[*b*]indole derivatives.

Moreover, as is often the case, the reported biological activities attracted the interest of both medicinal and synthetic chemists for the rational design of new therapeutic agents (Figure 2) and for the development of efficient synthetic methods.

**Scheme 1:**
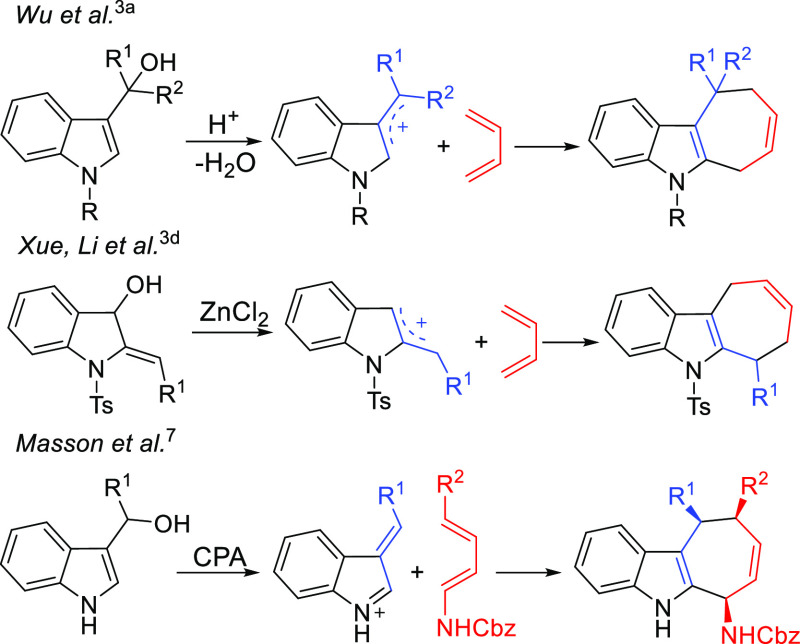
Indolyl Derivatives as 3C Partners in (4 + 3) Cycloadditions with Dienes CPA = chiral phosphoric acid.

It is about this last aspect that Gaich and Stempel have made several useful points. Notably, apart from the well-known Fischer indole synthesis, limited to the synthesis of symmetrically substituted cyclohepta[*b*]indoles,^2^ most reported methodologies involve the use of cycloaddition reactions,^3^ sigmatropic rearrangements,^4^ and palladium-catalyzed cyclizations.^5^ The most representative and versatile protocols involve (4 + 3)^6^ cycloadditions (Scheme 1) and were developed, beyond the examples reported by Gaich and Stempel, also in their enantioselective version.^7^

**Scheme 2:**
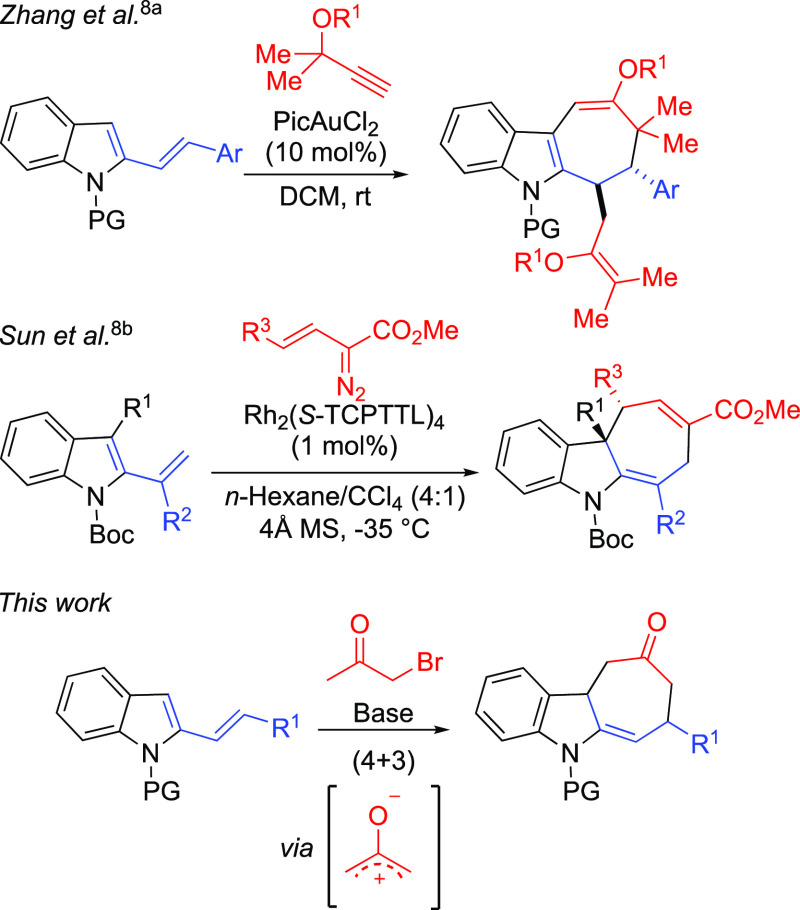
Previous and Present Works Using Indoles as 4C Components PicAuCl_2_ = dichloro(2-pyridinecarboxylato) gold. Rh_2_(*S*-TCPTTL)_4_ = tetrakis[*N*-tetrachlorophthaloyl-(*S*)-*tert*-leucinato]dirhodium bis(ethyl acetate) adduct.

In these cycloaddition reactions, the indolyl moiety functions as the 3C partner, whereas (4 + 3) cycloaddition reactions having indoles as the 4C component have become operative only more recently (Scheme 2).^8^

For example, in 2017, Zhang and co-workers reported a regioselective gold-catalyzed (4 + 3) cascade cycloaddition/CH functionalization of 2-vinylindoles and propargylic esters leading to highly substituted derivatives.^8a^ In 2018, Sun described an enantioselective rhodium-catalyzed (4 + 3) cycloaddition of both 2- and 3-vinylindoles with vinyldiazoesters leading to dearomatized cyclohepta[*b*]indolines in high yields and enantiomeric excesses.^8b^ A 3-alkenylindole was also considered as the reactive intermediate in the iron(III)-catalyzed reaction between simple indoles and *o*-hydroxychalcone.^8c^ Taking into account these precedents and our interest in the synthesis of complex indole derivatives through cycloaddition reactions of 2-vinylindoles,^9^ we decided to test the reactivity of 2-vinylindoles with oxyallyl cations in order to synthetize cyclohepta[*b*]indoles through (4 + 3) cycloaddition reactions. The use of oxyallyl cations as three-carbon partners in [3 + *n*] cycloadditions has been widely studied and includes both (3 + 2)^10^ and (4 + 3)^11^ cycloaddition reactions. Oxyallyl cations can be generated from α-haloketones, α,α′-dihaloketones, and allene oxides and by Nazarov cyclization,^12^ among other precursors. We chose to focus our attention on the base-mediated dehydrohalogenation of α-haloketones. This approach, in fact, employs simple and easy-accessible starting materials allowing for the easy generation of diversely substituted oxyallyl cations. In this paper, we report a full account of the obtained results.

## Results and Discussion

In order to test the viability of our idea, 2-vinylindole **1a** and 2-bromocyclopentan-1-one **2a** were selected as model substrates and reacted in the presence of different bases and/or fluorinated solvents. These solvents, in fact, possess unique qualities, including the capability to activate carbonyl groups and stabilize cationic intermediates, and were reported as solvents of choice in related reactions.^13^ The results obtained during the optimization of the reaction conditions are summarized in Table 1.

**Table 1:**
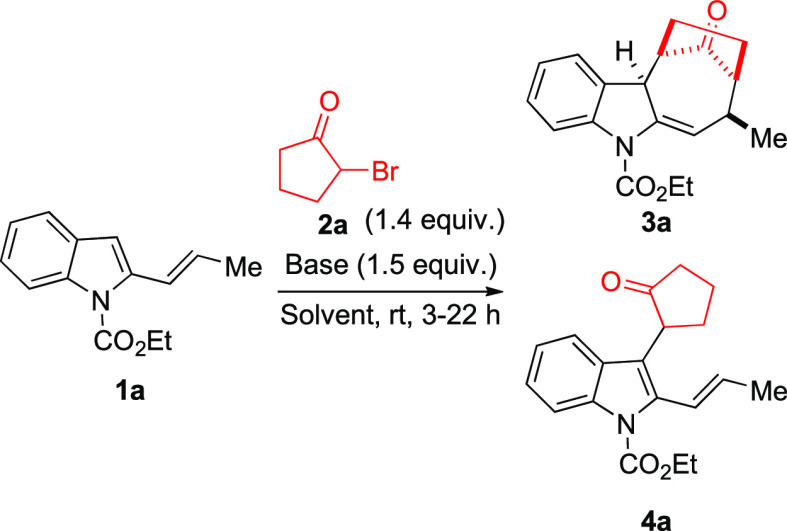
Optimization of Reaction Conditions for the Synthesis of **3a**^a^^,^^c^

entry	base	solvent	time, h	**3a**^b^ (%)	**4a**^b^ (%)
1	Na_2_CO_3_	TFE (1 M)	3	67	17
2	Et_3_N	TFE (1 M)	1	56	26
3	DIPEA	TFE (1 M)	1	75	17
4	DBU	TFE (1 M)	3	50	15
5^c^	DIPEA	TFE (1 M)	2	53	32
6	DIPEA	HFIP (1 M)	1	53	47
7	DIPEA	TFE (1 equiv) toluene (0.5 M)	22	32	<5
8	DIPEA	TFE (3 equiv) toluene (0.5 M)	6	53	<5
9	DIPEA	TFE (6 equiv) toluene (0.5 M)	1	88	<5
10	DIPEA	TFE (6 equiv) CH_2_Cl_2_ (0.5 M)	1	74	13
11	DIPEA	LiClO_4_ (1 equiv) Et_2_O (0.5 M)	22	27	<5

a Reaction conditions: **1a** (0.2 mmol), **2a** (0.28 mmol), base (0.3 mmol) in the stated solvent or in TFE/solvent mixture at rt for 1–22 h.

b Isolated yield.

c Reaction performed at −20 °C.

At the outset, we focused our attention on the influence of different bases on the reaction outcome using 2,2,2-trifluoroethanol (TFE) as the solvent. Both inorganic (Na_2_CO_3_, entry 1) and organic bases, [Et_3_N, *N*,*N*-diisopropylethylamine (DIPEA) and 1,8-diazabicycloundec-7-ene (DBU), entries 2–4], led to the formation of desired dearomatized cycloadduct **3a** together with a minor amount of product **4a** arising from the nucleophilic addition of C3 of the indole nucleus on the in situ generated oxyallyl cation.^14^ Better results in terms of the **3a/4a** ratio were achieved with DIPEA, which was selected as the best base for the following optimization steps. Then, in order to reduce the competitive formation of **4a**, we modified both the reaction temperature and solvent. However, the reduction of the reaction temperature down to −20 °C (entry 5), as well as the use of 1,1,1,3,3,3-hexafluoro-2-propanol (HFIP) (entry 6), had a negative impact on the formation of **3a**, increasing the formation of **4a**. Taking into account these results, we decided to verify the influence of TFE in promoting the formation of the desired cycloadduct **3a**, by a progressive increase of its concentration from 1 to 6 equiv in a 0.5 M solution of the reactants in toluene. As a result, we observed that the use of an equimolar amount of TFE (entry 7) significantly reduced the reaction rate but strongly inhibited the formation of **4a**. A better yield and faster reaction time were obtained using 3 equiv of TFE (entry 8). The optimal 88% yield of **3a** was finally achieved employing 6 equiv of fluorinated alcohol (entry 9). Switching from toluene to dichloromethane slightly worsened the reaction outcome in both terms of yield and selectivity (entry 10), while the use of a classical Lewis acid such as LiClO_4_ in diethyl ether led to a significantly lower yield (entry 11).^11f^ Notably, in all tested reactions, **3a** was isolated as a single diastereoisomer, the structure of which was fully elucidated by 1D- and 2D-NMR analyses (see Supporting Information).

**Scheme 3:**
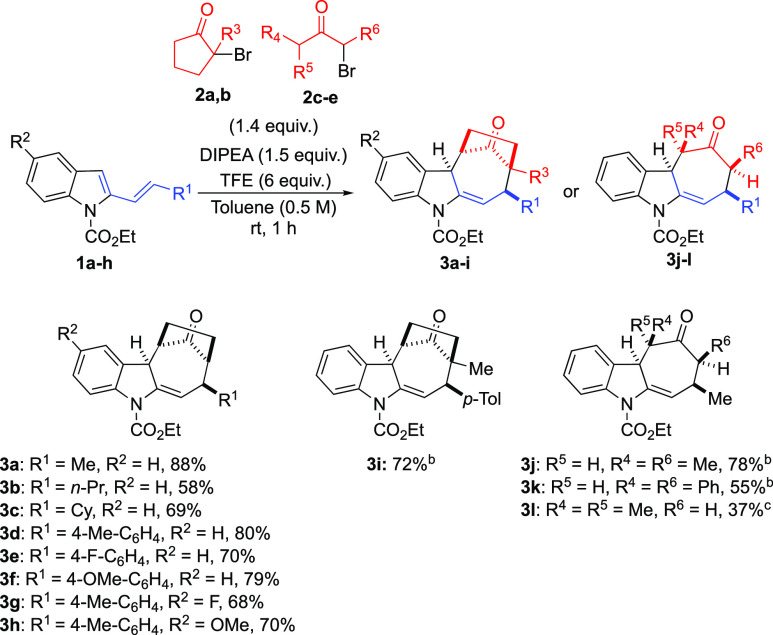
Scope of the Reaction between **1a–h** and **2a–e** Reaction conditions: **1a–h** (0.2 mmol), **2a–e** (0.28 mmol), DIPEA (0.3 mmol), and TFE (1.2 mmol) in toluene (0.4 mL) for 1 h at rt. ^b^TFE (1 M) was used as the solvent for 24 h at rt. ^c^Na_2_CO_3_ (0.3 mmol) was used as a base in TFE (1 M) for 48 h at 40 °C.

With the best conditions in hand, we then explored the scope of the reaction with different substituted 2-vinylindoles and α-bromoketones (Scheme 3).

**Scheme 4:**
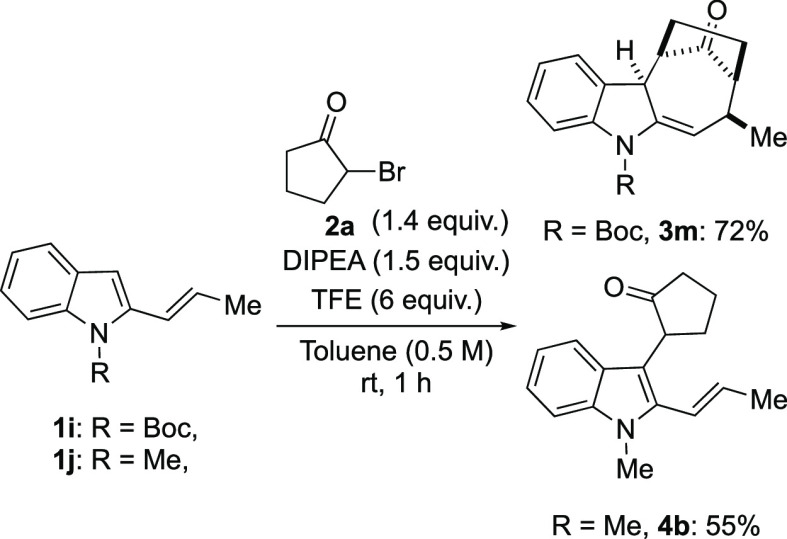
Reaction between **1i–j** and **2a**

We first focused on the modification of the indole vinyl moiety by using different β-alkyl and β-aryl-substituted 2-vinylindoles. The substitution of the methyl group with a longer alkyl chain or with a cyclohexyl ring was well tolerated, and the corresponding indolines **3b** and **3c** were isolated in 58 and 69% yield, respectively, in addition to residual amounts of starting vinylindoles, nucleophilic substitution products (less than <10%), and traces of other unidentified side products. Aryl-substituted 2-vinylindoles reacted efficiently as well. In particular, 4-methylstyrylvinylindole (**1d**) afforded **3d** in a satisfying 80% yield, while related vinylindoles bearing electron-withdrawing (**1e**) or electron-donating (**1f**) substituents led to cycloadducts **3e–f** in comparable 70 and 79% yields. Next, we introduced different substituents on 5-position of the indole skeleton in order to evaluate variation in the reactivity of the vinylindole due to a reduced or augmented nucleophilicity of the carbon in position 3. As a result, we observed that 5-fluoro derivative **1g** smoothly reacted with **2a** to give **3g** in 68% yield, while 5-methoxy-substituted **1h** led to **3h** in 70% yield. We then evaluated the influence of ketones other than 2-bromocyclopentan-1-one on the reaction course. The employment of 2-bromo-2-methylcyclopentan-1-one (**2b**) was tolerated; however, the reaction performed under optimized conditions resulted in a significantly lower conversion of starting materials even after prolonged reaction times (less than 10% after 96 h at rt). Surprisingly, with this more substituted ketone, the use of TFE as the sole solvent (1 M) permitted the isolation of **3i** in a satisfying 72% yield. Similarly, symmetrically substituted acyclic ketones **2c** and **2d** led to the corresponding products **3j** and **3k** in 78 and 55% yields, respectively, only when TFE was used as the solvent. In all the last cases, a residual amount of unreacted vinylindole was recovered along with traces of unidentified byproducts. On the other hand, the reaction between **1a** and non-symmetrically disubstituted ketone **2e** was more challenging and did not proceed even in TFE at 40 °C. In this case, after a brief screening of reactions conditions, we were able to isolate **3l** as a single isomer in moderate 37% yield, only by using Na_2_CO_3_ in TFE (1 M) for 48 h at 40 °C. Finally, we verified the influence of the substituent on vinylindole nitrogen employing *N*-Boc and *N*-methyl 2-vinylindoles **1i** and **1j** under optimized reaction conditions. The use of Boc-derivative **1i** led to results comparable to those obtained with **1a**, affording **3m** in 72% yield. On the other hand, the presence of a mild electron-donating group on the indole nitrogen gave the nucleophilic addition product **4b** as exclusive reaction product in 55% yield beside a small amount of unreacted **1j**, confirming the pivotal presence of an electron-withdrawing protecting group on vinylindole nitrogen in order to support the cycloaddition pathway (Scheme 4).^15^

**Scheme 5:**
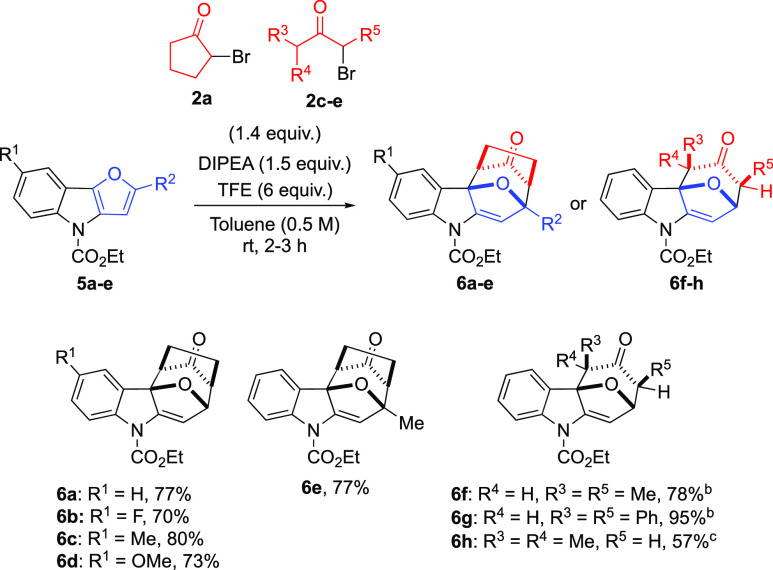
Scope of the Reaction between **5a–e** and **2a–e** Reaction conditions: **5a–e** (0.2 mmol), **2a,c–e** (0.28 mmol), DIPEA (0.3 mmol), and TFE (1.2 mmol) in toluene (0.4 mL) for 2–3 h at rt. ^b^TFE (1 M) was used as the solvent for 24 h at rt. ^c^Na_2_CO_3_ (0.3 mmol) was used as base in TFE (1 M) for 48 h at 40 °C.

Moreover, in the context of our studies on the metal-catalyzed functionalization of indoles,^16^ we recently reported the synthesis of ethyl 4*H*-furo[3,2-*b*]indole-4-carboxylates, an interesting class of heterocyclic compounds, which could be employed in gold-catalyzed reactions to give indolin-3-one derivatives.^17^ Taking a look into the structure of these substrates, we observed that they could be considered as an attractive alternative to 2-vinylindoles, in which the diene system is embedded in the furan ring and constrained in a s-cis conformation. Thus, we decided to test their reactivity in these (4 + 3) cycloadditions under the previously optimized conditions in order to expand the scope of our transformation (Scheme 5).

As supposed, when we reacted 4*H*-furo[3,2-*b*]indole-4-carboxylate **5a** with cyclopentyl oxyallyl cation generated in situ with TFE and DIPEA, we were able to isolate 7,8-dihydro-5*H*-7,10a-epoxycyclohepta[*b*]indole derivative **6a** as a single product in high yields (77%) after 2 h. Notably, in this case, no product arising from the nucleophilic substitution on the furan moiety was observed or isolated. As for **3a**, the structure of indoline **6a** was confirmed by 2D-NMR spectra and by X-ray diffraction analysis on a single crystal (see Supporting Information for details).

Similarly, 5-substituted furoindoles **5b–d** were efficiently transformed into their corresponding cycloaddition products **6b–d**, suggesting that the presence of both electron-withdrawing and electron-donating groups on this position does not affect the reaction outcome. We also employed furoindoles substituted on the furan moiety. In this case, methyl-substituted **5e** afforded **6e** in 77% yield after 3 h. Finally, as for 2-vinylindoles, 2-bromopentan-3-one (**2c**) and 1-bromo-1,3-diphenylpropan-2-one (**2d**) were used instead of **2a**. The reaction of these haloketones required the use of TFE as the solvent and resulted in the isolation of **6f** and **6g** in 78 and 95% yield, respectively. In addition, 1-bromo-3-methylbutan-2-one (**2e**) reacted with **5a** to give **6h** as a single isomer in 57% yield, but only when Na_2_CO_3_ was used as a base in TFE at 40 °C for 48 h.

**Scheme 6:**
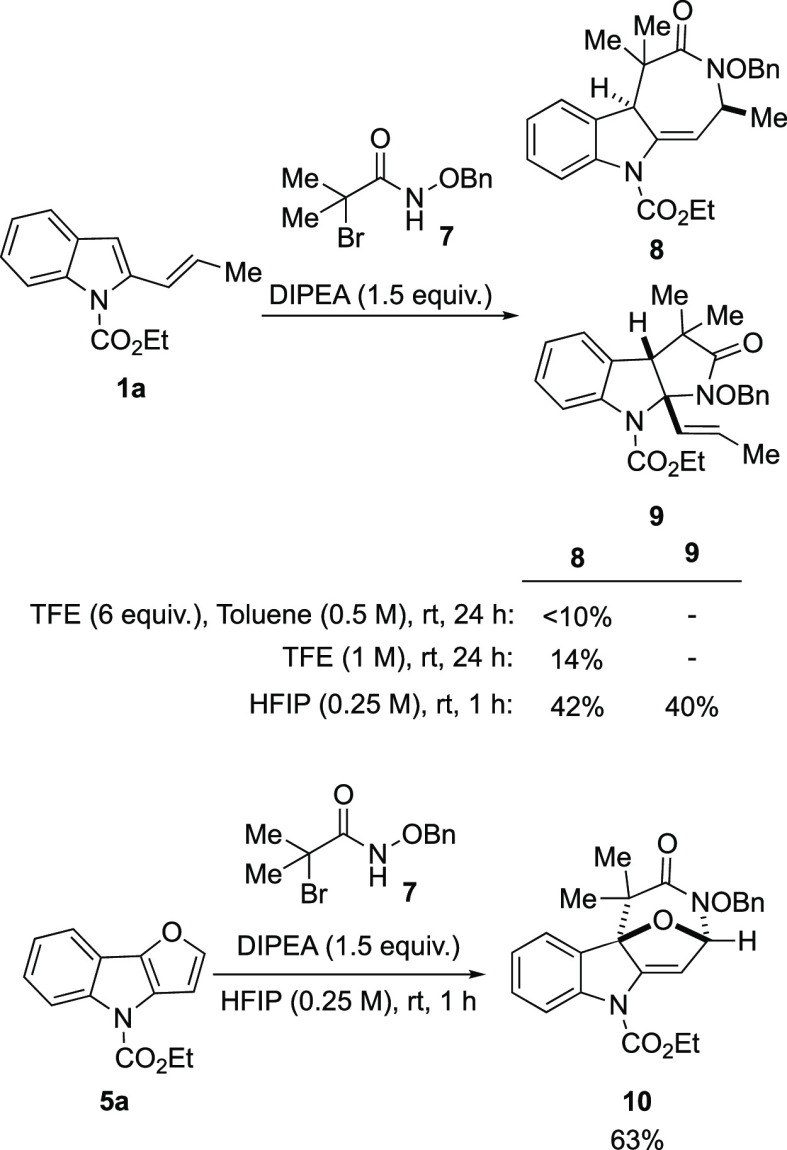
Extension of the Scope to Aza-Oxyallyl cations

Further, considering the great number of reports on cycloaddition reactions with aza-oxyallyl cations,^18^ we decided to examine whether these substrates could be suitable partners in the (4 + 3) cycloaddition with vinylindole **1a** under our optimized conditions (Scheme 6). However, in this case, the reactions were extremely slow, and only traces of products were observed after 24 h. Using pure TFE as the solvent, we were able to isolate a 14% yield of **8** after 24 h, while the switch to other fluorinated alcohols such as HFIP led to rapid and full conversion of the starting material to give a separable 1:1 mixture of (4 + 3) and (3 + 2) cycloaddition products, **8** and **9**,^19^ in overall 82% yield. Further studies to improve the selectivity toward (4 + 3) cycloadducts are now in progress in our laboratory. In addition, we tested the reactivity of furoindole **5a**, and in this case, we were able to isolate cycloadduct **10** as a single product in 63% yield.

**Scheme 7:**
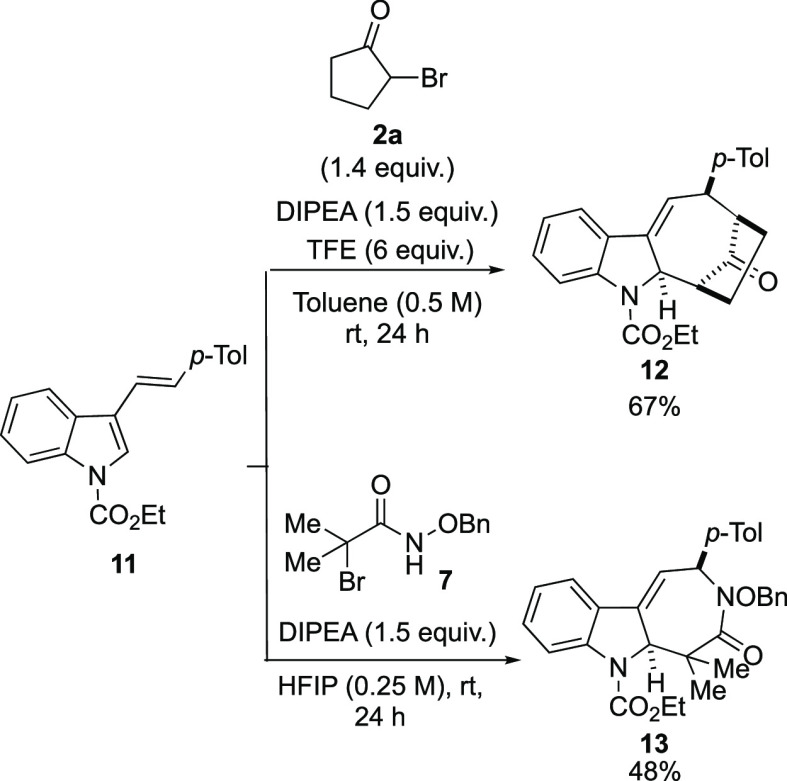
Reaction between 3-Vinylindole **11** and **2a** or **7**

Subsequently, we studied the behavior of 3-vinylindoles by reacting **11** and **2a** under the optimized conditions. Substrate **11** was less reactive than the isomeric 2-vinylindole **3d**, and the reaction required 24 h to afford cycloadduct **12** in 67% yield (Scheme 7). In addition, the same substrate reacted with azaoxyallyl cation generated from **7** to give (4 + 3) derivative **13** as a single product using HFIP as the solvent. In this case, the reaction was also slow and required 24 h to provide **13**, in addition to unreacted 3-vinylindole.

**Scheme 8:**
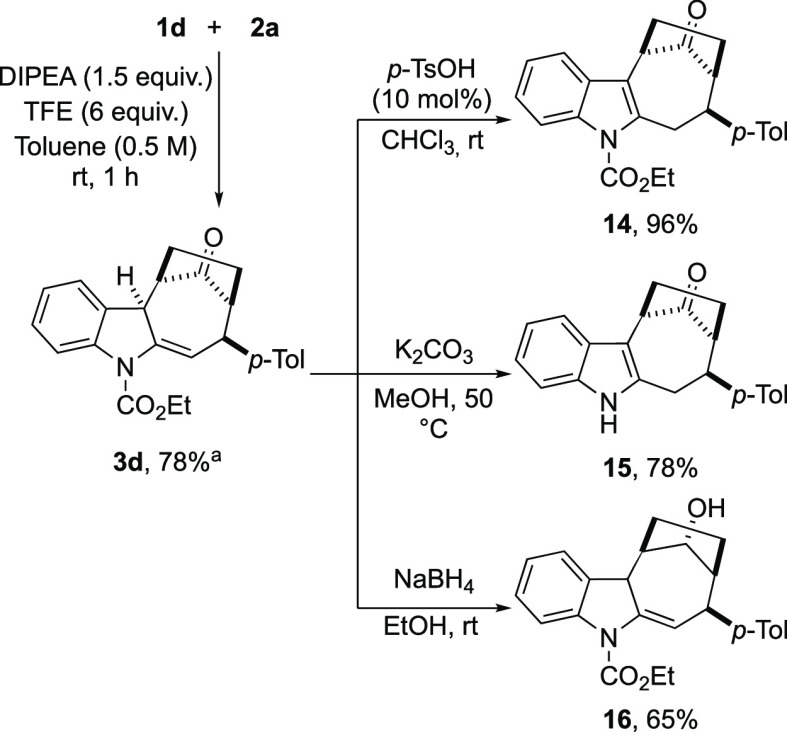
Selective Functional Group Transformations on Product **3d** Reaction performed on 1.64 mmol scale.

Having synthesized a series of cyclohepta[*b*]indoles **3a–l**, we finally focused our attention in proposing simple and effective modifications of these scaffolds. To this end, **3d** was prepared on a gram scale, and it was subjected to selected transformations (Scheme 8). Thus, we observed that **3d** quantitatively aromatized to give **14** up on treatment with catalytic amounts of *p*-TsOH, while under basic hydrolytic conditions, NH-free aromatic cycloheptaindole **15** was isolated in 78% yield. Moreover, the cycloheptanone ring of **3d** was effectively and selectively reduced with sodium borohydride to give the corresponding alcohol **16** in 65% yield.

**Scheme 9:**
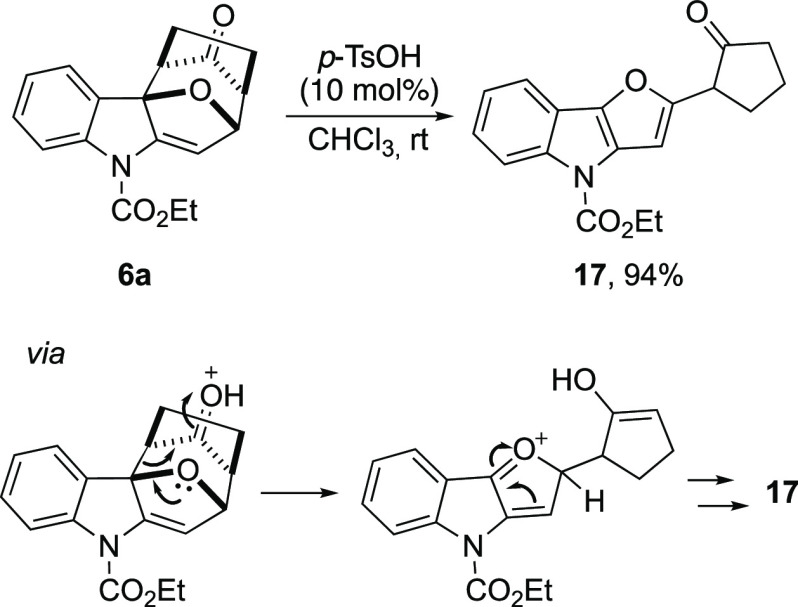
Behavior of **6a** under Acid Conditions

As above mentioned, aromatization of **3d** easily occurred under acid conditions affording the corresponding product almost quantitatively. For this reason, we became interested in verifying the behavior of **6a** under the same reaction conditions, considering that aromatization of such a product would probably require the ring-opening of the epoxy ring. Nevertheless, when we treated **6a** with catalytic amounts of *p*-TsOH in chloroform, we isolated the sole 2-(2-oxocyclopentyl)-4*H*-furo[3,2-*b*]indole derivative **17** in high 94% yield (Scheme 9). This result was not unexpected, and a similar behavior has already been described by Harmata for the acidic treatment of cycloadducts synthetized starting from 2-chloro-cyclopentanones and furans.^20^ Additionally, the conversion of **6a** to substituted furan **17** could be mechanistically ascribed to a Grob fragmentation^21^ of protonated **6a**, followed by the re-aromatization of the furan moiety and keto–enol tautomerism to regenerate the cyclopentanone ring (Scheme 9).

**Scheme 10:**
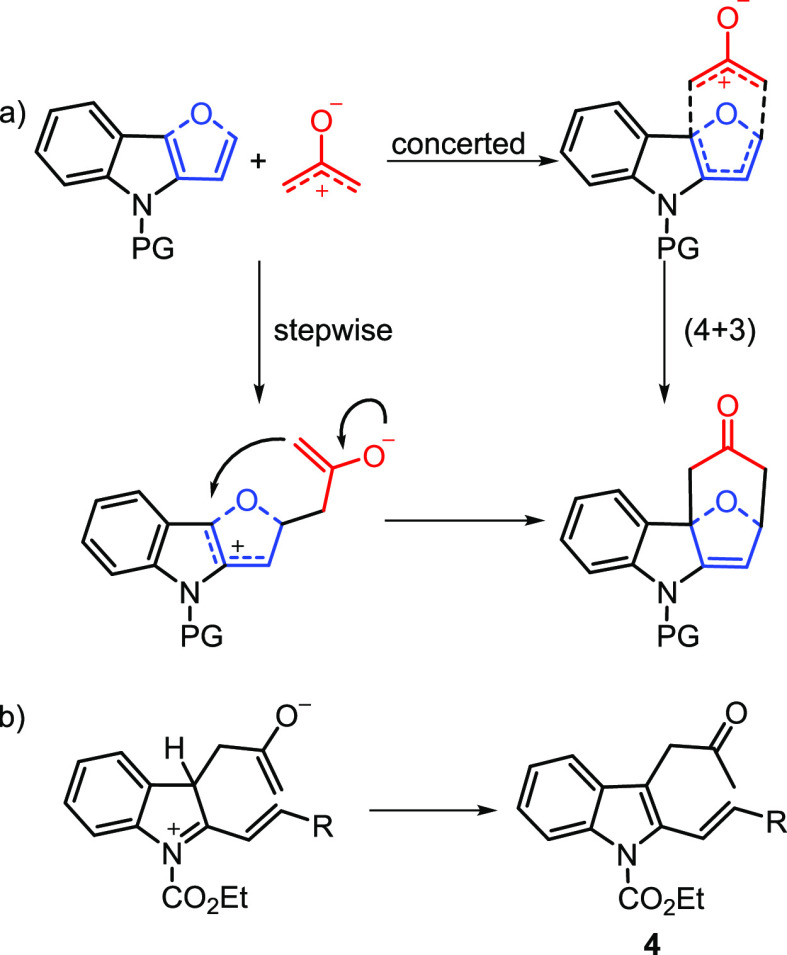
(a) Plausible Reaction Mechanism for (4 + 3) Cycloaddition with Oxyallyl Cations; (b) Formation of Nucleophilic Substitution Compound **4**

A plausible reaction mechanism for the (4 + 3)-cycloaddition reactions is not easy to describe nor to predict. In general, the reaction can be viewed as a (4 + 3) cycloaddition that relies on the use of α-haloketones as oxyallyl cation precursors (C3 fragment) and 2-vinylindoles or furoindoles as dienes (C4 fragment). Moreover, based on the IUPAC convention, the process is a homologue of the Diels–Alder reaction, a standard [4 + 2] cycloaddition considering the numbers of electrons involved. As reported in the literature,^11b,11c,11f^ these reactions occur through pathways ranging from a classical pure concerted process to processes that are stepwise (Scheme 10).

**Figure 3:**
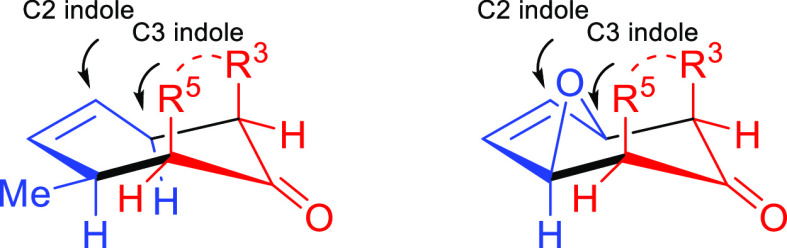
Stereochemical outcome derived from the endo approach.

The nature of the substrates involved as well as the reaction conditions employed affect the mechanism and in turn the chemical and stereochemical outcome of the reaction. In our cycloadditions, we observed complete regio- and diastereoselectivity. The stereochemistry of the isolated compounds arose from an endo approach between the diene and the dienophile (Figure 3).

Both open chain internal–external ring dienes (vinylindoles) and dienes embedded in a furan ring (furoindoles) gave analogous results. The same occurred using both cyclic and acyclic oxyallyl cation precursors. Based on these results, our reactions could be viewed as proceeding via a concerted mechanism. However, looking at the electronic features of the reacting dienes (polarized, electron rich) and dienophiles (electrophilic, TFE-stabilized), a pseudoconcerted or fast stepwise process cannot be excluded. In this context, Cramer^22^ and co-workers recently reported the results of their computational studies on the mechanism of related reactions. In particular, they demonstrated that stepwise processes are more favored for electron-rich dienes and electrophilic oxyallyl cations. Furthermore, a mechanism involving cationic intermediates is plausibly operating in the reaction of 2-vinylindoles as demonstrated by the isolation of compound **4a**, arising from the first intermediate of the stepwise process by a proton elimination/re-aromatization reaction (Scheme 10).

Finally, several remarks on the role of TFE on the reaction outcome can be made. The role of TFE in these reactions is to assist and accelerate the deprotonation of α-haloketones and their subsequent ionization, via hydrogen bond formation. Cyclic ketones require low amounts of TFE probably because they are sufficiently reactive to participate in the cycloaddition. Indeed, an excess of TFE lowers the reaction selectivity, favoring the formation of undesired nucleophilic substitution compounds. However, when open chain and hindered substrates were involved, pure TFE must be used as the solvent, in some cases in the presence of a base stronger than DIPEA in order to facilitate both the enolization and the abstraction steps.

## Conclusions

In conclusion, we developed a selective and efficient synthesis of complex cyclohepta[*b*]indole derivatives through the dearomative (4 + 3) cycloaddition reaction of vinylindoles or 4*H*-furo[3,2-*b*]indoles with oxyallyl cations. Oxyallyl cations were efficiently generated in situ starting from the corresponding α-haloketones using DIPEA and TFE under mild reaction conditions.

Differently from the well-known methods for synthetizing cyclohepta[*b*]indoles, in which the indolyl moiety contributes to the (4 + 3) cycloaddition as a 3C unit, our approach exploits the ability of vinylindoles to react as a 4C partner in these cycloaddition reactions. It is worth noting that the use of these latter substrates in (4 + 3) cycloaddition reactions has been scarcely described in the literature. Moreover, the existing methodologies require the intermediacy of a metal vinylcarbene intermediate as a 3C partner, generated from propargyl esters or vinyldiazoacetates under gold and rhodium catalysis.^8^ Thus, the results obtained herein represent an expansion of the reactivity of vinylindoles as a 4C partner with C3 counterparts such as oxyallyl cations and demonstrate their utility as building blocks to create complex molecular architectures. Finally, a clear advantage resides in the use of simple and inexpensive starting materials, solvents, and additives that do not require the use of strictly controlled reaction conditions. The extension of the scope to other substrates such as 3-vinylindoles and aza-oxyallyl cations was also briefly explored as were further transformations of the obtained products.

## Experimental Section

All chemicals and solvents are commercially available and were used after distillation or treatment with drying agents. Silica gel F254 thin-layer plates were employed for thin-layer chromatography. Silica gel 40–63 μm/60 Å was employed for flash column chromatography. Melting points were measured with a PerkinElmer DSC 6 calorimeter at a heating rate of 5 °C/min and are uncorrected. ^1^H and ^13^C NMR spectra were determined with a Varian-Gemini 300, a Bruker 300, 500 AVANCE or 600 Bruker spectrometers at room temperature in CDCl_3_, CD_2_Cl_2_, C_6_D_6_, or acetone-*d*_6_ with residual solvent peaks as the internal reference. The APT sequences were used to distinguish the methine and methyl carbon signals from those arising from methylene and quaternary carbon atoms. Two-dimensional NMR experiments were performed for products **3a**, **3d**, **3i**, **3j**, **3l**, **6a**, **6f**, **6h**, **8**, **9**, **10**, **12**, **16**, and **17** to aid the assignment of structures. Low-resolution mass spectrometry (MS) spectra were recorded with a Thermo-Finnigan LCQ advantage AP electrospray/ion trap equipped instrument using a syringe pump device to directly inject sample solutions.

2-Vinylindoles **1a–j**,^23^ ethyl 4*H*-furo[3,2-*b*]indole-4-carboxylates **5a–e**,^17^ ethyl (*E*)-3-(4-methylstyryl)-1*H*-indole-1-carboxylate **11**,^24^ α-haloketones **2a–e**,^25^ and *N*-(benzyloxy)-2-bromo-2-methylpropanamide **7**(19) are known compounds and were prepared according to literature procedures.

### General Procedure for the Reaction between 2-Vinylindoles **1a–i** and α-Haloketones **2a–e**

To a stirring solution of 2-vinylindole **1a–i** (0.2 mmol, 1.0 equiv), α-haloketone **2a–e** (0.28 mmol, 1.4 equiv), and TFE (86.4 μL, 1.2 mmol, 6.0 equiv) in toluene (0.4 mL, 0.5 M), DIPEA (52.3 μL, 0.3 mmol, 1.5 equiv) was added, and the mixture was stirred for 1 h at room temperature. The solvent was then removed, and the crude was purified by column chromatography to yield the corresponding cyclohepta[*b*]indole **3a–m**.

#### Ethyl 7-Methyl-12-oxo-7,8,9,10,11,11a-hexahydro-5*H*-8,11-methanocycloocta[*b*]indole-5-carboxylate (**3a**)

The general procedure was followed using ethyl (*E*)-2-(prop-1-en-1-yl)-1*H*-indole-1-carboxylate **1a** (46.0 mg, 0.2 mmol) and 2-bromocyclopentan-1-one **2a** (46.0 mg, 0.28 mmol). The purification of the crude by flash chromatography (SiO_2_, hexane/ethyl acetate 95:5) yielded **3a** (55 mg, 88%) as a yellow thick wax. ^1^H NMR (300 MHz, C_6_D_6_): 7.91 (d, *J* = 8.2 Hz, 1H), 7.08 (t, *J* = 7.4 Hz, 1H), 6.84 (t, *J* = 7.4 Hz, 1H), 6.76 (d, *J* = 7.7 Hz, 1H), 6.70 (t, *J* = 3.1 Hz, 1H), 4.05 (q, *J* = 7.1 Hz, 2H), 3.69 (m, 1H), 2.69 (m, 1H), 2.49 (m, 1H), 2.14 (m, 1H), 1.41–1.28 (m, 3H), 1.10 (m, 1H), 1.02–0.90 (m, 6H). ^13^C{^1^H} NMR (126 MHz, C_6_D_6_): 219.3 (C), 152.5 (C), 142.7 (C), 141.1 (C), 130.1 (C), 127.9 (CH), 123.5 (CH), 123.2 (CH), 116.7 (CH), 116.2 (CH), 61.7 (CH_2_), 54.3 (CH), 49.4 (CH), 47.6 (CH), 36.6 (CH), 22.7 (CH_3_), 21.1 (CH_2_), 20.4 (CH_2_), 13.9 (CH_3_). ESI(+)-MS *m*/*z* (%): 312 (100) [M + H]^+^. Anal. Calcd for C_19_H_21_NO_3_: C, 73.29; H, 6.80; N, 4.50. Found: C, 73.44; H, 6.77; N, 4.51.

#### Ethyl 12-Oxo-7-propyl-7,8,9,10,11,11a-hexahydro-5*H*-8,11-methanocycloocta[*b*]indole-5-carboxylate (**3b**)

The general procedure was followed using ethyl (*E*)-2-(pent-1-en-1-yl)-1*H*-indole-1-carboxylate **1b** (51.5 mg, 0.2 mmol) and 2-bromocyclopentan-1-one **2a** (46.0 mg, 0.28 mmol). The purification of the crude by flash chromatography (SiO_2_, hexane/ethyl acetate 95:5) yielded **3b** (39 mg, 58%) as a yellow thick wax. ^1^H NMR (300 MHz, CDCl_3_): 7.76 (d, *J* = 8.2 Hz, 1H), 7.26 (m, 1H), 7.21 (d, *J* = 7.9 Hz, 1H), 7.09 (t, *J* = 7.6 Hz, 1H), 6.60 (t, *J* = 3.1 Hz, 1H), 4.40 (q, *J* = 7.1 Hz, 2H), 3.83 (s, 1H), 2.86 (m, 1H), 2.44 (d, *J* = 7.0 Hz, 1H), 2.31 (s, 1H), 1.85–1.76 (m, 2H), 1.66–1.48 (m, 2H), 1.44 (m, 6H), 1.29 (m, 1H), 0.96 (t, *J* = 6.9 Hz, 3H). ^13^C{^1^H} NMR (126 MHz, C_6_D_6_): 219.5 (C), 152.5 (C), 142.7 (C), 141.5 (C), 130.2 (C), 128.0 (CH), 123.5 (CH), 123.3 (CH), 116.2 (CH), 115.5 (CH), 61.7 (CH_2_), 52.6 (CH), 50.1 (CH), 47.5 (CH), 41.9 (CH), 38.9 (CH_2_), 21.2 (CH_2_), 20.9 (CH_2_), 20.7 (CH_2_), 13.9 (CH_3_), 13.7 (CH_3_). ESI(+)-MS *m*/*z* (%): 393 (100) [M + CH_3_ONa]^+^. Anal. Calcd for C_21_H_25_NO_3_: C, 74.31; H, 7.42; N, 4.13. Found: C, 74.23; H, 7.45; N, 4.12.

#### Ethyl 7-Cyclohexyl-12-oxo-7,8,9,10,11,11a-hexahydro-5*H*-8,11-methanocycloocta[*b*]indole-5-carboxylate (**3c**)

The general procedure was followed using ethyl (*E*)-2-(2-cyclohexylvinyl)-1*H*-indole-1-carboxylate **1c** (59.5 mg, 0.2 mmol) and 2-bromocyclopentan-1-one **2a** (46.0 mg, 0.28 mmol). The purification of the crude by flash chromatography (SiO_2_, hexane/ethyl acetate 95:5) yielded **3c** (52 mg, 69%) as a white thick wax. ^1^H NMR (500 MHz, C_6_D_6_): 7.97 (d, *J* = 8.2 Hz, 1H), 7.09 (m, 1H), 6.97 (t, *J* = 3.6 Hz, 1H), 6.85 (td, *J* = 7.4, 1.0 Hz, 1H), 6.81 (m, 1H), 4.06 (m, 2H), 3.66 (m, 1H), 2.69 (dq, *J* = 7.9, 1.8 Hz, 1H), 2.45 (m, 1H), 2.17 (m, 1H), 1.74–1.59 (m, 4H), 1.56 (m, 1H), 1.48–1.39 (m, 3H), 1.33 (m, 1H), 1.18–1.05 (m, 6H), 0.99 (t, *J* = 7.1 Hz, 3H). ^13^C{^1^H} NMR (126 MHz, C_6_D_6_): 219.4 (C), 152.6 (C), 142.7 (C), 141.9 (C), 130.3 (C), 128.0 (CH), 123.5 (CH), 123.5 (CH), 116.2 (CH), 113.9 (CH), 61.7 (CH_2_), 51.6 (CH), 50.4 (CH), 48.1 (CH), 47.4 (CH), 44.3 (CH), 30.7 (CH_2_), 30.2 (CH_2_), 26.6 (CH_2_), 26.5 (CH_2_), 26.4 (CH_2_), 22.3 (CH_2_), 21.3 (CH_2_), 13.9 (CH_3_). ESI(+)-MS *m*/*z* (%): 380 (100) [M + H]^+^. Anal. Calcd for C_24_H_29_NO_3_: C, 75.96; H, 7.70; N, 3.69. Found: C, 75.84; H, 7.73; N, 3.70.

#### Ethyl 12-Oxo-7-(*p*-tolyl)-7,8,9,10,11,11a-hexahydro-5*H*-8,11-methanocycloocta[*b*]indole-5-carboxylate (**3d**)

The general procedure was followed using ethyl 2-(4-methylstyryl)-1*H*-indole-1-carboxylate **1d** (61.0 mg, 0.2 mmol) and 2-bromocyclopentan-1-one **2a** (46.0 mg, 0.28 mmol). The purification of the crude by flash chromatography (SiO_2_, hexane/ethyl acetate 9:1) yielded **3d** (60 mg 80%) as a yellow solid (mp 94–99 °C). ^1^H NMR (300 MHz, C_6_D_6_): 8.13 (d, *J* = 8.2 Hz, 1H), 7.41 (m, 1H), 7.31–7.18 (m, 3H overlapped with C_6_D_6_), 7.07 (d, *J* = 7.9 Hz, 2H), 6.98 (t, *J* = 7.6 Hz, 1H), 6.91 (d, *J* = 7.4 Hz, 1H), 4.12 (m, 2H), 3.87 (s, 2H), 2.87 (m, 1H), 2.71 (m, 1H), 2.21 (s, 3H), 1.78 (m, 1H), 1.44 (m, 2H), 1.34 (m, 1H), 1.03 (t, *J* = 7.1 Hz, 3H). ^13^C{^1^H} NMR (75 MHz, C_6_D_6_): 218.2 (C), 152.6 (C), 143.0 (C), 142.9 (C), 142.0 (C), 135.8 (C), 130.1 (C), 129.5 (2 × CH), 128.2 (CH), 127.6 (2 × CH), 123.6 (CH), 123.3 (CH), 116.3 (CH), 115.1 (CH), 61.9 (CH_2_), 56.0 (CH), 49.8 (CH), 48.1 (CH), 47.6 (CH), 21.2 (CH_2_), 21.0 (CH_2_), 20.7 (CH_3_), 14.0 (CH_3_). ESI(+)-MS *m*/*z* (%): 388 (100) [M + H]^+^. Anal. Calcd for C_25_H_25_NO_3_: C, 77.49; H, 6.50; N, 3.61. Found: C, 77.63; H, 6.52; N, 3.60.

#### Ethyl 7-(4-Fluorophenyl)-12-oxo-7,8,9,10,11,11a-hexahydro-5*H*-8,11-methanocycloocta[*b*]indole-5-carboxylate (**3e**)

The general procedure was followed using ethyl (*E*)-2-(4-fluorostyryl)-1*H*-indole-1-carboxylate **1e** (62.0 mg, 0.2 mmol) and 2-bromocyclopentan-1-one **2a** (46.0 mg, 0.28 mmol). The purification of the crude by flash chromatography (SiO_2_, hexane/ethyl acetate 9:1) yielded **3e** (55 mg, 70%) as a white solid (mp 108–112 °C). ^1^H NMR (300 MHz, C_6_D_6_): 8.08 (d, *J* = 8.2 Hz, 1H), 7.27 (m, 1H, overlapped with C_6_D_6_), 7.23 (m, 1H), 7.04 (m, 2H), 6.98 (m, 1H), 6.93–6.82 (m, 3H), 4.13 (q, *J* = 7.1 Hz, 2H), 3.84 (m, 1H), 3.73 (m 1H), 2.86 (m, 1H), 2.55 (d, *J* = 6.8 Hz, 1H), 1.71–1.56 (m, 1H), 1.50–1.19 (m, 3H), 1.03 (t, *J* = 7.1 Hz, 3H). ^13^C{^1^H} NMR (75 MHz, C_6_D_6_): 218.0 (C), 161.6 (d, *J* = 240.2 Hz, C), 152.6 (C), 142.8 (C), 142.2 (C), 141.6 (d, *J* = 3,1 Hz, C), 129.9 (C), 129.1 (d, *J* = 8,1 Hz, 2 × CH), 128.3 (CH), 123.7 (CH), 123.3 (CH), 116.3 (CH), 115.5 (d, *J* = 20.6 Hz, 2 × CH), 114.6 (CH), 62.0 (CH_2_), 55.7 (CH), 49.7 (CH), 48.1 (CH), 47.1 (CH), 21.2 (CH_2_), 20.8 (CH_2_), 14.0 (CH_3_). ESI(+)-MS *m*/*z* (%): 392 (100) [M + H]^+^. Anal. Calcd for C_24_H_22_FNO_3_: C, 73.64; H, 5.67; N, 3.58. Found: C, 73.71 H, 5.69; N, 3.59.

#### Ethyl 7-(4-Methoxyphenyl)-12-oxo-7,8,9,10,11,11a-hexahydro-5*H*-8,11-methanocycloocta[*b*]indole-5-carboxylate (**3f**)

The general procedure was followed using ethyl 2-(4-methoxystyryl)-1*H*-indole-1-carboxylate **1f** (64.0 mg, 0.2 mmol) and 2-bromocyclopentan-1-one **2a** (46.0 mg, 0.28 mmol). The purification of the crude by flash chromatography (SiO_2_, hexane/ethyl acetate 9:1) yielded **3f** (64 mg, 79%) as a yellow solid (mp 135–138 °C). ^1^H NMR (300 MHz, C_6_D_6_): 8.12 (d, *J* = 8.2 Hz, 1H), 7.41 (s, 1H), 7.26 (m, 1H, overlapped with C_6_D_6_), 7.22 (m, 2H), 6.98 (td, *J* = 7.4, 0.9 Hz, 1H), 6.92 (d, *J* = 7.5 Hz, 1H), 6.89–6.84 (m, 2H), 4.17–4.10 (m, 2H), 3.86 (m, 2H), 3.42 (s, 3H), 2.88 (m, 1H), 2.71 (d, *J* = 7.5 Hz, 1H), 1.80 (m, 1H), 1.51–1.28 (m, 3H), 1.04 (t, *J* = 7.1 Hz, 3H). ^13^C{^1^H} NMR (75 MHz, C_6_D_6_): 218.3 (C), 158.7 (C), 152.7 (C), 142.9 (C), 141.9 (C), 138.0 (C), 130.1 (C), 128.6 (2 × CH), 127.2 (CH), 123.6 (CH), 123.3 (CH), 116.3 (CH), 115.3 (CH), 114.3 (2 × CH), 61.9 (CH_2_), 56.2 (CH_3_), 54.6 (CH), 49.8 (CH), 48.1 (CH), 47.3 (CH), 21.3 (CH_2_), 21.0 (CH_2_), 14.0 (CH_3_). ESI(+)-MS *m*/*z* (%): 404 (100) [M + H]^+^. Anal. Calcd for C_25_H_25_NO_4_: C, 74.42; H, 6.25; N, 3.47. Found: C, 74.35; H, 6.27; N, 3.48.

#### Ethyl 2-Fluoro-12-oxo-7-(*p*-tolyl)-7,8,9,10,11,11a-hexahydro-5*H*-8,11-methanocycloocta[*b*]indole-5-carboxylate (**3g**)

The general procedure was followed using ethyl 5-fluoro-2-(4-methylstyryl)-1*H*-indole-1-carboxylate **1g** (64.6 mg, 0.2 mmol) and 2-bromocyclopentan-1-one **2a** (46.0 mg, 0.28 mmol). The purification of the crude by flash chromatography (SiO_2_, hexane/ethyl acetate 9:1) yielded **3g** (59 mg, 68%) as a yellow solid (mp 151–153 °C). ^1^H NMR (300 MHz, C_6_D_6_): 7.77 (dd, *J* = 9.0, 4.6 Hz, 1H), 7.18 (t, *J* = 3.2 Hz, 1H), 7.11 (m, 2H, overlapped with C_6_D_6_), 6.91 (m, 2H), 6.71 (m, 1H), 6.44 (m, 1H), 3.94 (m, 2H), 3.69 (m, 1H), 3.58 (m, 1H), 2.53 (m, 2H), 2.06 (s, 3H), 1.61 (m, 1H), 1.27 (m, 2H), 1.08 (m, 1H), 0.86 (t, *J* = 7.1, 3H). ^13^C{^1^H} NMR (75 MHz, C_6_D_6_): 217.6 (C), 158.5 (d, *J* = 242.2 Hz, C), 152.3 (C), 142.7 (C), 141.6 (C), 138.7 (C), 135.8 (C), 131.9 (d, *J* = 8,4 Hz, C), 129.4 (2 × CH), 127.4 (2 × CH), 117.1 (d, *J* = 7.8 Hz, CH), 115.3 (CH), 114.4 (d, *J* = 22.8 Hz, CH), 110.4 (d, *J* = 24.3 Hz, CH), 61.8 (CH_2_), 55.7 (CH), 49.2 (CH), 47.8 (CH), 47.4 (CH), 21.0 (CH_2_), 20.8 (CH_2_), 20.5 (CH_3_), 13.8 (CH_3_). ESI(+)-MS *m*/*z* (%): 428 (100) [M + Na]^+^. Anal. Calcd for C_25_H_24_FNO_3_: C, 74.06; H, 5.97; N, 3.45. Found: C, 73.88; H, 5.99; N, 3.44.

#### Ethyl 2-Methoxy-12-oxo-7-(*p*-tolyl)-7,8,9,10,11,11a-hexahydro-5*H*-8,11-methanocycloocta[*b*]indole-5-carboxylate (**3h**)

The general procedure was followed using ethyl 5-methoxy-2-(4-methylstyryl)-1*H*-indole-1-carboxylate **1h** (67.0 mg, 0.2 mmol) and 2-bromocyclopentan-1-one **2a** (46.0 mg, 0.28 mmol). The purification of the crude by flash chromatography (SiO_2_, hexane/ethyl acetate 95:5) yielded **3h** (57 mg, 70%) as a white solid (mp 83–85 °C). ^1^H NMR (300 MHz, C_6_D_6_): 7.91 (d, *J* = 8.9 Hz, 1H), 7.28 (m, 1H), 7.12 (m, 2H overlapped with C_6_D_6_), 6.92 (d, *J* = 7.8 Hz, 2H), 6.69 (m, 1H), 6.59 (m, 1H), 4.00 (m, 2H), 3.73 (m, 2H), 3.26 (s, 3H), 2.72 (m, 1H), 2.56 (m, 1H), 2.06 (s, 3H), 1.66 (dt, *J* = 10.6, 4.7 Hz, 1H), 1.38–1.17 (m, 3H), 0.91 (t, *J* = 7.1 Hz, 3H). ^13^C{^1^H} NMR (75 MHz, C_6_D_6_): 218.0 (C), 156.7 (C), 152.4 (C), 142.9 (C), 142.1 (C), 136.3 (C), 135.7 (C), 131.3 (C), 129.3 (2 × CH), 127.4 (2 × CH), 116.9 (CH), 114.9 (CH), 113.3 (CH), 109.0 (CH), 61.6 (CH_2_), 55.8 (CH), 54.8 (CH), 49.7 (CH), 48.2 (CH), 47.5 (CH_3_), 21.1 (CH_2_), 20.9 (CH_2_), 20.5 (CH_3_), 13.9 (CH_3_). ESI(+)-MS *m*/*z* (%): 418 (100) [M + H]^+^. Anal. Calcd for C_26_H_27_NO_4_: C, 74.80; H, 6.52; N, 3.35. Found: C, 74.92; H, 6.51; N, 3.34.

#### Ethyl 7,8,10-Trimethyl-9-oxo-8,9,10,10a-tetrahydrocyclohepta[*b*]indole-5(7*H*)-carboxylate (**3i**)

The general procedure was followed using ethyl 2-(4-methylstyryl)-1*H*-indole-1-carboxylate **1d** (61.0 mg, 0.2 mmol) and 2-bromo-5-methylcyclopentan-1-one **2b** (49.5 mg, 0.28 mmol) for 24 at rt. TFE (0.2 mL, 1 M) was used as the solvent instead of toluene. The purification of the crude by column chromatography (SiO_2_, hexane/ethyl acetate 9:1) yielded **3i** (58 mg 72%) as a white thick wax. ^1^H NMR (500 MHz, C_6_D_6_): 8.02 (d, *J* = 8.2 Hz, 1H), 7.22 (t, *J* = 3.2 Hz, 1H), 7.12 (m, 1H), 7.03 (br s, 2H), 6.93 (d, *J* = 7.9 Hz, 2H), 6.89–6.86 (m, 2H), 3.98 (m, 2H), 3.86 (d, *J* = 2.6 Hz, 1H), 3.23 (t, *J* = 3.1 Hz, 1H), 2.91 (m, 1H), 2.15 (m, 1H), 2.11 (s, 3H), 1.35 (m, 1H), 1.22 (m, 1H), 1.10 (m, 1H), 0.92–0.86 (m, *J* = 12.4, 5.2 Hz, 6H). ^13^C{^1^H} NMR (126 MHz, C_6_D_6_): 220.4 (C), 152.5 (C), 142.9 (C), 141.6 (C), 140.8 (C), 135.9 (C), 130.1 (C), 128.9 (2 × CH), 128.1 (2 × CH), 128.0 (CH), 123.5 (CH), 123.1 (CH), 117.4 (CH), 116.2 (CH), 61.7 (CH_2_), 53.8 (C), 52.2 (CH), 50.6 (CH), 48.1 (CH), 30.0 (CH_2_), 21.7 (CH_3_), 20.6 (CH_3_), 19.6 (CH_2_), 13.8 (CH_3_). ESI(+)-MS *m*/*z* (%): 402 (100) [M + H]^+^. Anal. Calcd for C_26_H_27_NO_3_: C, 77.78; H, 6.78; N, 3.49. Found: C, 77.62; H, 6.76; N, 3.50.

#### Ethyl 7,8,10-Trimethyl-9-oxo-8,9,10,10a-tetrahydrocyclohepta[*b*]indole-5(7*H*)-carboxylate (**3j**)

The general procedure was followed using ethyl (*E*)-2-(prop-1-en-1-yl)-1*H*-indole-1-carboxylate **1a** (51.5 mg, 0.2 mmol) and 2-bromopentan-3-one **2c** (46.0 mg, 0.28 mmol) for 24 h at rt. TFE (0.2 mL, 1 M) was used as the solvent instead of toluene. The purification of the crude by column chromatography (SiO_2_, hexane/ethyl acetate 9:1) yielded **3j** (49 mg 78%) as a white thick wax. ^1^H NMR (300 MHz, C_6_D_6_): 8.12 (d, *J* = 8.3 Hz, 1H), 7.24 (m, 1H overlapped with C_6_D_6_), 7.09 (d, *J* = 7.4 Hz, 1H), 6.96 (td, *J* = 7.4, 0.8 Hz, 1H), 6.74 (dd, *J* = 5.8, 2.2 Hz, 1H), 4.16 (q, *J* = 7.1 Hz, 2H), 3.51 (m, 1H), 2.48–2.28 (m, 2H), 2.06 (m, 1H), 1.25 (d, *J* = 7.1 Hz, 3H), 1.15 (d, *J* = 7.0 Hz, 3H), 1.19–1.05 (m, 6H).

^13^C{^1^H} NMR (75 MHz, C_6_D_6_): 212.2 (C), 152.3 (C), 143.8 (C), 142.7 (C), 130.0 (C), 128.2 (CH), 125.8 (CH), 122.8 (CH), 117.1 (CH), 116.3 (CH), 61.9 (CH_2_), 55.5 (CH), 53.2 (CH), 46.2 (CH), 35.9 (CH), 19.5 (CH_3_), 15.6 (CH_3_), 14.8 (CH_3_), 14.0 (CH_3_). ESI(+)-MS *m*/*z* (%): 314 (100) [M + H]^+^. Anal. Calcd for C_19_H_23_NO_3_: C, 72.82; H, 7.40; N, 4.47. Found: C, 72.69; H, 7.38; N, 4.46.

#### Ethyl 7-Methyl-9-oxo-8,10-diphenyl-8,9,10,10a-tetrahydrocyclohepta[*b*]indole-5(7*H*)-carboxylate (**3k**)

The general procedure was followed using ethyl (*E*)-2-(prop-1-en-1-yl)-1*H*-indole-1-carboxylate **1a** (46.0 mg, 0.2 mmol) and 1-chloro-1,3-diphenylpropan-2-one **2d** (63.0 mg, 0.28 mmol) for 24 h at rt. TFE (0.2 mL, 1 M) was used as the solvent instead of toluene. The purification of the crude by column chromatography (SiO_2_, hexane/ethyl acetate 95:5) yielded **3k** (48 mg 55%) as a a yellow thick wax.

^1^H NMR (300 MHz, C_6_D_6_): 7.99 (d, *J* = 8.3 Hz, 1H), 7.09–6.97 (m, 6H), 6.93–6.82 (m, 5H), 6.68 (dd, *J* = 5.8, 2.4 Hz, 1H), 6.55 (td, *J* = 7.5, 1.0 Hz, 1H), 6.46 (dd, *J* = 6.9, 0.7 Hz, 1H), 4.74 (dd, *J* = 11.0, 2.1 Hz, 1H), 4.05 (q, *J* = 7.1 Hz, 2H), 3.80 (d, *J* = 11.0 Hz, 1H), 3.69 (d, *J* = 11.3 Hz, 1H), 3.16 (m, 1H), 1.06–0.91 (m, 6H). ^13^C{^1^H} NMR (75 MHz, C_6_D_6_): 205.8 (C), 152.3 (C), 142.5 (C), 142.3 (C), 136.8 (C), 136.2 (C), 130.9 (C), 129.1 (2 × CH), 128.6 (4 × CH), 128.1 (CH), 128.0 (2 × CH), 126.9 (CH), 126.5 (CH), 124.1 (CH), 123.0 (CH), 116.0 (CH), 115.0 (CH), 66.8 (CH), 65.2 (CH), 61.9 (CH_2_), 43.1 (CH), 32.5 (CH), 20.1 (CH_3_), 13.9 (CH_3_). ESI(+)-MS *m*/*z* (%): 438 (100) [M + H]^+^. Anal. Calcd for C_29_H_27_NO_3_: C, 79.61; H, 6.22; N, 3.20. Found: C, 79.75; H, 6.23; N, 3.21.

#### Ethyl 7,10,10-Trimethyl-9-oxo-8,9,10,10a-tetrahydrocyclohepta[*b*]indole-5(7*H*)-carboxylate (**3l**)

The general procedure was followed using ethyl (*E*)-2-(pent-1-en-1-yl)-1*H*-indole-1-carboxylate **1a** (46.0 mg, 0.2 mmol) and 1-bromo-3-methylbutan-2-one **2e** (46.0 mg, 0.28 mmol) for 24 h at 40 °C. TFE (0.2 mL, 1 M) was used as the solvent instead of toluene, while Na_2_CO_3_ (32.0 mg, 0.3 mmol) was used as the base. The purification of the crude by column chromatography (SiO_2_, hexane/ethyl acetate 95:5) yielded **3l** (23 mg, 37%) as a yellow thick wax. ^1^H NMR (300 MHz, C_6_D_6_): 8.11 (d, *J* = 8.3 Hz, 1H), 7.25 (m, 1H, overlapped with C_6_D_6_), 7.09 (d, *J* = 7.6 Hz, 1H), 6.98 (t, *J* = 7.5 Hz, 1H), 6.70 (s, 1H), 4.14 (q, *J* = 7.1 Hz, 2H), 3.93 (s, 1H), 2.98 (dd, *J* = 11.7, 5.7 Hz, 1H), 2.62 (br s, 1H), 2.22 (dd, *J* = 11.8, 5.3 Hz, 1H), 1.30–1.14 (m, 6H), 1.06 (t, *J* = 7.1 Hz, 3H), 0.92 (s, 3H). ^13^C{^1^H} NMR (75 MHz, C_6_D_6_): 211.0 (C), 152.4 (C), 143.5 (C), 141.0 (C), 128.6 (C), 128.2 (CH), 125.7 (CH), 122.7 (CH), 116.4 (CH), 115.7 (CH), 61.8 (CH_2_), 54.5 (C), 49.0 (CH), 45.1 (CH_2_), 31.2 (CH), 23.6 (CH_3_), 23.6 (CH_3_), 17.4 (CH_3_), 13.9 (CH_3_). ESI(+)-MS *m*/*z* (%): 314 (100) [M + H]^+^. Anal. Calcd for C_19_H_23_NO_3_: C, 72.82; H, 7.40; N, 4.47. Found: C, 72.63; H, 7.38; N, 4.48.

#### *Tert*-Butyl 7-Methyl-12-oxo-7,8,9,10,11,11a-hexahydro-5*H*-8,11-methanocycloocta[*b*]indole-5-carboxylate (**3m**)

The general procedure was followed using *tert*-butyl (*E*)-2-(prop-1-en-1-yl)-1*H*-indole-1-carboxylate **1i** (51.5 mg, 0.2 mmol) and 2-bromocyclopentan-1-one **2a** (46.0 mg, 0.28 mmol). The purification of the crude by column chromatography (SiO_2_, hexane/ethyl acetate 95:5) yielded **3m** (49 mg, 72%) as a white solid (mp 149–154 °C). ^1^H NMR (300 MHz, C_6_D_6_): 8.08 (d, *J* = 8.2 Hz, 1H), 7.22 (m, 1H), 6.95 (t, *J* = 7.4 Hz, 1H), 6.88 (d, *J* = 7.6 Hz, 1H), 6.83 (t, *J* = 3.1 Hz, 1H), 3.79 (s, 1H), 2.80 (m, 1H), 2.62 (m, 1H), 2.24 (s, 1H), 1.53 (s, 9H), 1.43 (m, 4H), 1.09 (d, *J* = 7.2 Hz, 3H). ^13^C{^1^H} NMR (75 MHz, C_6_D_6_): 219.3 (C), 151.5 (C), 143.1 (C), 141.5 (C), 130.3 (C), 128.0 (CH), 123.4 (CH), 123.3 (CH), 116.7 (CH), 116.3 (CH), 81.9, (C) 54.4 (CH), 49.5 (CH), 47.7 (CH), 36.8 (CH), 27.9 (3 × CH_3_), 22.9 (CH_3_), 21.2 (CH_2_), 20.5 (CH_2_). ESI(+)-MS *m*/*z* (%): 361 (65) [M + Na]^+^. Anal. Calcd for C_21_H_25_NO_3_: C, 74.31; H, 7.42; N, 4.13. Found: C, 74.57; H, 7.44; N, 4.15.

### Preparation and Characterization Data for Compounds **4a–b**

#### Ethyl (*E*)-3-(2-Oxocyclopentyl)-2-(prop-1-en-1-yl)-1*H*-indole-1-carboxylate (**4a**)

**4a** was isolated during the screening of reaction conditions (see Table 1) as a secondary product by reacting ethyl (*E*)-2-(prop-1-en-1-yl)-1*H*-indole-1-carboxylate **1a** (46.0 mg, 0.2 mmol) and 2-bromocyclopentan-1-one **2a** (46.0 mg, 0.28 mmol) in a fluorinated alcohol (TFE or HFIP, 0.2 mL, 1 M) and in the presence of a base (1.5 equiv) at the temperature and for the time stated in Table 1. The removal of the solvent and purification of the crude by column chromatography (SiO_2_, hexane/ethyl acetate 95:5) yielded progressively **3a** (see previous section for characterization) and **4a**. ^1^H NMR (300 MHz, CD_2_Cl_2_): 8.18 (dt, *J* = 8.3, 0.9 Hz, 1H), 7.30 (m, 1H), 7.23–7.18 (m, 2H), 6.66 (dq, *J* = 15.8, 1.8 Hz, 1H), 5.86 (dq, *J* = 15.8, 6.6 Hz, 1H), 4.49 (q, *J* = 7.1 Hz, 2H), 3.70 (m, 1H), 2.62–2.22 (m, 5H), 2.04 (m, 1H), 1.97 (dd, *J* = 6.6, 1.8 Hz, 3H), 1.49 (t, *J* = 7.1 Hz, 3H). ^13^C{^1^H} NMR (75 MHz, CD_2_Cl_2_): 218.2 (C), 151.7 (C), 137.5 (C), 135.6 (C), 130.8 (CH), 128.0 (C), 124.1 (CH), 122.8 (CH), 122.4 (CH), 119.2 (CH), 116.4 (C), 115.9 (CH), 63.1 (CH_2_), 47.8 (CH), 38.5 (CH_2_), 30.9 (CH_2_), 21.3 (CH_2_), 18.3 (CH_3_), 14.1 (CH_3_). ESI(+)-MS *m*/*z* (%): 334 (100) [M + Na]^+^. Anal. Calcd for C_19_H_21_NO_3_: C, 73.29; H, 6.80; N, 4.50. Found: C, 73.17; H, 6.78; N, 4.52.

#### (*E*)-2-(1-Methyl-2-(prop-1-en-1-yl)-1*H*-indol-3-yl)cyclopentan-1-one (**4b**)

The general procedure employed for the synthesis of **3a–m** was followed using (*E*)-1-methyl-2-(prop-1-en-1-yl)-1*H*-indole **1j** (34 mg, 0.2 mmol) and 2-bromocyclopentan-1-one **2a** (46 mg, 0.28 mmol). The purification of the crude by column chromatography (SiO_2_, hexane/ethyl acetate 95:5) yielded **4b** (28 mg, 55%) as a thick wax. ^1^H NMR (300 MHz, CDCl_3_): 7.28–7.22 (dd, *J* = 7.5, 4.1 Hz, 2H), 7.17 (m, 1H), 7.02 (m, 1H), 6.39 (dd, *J* = 15.9, 1.7 Hz, 1H), 6.00 (dq, *J* = 15.8, 6.6 Hz, 1H), 3.75–3.54 (m, 4H), 2.63–2.47 (m, 2H), 2.42–2.31 (m, 2H), 2.25 (m, 1H), 2.06–1.88 (m, 4H). ^13^C{^1^H} NMR (75 MHz, CDCl_3_): 219.6 (C), 137.2 (C), 137.0 (C), 132.9 (CH), 125.8 (C), 121.5 (CH), 120.2 (CH), 119.1 (CH), 119.0 (CH), 109.4 (CH), 109.0 (C), 48.1 (CH), 38.6 (CH_2_), 31.6 (CH_2_), 30.4 (CH_3_), 21.4 (CH_2_), 19.1 (CH_3_). ESI(+)-MS *m*/*z* (%): 252 (65) [M – H]^−^. Anal. Calcd. for C_17_H_19_NO: C, 80.60; H, 7.56; N, 5.53. Found: C, 80.83; H, 7.58; N, 5.54.

### General Procedure for the Reaction between 4*H*-Furo[3,2-*b*]indole **5a–e** and α-haloketones **2a,c–e**

To a stirring solution of 4*H*-furo[3,2-*b*]indole **5a–e** (0.2 mmol, 1.0 equiv), α-haloketone **2a,c–e** (0.28 mmol, 1.4 equiv), and TFE (86.4 μL, 1.2 mmol, 6.0 equiv) in toluene (0.4 mL, 0.5 M), DIPEA (52.3 μL, 0.3 mmol, 1.5 equiv) was added, and the mixture was stirred for 2–3 h at room temperature. The Solvent was then removed, and the crude was purified by column chromatography to yield the corresponding cyclohepta[*b*]indoline **6a–h**.

#### Ethyl 13-Oxo-8,9,10,11-tetrahydro-7,11a-epoxy-8,11-methanocycloocta[*b*]indole-5(7*H*)-carboxylate (**6a**)

The general procedure was followed using ethyl 4*H*-furo[3,2-*b*]indole-4-carboxylate **5a** (46.0 mg, 0.2 mmol) and 2-bromocyclopentan-1-one **2a** (46.0 mg, 0.28 mmol) for 3 h at rt. The purification of the crude by flash chromatography (SiO_2_, hexane/ethyl acetate 95:5) yielded **6a** (48 mg, 77%) as a yellow solid (mp 116–118 °C). ^1^H NMR (300 MHz, CDCl_3_): 7.98 (d, *J* = 6.4 Hz, 1H), 7.48 (m, 1H), 7.41 (td, *J* = 8.2, 1.2 Hz, 1H), 7.14 (td, *J* = 7.5, 0.6 Hz, 1H), 5.81 (s, 1H), 4.99 (dd, *J* = 4.2, 2.2 Hz, 1H), 4.40 (m, 2H), 2.67 (m, 1H), 2.42–2.27 (m, 2H), 2.15 (m, 1H), 1.98–1.87 (m, 2H), 1.41 (t, *J* = 7.1 Hz, 3H). ^13^C{^1^H} NMR (75 MHz, CDCl_3_): 208.2 (C), 151.3 (C), 150.6 (C), 145.3 (C), 130.6 (CH), 128.4 (C), 124.2 (CH), 123.3 (CH), 116.3 (CH), 107.3 (CH), 91.9 (C), 87.4 (CH), 62.9 (CH_2_), 56.7 (CH), 51.0 (CH), 22.4 (CH_2_), 21.1 (CH_2_), 14.4 (CH_3_). ESI(+)-MS *m*/*z* (%): 312 (100) [M + H]^+^. Anal. Calcd for C_18_H_17_NO_4_: C, 69.44; H, 5.50; N, 4.50. Found: C, 69.34; H, 5.48; N, 4.52.

#### Ethyl 2-Fluoro-13-oxo-8,9,10,11-tetrahydro-7,11a-epoxy-8,11-methanocycloocta[*b*]indole-5(7*H*)-carboxylate (**6b**)

The general procedure was followed using ethyl 7-fluoro-4*H*-furo[3,2-*b*]indole-4-carboxylate **5b** (50.0 mg, 0.2 mmol) and 2-bromocyclopentan-1-one **2a** (46.0 mg, 0.28 mmol) for 2 h at rt. The purification of the crude by flash chromatography (SiO_2_, hexane/ethyl acetate 95:5) yielded **6b** (46 mg, 70%) as a yellow solid (mp 136–140 °C). ^1^H NMR (300 MHz, C_6_D_6_): 7.99 (br s, 1H), 6.88 (dd, *J* = 7.5, 2.7 Hz, 1H), 6.66 (td, *J* = 9.0, 2.8 Hz, 1H), 5.45 (br s, 1H), 4.33 (dd, *J* = 4.3, 2.2 Hz, 1H), 3.81 (m, 2H), 2.19 (m, 1H), 2.04 (m, 1H), 1.80 (m, 1H), 1.65 (m, 1H), 1.50–1.23 (m, 2H), 0.79 (t, *J* = 7.1 Hz, 3H). ^13^C{^1^H} NMR (75 MHz, C_6_D_6_): 205.0 (C), 159.3 (d, *J* = 243.6 Hz, C), 150.8 (C), 150.6 (C), 141.4 (C), 130.3 (d, *J* = 7.7 Hz, C), 117.3 (d, *J* = 8.1 Hz, CH), 116.5 (d, *J* = 23.2 Hz, CH), 110.8 (d, *J* = 24.5 Hz, CH), 107.3 (CH), 91.19 (C), 87.0 (CH), 62.31 (CH_2_), 56.1 (CH), 50.7 (CH), 22.2 (CH_2_), 20.8 (CH_2_), 13.7 (CH_3_). ESI(+)-MS *m*/*z* (%): 328 (100) [M – H]^−^. Anal. Calcd for C_18_H_16_FNO_4_: C, 65.65; H, 4.90; N, 4.25. Found: C, 65.82; H, 4.92; N, 4.24.

#### Ethyl 2-Methyl-13-oxo-8,9,10,11-tetrahydro-7,11a-epoxy-8,11-methanocycloocta[*b*]indole-5(7*H*)-carboxylate (**6c**)

The general procedure was followed using ethyl 7-methyl-4*H*-furo[3,2-*b*]indole-4-carboxylate **5c** (49.0 mg, 0.2 mmol) and 2-bromocyclopentan-1-one **2a** (46.0 mg, 0.28 mmol) for 2 h at rt. The purification of the crude by flash chromatography (SiO_2_, hexane/ethyl acetate 95:5 to 9:1) yielded **6c** (52 mg, 80%) as an orange solid (mp 114–117 °C). ^1^H NMR (300 MHz, C_6_D_6_): 8.16 (br s, 1H), 7.07 (m, 1H), 6.85 (m, 1H), 5.53 (br s, 1H), 4.42 (dd, *J* = 4.2, 2.2 Hz, 1H), 3.84 (m, 2H), 2.31–2.13 (m, 2H), 2.04 (m, 1H), 1.93 (s, 3H), 1.75 (m, 1H), 1.54–1.34 (m, 2H), 0.81 (t, *J* = 7.1 Hz, 3H).

^13^C{^1^H} NMR (75 MHz, C_6_D_6_): 205.5 (C), 150.98 (C), 143.5 (C), 133.4 (C), 130.7 (CH), 129.1 (C), 126.7 (C), 123.9 (CH), 116.0 (CH), 106.9 (CH), 91.8 (C), 87.0 (CH), 62.2 (CH_2_), 56.5 (CH), 50.7 (CH), 22.3 (CH_2_), 21.0 (CH_2_), 20.4 (CH_3_), 13.7 (CH_3_). ESI(+)-MS *m*/*z* (%): 326 (100) [M + H]^+^. Anal. Calcd for C_19_H_19_NO_4_: C, 70.14; H, 5.89; N, 4.31. Found: C, 69.92; H, 5.90; N, 4.29.

#### Ethyl 2-Methoxy-13-oxo-8,9,10,11-tetrahydro-7,11a-epoxy-8,11-methanocycloocta[*b*]indole-5(7*H*)-carboxylate (**6d**)

The general procedure was followed using ethyl 7-methoxy-4*H*-furo[3,2-*b*]indole-4-carboxylate **5d** (52.0 mg, 0.2 mmol) and 2-bromocyclopentan-1-one **2a** (46.0 mg, 0.28 mmol) for 2 h at rt. The purification of the crude by flash chromatography (SiO_2_, hexane/ethyl acetate 9:1 to 8:2) yielded **6d** (50 mg, 73%) as a white solid (mp 118–122 °C). ^1^H NMR (300 MHz, CDCl_3_): 7.86 (br s, 1H), 6.99 (d, *J* = 2.7 Hz, 1H), 6.90 (dd, *J* = 9.0, 2.7 Hz, 1H), 5.74 (br s, 1H), 4.96 (dd, *J* = 4.2, 2.2 Hz, 1H), 4.36 (m, 2H), 3.80 (s, 3H), 2.64 (m, 1H), 2.36 (m, 1H), 2.27 (m, 1H), 2.09 (m, 1H), 2.02–1.82 (m, 2H), 1.37 (t, *J* = 7.1 Hz, 3H). ^13^C{^1^H} NMR (75 MHz, CDCl_3_): 208.0 (C), 156.5 (C), 151.3 (C), 150.8 (C), 138.8 (C), 129.4 (C), 116.9 (CH), 115.4 (CH), 109.3 (CH), 106.9 (CH), 91.7 (C), 87.4 (CH), 62.7 (CH_2_), 56.5 (CH_3_), 55.7 (CH), 50.9 (CH), 22.3 (CH_2_), 21.1 (CH_2_), 14.4 (CH_3_). ESI(+)-MS *m*/*z* (%): 363 (100) [M + Na]^+^. Anal. Calcd for C_19_H_19_NO_5_: C, 66.85; H, 5.61; N, 4.10. Found: C, 66.76; H, 5.63; N, 4.11.

#### Ethyl 7-Methyl-13-oxo-8,9,10,11-tetrahydro-7,11a-epoxy-8,11-methanocycloocta[*b*]indole-5(7*H*)-carboxylate (**6e**)

The general procedure was followed using ethyl 2-methyl-4*H*-furo[3,2-*b*]indole-4-carboxylate **5e** (49.0 mg, 0.2 mmol) and 2-bromocyclopentan-1-one **2a** (46.0 mg, 0.28 mmol) for 3 h at rt. The purification of the crude by flash chromatography (SiO_2_, hexane/ethyl acetate 9:1 to 8:2) yielded **6e** (50 mg, 77%) as a white solid (mp 155–160 °C). ^1^H NMR (300 MHz, CDCl_3_): 7.95 (br s, 1H), 7.43 (m, 1H), 7.37 (m, 1H), 7.10 (td, *J* = 7.5, 0.9 Hz, 1H), 5.63 (br s, 1H), 4.36 (m, 2H), 2.45 (m, 1H), 2.30 (m, 1H), 2.21 (m, 1H), 2.10 (m, 1H), 1.95–1.80 (m, 2H), 1.50 (s, 3H), 1.39 (t, *J* = 7.1 Hz, 3H). ^13^C{^1^H} NMR (75 MHz, CDCl_3_): 208.6 (C), 151.3 (C), 150.0 (C), 145.0 (C), 130.5 (CH), 128.6 (C), 124.2 (CH), 123.2 (CH), 116.2 (CH), 110.6 (CH), 93.5 (C), 91.3 (C), 62.8 (CH_2_), 55.4 (CH), 54.5 (CH), 21.3 (CH_2_), 21.1 (CH_3_), 19.8 (CH_2_), 14.4 (CH_3_). ESI(+)-MS *m*/*z* (%): 348 (100) [M + Na]^+^. Anal. Calcd for C_19_H_19_NO_4_: C, 70.14; H, 5.89; N, 4.31. Found: C, 70.33; H, 5.88; N, 4.30.

#### Ethyl 8,10-Dimethyl-9-oxo-7,8,9,10-tetrahydro-5*H*-7,10a-epoxycyclohepta[*b*]indole-5-carboxylate (**6f**)

The general procedure was followed using ethyl 4*H*-furo[3,2-*b*]indole-4-carboxylate **5a** (46.0 mg, 0.2 mmol) and 2-bromopentan-3-one **2c** (46 mg, 0.28 mmol) for 24 at rt. TFE (0.2 mL, 1 M) was used as the solvent instead of toluene. The purification of the crude by column chromatography (SiO_2_, hexane/ethyl acetate 9:1) yielded **6f** (49 mg 78%) as an orange solid (mp 102–105 °C). ^1^H NMR (300 MHz, CDCl_3_): 7.89 (d, *J* = 7.0 Hz, 1H), 7.52–7.33 (m, 2H), 7.17 (td, *J* = 7.5, 0.8 Hz, 1H), 5.83 (br s, 1H), 5.13 (dd, *J* = 4.8, 2.5 Hz, 1H), 4.37 (m, 2H), 3.28–2.86 (m, 2H), 1.40 (t, *J* = 7.1 Hz, 3H), 1.11 (d, *J* = 7.0 Hz, 3H), 0.62 (d, *J* = 7.2 Hz, 3H). ^13^C{^1^H} (75 MHz, CDCl_3_) 207.8 (C), 151.3 (C), 150.3 (C), 145.9 (C), 130.4 (CH), 127.3 (C), 124.5 (CH), 123.8 (CH), 115.8 (CH), 107.6 (CH), 92.4 (C), 88.3 (CH), 62.9 (CH_2_), 54.7 (CH), 49.9 (CH), 14.4 (CH_3_), 11.0 (CH_3_), 8.7 (CH_3_). ESI(+)-MS *m*/*z* (%): 314 (100) [M + H]^+^. Anal. Calcd for C_18_H_19_NO_4_: C, 69.00; H, 6.11; N, 4.47. Found: C, 68.86; H, 6.12; N, 4.45.

#### Ethyl 9-Oxo-8,10-diphenyl-7,8,9,10-tetrahydro-5*H*-7,10a-epoxycyclohepta[*b*]indole-5-carboxylate (**6g**)

The general procedure was followed using ethyl 4*H*-furo[3,2-*b*]indole-4-carboxylate **5a** (46.0 mg, 0.2 mmol) and 1-chloro-1,3-diphenylpropan-2-one **2d** (63 mg, 0.28 mmol) for 24 h at rt. TFE (0.2 mL, 1 M) was used as the solvent instead of toluene. The purification of the crude by column chromatography (SiO_2_, hexane/ethyl acetate 95:5) yielded **6g** (83 mg 95%) as a yellow solid (mp 202–204 °C). ^1^H NMR (300 MHz, CDCl_3_): 7.50 (m, 1H), 7.45–7.28 (m, 6H), 7.19–6.95 (m, 5H), 6.85–6.75 (m, 2H), 5.97 (br s, 1H), 5.39 (dd, *J* = 4.9, 2.5 Hz, 1H), 4.44 (d, *J* = 4.9 Hz, 1H), 4.38 (qd, *J* = 7.1, 2.2 Hz, 2H), 4.24 (s, 1H), 1.41 (t, *J* = 7.1 Hz, 3H). ^13^C{^1^H} (75 MHz, CDCl_3_): 205.3 (C), 150.7 (C), 150.1 (C), 145.1 (C), 135.4 (C), 133.3 (C), 130.3 (CH), 129.9 (2 × CH), 129.8 (CH), 128.6 (2 × CH), 127.6 (CH), 127.4 (2 × CH), 127.1 (CH), 126.9 (C), 123.9 (CH), 123.9 (CH), 115.3 (CH), 108.9 (CH), 92.6 (C), 88.6 (CH), 65.8 (CH), 62.7 (CH_2_), 61.5 (CH), 14.5 (CH_3_). CH_sp^2^_ is overlapped with another CH_sp^2^_. ESI(−)-MS *m*/*z* (%): 436 (50) [M + H]^+^. Anal. Calcd for C_28_H_23_NO_4_: C, 76.87; H, 5.30; N, 3.20. Found: C, 77.05; H, 5.31; N, 3.21.

#### Ethyl 10,10-Dimethyl-9-oxo-7,8,9,10-tetrahydro-5*H*-7,10a-epoxycyclohepta[*b*]indole-5-carboxylate (**6h**)

The general procedure was followed using ethyl 4*H*-furo[3,2-*b*]indole-4-carboxylate **5a** (46.0 mg, 0.2 mmol) and 1-bromo-3-methylbutan-2-one **2e** (46 mg, 0.28 mmol) for 48 h at 40 °C. TFE (0.2 mL, 1 M) was used as the solvent instead of toluene, while Na_2_CO_3_ (32 mg, 0.3 mmol) was used as the base. The purification of the crude by column chromatography (SiO_2_, hexane/ethyl acetate 95:5) yielded **6h** (36 mg 57%) as a yellow thick wax. ^1^H NMR (300 MHz, CDCl_3_): 7.94 (d, *J* = 7.9 Hz, 1H), 7.49–7.35 (m, 2H), 7.15 (td, *J* = 7.5, 0.6 Hz, 1H), 5.84 (s, 1H), 5.28 (m, 1H), 4.39 (m, 2H), 3.24 (dd, *J* = 16.2, 4.9 Hz, 1H), 2.58 (dd, *J* = 16.2, 0.8 Hz, 1H), 1.47–1.37 (m, 6H), 0.71 (s, 3H). ^13^C{^1^H} (75 MHz, CDCl_3_): 210.0 (C), 151.3 (C), 150.4 (C), 146.2 (C), 130.4 (CH), 126.0 (C), 124.8 (CH), 123.8 (CH), 115.6 (CH), 109.6 (CH), 92.8 (C), 83.2 (CH), 62.8 (CH_2_), 56.5 (C), 44.2 (CH_2_), 21.0 (CH_3_), 17.4 (CH_3_), 14.4 (CH_3_). ESI(+)-MS *m*/*z* (%): 314 (100) [M + H]^+^. Anal. Calcd for C_18_H_19_NO_4_: C, 69.00; H, 6.11; N, 4.47. Found: C, 68.87; H, 6.09; N, 4.47.

### General Procedure for the Reaction between **1a** or **5a** and *N*-(Benzyloxy)-2-bromo-2-methylpropanamide **7**

To a stirring solution of 2-vinylindole **1a** or 4*H*-furo[3,2-*b*]indole **5a** (0.2 mmol, 1.0 equiv) and *N*-(benzyloxy)-2-bromo-2-methylpropanamide **7** (82 mg, 0.3 mmol, 1.5 equiv) in HFIP (0.84 mL, 0.25 M), DIPEA (52.3 μL, 0.3 mmol, 1.5 equiv) was added, and the mixture was stirred for 1 at room temperature. The solvent was then removed, and the crude was purified by column chromatography to yield the corresponding products **8–10**.

#### Ethyl 3-(Benzyloxy)-1,1,4-trimethyl-2-oxo-2,3,4,10b-tetrahydroazepino[4,5-*b*]indole-6(1*H*)-carboxylate (**8**) and Ethyl (*E*)-1-(Benzyloxy)-3,3-dimethyl-2-oxo-8a-(prop-1-en-1-yl)-2,3,3a,8a-tetrahydropyrrolo[2,3-*b*]indole-8(1*H*)-carboxylate (**9**)

The general procedure was followed using ethyl (*E*)-2-(prop-1-en-1-yl)-1*H*-indole-1-carboxylate **1a** (46.0 mg, 0.2. mmol) and *N*-(benzyloxy)-2-bromo-2-methylpropanamide **7** (82.0 mg, 0.3 mmol). The purification of the crude by flash chromatography (SiO_2_, hexane/ethyl acetate 9:1) yielded progressively **9** (34 mg, 40%) and **8** (35 mg, 42%) as clear thick oils. **8**: ^1^H NMR (300 MHz, C_6_D_6_): 8.08 (d, *J* = 8.3 Hz, 1H), 7.60–7.47 (m, 2H), 7.32–7.18 (m, 4H), 7.11 (d, *J* = 7.6 Hz, 1H), 6.96 (td, *J* = 7.5, 1.0 Hz, 1H), 6.68 (dd, *J* = 4.6, 2.1 Hz, 1H), 4.96 (d, *J* = 10.5 Hz, 1H), 4.84 (d, *J* = 10.5 Hz, 1H), 4.41 (m, 1H), 4.26–4.00 (m, 3H), 1.57 (s, 3H), 1.46 (d, *J* = 6.7 Hz, 3H), 1.05 (dd, *J* = 8.9, 5.3 Hz, 6H). ^13^C{^1^H} (75 MHz, C_6_D_6_): 179.3 (C), 152.2 (C), 143.5 (C), 143.2 (C) 136.8 (C), 129.4 (2 × CH), 128.3 (C), 128.3 (CH), 128.3 (2 × CH), 128.2 (CH), 126.5 (CH), 122.9 (CH), 116.0 (CH), 110.8 (CH), 76.2 (CH_2_), 62.1 (CH_2_), 57.6 (CH), 51.2 (C), 48.7 (CH), 26.2 (CH_3_), 19.9 (CH_3_), 19.8 (CH_3_), 13.9 (CH_3_). ESI(+)-MS *m*/*z* (%): 421 (100) [M + H]^+^. Anal. Calcd for C_25_H_28_N_2_O_4_: C, 71.41; H, 6.71; N, 6.66. Found: C, 71.34; H, 6.73; N, 6.64.

**9**: ^1^H NMR (300 MHz, C_6_D_6_): 8.29 (d, *J* = 8.2 Hz, 1H), 7.30 (m, 1H), 7.14–6.94 (m, 5H), 6.82–6.68 (m, 2H), 5.68 (dq, *J* = 15.5, 6.2 Hz, 1H), 5.56 (dd, *J* = 15.5, 1.2 Hz, 1H), 5.03 (d, *J* = 2.9 Hz, 2H), 4.15–3.86 (m, 2H), 3.14 (s, 1H), 1.35 (dd, *J* = 6.3, 1.3 Hz, 3H), 1.19 (s, 3H), 0.99–0.85 (m, 6H). ^13^C{^1^H} (75 MHz, C_6_D_6_): 162.5 (C), 152.4 (C), 142.8 (C), 138.7 (C), 131.0 (CH), 129.0 (CH), 127.2 (CH), 126.0 (CH), 126.0 (C), 125.5 (CH), 122.3 (CH), 115.3 (CH), 103.6 (C), 75.9 (CH_2_), 61.2 (CH_2_), 61.2 (CH), 43.4 (C), 29.5 (CH_3_), 24.4 (CH_3_), 16.8 (CH_3_), 14.0 (CH_3_). 4 × CH_sp^2^_ are overlapped with C_6_D_6_. ESI(+)-MS *m*/*z* (%): 421 (100) [M + H]^+^. Anal. Calcd for C_25_H_28_N_2_O_4_: C, 71.41; H, 6.71; N, 6.66. Found: C, 71.68; H, 6.72; N, 6.67.

#### Ethyl 3-(Benzyloxy)-1,1-dimethyl-2-oxo-1,2,3,4-tetrahydro-6*H*-4,10b-epoxyazepino[4,5-*b*]indole-6-carboxylate (**10**)

The general procedure was followed using ethyl 4*H*-furo[3,2-*b*]indole-4-carboxylate **5a** (46.0 mg, 0.2 mmol) and *N*-(benzyloxy)-2-bromo-2-methylpropanamide **7** (82.0 mg, 0.3 mmol). The purification of the crude by flash chromatography (SiO_2_, hexane/ethyl acetate 8:2) yielded **10** (53 mg, 63%) as a transparent oil. ^1^H NMR (300 MHz, C_6_D_6_): 8.27 (br s, 1H), 7.49 (m, 2H), 7.37–7.08 (m, 5H), 6.86 (td, *J* = 7.6, 0.8 Hz, 1H), 6.01 (br s, 1H), 5.54 (d, *J* = 1.8 Hz, 1H), 5.21 (d, *J* = 10.6 Hz, 1H), 4.97 (d, *J* = 10.6 Hz, 1H), 3.97 (m, 2H), 1.69 (s, 3H), 1.02 (s, 3H), 0.88 (t, *J* = 7.1 Hz, 3H). ^13^C{^1^H} (75 MHz, C_6_D_6_): 174.2 (C), 153.7 (C), 150.8 (C), 146.8 (C), 136.2 (C), 130.6 (CH), 129.6 (2 × CH), 128.5 (CH), 128.4 (2 × CH), 125.6 (CH), 125.3 (C), 123.6 (CH), 115.7 (CH), 110.5 (CH), 96.3 (CH), 94.7 (C), 77.8 (CH_2_), 62.5 (CH_2_), 52.4 (CH_2_), 22.9 (CH_3_), 18.7 (CH_3_), 13.8 (CH_3_). ESI(+)-MS *m*/*z* (%): 421 (100) [M + H]^+^. Anal. Calcd for C_24_H_24_N_2_O_5_: C, 68.56; H, 5.75; N, 6.66. Found: C, 68.38; H, 5.77; N, 6.68.

### Reaction between 3-Vinylindole **11** and **2a** or **7**

#### Ethyl 12-Oxo-10-(*p*-tolyl)-5a,6,7,8,9,10-hexahydro-5*H*-6,9-methanocycloocta[*b*]indole-5-carboxylate (**12**)

The general procedure employed for the synthesis of **3a–m** was followed using ethyl (*E*)-3-(4-methylstyryl)-1*H*-indole-1-carboxylate **11** (61.1 mg, 0.2 mmol) and 2-bromocyclopentan-1-one **2a** (46.0 mg, 0.28 mmol) for 24 h at rt. The purification of the crude by column chromatography (SiO_2_, hexane/ethyl acetate 9:1) yielded **12** (52 mg, 67%) as a white solid (mp 85–90 °C). ^1^H NMR (300 MHz, C_6_D_6_): 8.35 (br s, 1H), 7.25–7.16 (m, 2H), 7.15–7.10 (m, 4H), 6.92 (t, *J* = 7.2 Hz, 1H), 6.47 (t, *J* = 3.5, 1H), 4.73 (s, 1H), 4.20 (m, 2H), 3.75–3.64 (m, 2H), 2.65 (m, 1H), 2.25 (s, 3H), 1.70 (m, 1H), 1.51–1.33 (m, 3H), 1.18 (t, *J* = 7.1 Hz, 3H). ^13^C{^1^H} (75 MHz, C_6_D_6_): 217.0 (C), 152.8 (C), 144.9 (C), 142.4 (C), 138.1 (C), 136.2 (C), 129.6 (2 × CH), 129.5 (CH), 129.3 (C), 127.6 (2 × CH), 123.0 (CH), 121.2 (CH), 119.4 (CH), 116.1 (CH), 66.0 (CH), 61.8 (CH_2_), 56.9 (CH), 50.3 (CH), 48.2 (CH), 21.3 (CH_2_), 20.7 (CH), 20.2 (CH_2_), 14.2 (CH_3_). ESI(+)-MS *m*/*z* (%): 388 (100) [M + H]^+^. Anal. Calcd for C_25_H_25_NO_3_: C, 77.49; H, 6.50; N, 3.61. Found: C, 77.24; H, 6.52; N, 3.60.

#### Ethyl-3-(benzyloxy)-5,5-dimethyl-4-oxo-2-(*p*-tolyl)-3,4,5,5a-tetrahydroazepino[4,5-*b*]indole-6(2*H*)-carboxylate (**13**)

The general procedure employed for the synthesis of **8–10** was followed using ethyl (*E*)-3-(4-methylstyryl)-1*H*-indole-1-carboxylate **11** (61.1 mg, 0.2 mmol) and *N*-(benzyloxy)-2-bromo-2-methylpropanamide **7** (82.0 mg, 0.3 mmol) for 24 h. The purification of the crude by flash chromatography (SiO_2_, hexane/ethyl acetate 9:1) yielded **13** (48 mg, 48%) as a clear thick oil. ^1^H NMR (500 MHz, C_6_D_6_): 7.94 (br s, 1H), 7.37–7.30 (m, 2H), 7.15 (ddt, *J* = 10.1, 8.4, 1.8 Hz, 4H), 7.11–7.03 (m, 2H), 6.97 (d, *J* = 7.8 Hz, 2H), 6.83 (dd, *J* = 4.1, 3.6 Hz, 1H), 6.73 (td, *J* = 7.5, 1.0 Hz, 1H), 5.75 (m, 1H), 5.63 (br s, 1H), 5.27 (t, *J* = 3.1 Hz, 1H), 4.64 (s, 2H), 4.03 (m, 2H), 2.06 (s, 3H), 1.65 (s, 3H), 1.11 (s, 3H), 1.00 (t, *J* = 7.1 Hz, 3H). ^13^C{^1^H} (126 MHz, C_6_D_6_): 181.7 (C), 154.2 (C), 145.6 (C), 137.8 (C), 137.7 (C), 137.2 (C), 136.9 (C), 129.6 (CH), 129.4 (2 × CH), 129.1 (2 × CH), 128.8 (C), 128.2 (2 × CH), 128.0 (2 × CH), 127.7 (CH), 123.3 (CH), 119.8 (CH), 117.4 (CH), 114.6 (CH), 74.9 (CH_2_), 69.2 (CH), 64.9 (CH), 61.8 (CH_2_), 54.3 (C), 26.6 (CH_3_), 20.6 (CH_3_), 19.0 (CH_3_), 14.0 (CH_3_). ESI(+)-MS *m*/*z* (%): 497 (100) [M + H]^+^. Anal. Calcd for C_31_H_32_N_2_O_4_: C, 74.98; H, 6.50; N, 5.64; found: C, 75.14; H, 6.51; N, 5.63.

### Preparation and Characterization Data for Compounds **14–17**

#### Ethyl 12-Oxo-7-(*p*-tolyl)-6,7,8,9,10,11-hexahydro-5*H*-8,11-methanocycloocta[*b*]indole-5-carboxylate (**14**)

To a stirring solution of **3d** (38.7 mg, 0.1 mmol) in CHCl_3_ (0.5 mL, 0.2 M), *p*-TSOH (1.90 mg, 0.01 mmol) was added, and the mixture was stirred for 2 h at room temperature. The reaction mixture was then quenched with NaHCO_3_-saturated solution (5 mL) and extracted with ethyl acetate (3 × 5 mL). The combined organic layers were dried over Na_2_SO_4_, filtered, and concentrated to yield **14** (37 mg, 96%) as a brownish solid (mp 177–180 °C). ^1^H NMR (300 MHz, CDCl_3_): 8.00 (m, 1H), 7.53 (m, 1H), 7.30–7.23 (m, 4H), 7.16 (m, 2H), 4.45 (q, *J* = 7.14, 2H), 4.11 (m, 1H), 3.69–3.51 (m, 3H), 2.85 (m, 1H), 2.50 (m, 1H), 2.35 (m, 4H), 2.23–2.11 (m, 2H), 1.45 (t, *J* = 7.1 Hz, 3H). ^13^C{^1^H} (75 MHz, CDCl_3_): 216.8 (C), 152.2 (C), 141.5 (C), 136.4 (C), 135.4 (C), 135.4 (C), 129.3 (2 × CH), 128.1 (C), 126.8 (2 × CH), 124.4 (CH), 123.0 (CH), 119.8 (C), 117.8 (CH), 115.4 (CH), 63.3 (CH_2_), 53.7 (CH), 49.0 (CH), 43.5 (CH), 28.4 (CH_2_), 28.1 (CH_2_), 21.0 (CH_3_), 20.2 (CH_2_), 14.3 (CH_3_). ESI(+)-MS *m*/*z* (%): 388 (100) [M + H]^+^. Anal. Calcd. for C_25_H_25_NO_3_: C, 77.49; H, 6.50; N, 3.61. Found: C, 77.74; H, 6.52; N, 3.61.

#### 7-(*p*-Tolyl)-6,7,8,9,10,11-hexahydro-5*H*-8,11-methanocycloocta[*b*]indol-12-one (**15**)

To a stirring solution of **3d** (50.0 mg, 0.13 mmol) in MeOH (1.4 mL, 0.01 M), K_2_CO_3_ (17.8 mg, 0.13 mmol) was added, and the mixture was stirred for 5 h at 50 °C. The solvent was then removed and the crude was diluted with water (5 mL) and extracted with ethyl acetate (3 × 5 mL). The combined organic layers were dried over Na_2_SO_4_, filtered, and concentrated. The crude was purified by column chromatography (SiO_2_, hexane/ethyl acetate 9:1) to yield **15** (32 mg, 78%) as a white solid (mp 186–190 °C). ^1^H NMR (300 MHz, acetone-*d*_6_): 10.04 (br s, 1H), 7.54 (m, 1H), 7.33–7.26 (m, 3H), 7.18 (m, 2H), 7.09–7.00 (m, 2H), 3.73–3.57 (m, 3H), 3.10 (m, 1H), 2.84 (s, 1H), 2.71 (m, 1H), 2.50–2.36 (m, 2H), 2.32 (s, 3H), 2.21 (m, 1H). ^13^C{^1^H} (75 MHz, acetone-*d*_6_): 215.7 (C), 142.0 (C), 136.0 (C), 134.8 (C), 133.5 (C), 129.2 (2 × CH), 127.3 (C), 126.7 (2 × CH), 121.1 (CH), 119.0 (CH), 117.3 (CH), 110.5 (CH), 110.5 (C), 52.8 (CH), 48.4 (CH), 44.0 (CH), 30.1 (CH_2_), 29.0 (CH_2_), 20.1 (CH_3_) 19.5 (CH_2_). ESI(+)-MS *m*/*z* (%): 316 (100) [M + H]^+^. Anal. Calcd for C_22_H_21_NO: C, 83.78; H, 6.71; N, 4.44. Found: C, 83.53; H, 6.70; N, 4.46.

#### Ethyl 12-Hydroxy-7-(*p*-tolyl)-7,8,9,10,11,11a-hexahydro-5*H*-8,11-methanocycloocta[*b*]indole-5-carboxylate (**16**)

To a stirring solution of **3d** (50.0 mg, 0.13 mmol) in EtOH (1.3 mL, 0.1 M), NaBH_4_ (4.90 mg, 0.13 mmol) was added, and the mixture was stirred for 2 h at room temperature. The reaction mixture was then quenched with NH_4_Cl saturated solution (5 mL) and extracted with ethyl acetate (3 × 5 mL). The combined organic layers were dried over Na_2_SO_4_, filtered, and concentrated. The crude was purified by column chromatography (SiO_2_, hexane/ethyl acetate 9:1) to yield **16** (33 mg 65%) as a white solid (mp 135–139 °C). ^1^H NMR (300 MHz, C_6_D_6_): 8.21 (d, *J* = 8.2 Hz, 1H), 7.42 (d, *J* = 8.0 Hz, 2H), 7.26 (m, 2H overlapped with C_6_D_6_), 7.16–7.04 (m, 4H), 4.73 (m, 1H), 4.53 (m 1H), 4.17–4.09 (m, 2H), 4.0 (t, *J* = 6.9 Hz, 1H) 2.50 (br s, 1H), 2.27 (m, 4H), 1.99 (m, 1H), 1.55–1.45 (m, 3H), 1.35 (br s, 1H), 1.04 (t, *J* = 7.1 Hz, 3H). ^13^C{^1^H} (75 MHz, C_6_D_6_): 152.9 (C), 145.8 (C), 143.6 (C), 142.5 (C), 135.0 (C), 132.8 (C), 129.3 (2 × CH), 128.9 (CH), 127.9 (2 × CH), 123.4 (CH), 122.9 (CH), 116.3 (CH), 115.4 (CH), 75.6 (CH), 61.7 (CH_2_), 49.5 (CH), 44.0 (CH), 43.2 (CH), 40.3 (CH), 23.5 (CH_2_), 23.4 (CH_2_), 20.8 (CH_3_), 14.0 (CH_3_). ESI(+)-MS *m*/*z* (%): 390 (100) [M + H]^+^. Anal. Calcd for C_25_H_27_NO_3_: C, 77.09; H, 6.99; N, 3.60. Found: C, 76.91; H, 7.01; N, 3.61.

#### Ethyl 2-(2-Oxocyclopentyl)-4*H*-furo[3,2-*b*]indole-4-carboxylate (**17**)

To a stirring solution of **6a** (47.0 mg, 0.15 mmol) in CHCl_3_ (0.75 mL, 0.2 M), *p*TSOH (3.00 mg, 0.015 mmol) was added, and the mixture was stirred for 1.5 h at room temperature. The reaction mixture was concentrated, and the crude was purified by column chromatography (SiO_2_, hexane/ethyl acetate 8:2) to yield **17** (44 mg 94%) as a pink solid (117–119 °C). ^1^H NMR (500 MHz, CDCl_3_): 8.33 (br s, 1H), 7.63 (m, 1H), 7.34–7.25 (m, 2H), 6.68 (s, 1H), 4.53 (q, *J* = 7.1 Hz, 2H), 3.59 (dd, *J* = 10.6, 8.5 Hz, 1H), 2.57 (m, 1H), 2.50 (ddd, *J* = 18.8, 8.5, 3.5 Hz, 1H), 2.44 (dd, *J* = 10.1, 8.5 Hz, 1H), 2.36 (m, 1H), 2.25 (m, 1H), 2.00 (m, 1H), 1.52 (t, *J* = 7.1 Hz, 3H). ^13^C{^1^H} (151 MHz, CDCl_3_): 214.8 (C), 155.3 (C), 151.0 (C), 142.7 (C), 138.2 (C), 129.7 (C), 123.6 (CH), 123.3 (CH), 118.1 (C), 116.3 (CH), 116.1 (CH), 100.8 (CH), 63.0 (CH_2_), 49.8 (CH), 37.9 (CH_2_), 29.6 (CH_2_), 21.0 (CH_2_), 14.5 (CH_3_).

ESI(+)-MS *m*/*z* (%): 312 (100) [M + H]^+^. Anal. Calcd for C_18_H_17_NO_4_: C, 69.44; H, 5.50; N, 4.50. Found: C, 69.21; H, 5.52; N, 4.48.

## Supporting Information Available

Crystallographic data for **6a** (CCDC no. 1964975) (CIF)
NMR spectra of all synthesized compounds, 2D-NMR spectra of compound products **3a**, **3d**, **3i**, **3j**, **3l**, **6a**, **6f**, **6h**, **8**, **9**, **10**, **12**, **16**, and **17** (PDF)

## Supplementary Material

jo9b03117_si_001.cif
