# Supplementary material for: Synthesis of Cyclohepta[b]indoles by (4 + 3) Cycloaddition of 2-Vinylindoles or 4H-Furo[3,2-b]indoles with Oxyallyl Cations
Source: J Org Chem. 2020 Jan 24;85(5):3265–76. doi: 10.1021/acs.joc.9b03117 (PMC7997566; doi:10.1021/acs.joc.9b03117)

Supporting Information

Synthesis of cyclohepta[*b*]indoles by (4+3) cycloaddition of 2-vinylindoles or 4*H*-furo [3,2-*b*]indoles with oxyallyl cations

Valentina Pirovano,[†*] Elisa Brambilla,[†] Andrea Moretti,[†] Silvia Rizzato,[ʃ] Giorgio Abbiati,[†] Donatella Nava[†] and Elisabetta Rossi*[†]

[†] Dipartimento di Scienze Farmaceutiche - Sezione di Chimica Generale e Organica "A. Marchesini", Università degli Studi di Milano, Via Venezian 21, 20133 Milano, Italy

[ʃ] Dipartimento di Chimica, Università degli Studi di Milano, Via Golgi 19, 20133, Milano, Italy

e-mail: valentina.pirovano@unimi.it or elisabetta.rossi@unimi.it

Table of Contents

COSY-, HSQC-, NOESY- and HMBC-NMR of products **3a, 3d, 3i 3j, 3l, 6a, 6f, 6h, 8, 9, 10, 12, 16** and **17**	S-2
Crystallographic data for **6a**	S-115
^{1}H- and ^{13}C-NMR spectra of new compounds	S-118

COSY-, HSQC- and NOESY-NMR of products 3a, 3i 3j, 3l, 6a, 6f, 6h, 8, 10, 12, 16 and 17

3a, COSY in C_6D_6 at T = 300 K

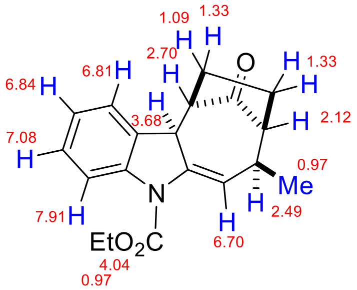

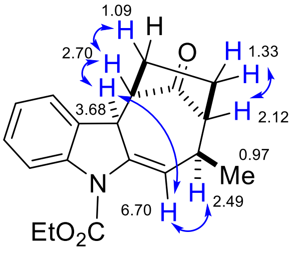

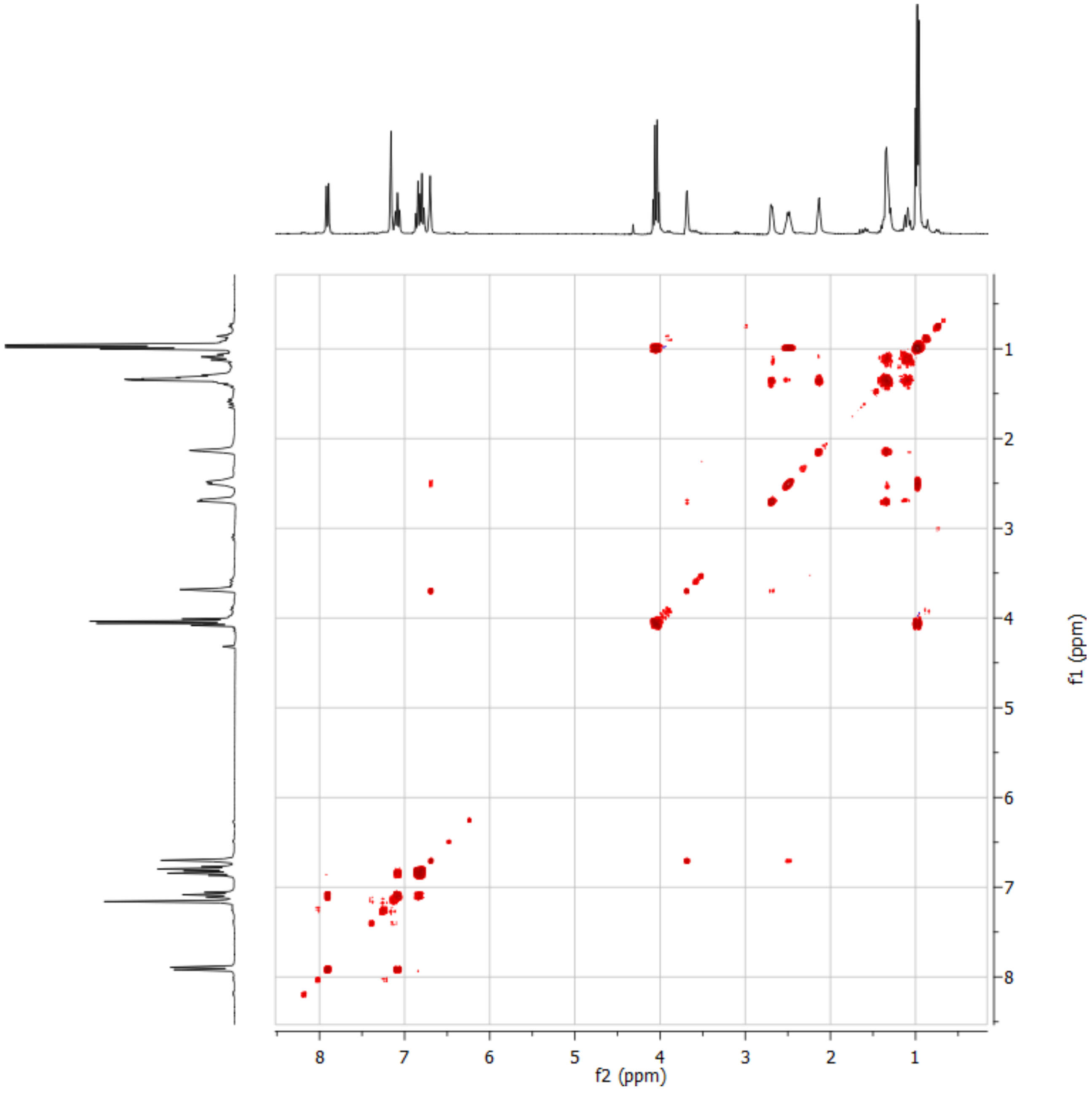

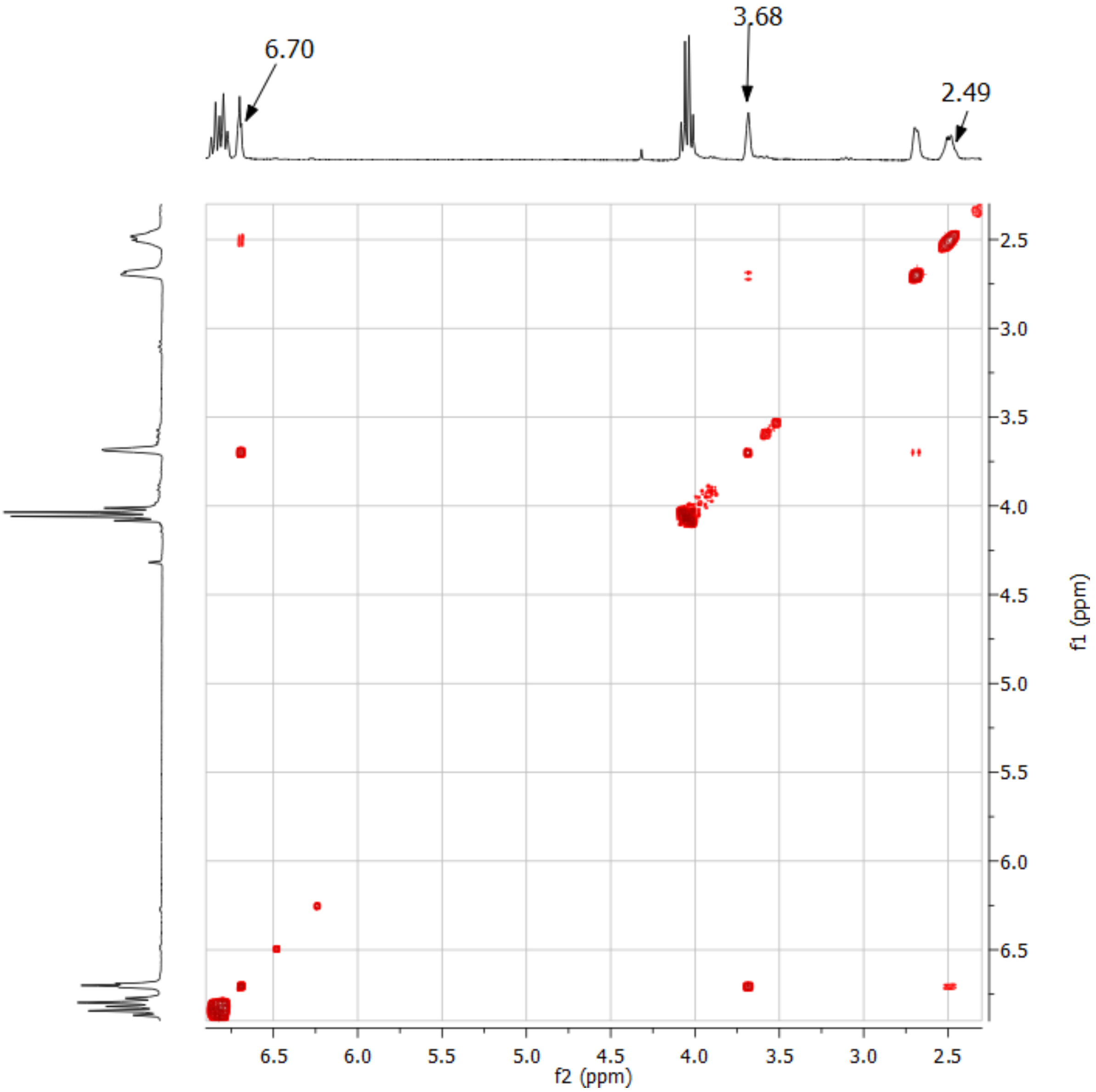
6.70
3.68
2.49
f2 (ppm)
f1 (ppm)
6.5
6.0
5.5
5.0
4.5
4.0
3.5
3.0
2.5

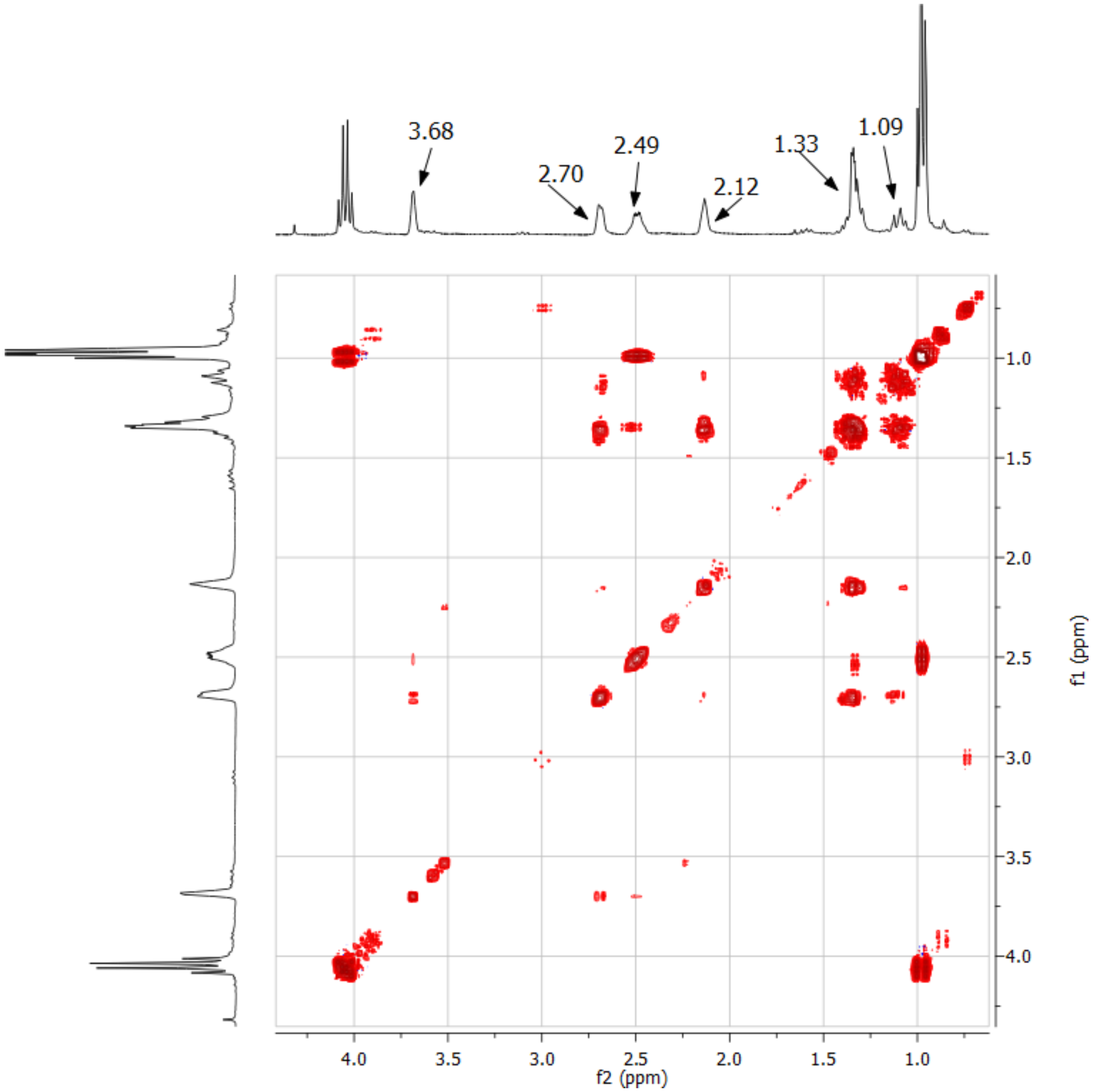
3.68
2.70
2.49
2.12
1.33
1.09
4.0
3.5
3.0
2.5
2.0
1.5
1.0
f2 (ppm)
f1 (ppm)

3a, HSQC in C_6D_6 at T = 300 K

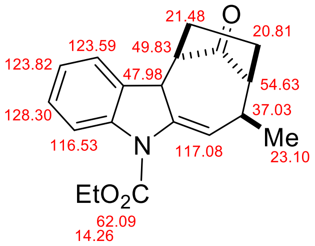

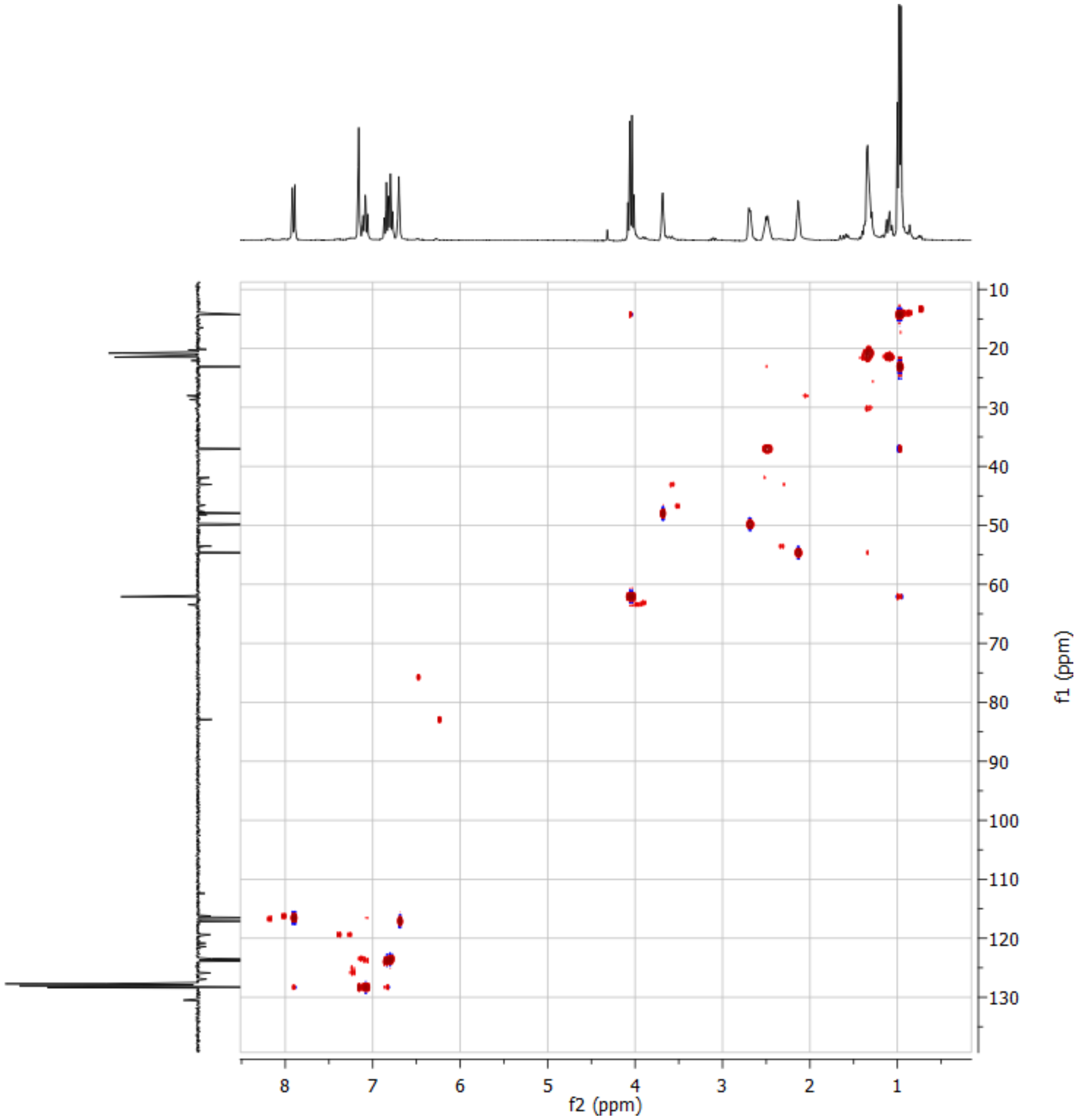

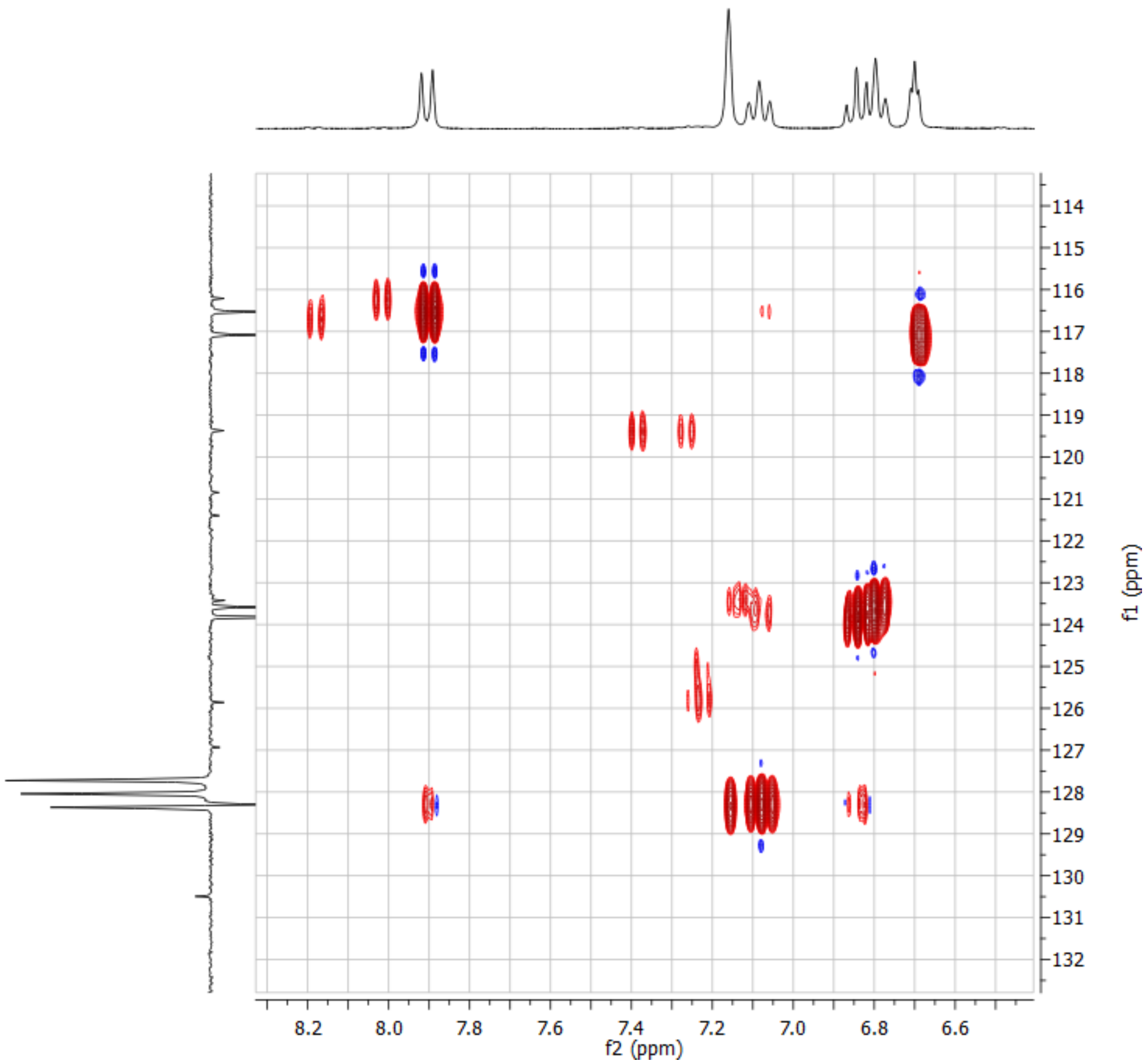
114
115
116
117
118
119
120
121
122
123
124
125
126
127
128
129
130
131
132
f1 (ppm)
8.2
8.0
7.8
7.6
7.4
7.2
7.0
6.8
6.6
f2 (ppm)

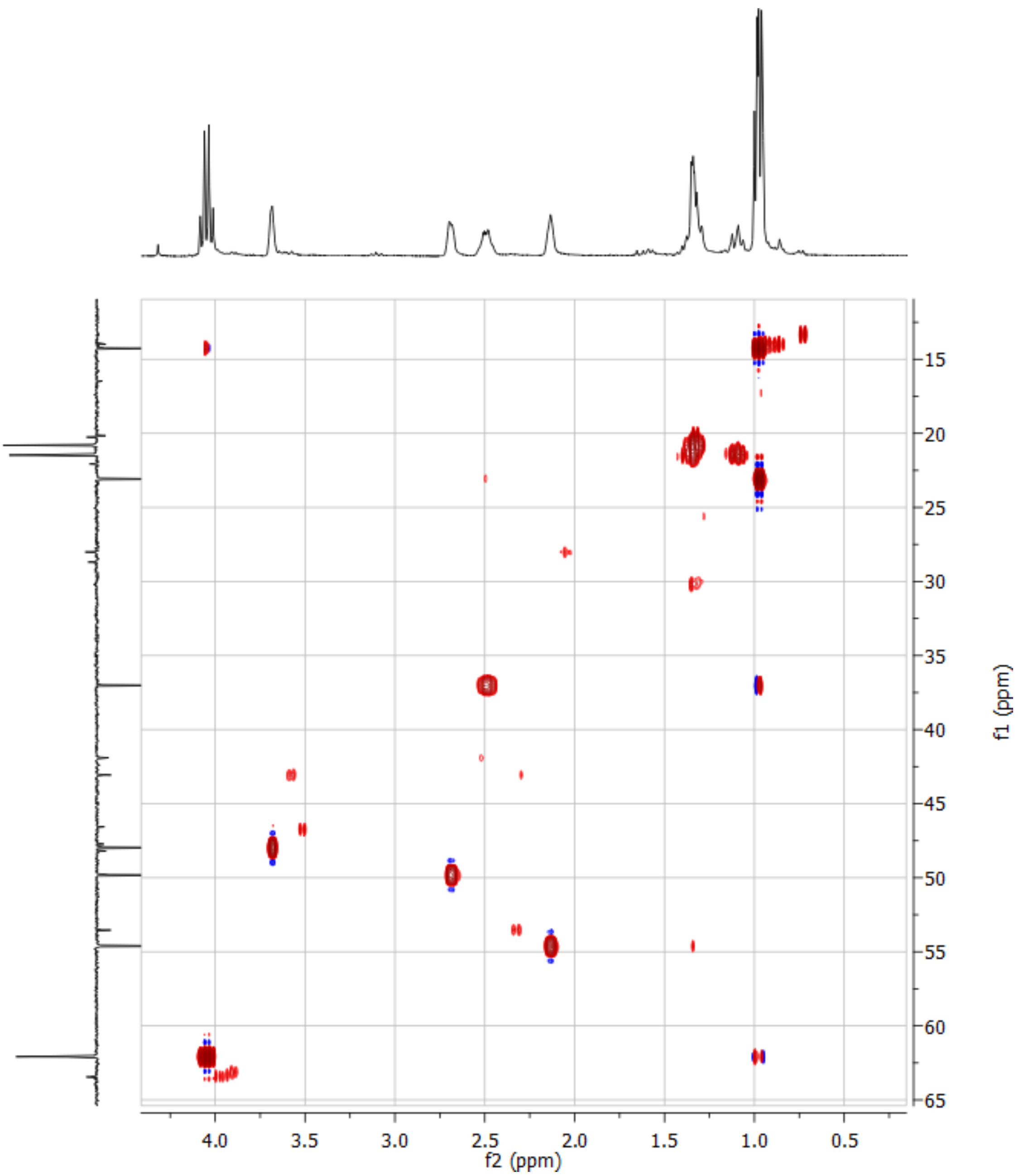

4.0
3.5
3.0
2.5
2.0
1.5
1.0
0.5
f2 (ppm)
15
20
25
30
35
40
45
50
55
60
65
f1 (ppm)

3a, NOESY in C_6D_6 at T = 300 K

For a better understanding of the diagnostic NOESY interactions, beside a picture reporting diagnostic cross picks with the use of arrows, we report several views of a MM2 minimized 3D model of **3a**.

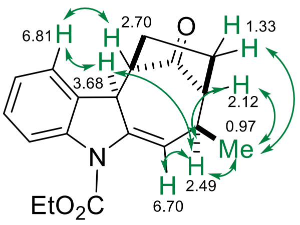

diagnostic cross picks

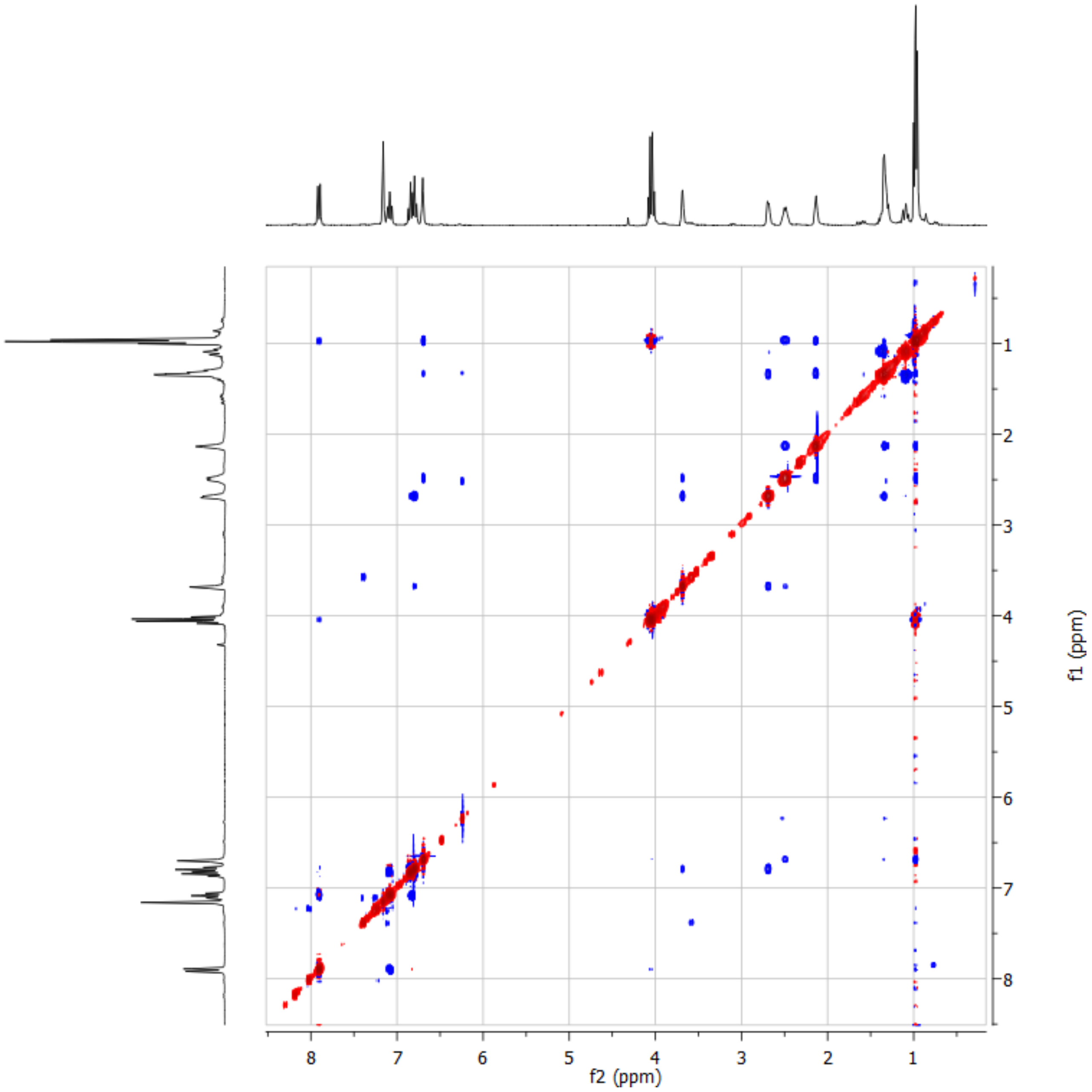

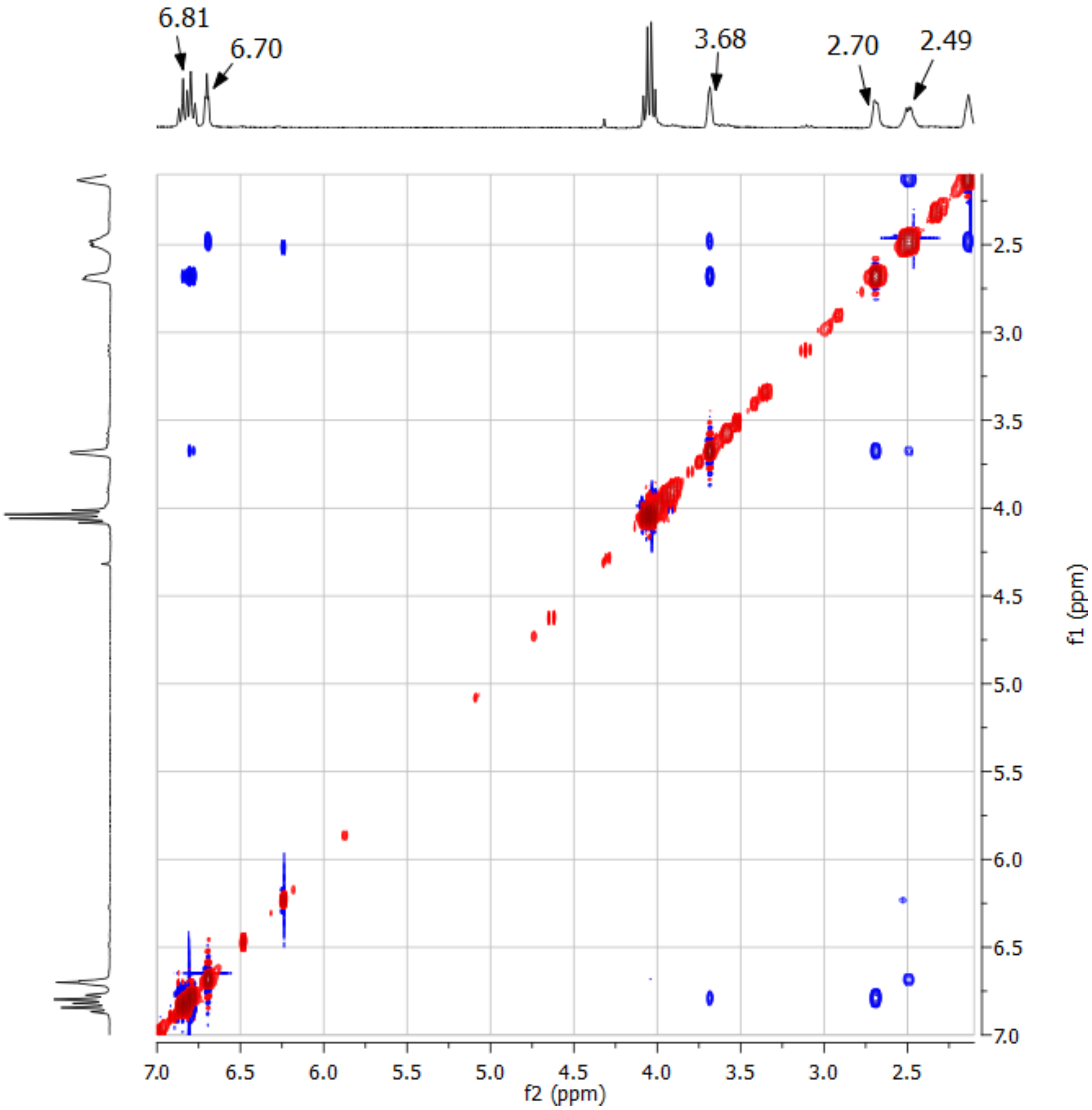
6.81
6.70
3.68
2.70
2.49
f2 (ppm)
f1 (ppm)

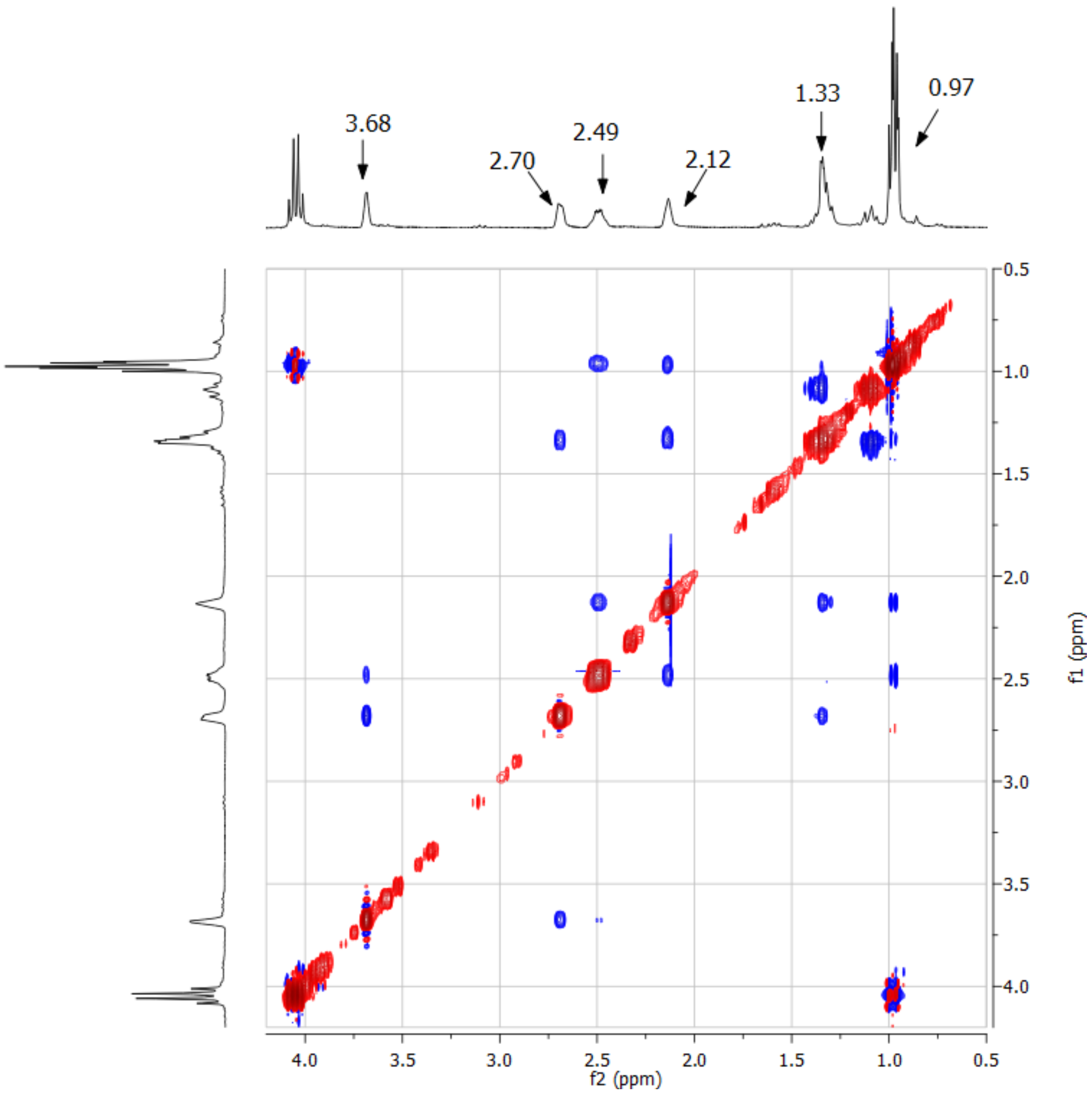

3.68
2.70
2.49
2.12
1.33
0.97
4.0
3.5
3.0
2.5
2.0
1.5
1.0
0.5
f2 (ppm)
f1 (ppm)

3a, MM2 minimized 3D structure

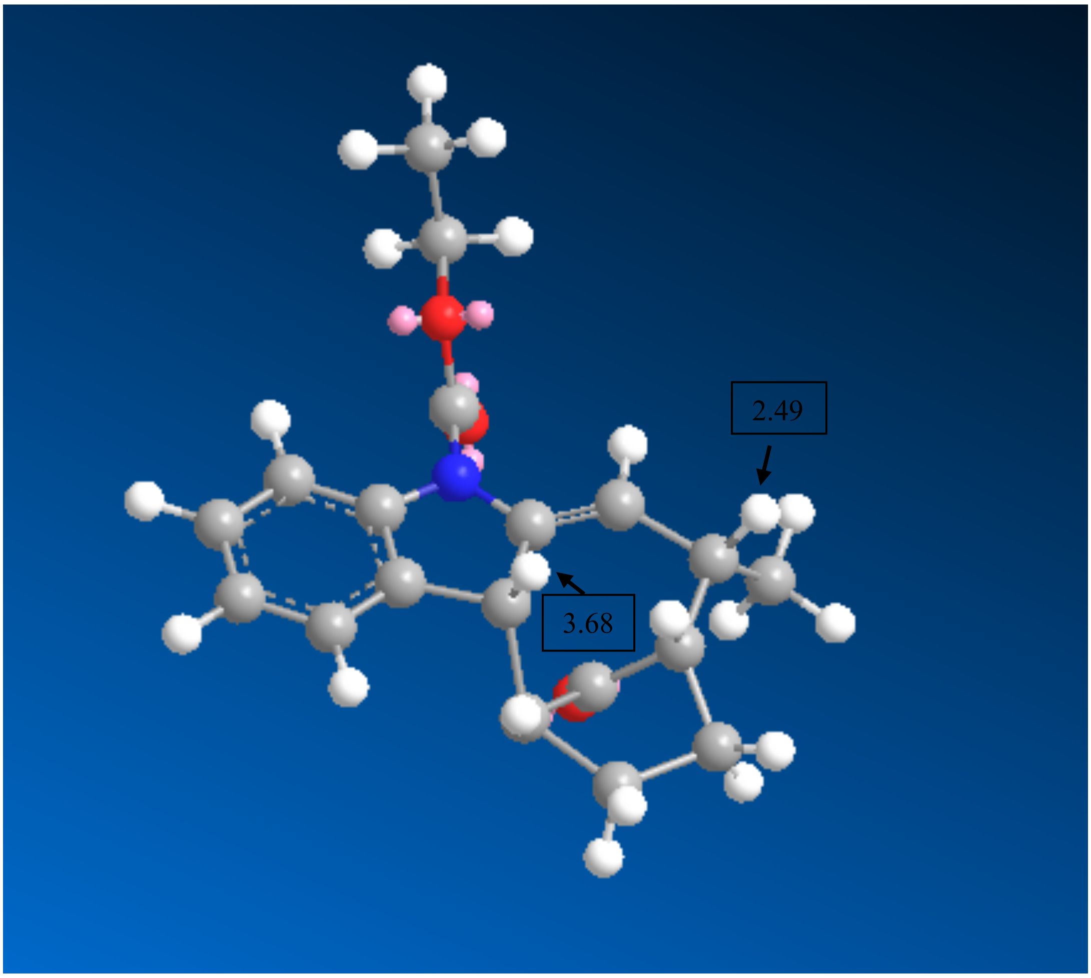

Figure S1: MM2 minimized 3D structure of **3a**.

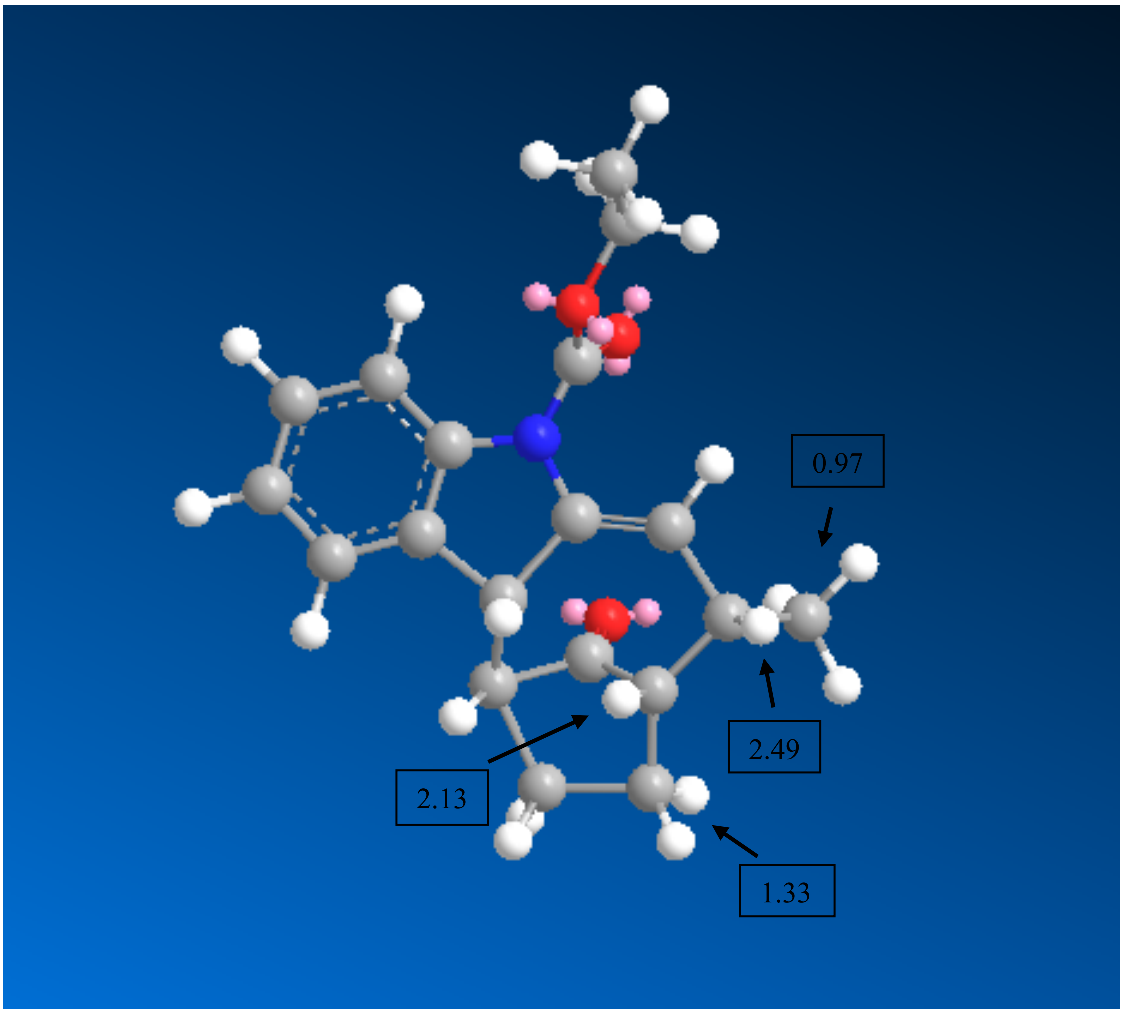

Figure S2: MM2 minimized 3D structure of **3a**.

3d, COSY in C_6D_6 at T = 300 K

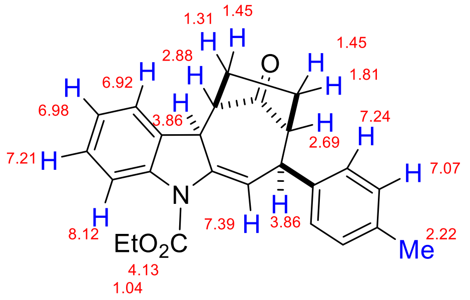

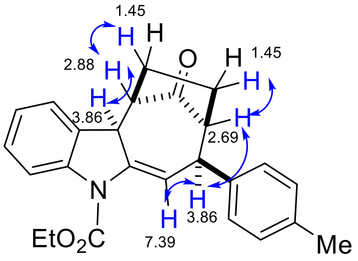

diagnostic cross picks

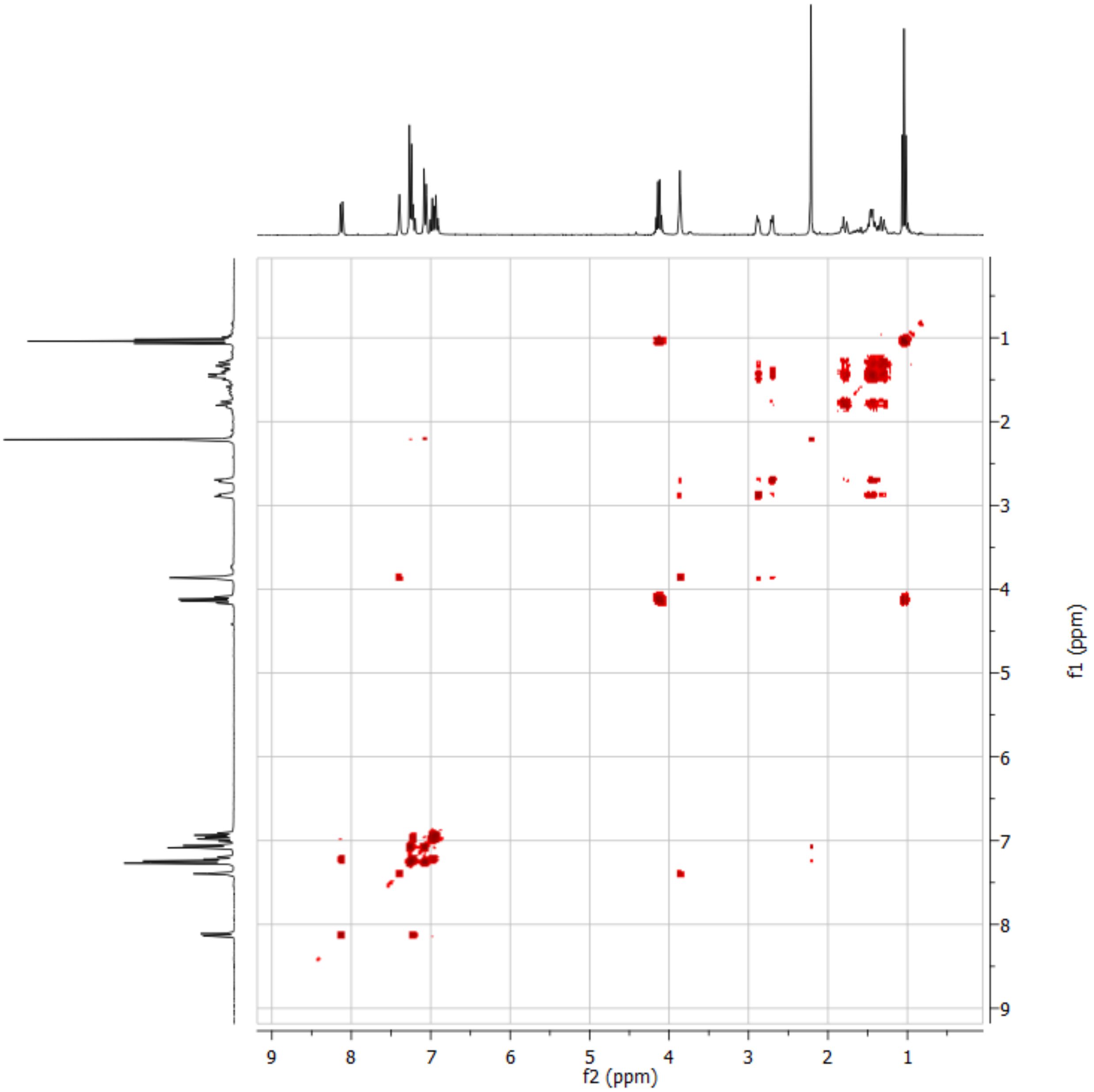

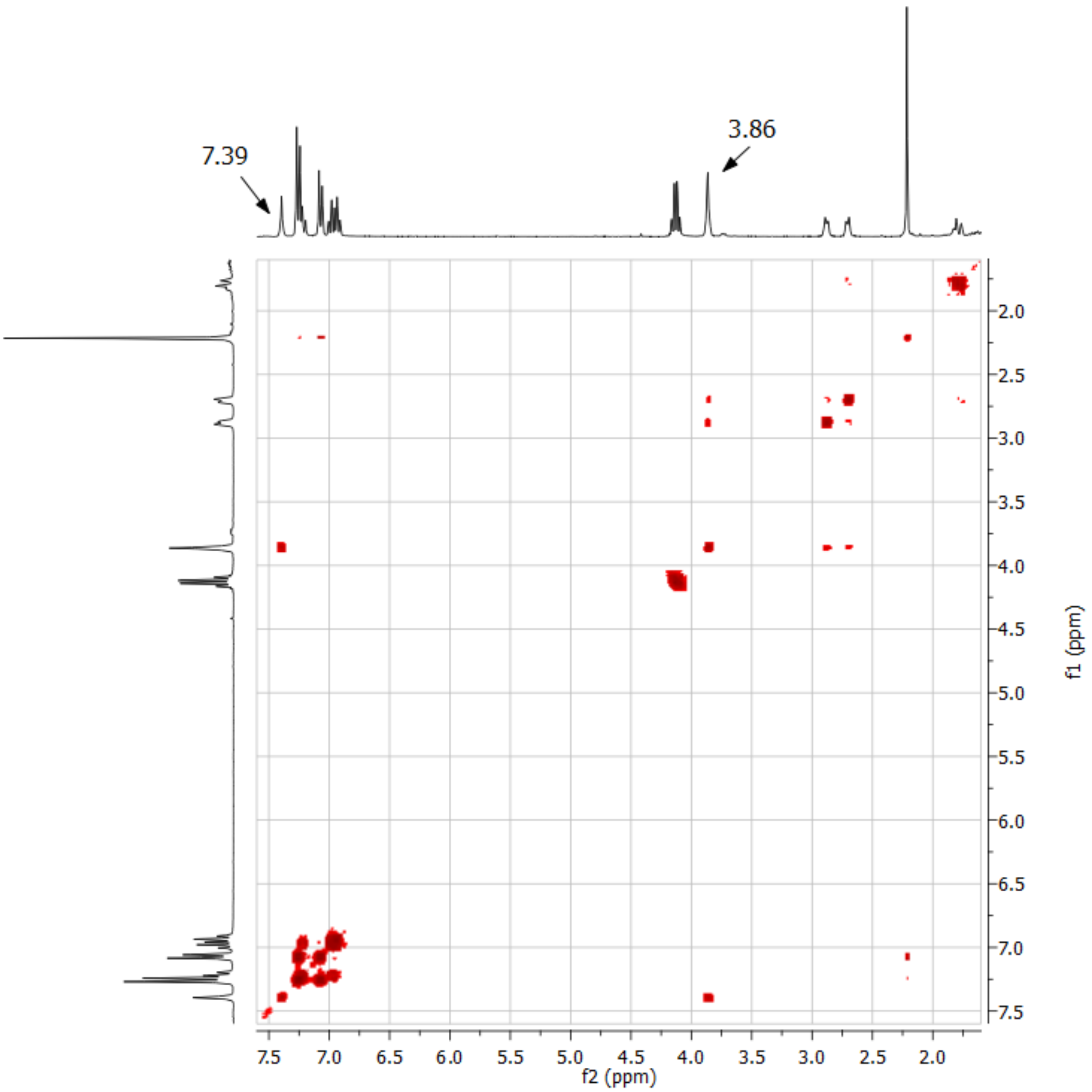

7.39
3.86
f1 (ppm)
f2 (ppm)

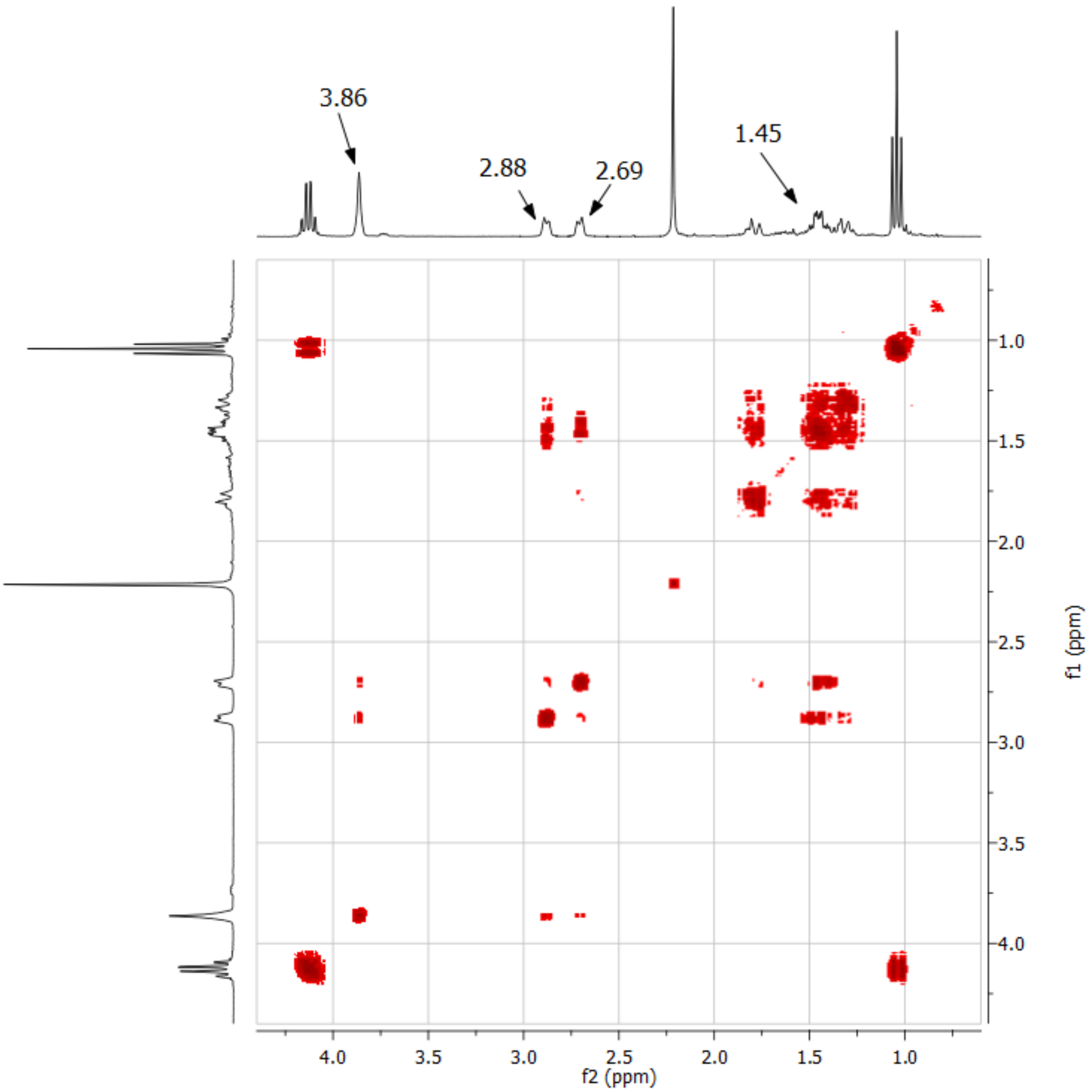
3.86
2.88
2.69
1.45
f2 (ppm)
f1 (ppm)

3d, HSQC in C_6D_6 at T = 300 K

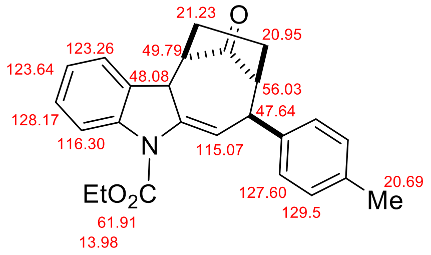

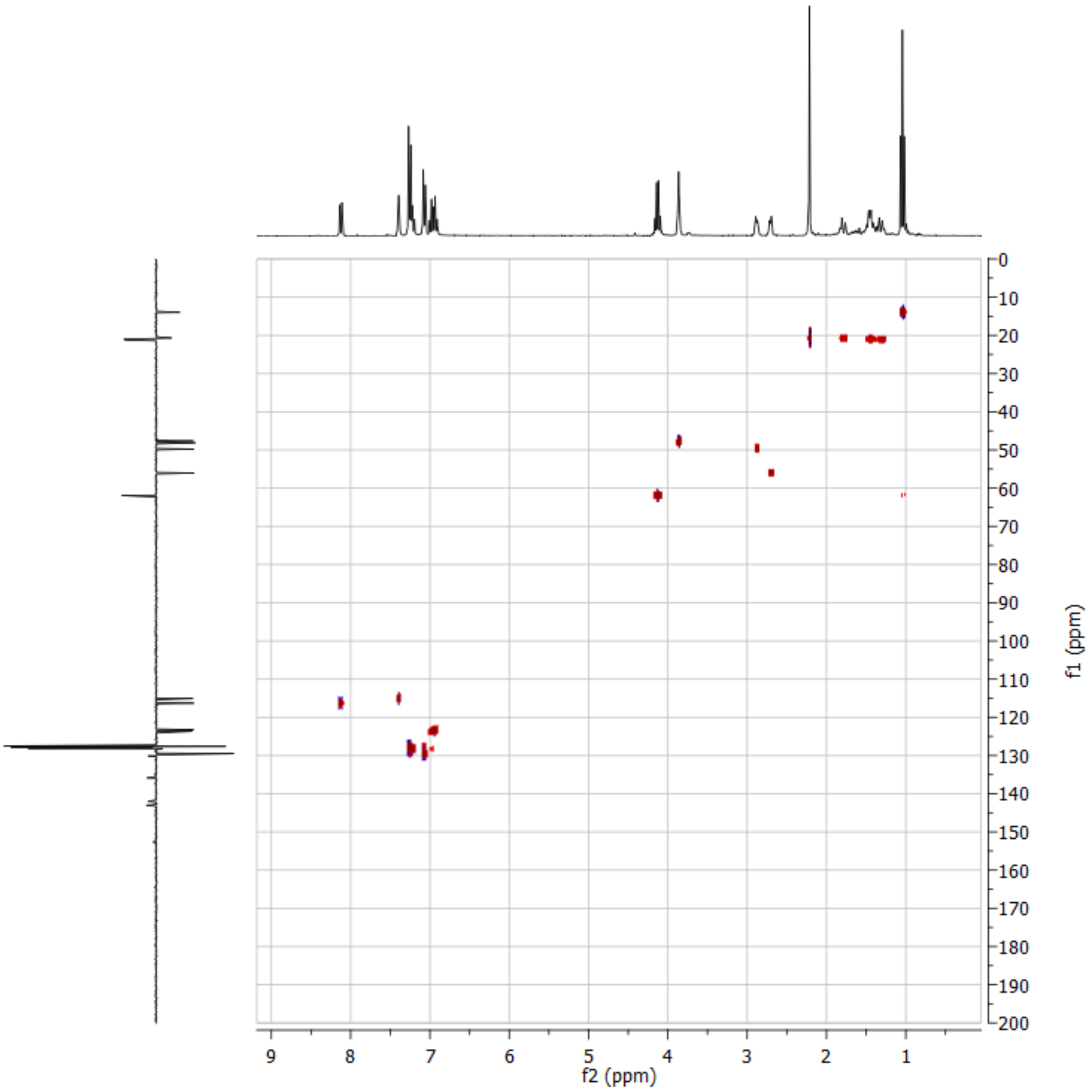

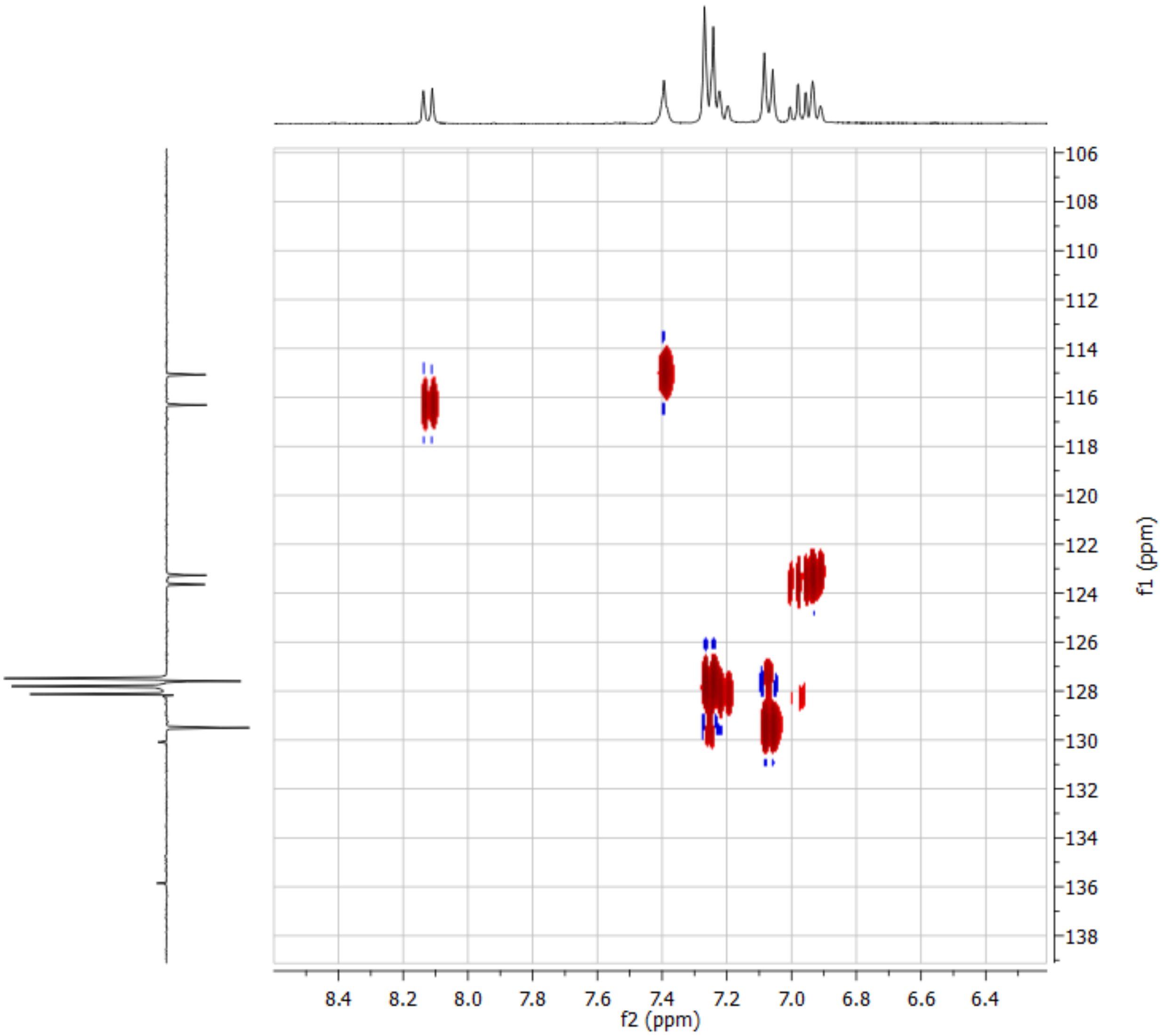
f2 (ppm)
f1 (ppm)

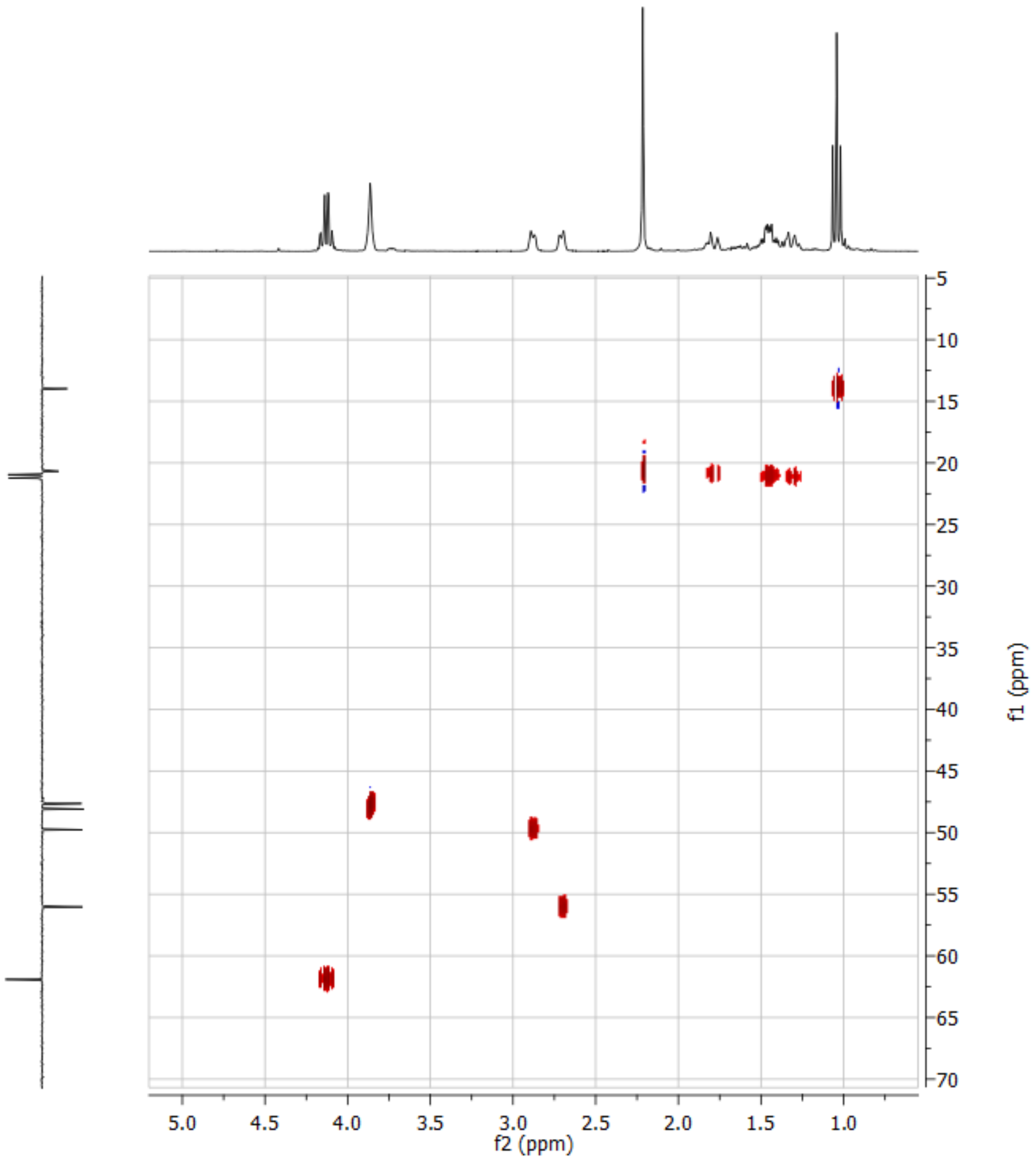
f1 (ppm)
f2 (ppm)
5.0
4.5
4.0
3.5
3.0
2.5
2.0
1.5
1.0
5
10
15
20
25
30
35
40
45
50
55
60
65
70

3d, NOESY in C_6D_6 at T = 300 K

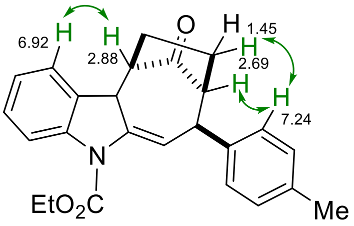

diagnostic cross picks

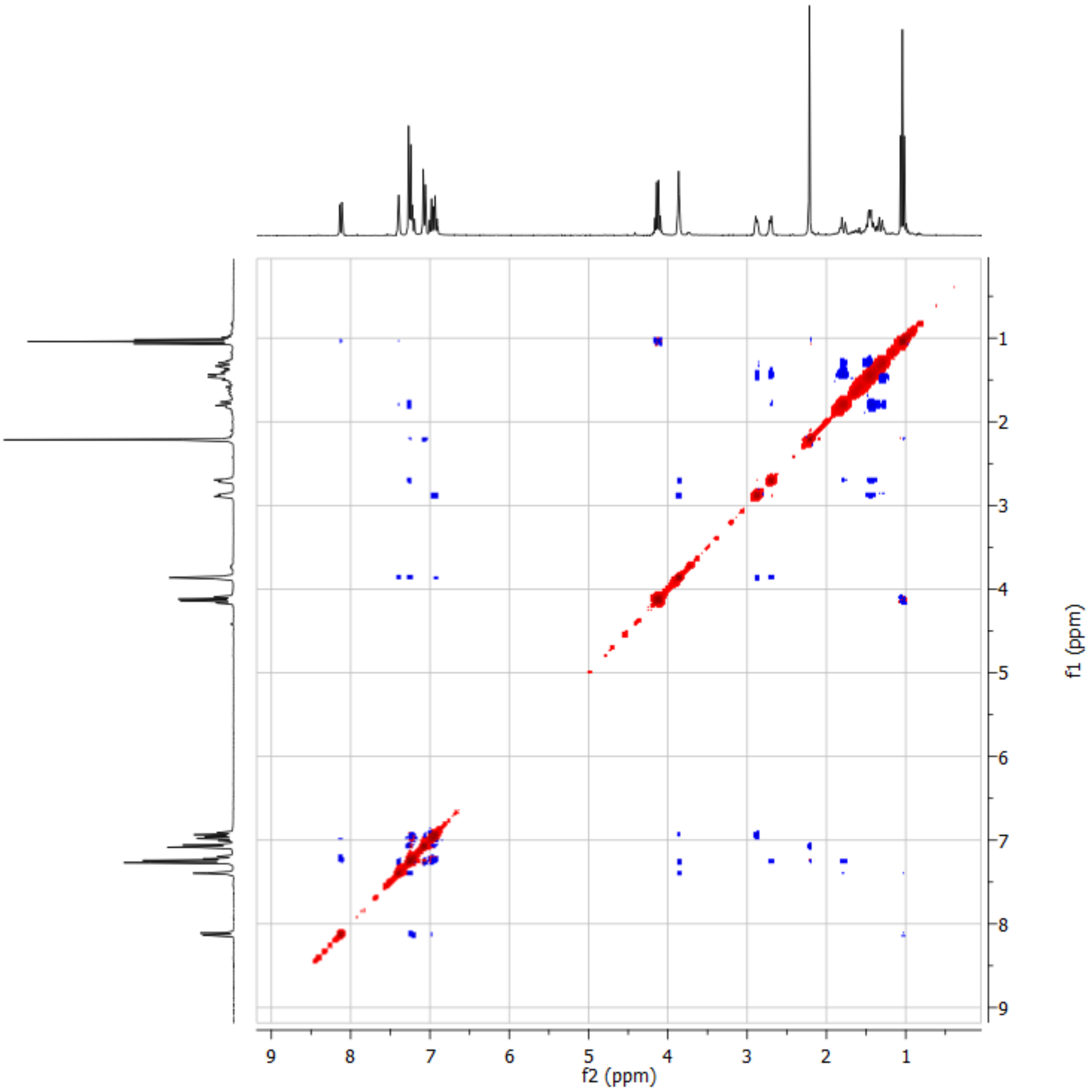

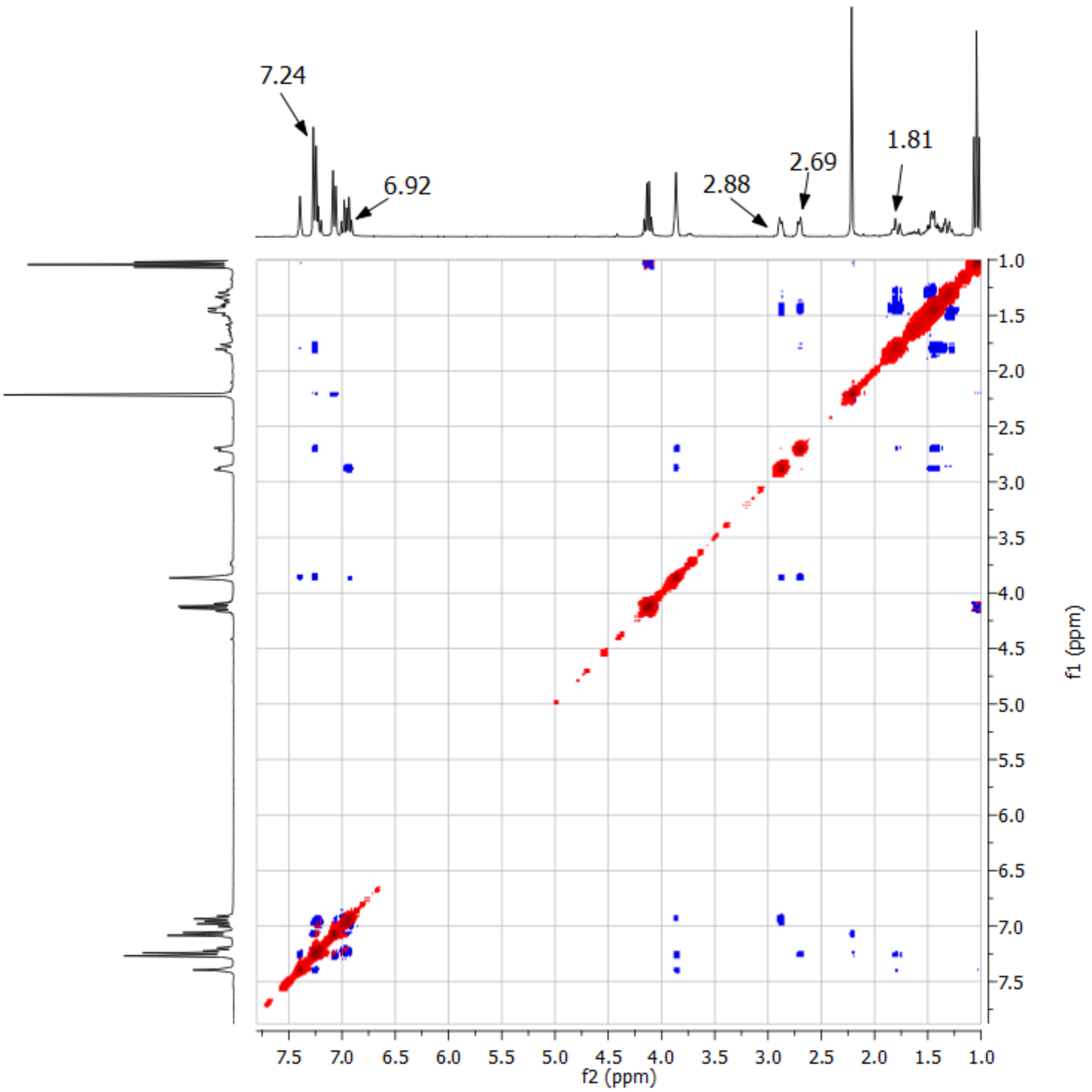
7.24
6.92
2.88
2.69
1.81
f2 (ppm)
f1 (ppm)

3d, HMBC in C_6D_6 at T = 300 K

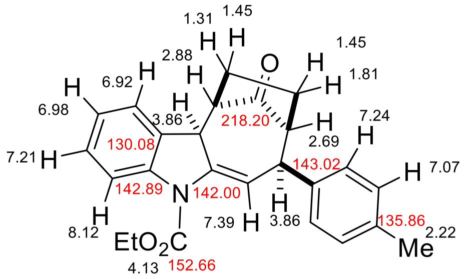

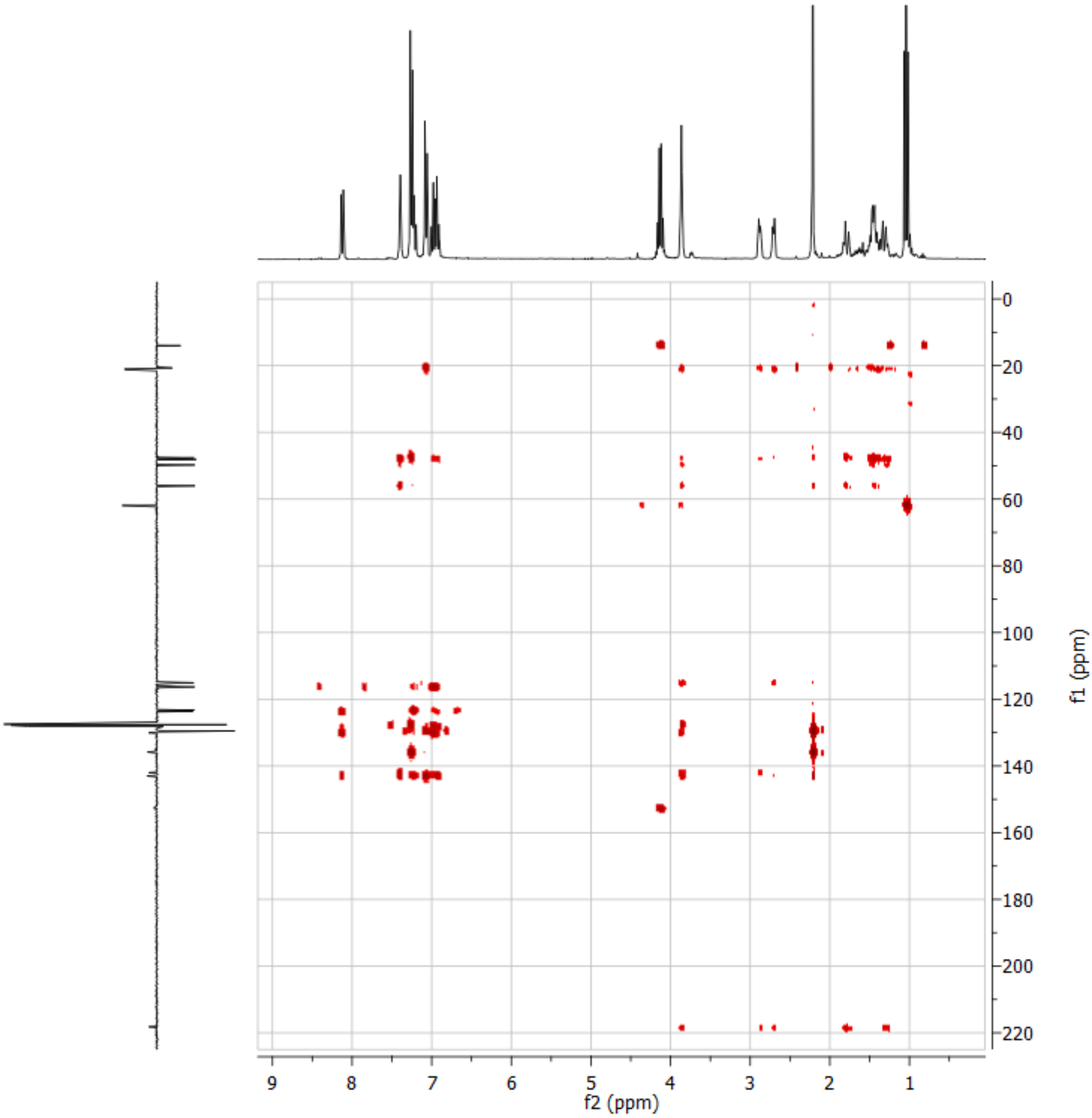

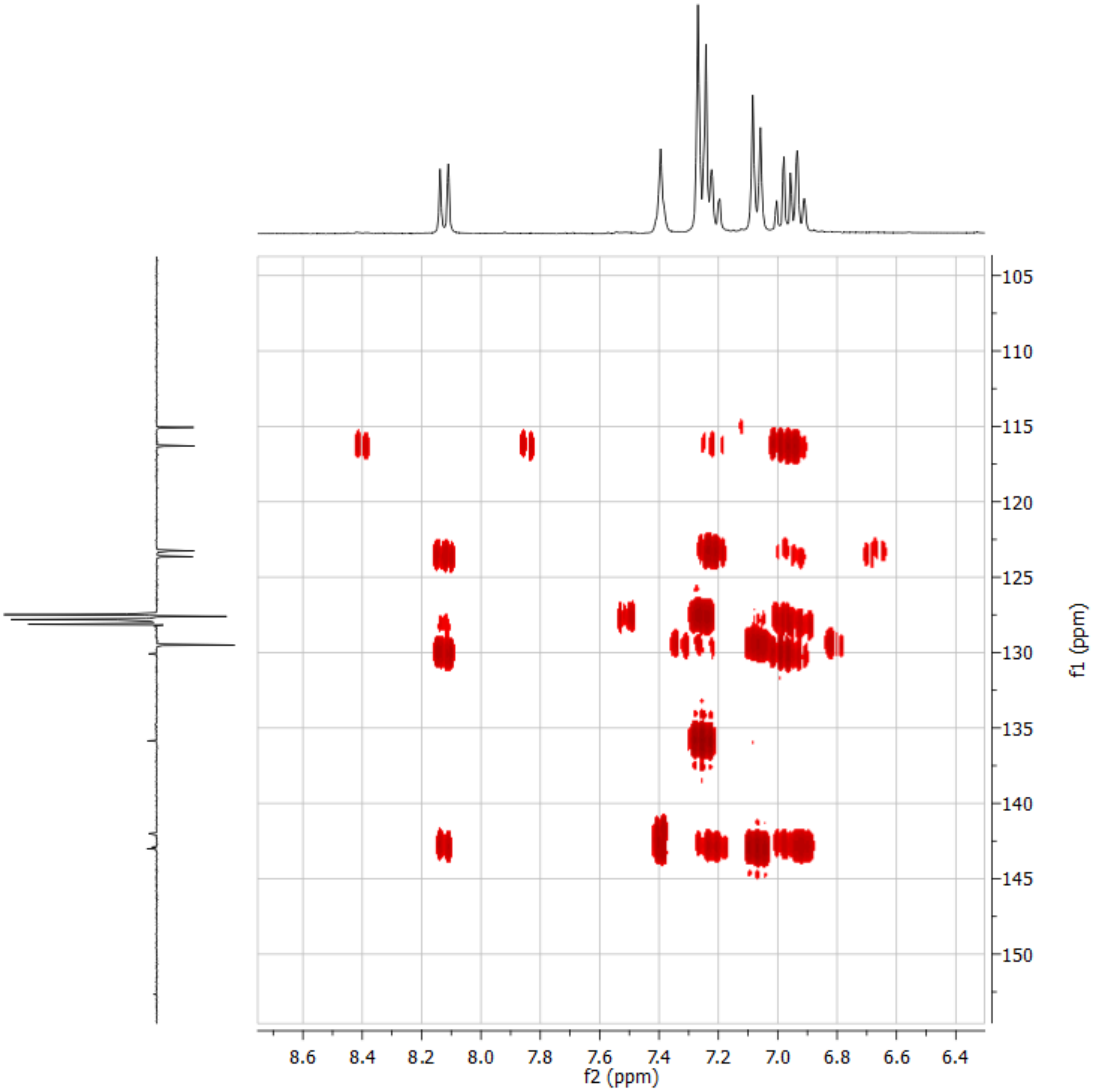

105
110
115
120
125
130
135
140
145
150
f1 (ppm)
8.6
8.4
8.2
8.0
7.8
7.6
7.4
7.2
7.0
6.8
6.6
6.4
f2 (ppm)

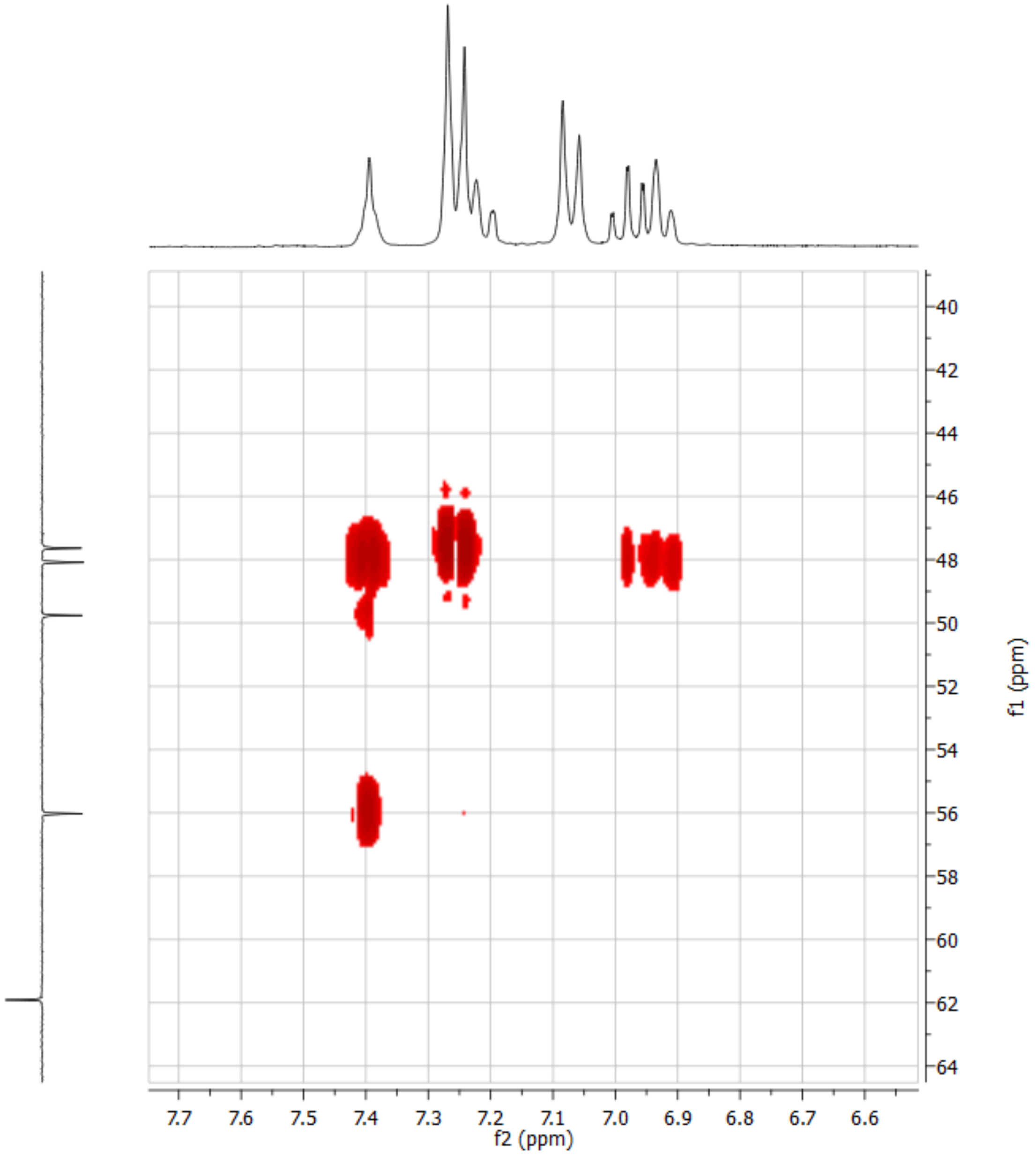
7.7
7.6
7.5
7.4
7.3
7.2
7.1
7.0
6.9
6.8
6.7
6.6
f2 (ppm)
40
42
44
46
48
50
52
54
56
58
60
62
64
f1 (ppm)

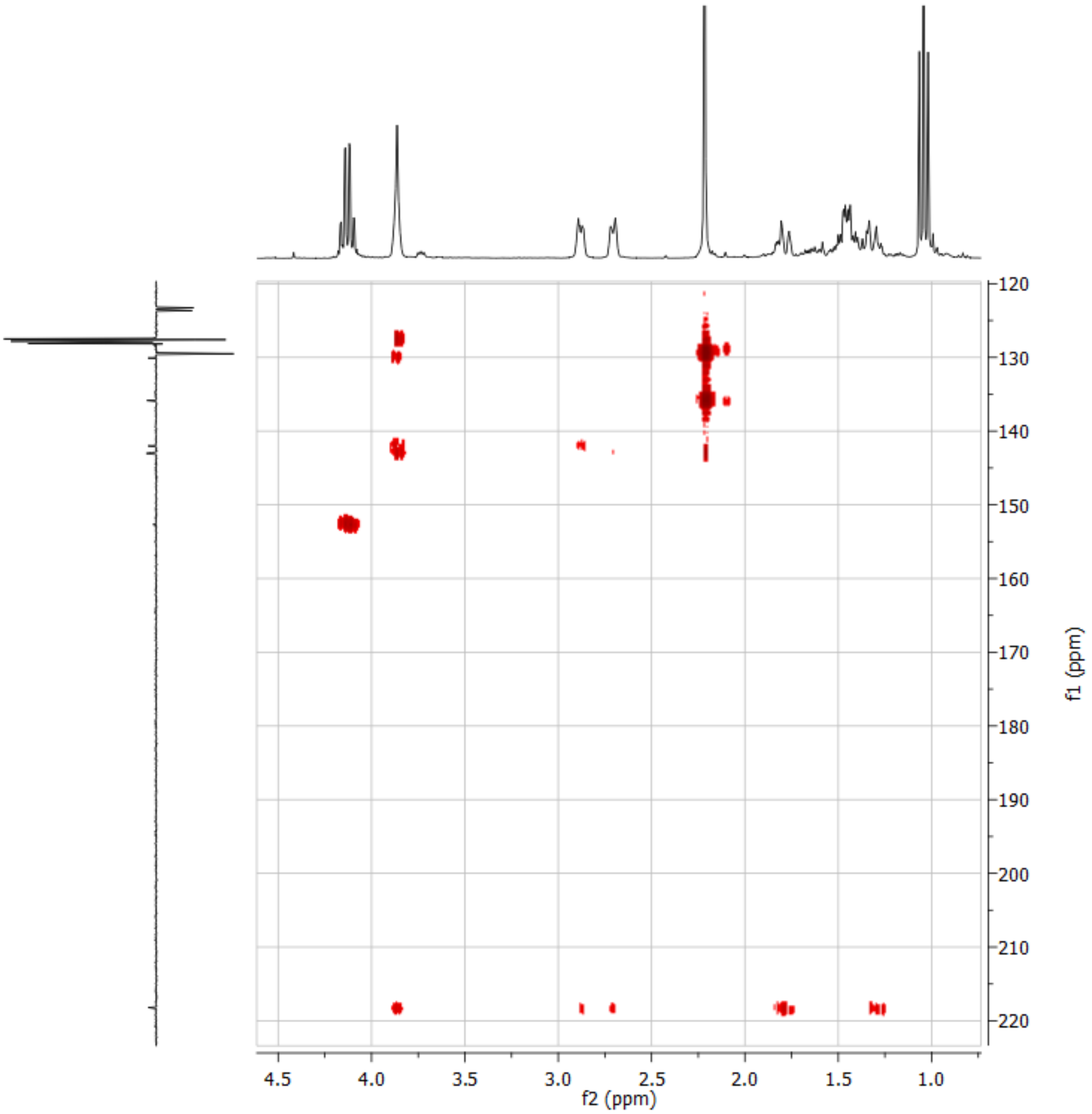

120
130
140
150
160
170
180
190
200
210
220
f1 (ppm)
4.5
4.0
3.5
3.0
2.5
2.0
1.5
1.0
f2 (ppm)

3i, COSY in C_6D_6 at T = 300 K

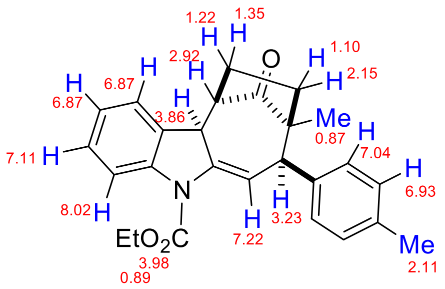

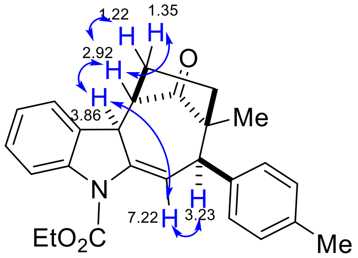

diagnostic cross picks

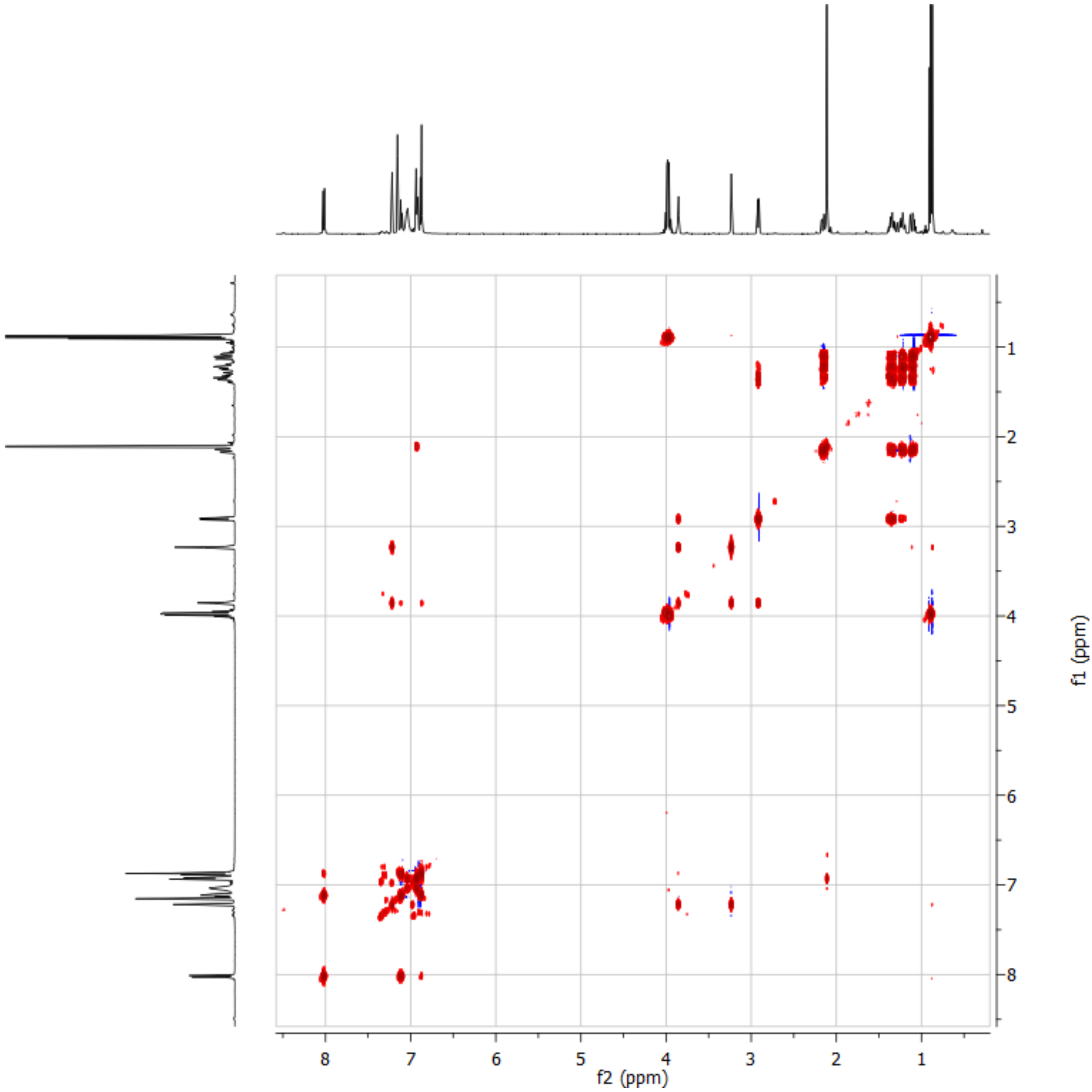

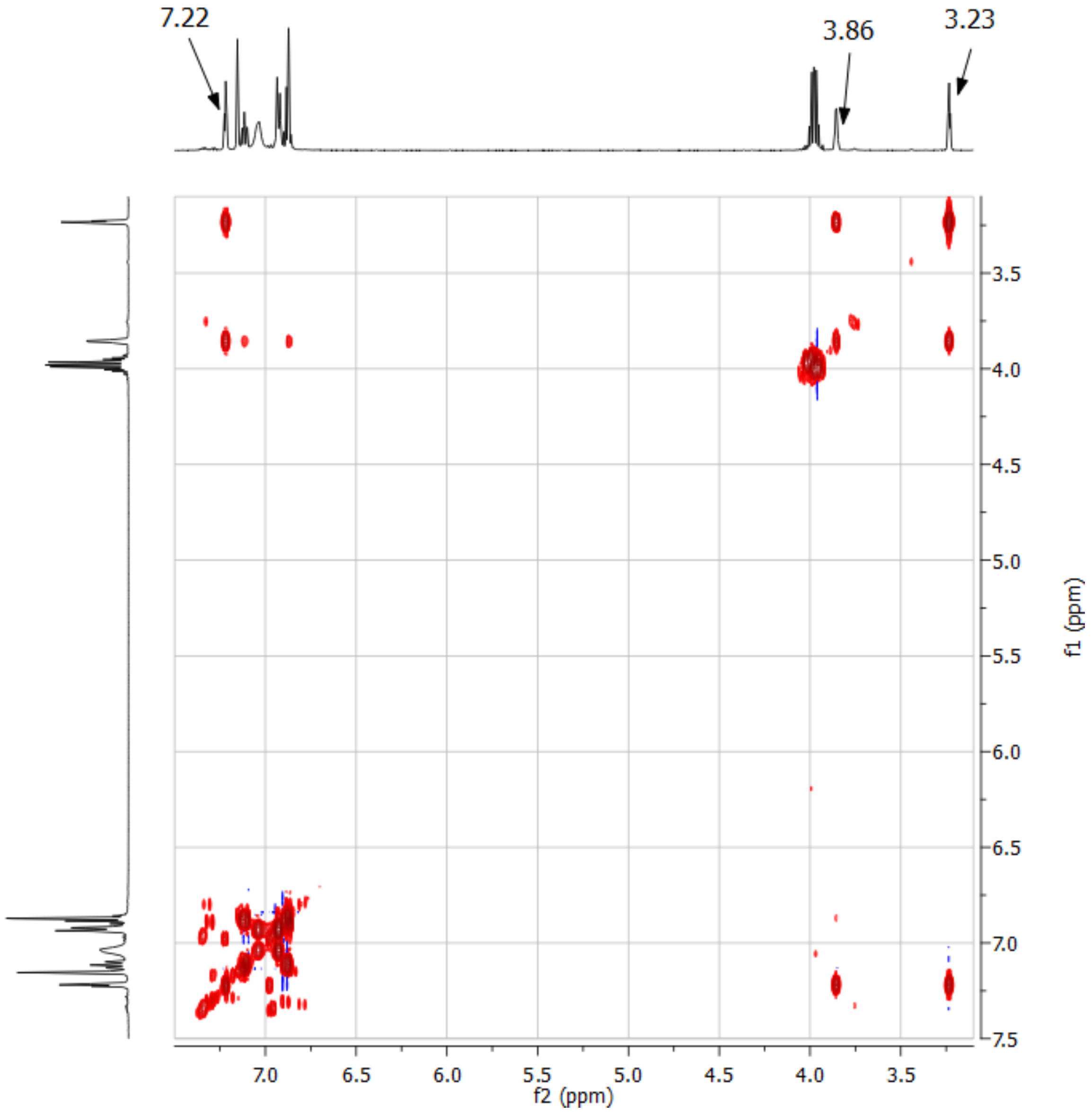
7.22
3.86
3.23
f1 (ppm)
f2 (ppm)

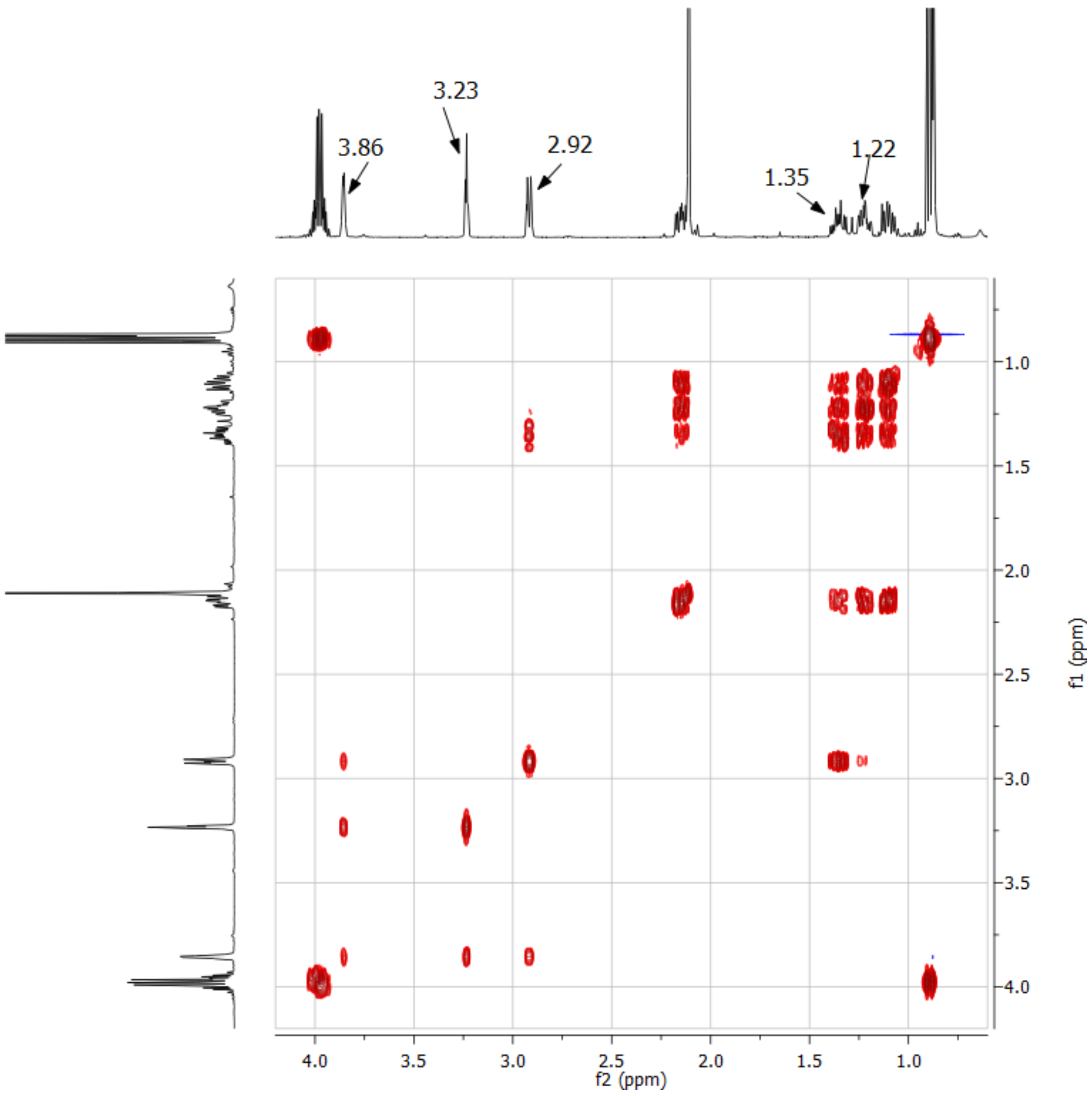
3.86
3.23
2.92
1.35
1.22
f2 (ppm)
f1 (ppm)

3i, HSQC in C_6D_6 at T = 300 K

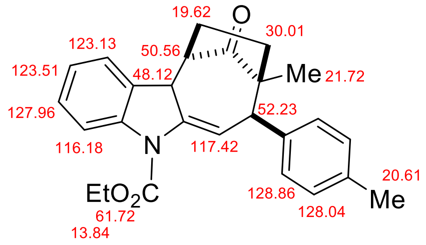

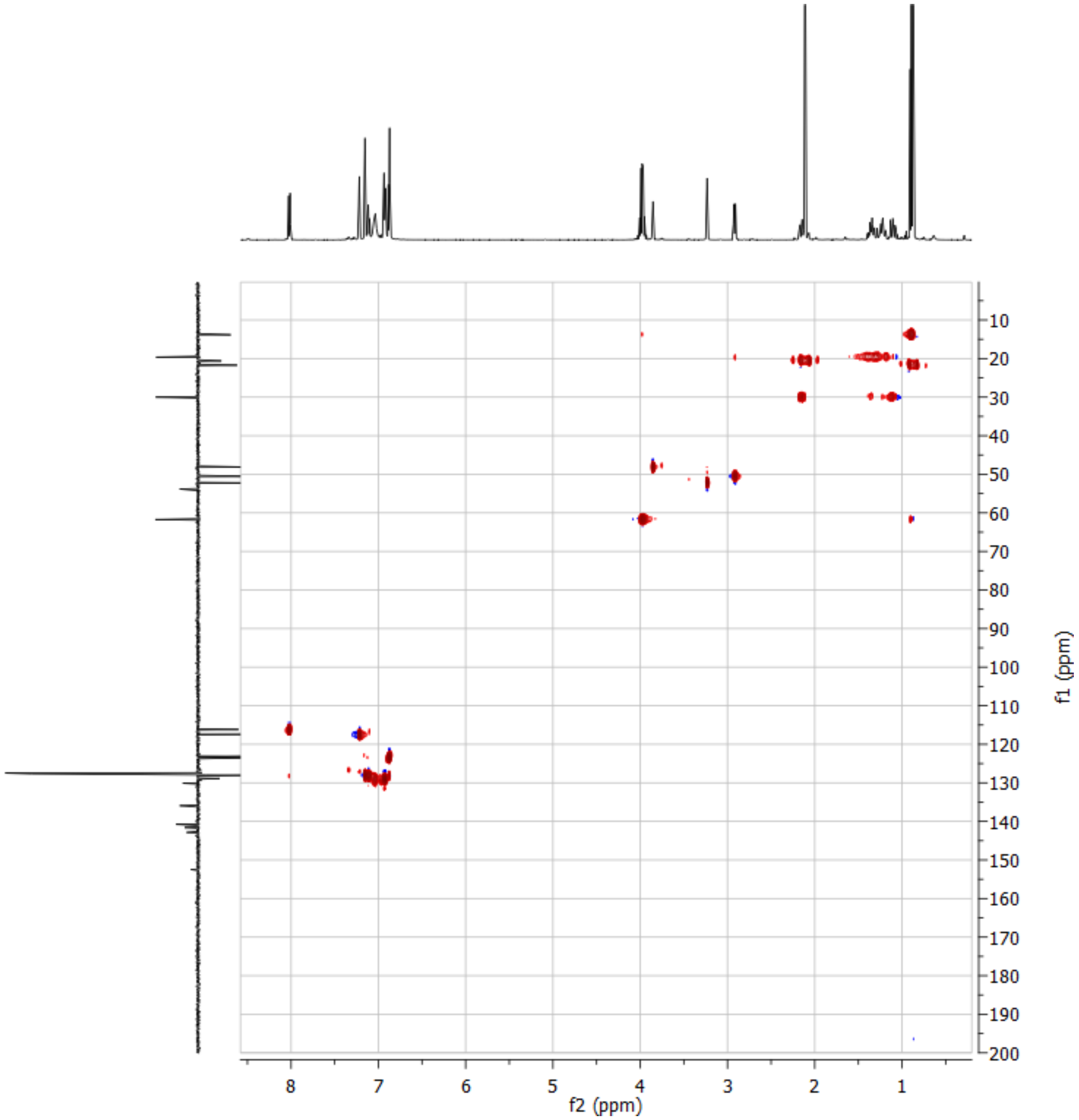

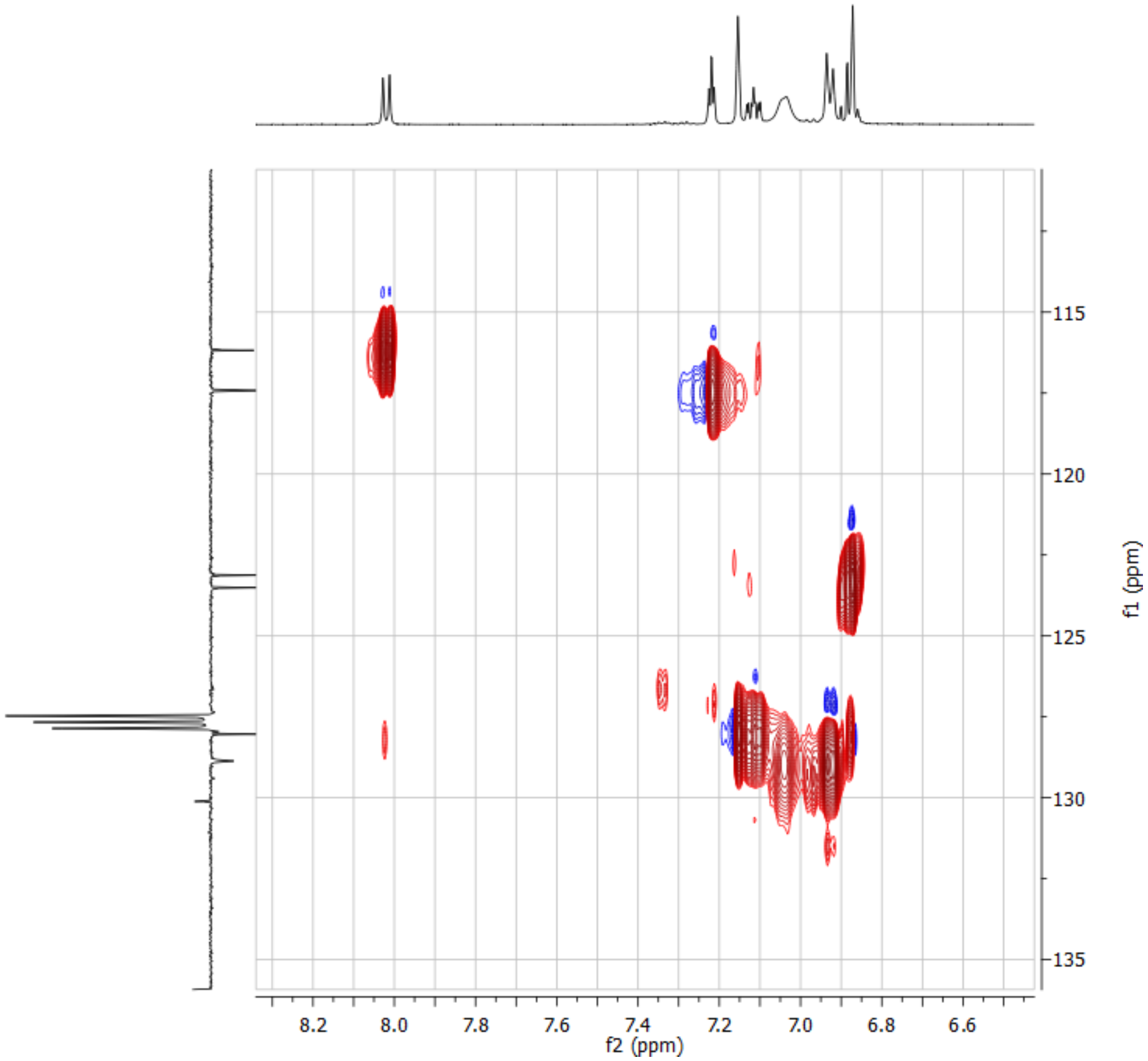

115
120
125
130
135
f1 (ppm)
8.2
8.0
7.8
7.6
7.4
7.2
7.0
6.8
6.6
f2 (ppm)

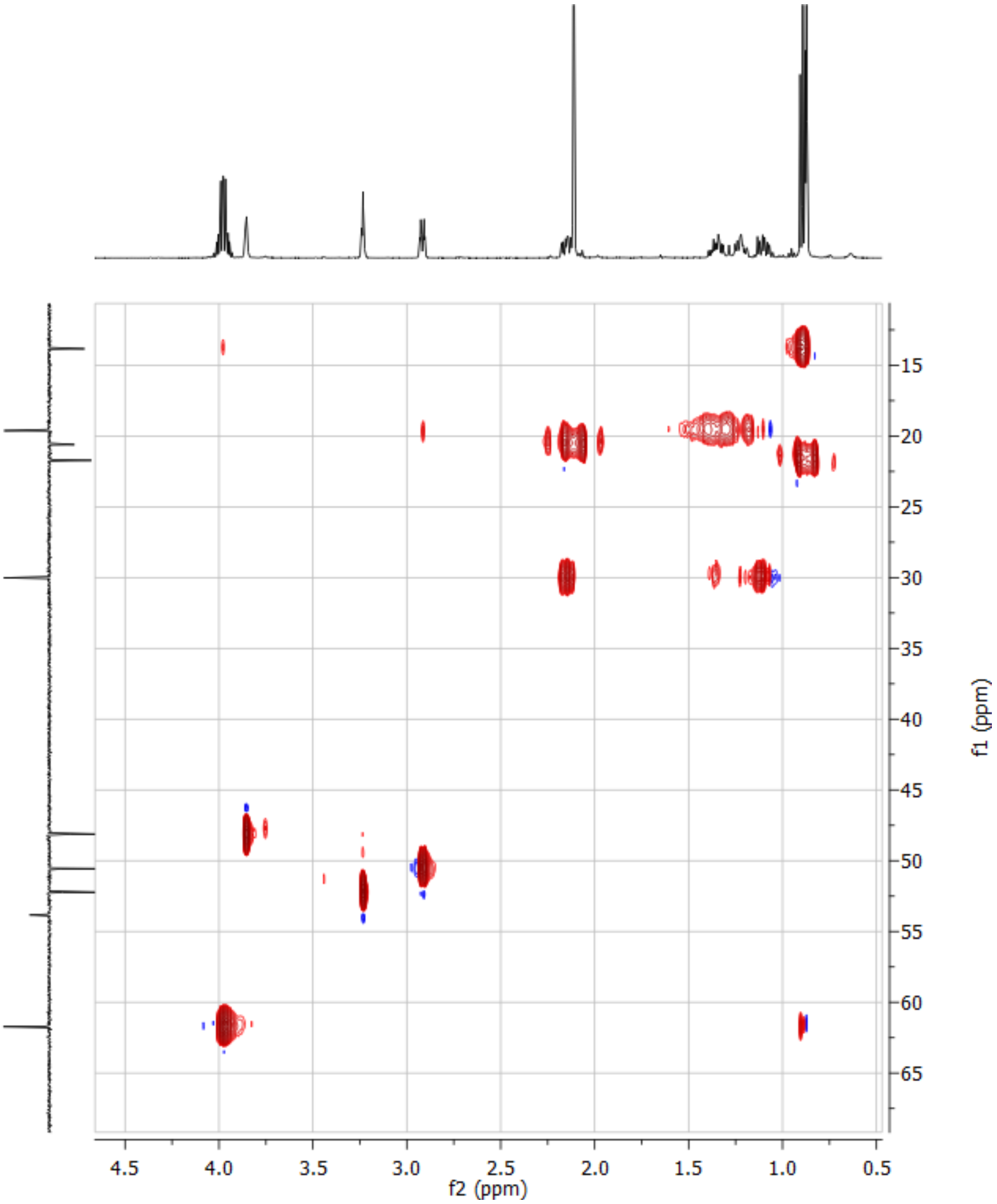
4.5
4.0
3.5
3.0
2.5
2.0
1.5
1.0
0.5
f2 (ppm)
15
20
25
30
35
40
45
50
55
60
65
f1 (ppm)

3i, NOESY in C_6D_6 at T = 300 K

For a better understanding of the diagnostic NOESY interactions, beside a picture reporting diagnostic cross picks with the use of arrows, we report several views of a MM2 minimized 3D model of **3i**.

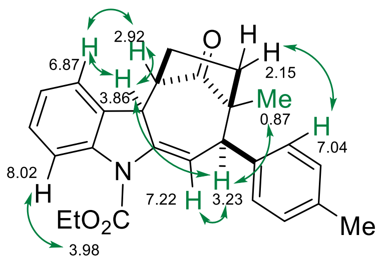

diagnostic cross picks

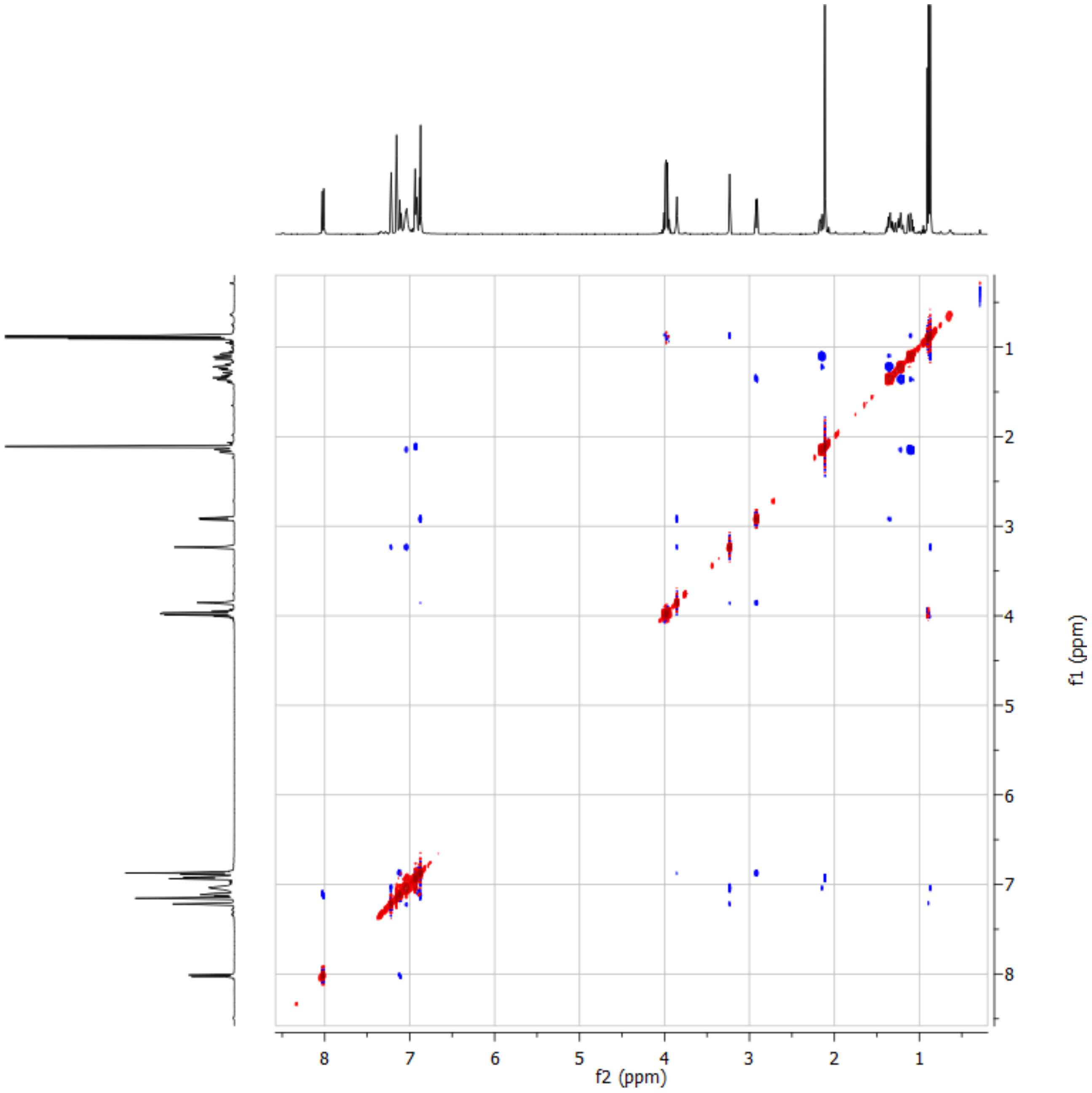

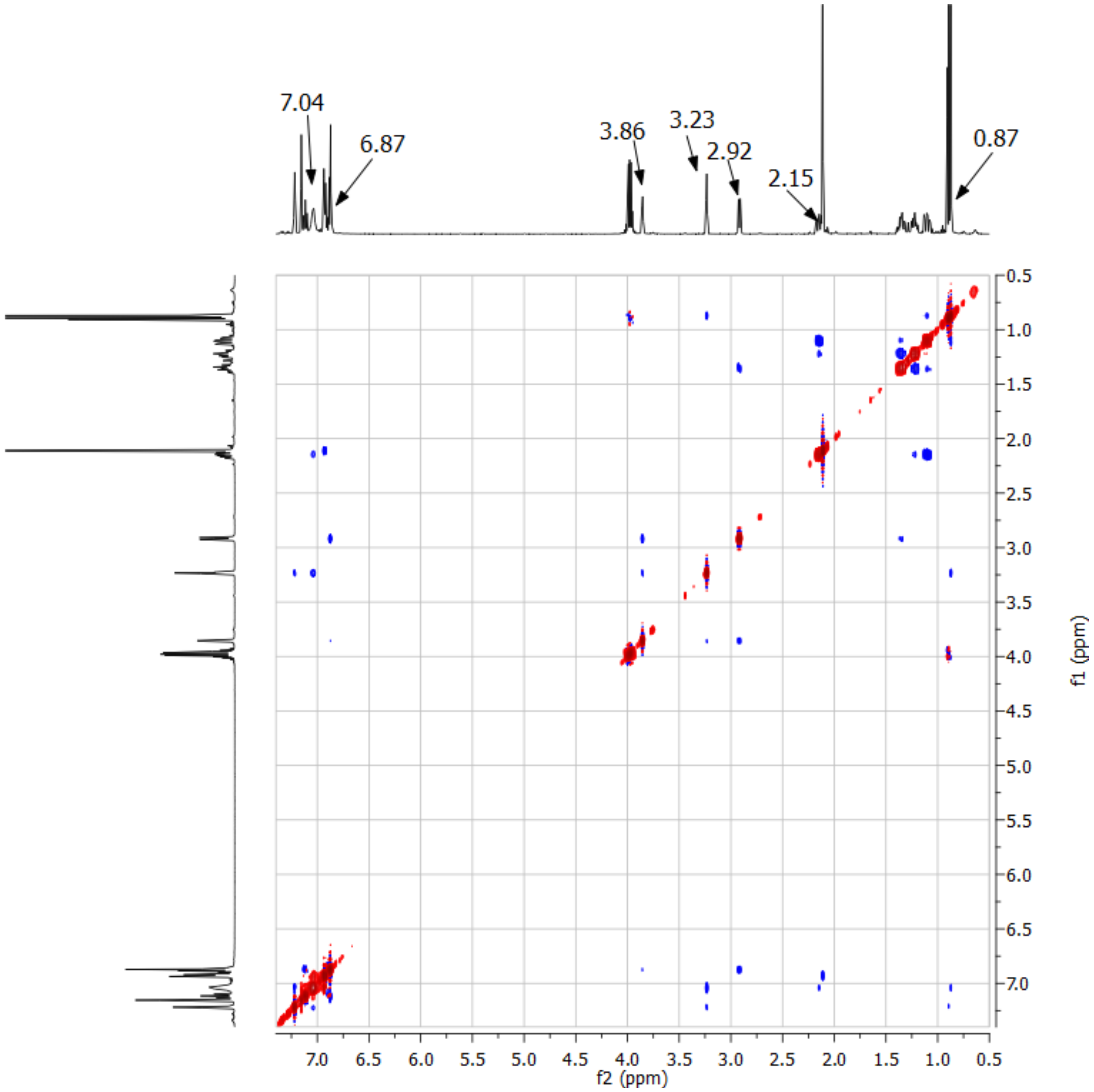
7.04
6.87
3.86
3.23
2.92
2.15
0.87
f2 (ppm)
f1 (ppm)

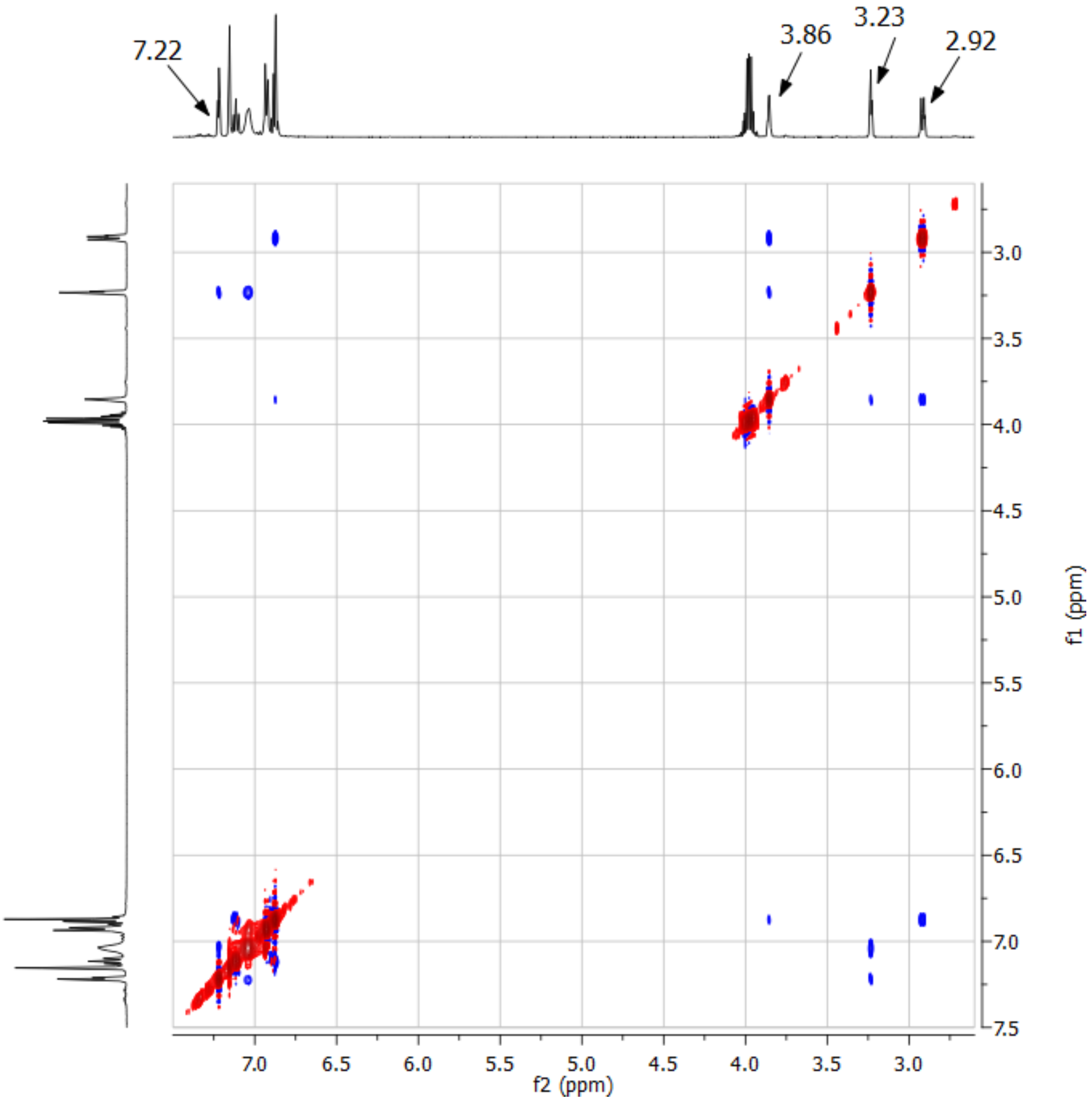
7.22
3.86
3.23
2.92
f2 (ppm)
f1 (ppm)

3i, MM2 minimized 3D structure

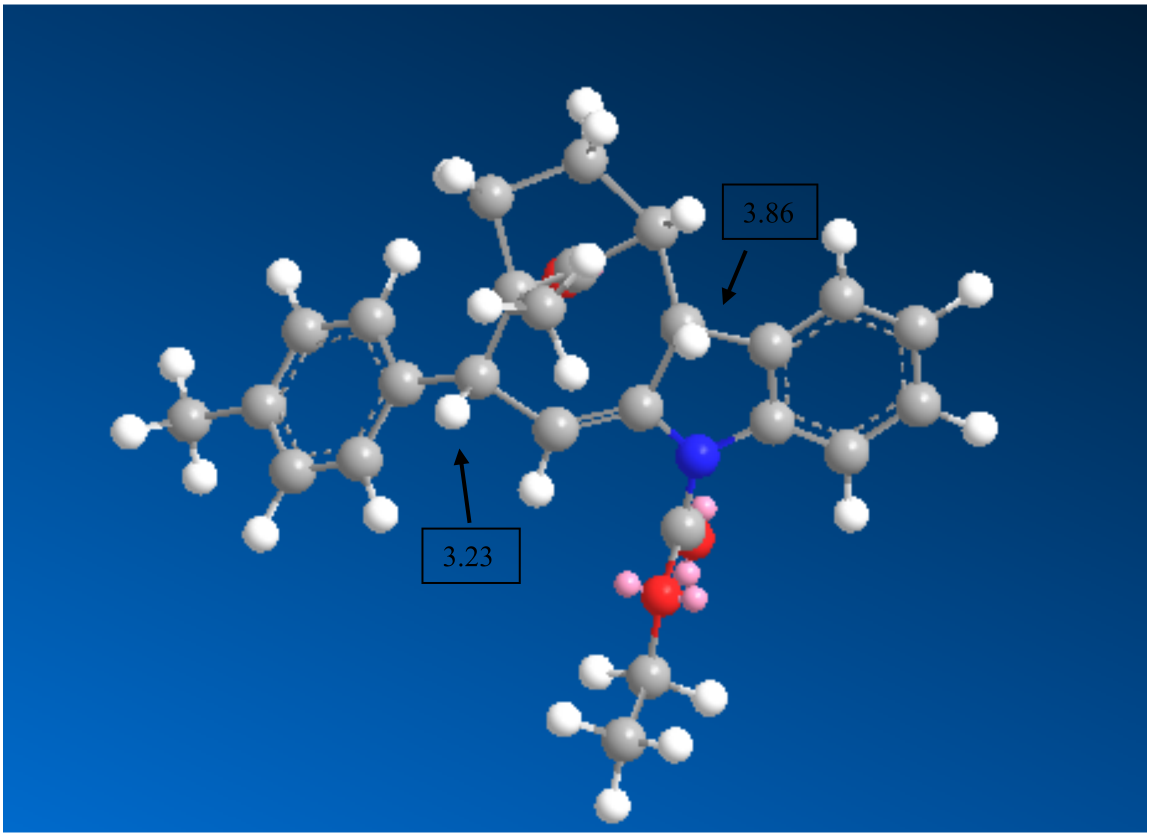

Figure S3: MM2 minimized 3D structure of **3i.**

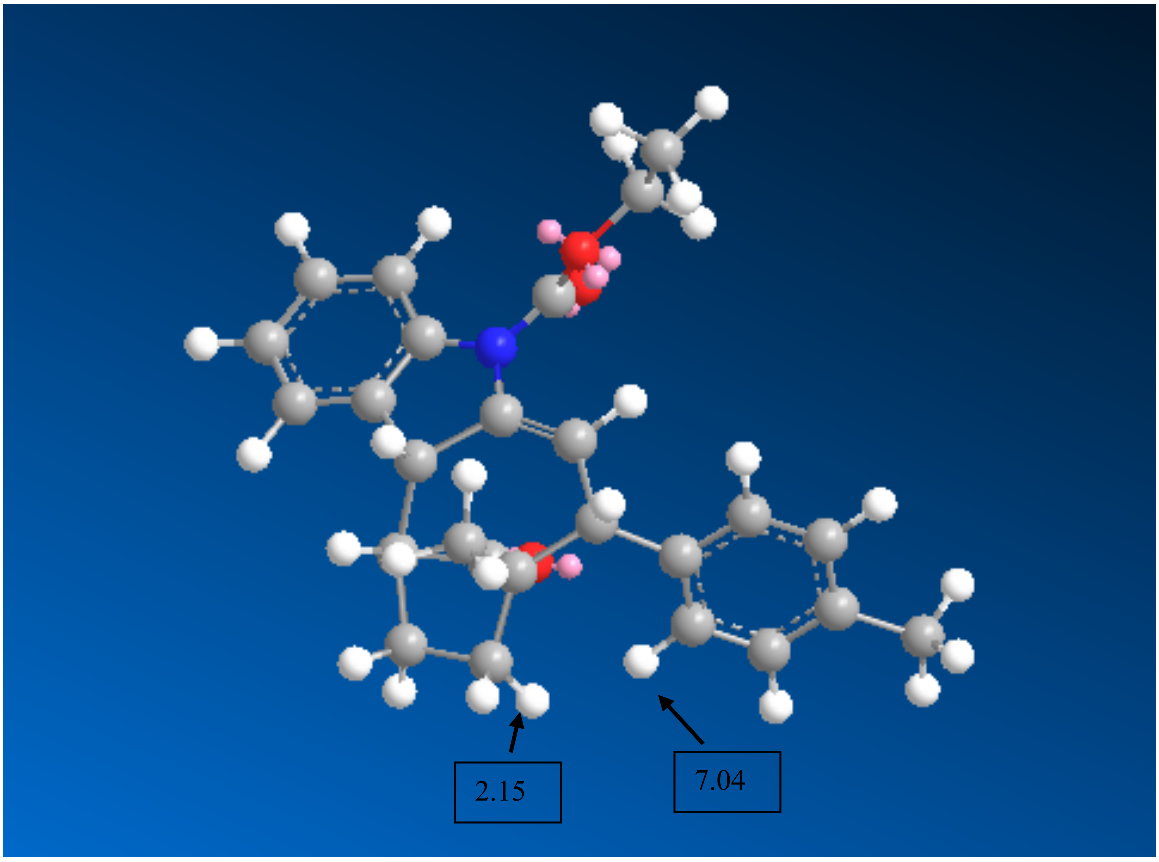

Figure S4: MM2 minimized 3D structure of **3i.**

3j, COSY in C_6D_6 at T = 300 K

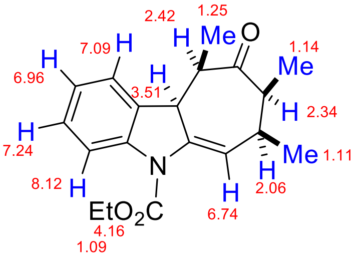

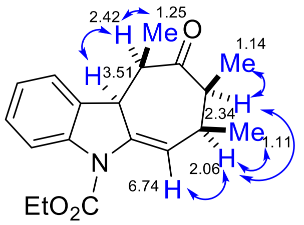

diagnostic cross picks

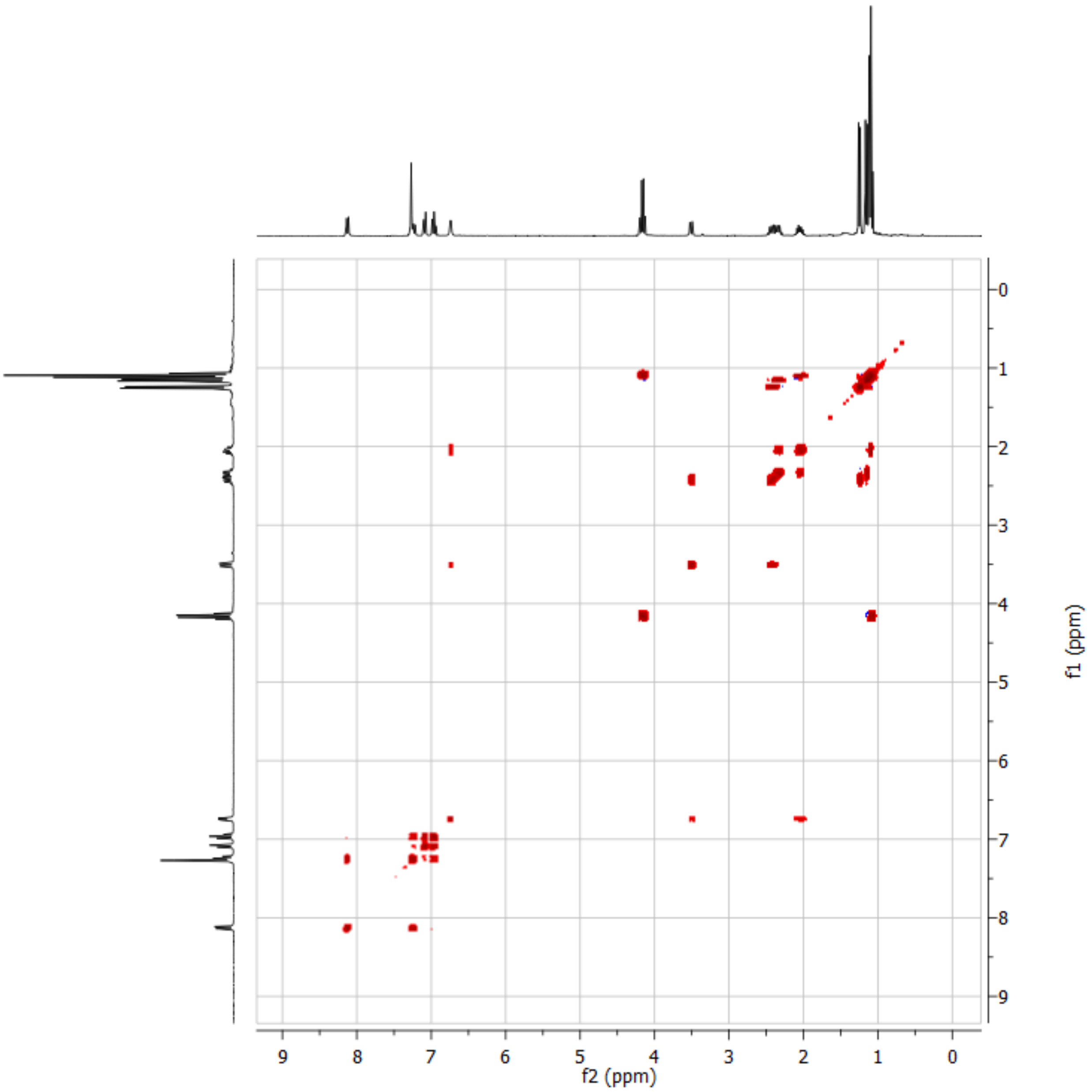

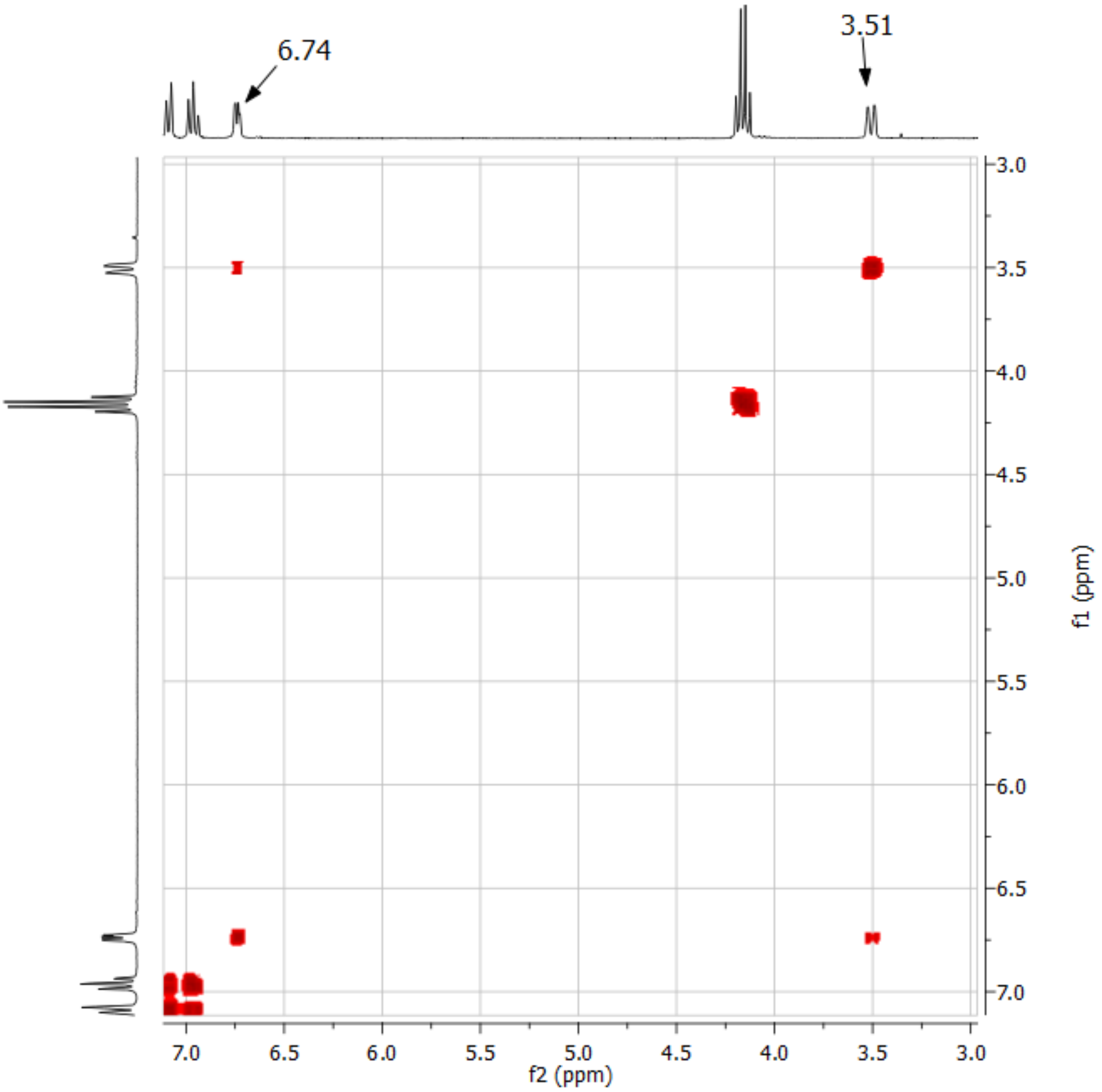

6.74
3.51
f1 (ppm)
f2 (ppm)
3.0
3.5
4.0
4.5
5.0
5.5
6.0
6.5
7.0

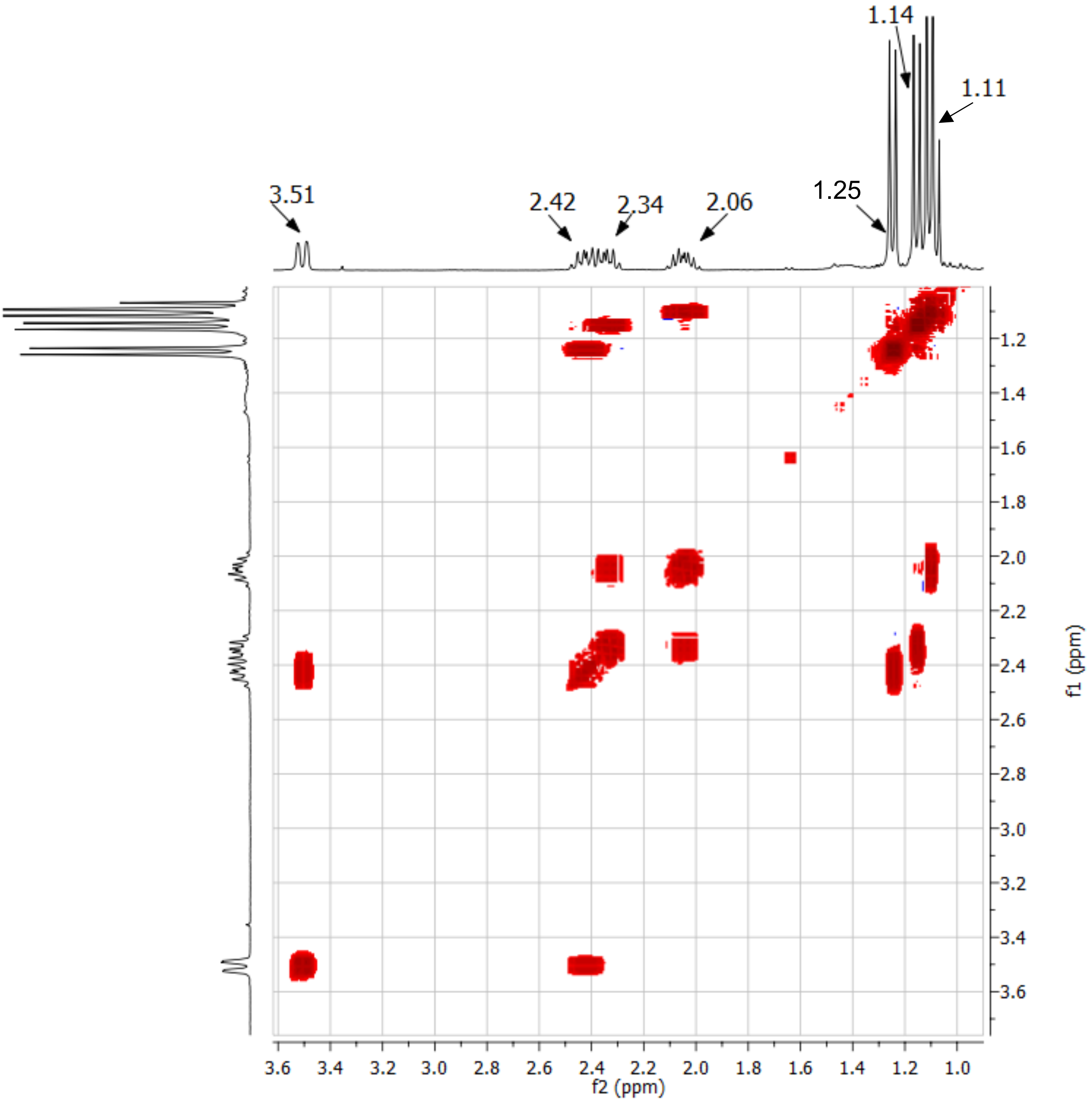

3.51
2.42
2.34
2.06
1.25
1.14
1.11
f2 (ppm)
f1 (ppm)

3j, HSQC in C_6D_6 at T = 300 K

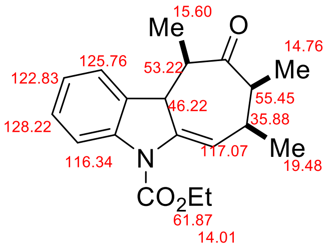

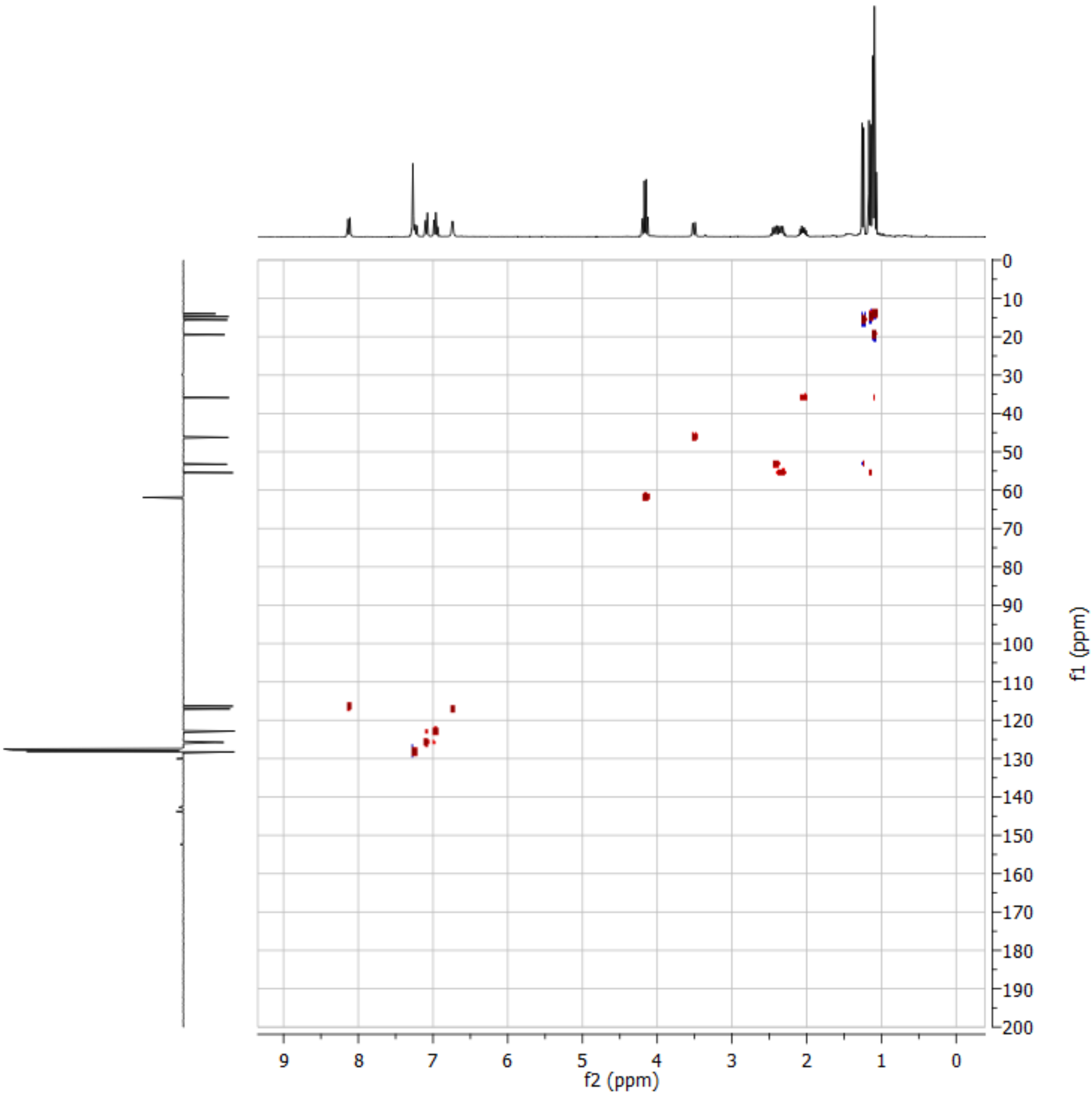

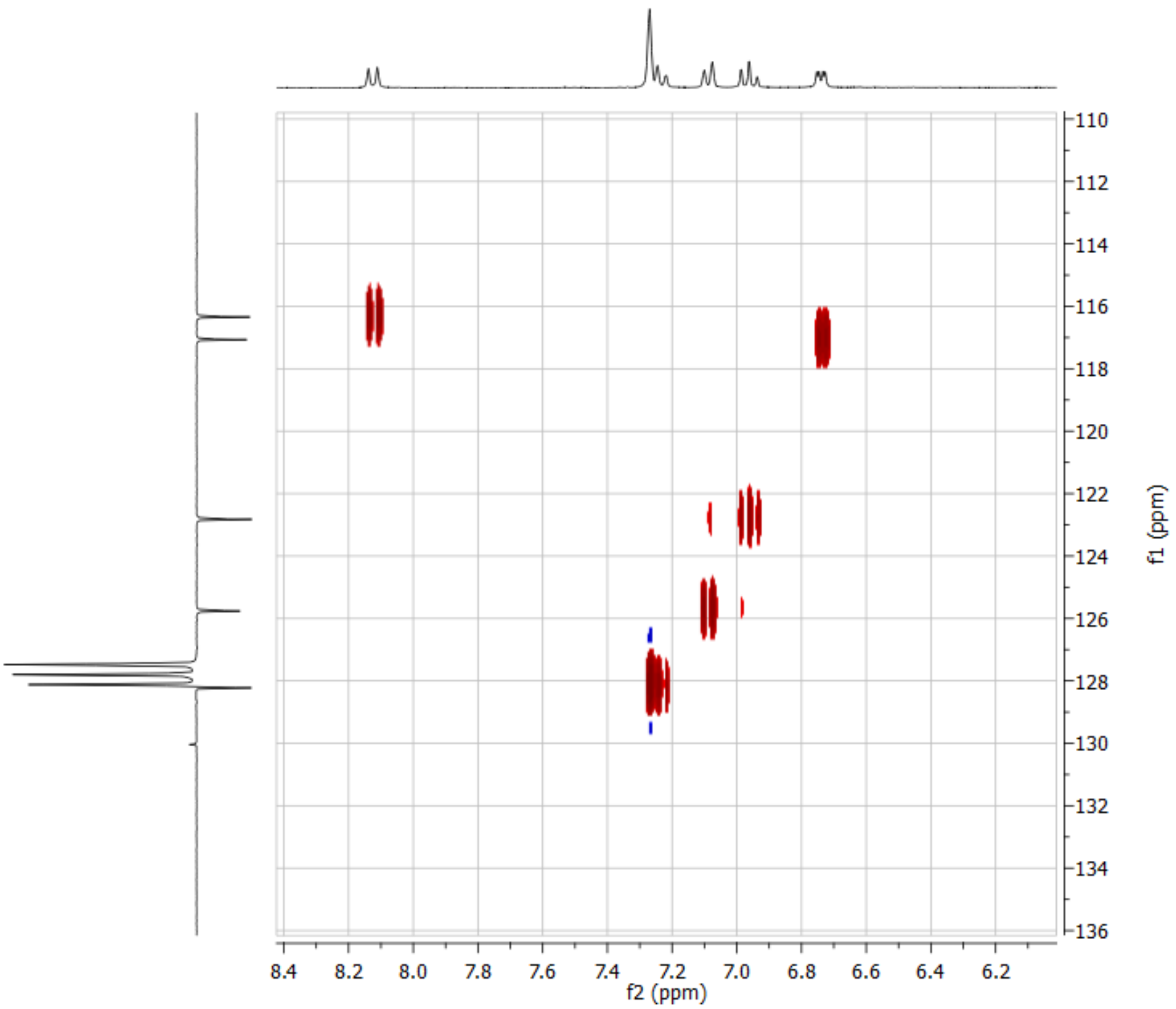

110
112
114
116
118
120
122
124
126
128
130
132
134
136
f1 (ppm)
8.4
8.2
8.0
7.8
7.6
7.4
7.2
7.0
6.8
6.6
6.4
6.2
f2 (ppm)

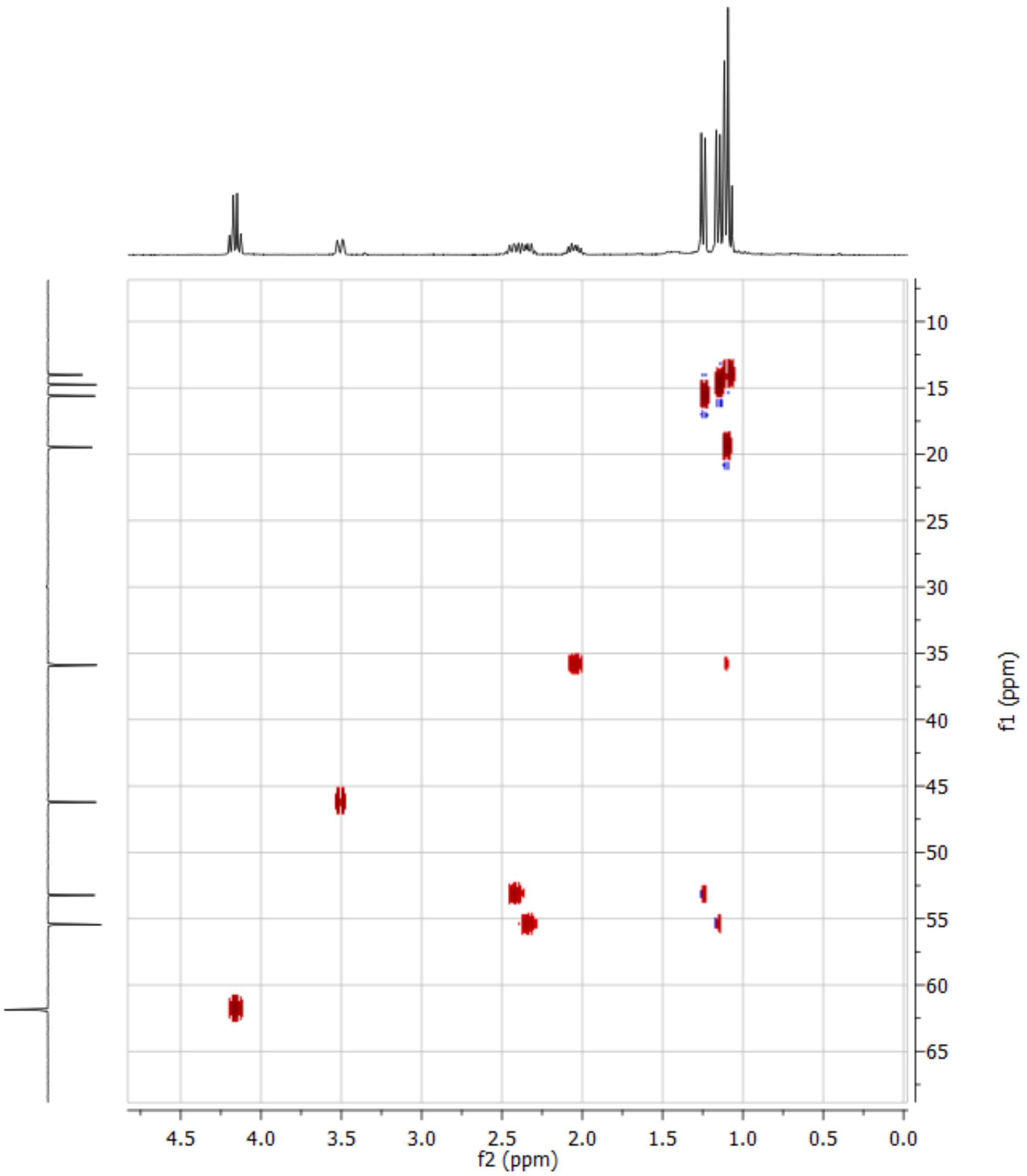

f1 (ppm)
f2 (ppm)

3j, NOESY in C_6D_6 at T = 300 K

For a better understanding of the diagnostic NOESY interactions, beside a picture reporting diagnostic cross picks with the use of arrows, we report a view of a MM2 minimized 3D model of **3j**.

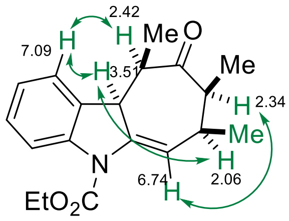

diagnostic cross picks

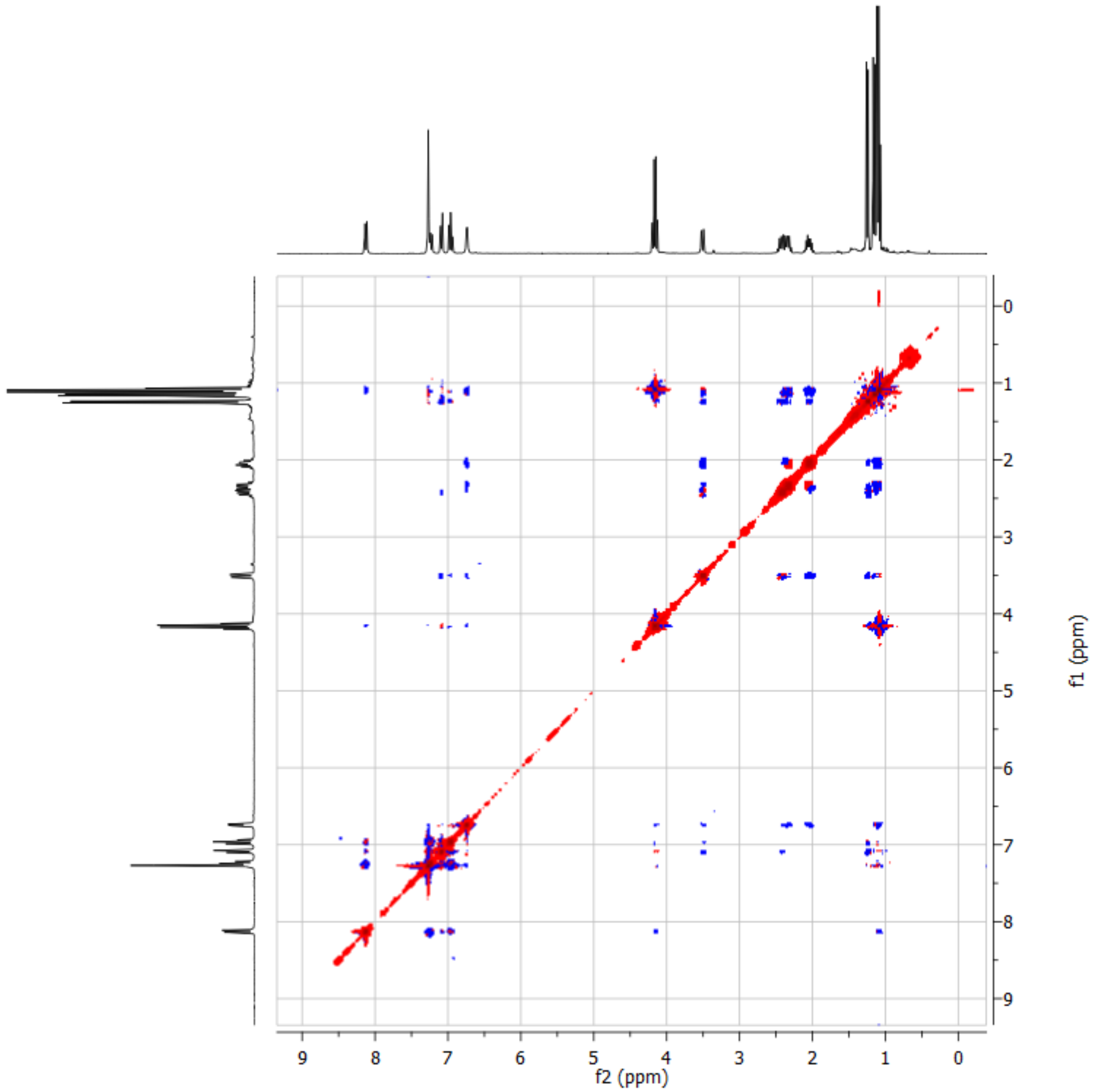

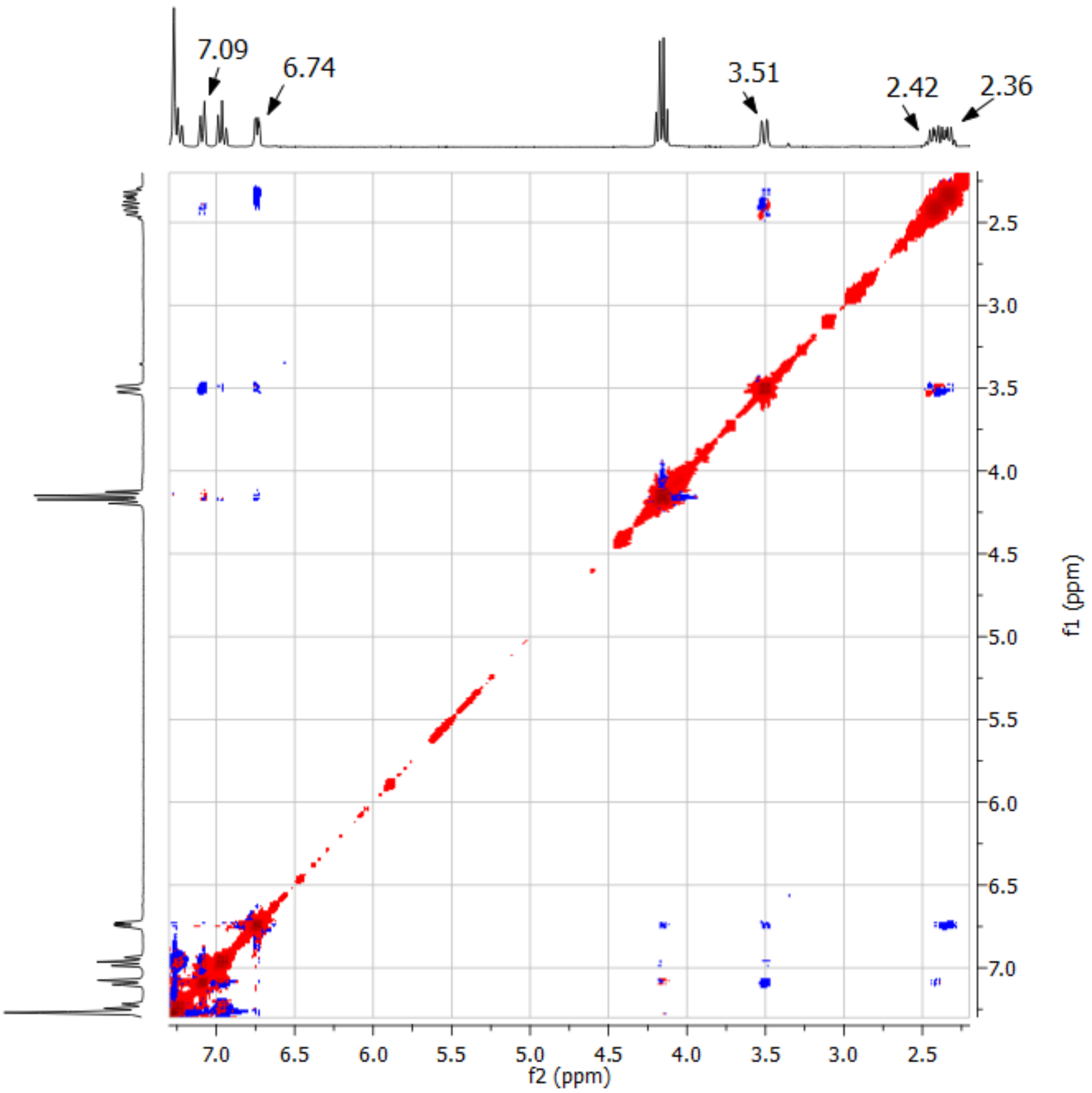

7.09
6.74
3.51
2.42
2.36
f2 (ppm)
f1 (ppm)

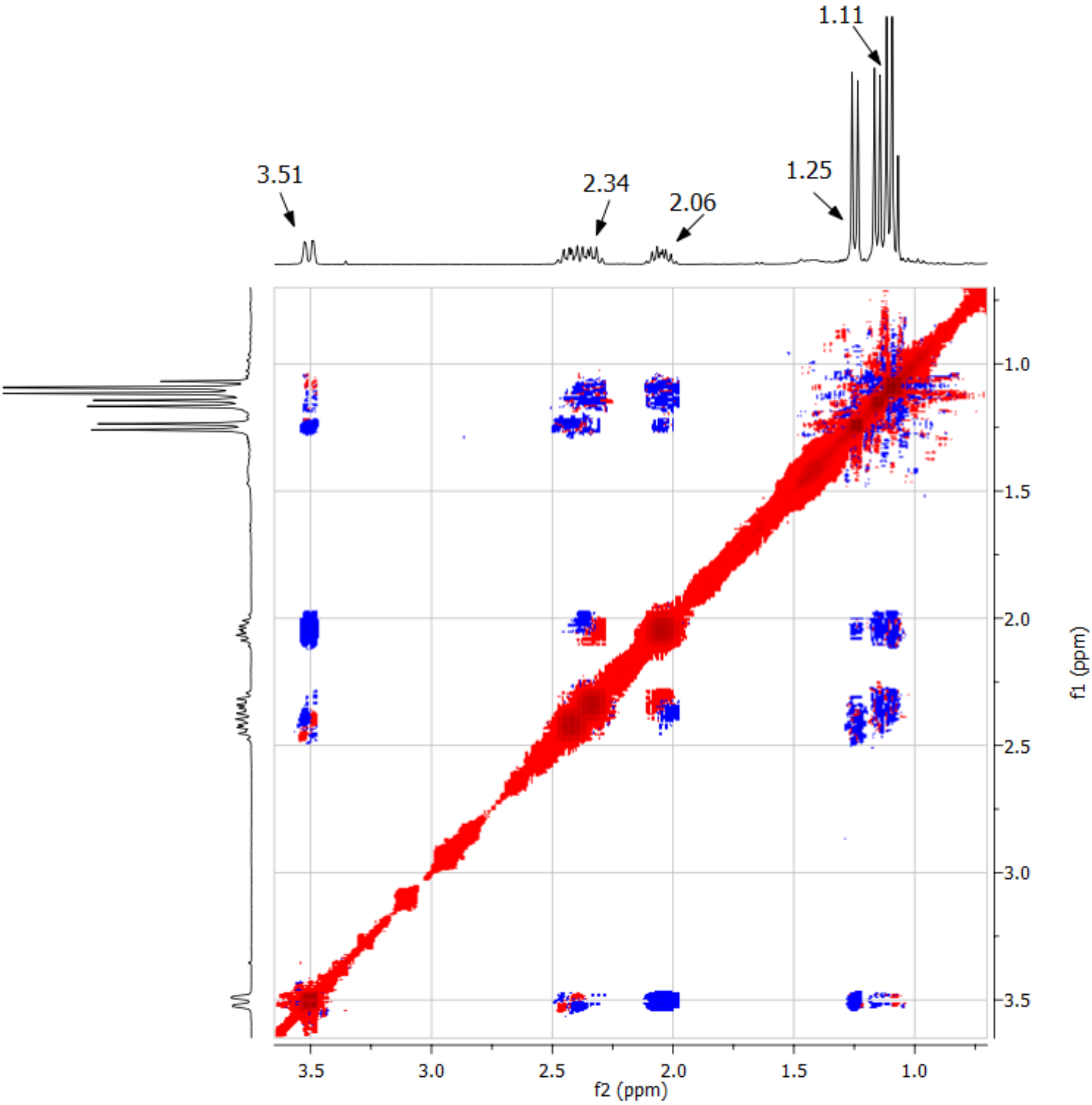
3.51
2.34
2.06
1.25
1.11
f1 (ppm)
1.0
1.5
2.0
2.5
3.0
3.5
3.5
3.0
2.5
2.0
1.5
1.0
f2 (ppm)

3j, MM2 minimized 3D structure

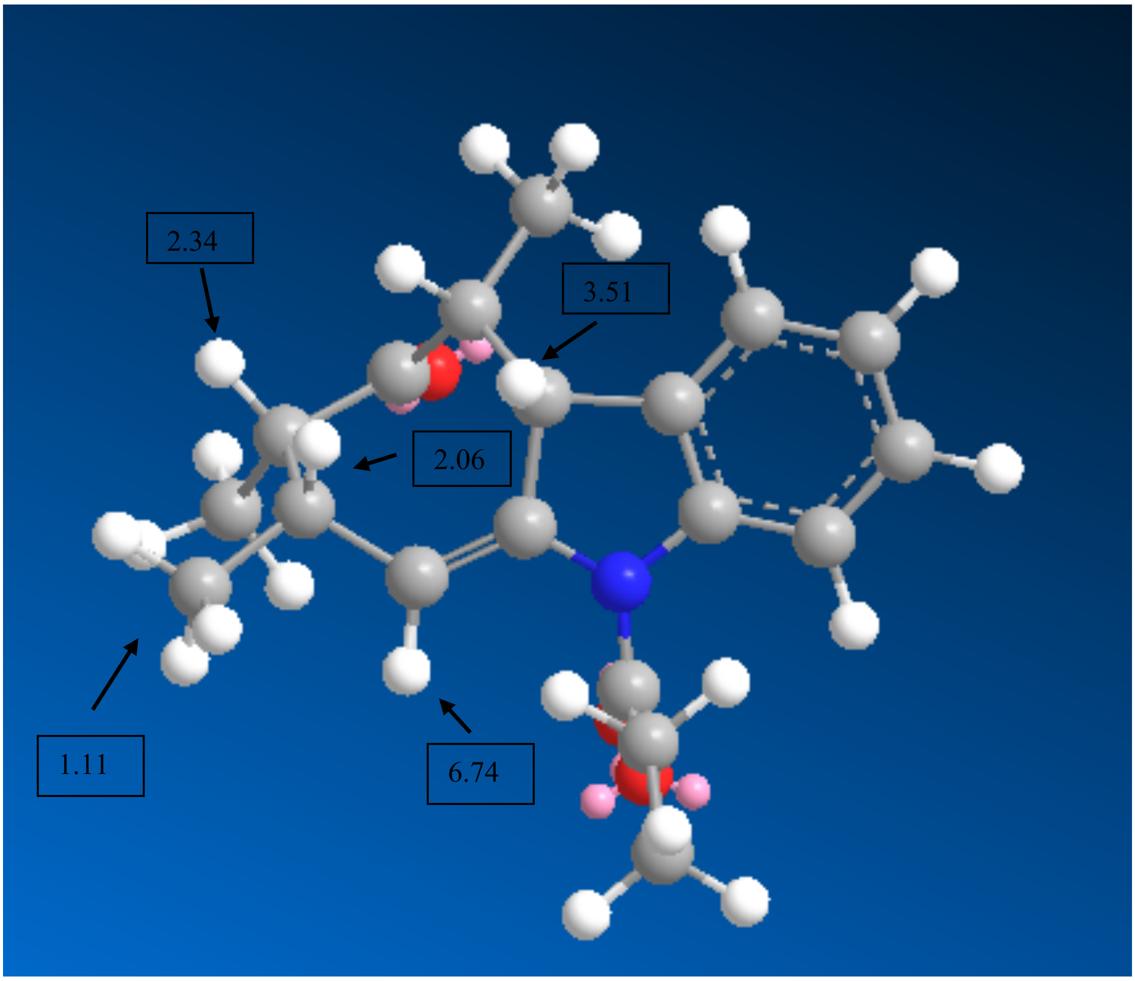

Figure S5: MM2 minimized 3D structure of **3j.**

3l, COSY in C_6D_6 at T = 300 K

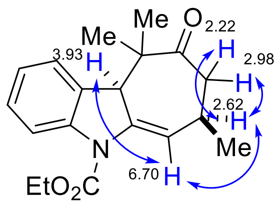

diagnostic cross picks

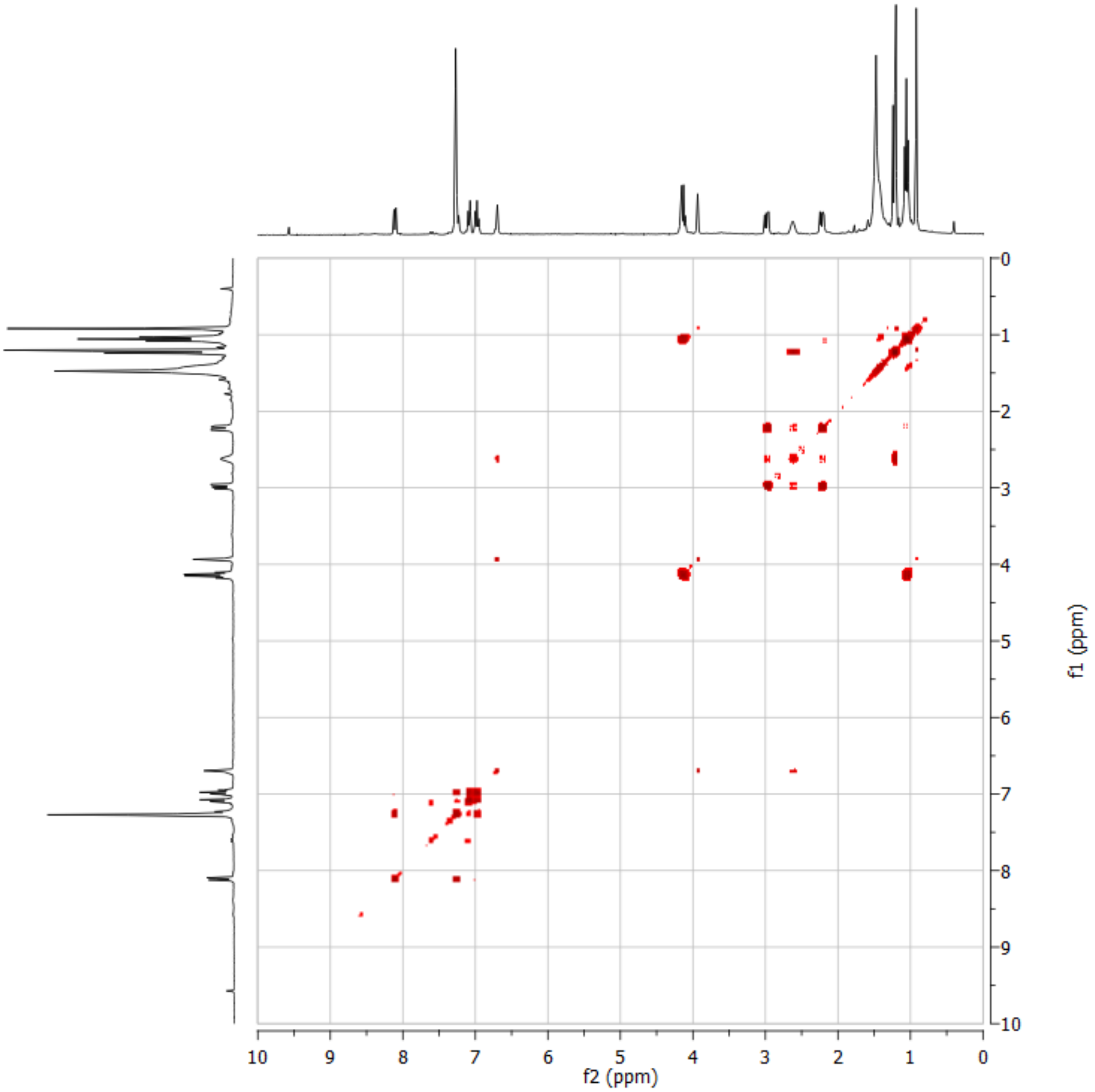

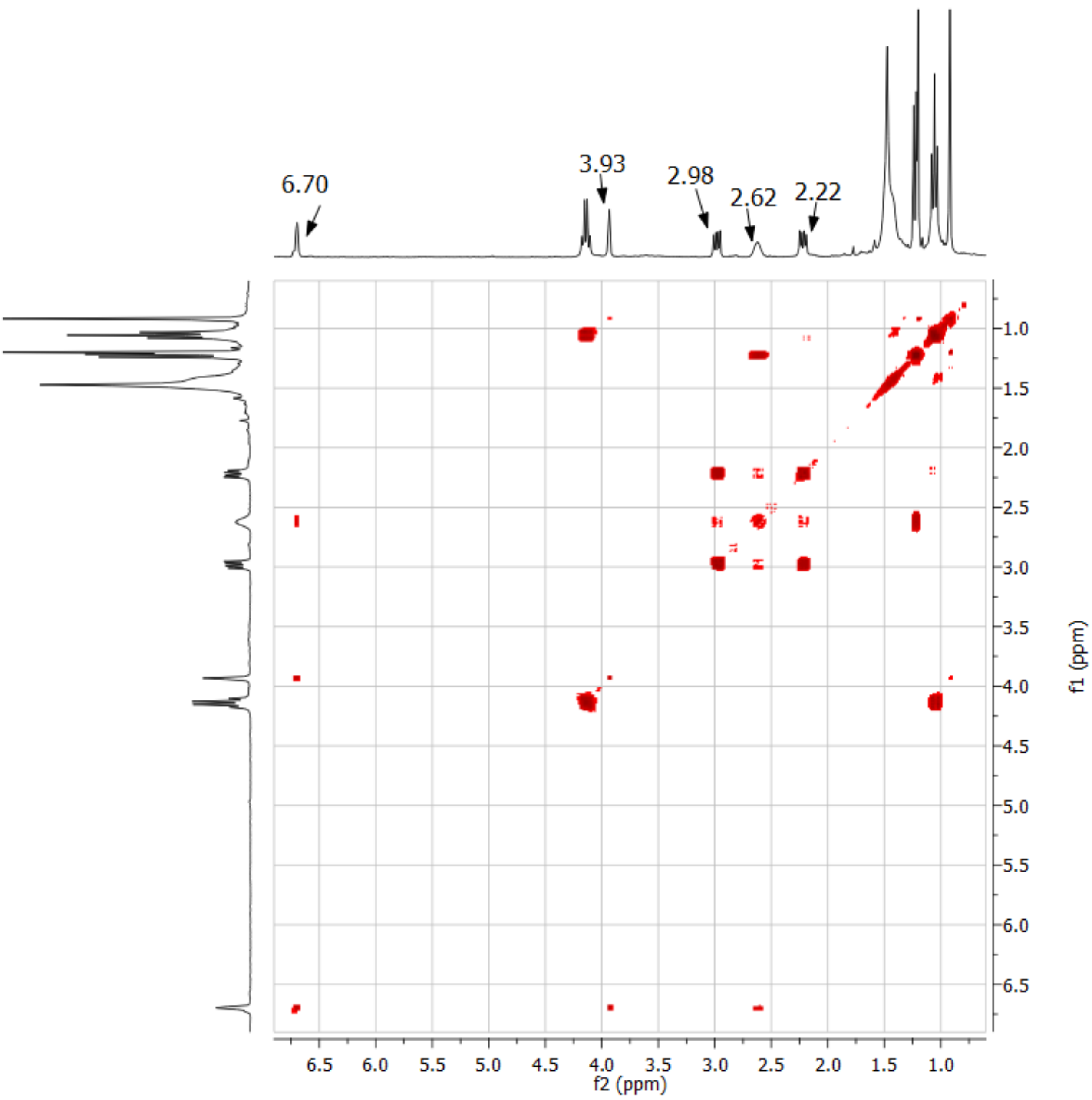
6.70
3.93
2.98
2.62
2.22
f2 (ppm)
f1 (ppm)

6a, COSY in $CDCl_3$ at T = 300 K

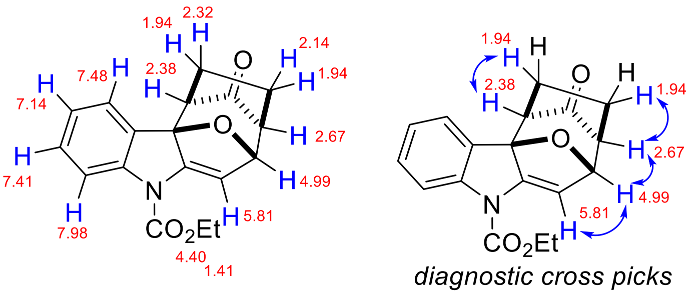

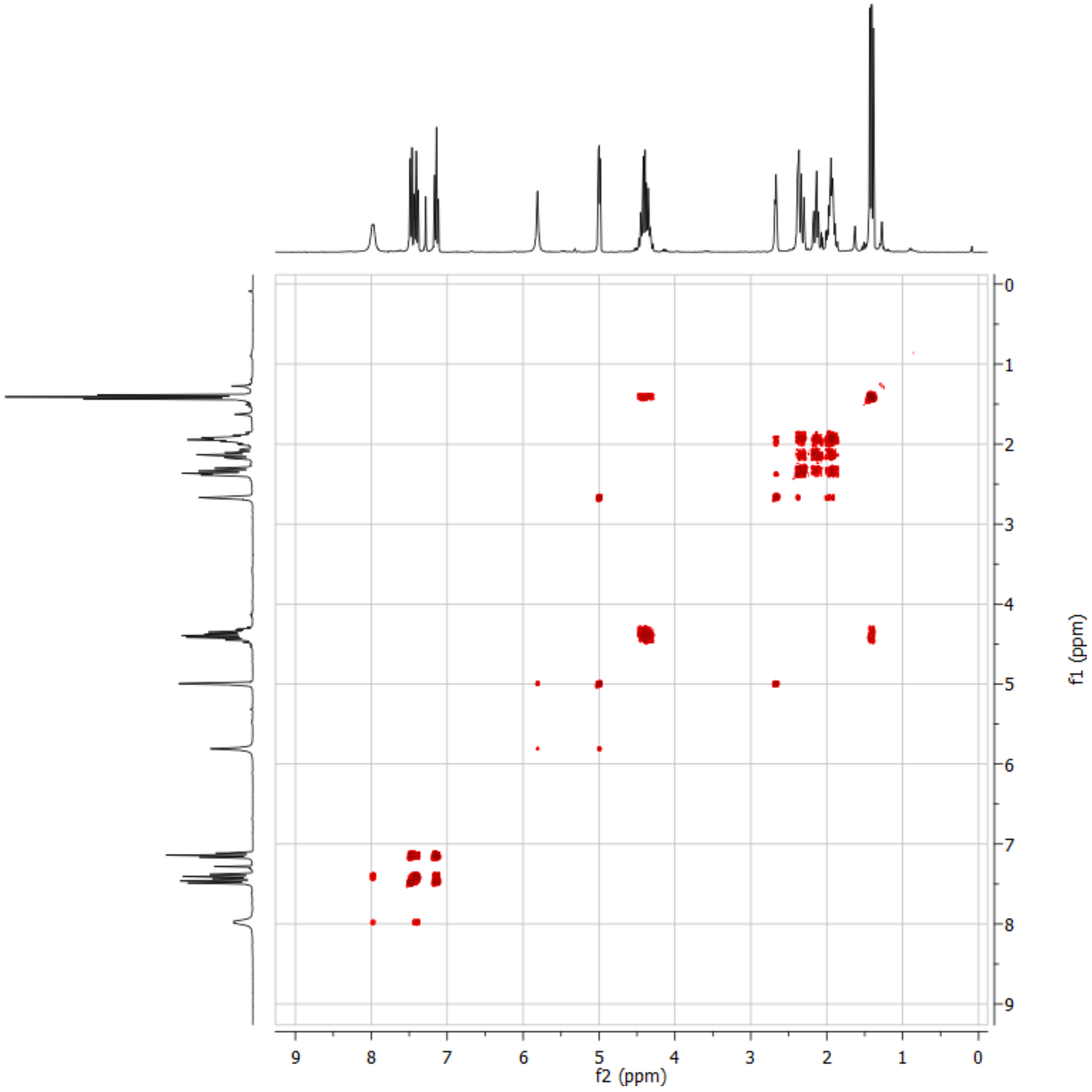

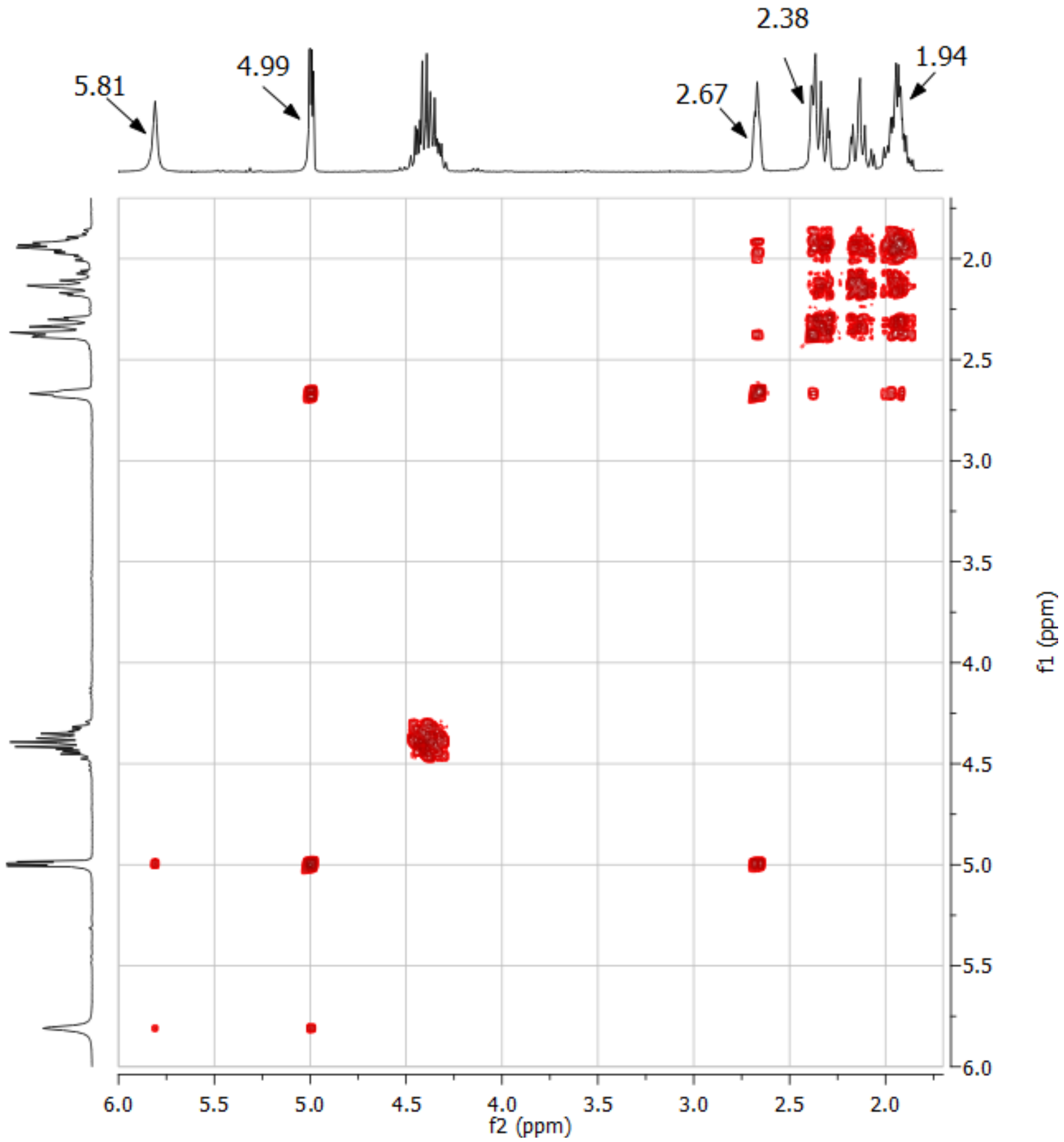
5.81
4.99
2.67
2.38
1.94
f2 (ppm)
f1 (ppm)

6a, HSQC in $CDCl_3$ at T = 300 K

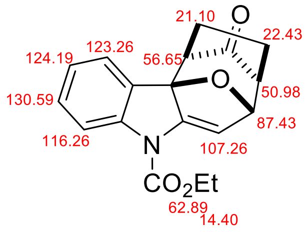

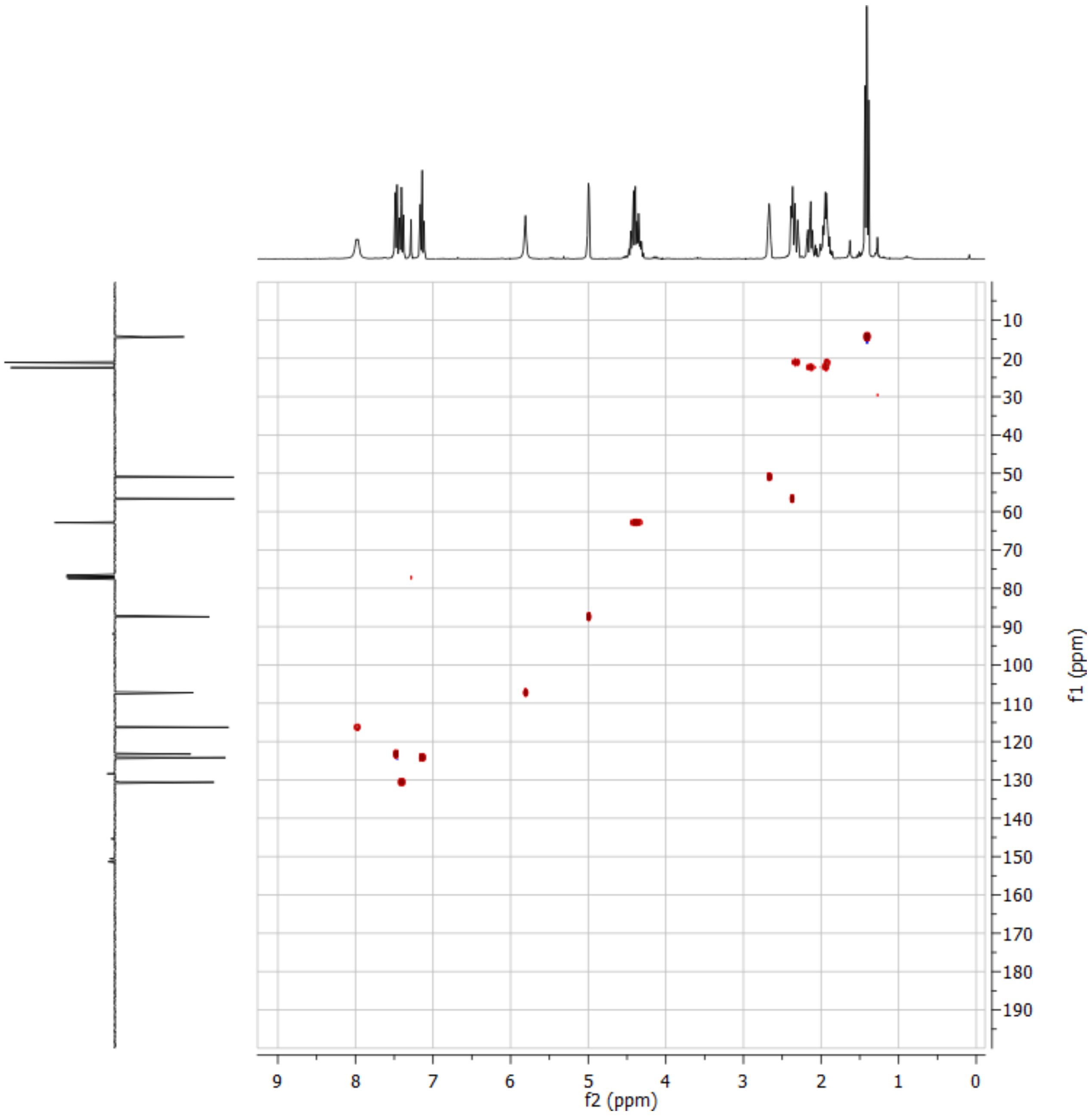

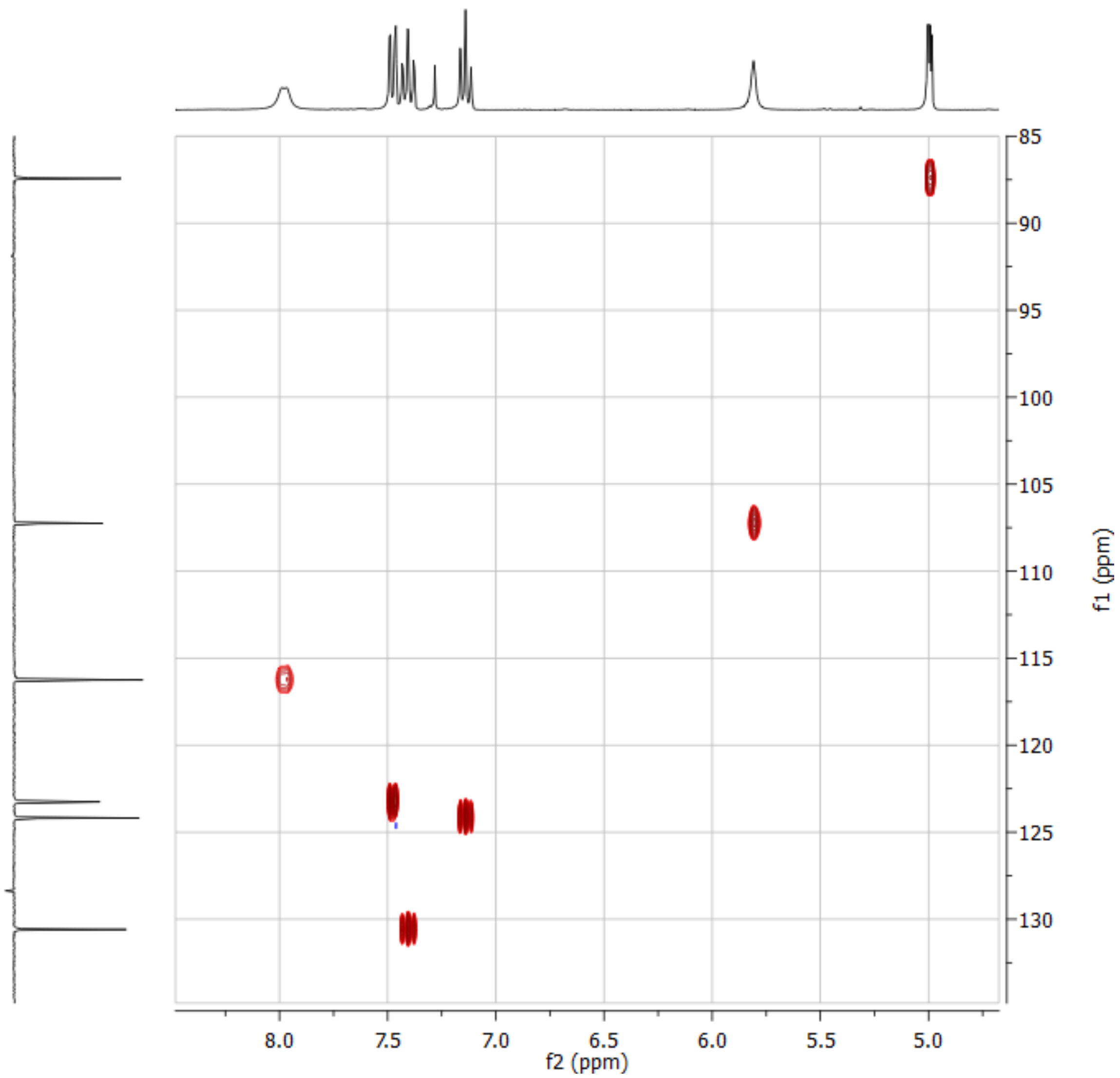

85
90
95
100
105
110
115
120
125
130
f1 (ppm)
8.0
7.5
7.0
6.5
6.0
5.5
5.0
f2 (ppm)

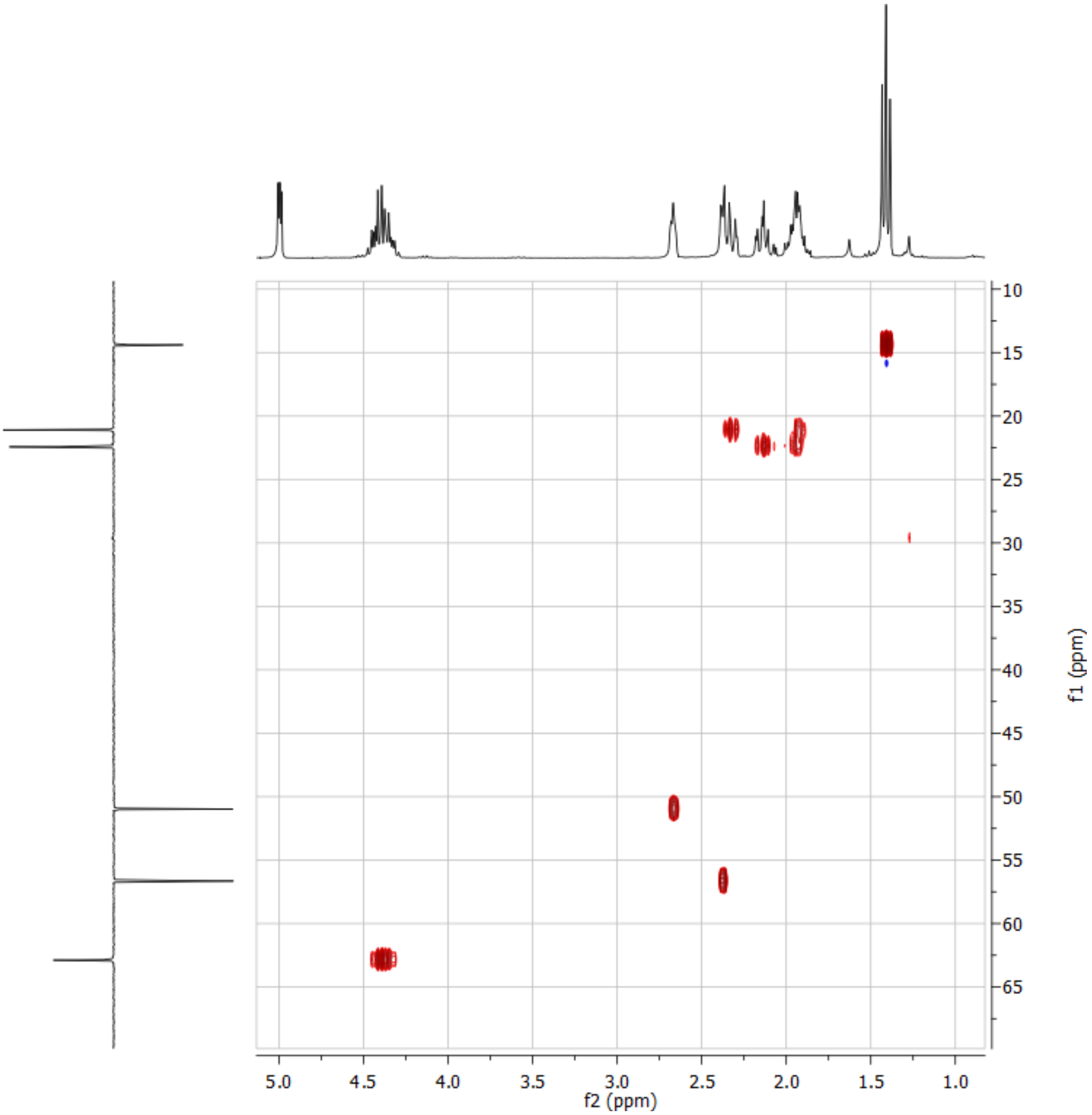

f1 (ppm)
f2 (ppm)
5.0
4.5
4.0
3.5
3.0
2.5
2.0
1.5
1.0
10
15
20
25
30
35
40
45
50
55
60
65

6a, NOESY in $CDCl_3$ at T = 300 K

The interaction between the two protons at 5.81 e 2.67 is also highlighted by the analysis of the structure obtained by X-rays (*vide infra*).

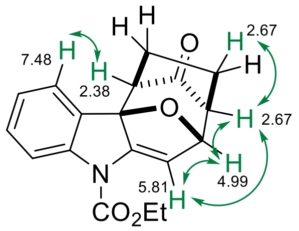

diagnostic cross picks

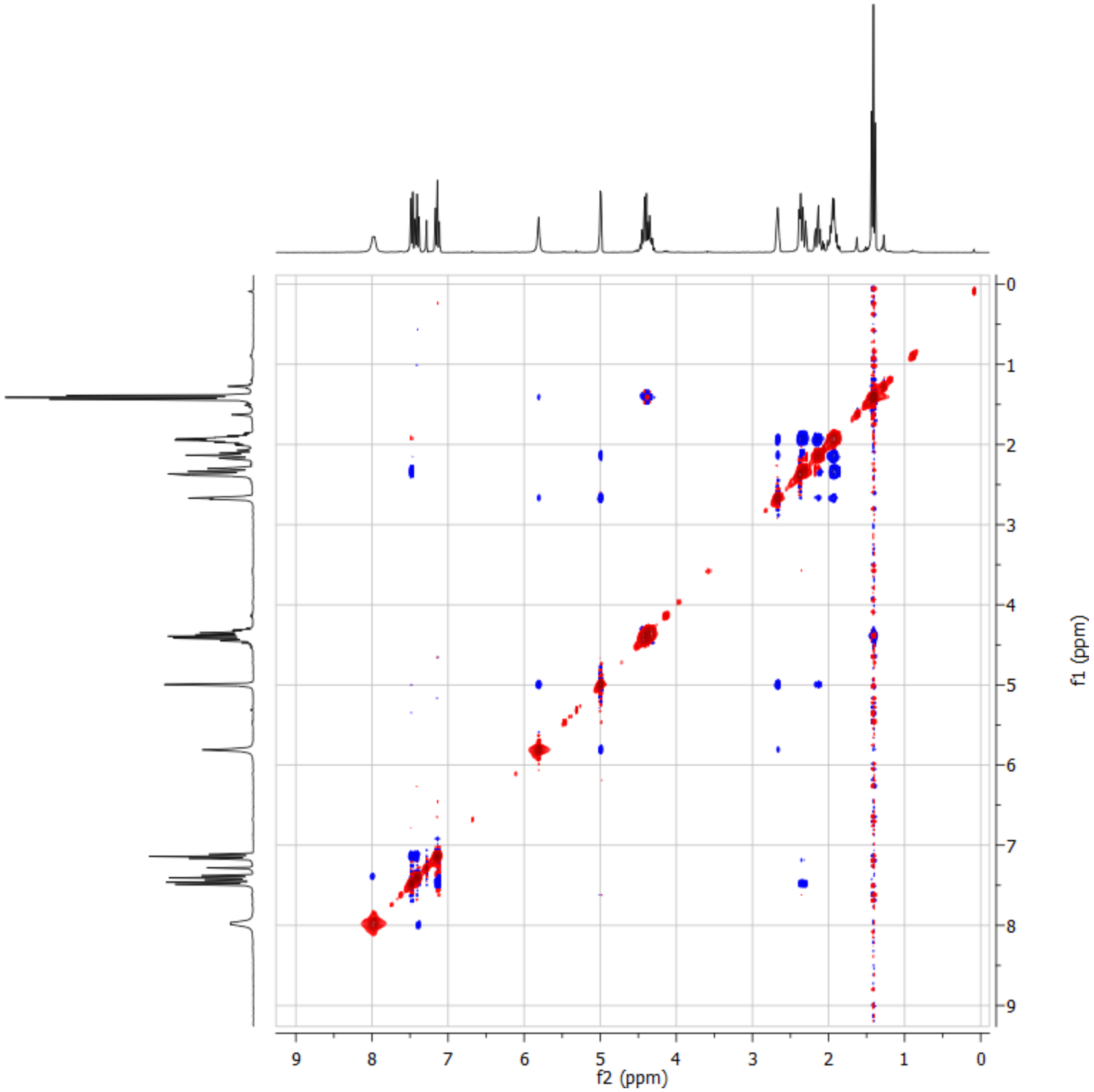

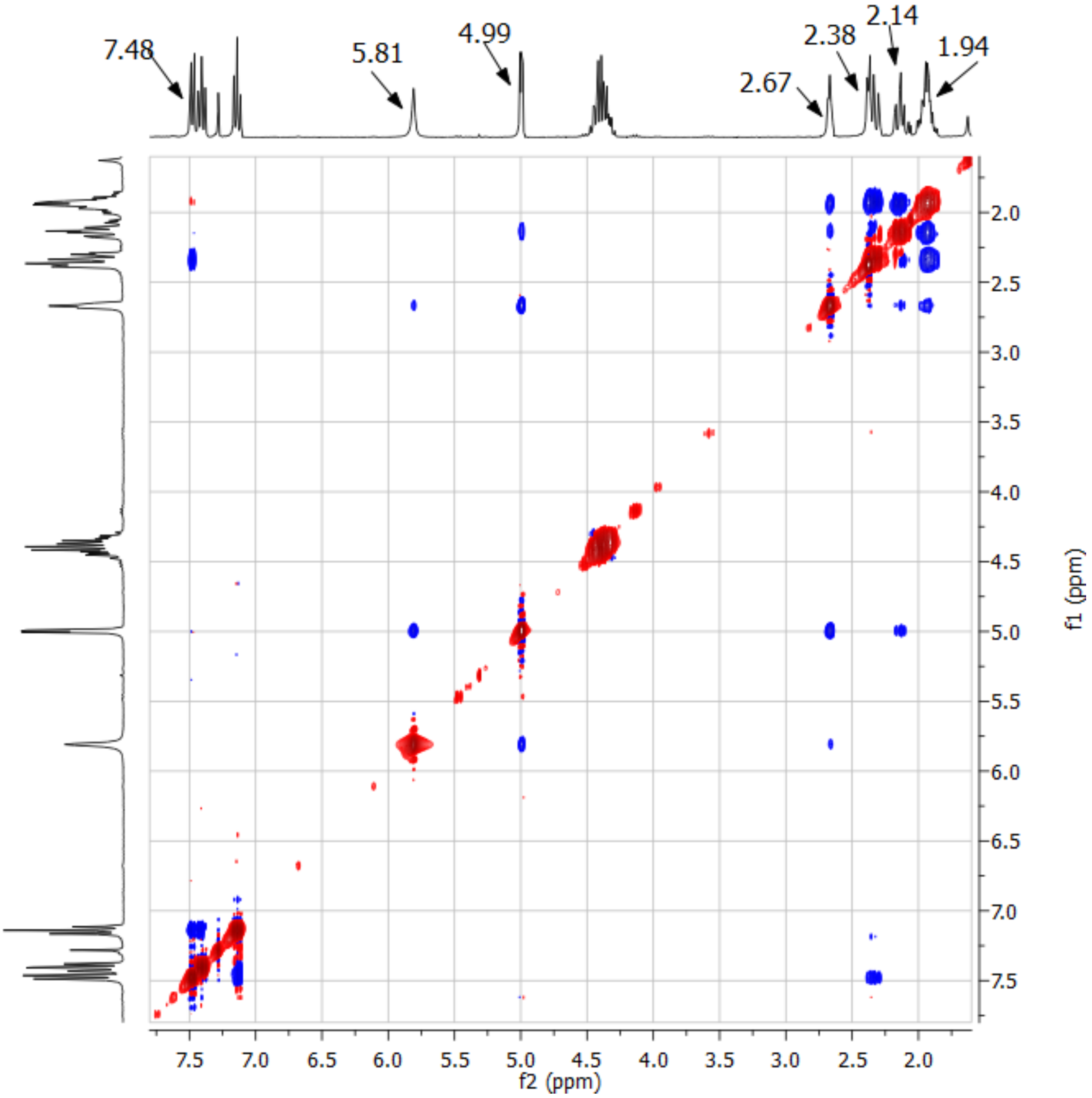

7.48
5.81
4.99
2.67
2.38
2.14
1.94
f2 (ppm)
f1 (ppm)

6a, HMBC in $CDCl_3$ at T = 300 K

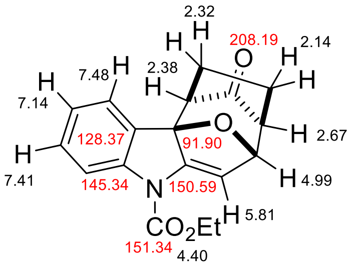

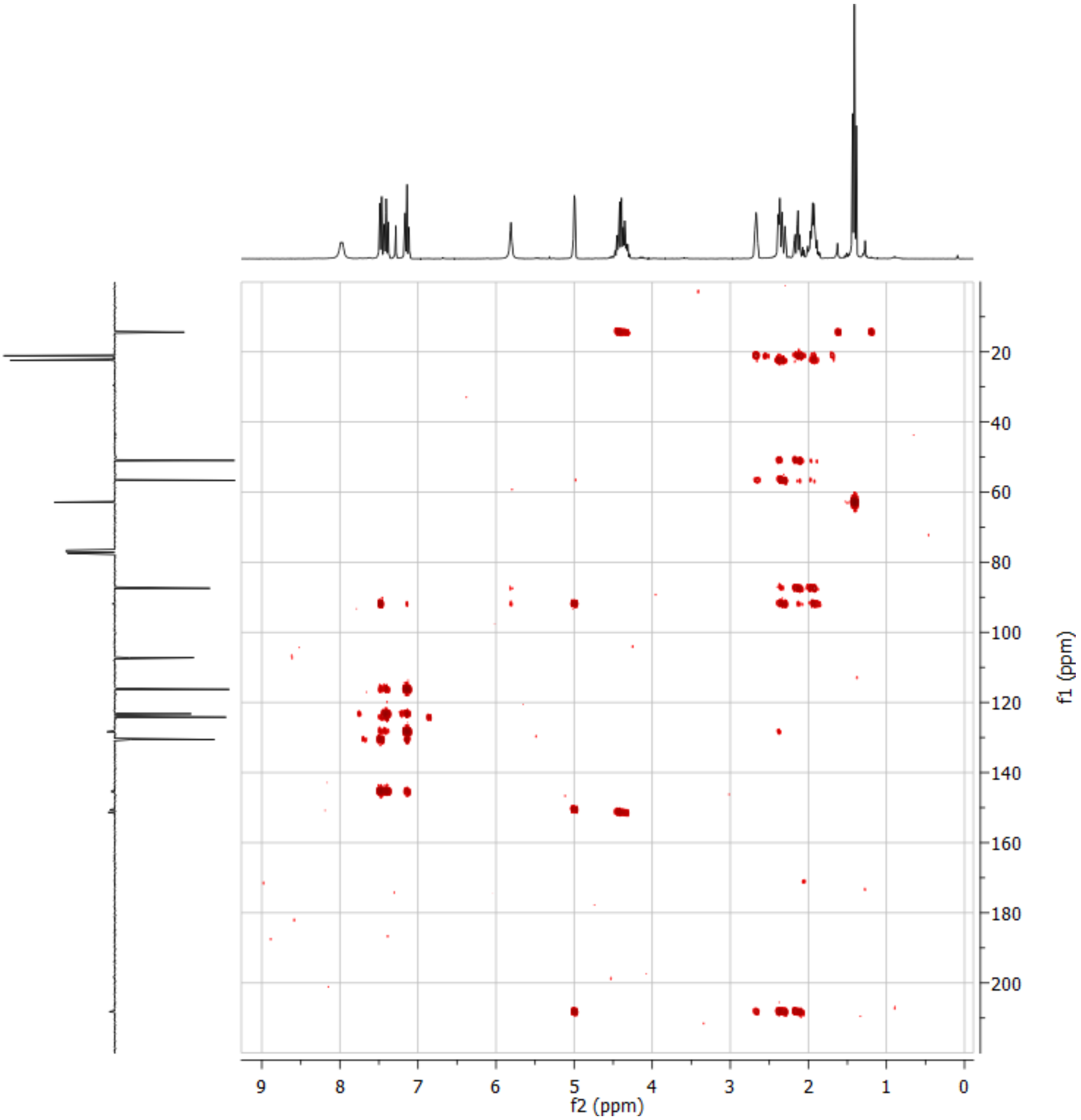

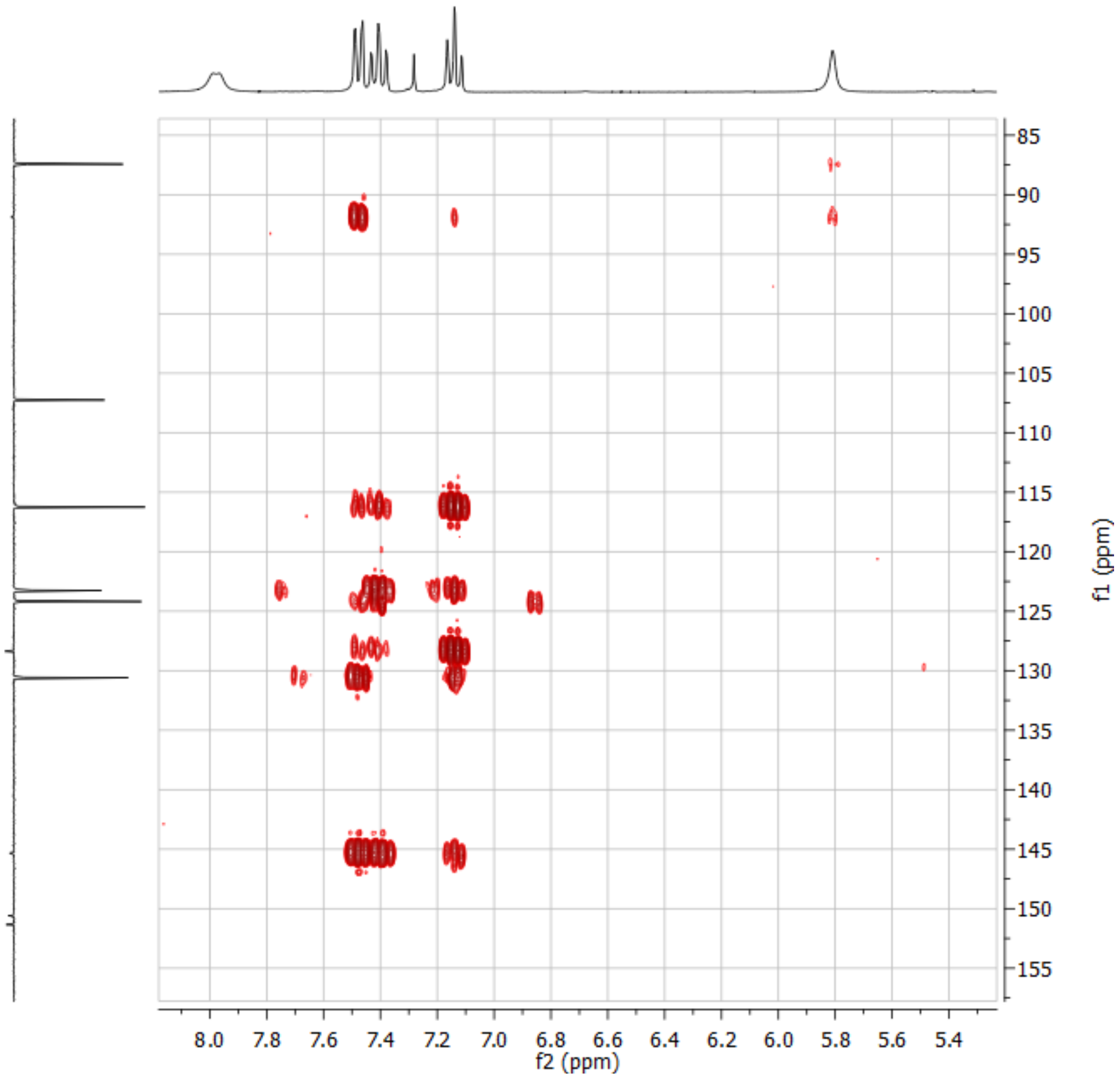

8.0
7.8
7.6
7.4
7.2
7.0
6.8
6.6
6.4
6.2
6.0
5.8
5.6
5.4
f2 (ppm)
85
90
95
100
105
110
115
120
125
130
135
140
145
150
155
f1 (ppm)

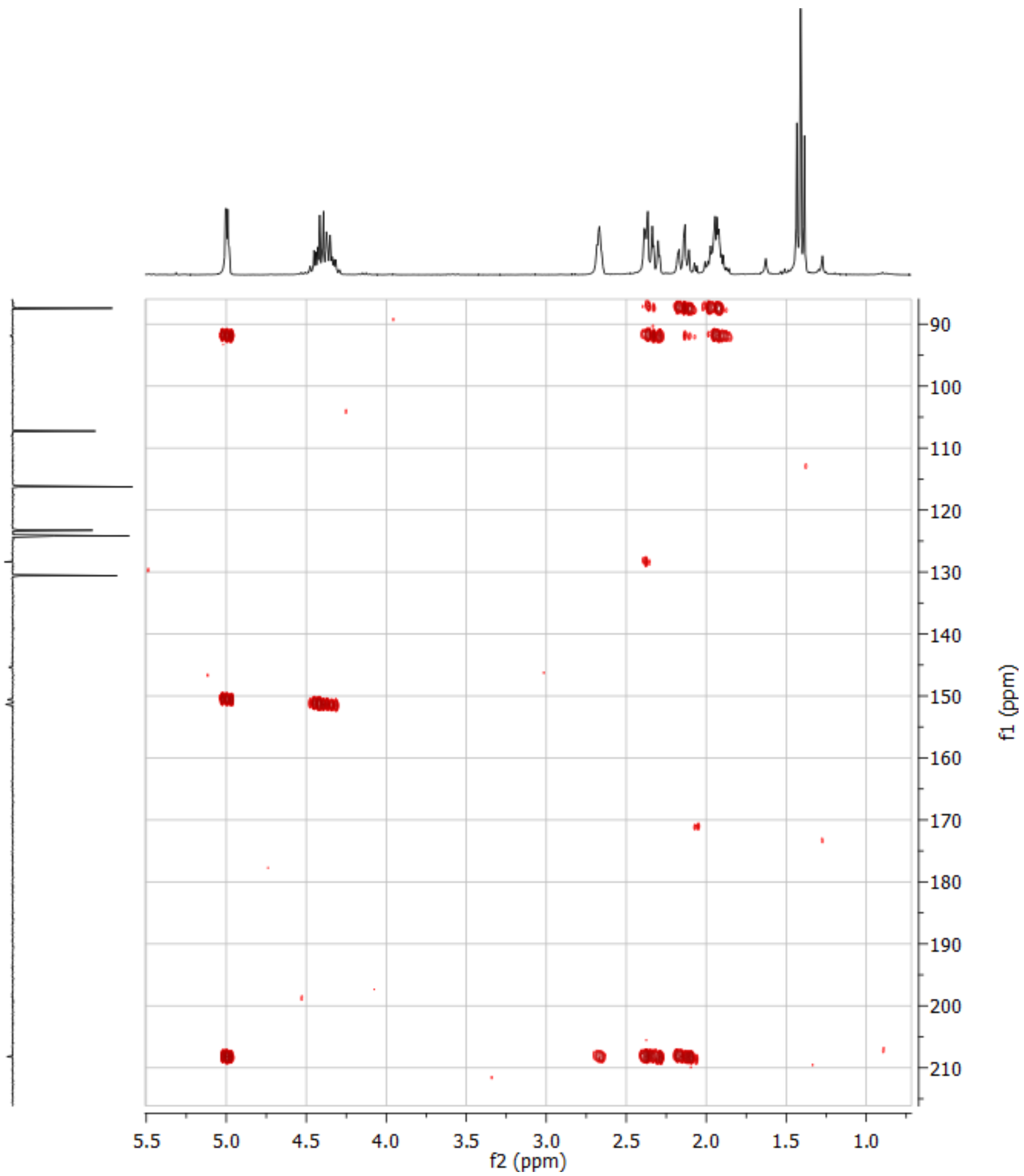

f2 (ppm)
f1 (ppm)

6f, COSY in $CDCl_3$ at T = 300 K

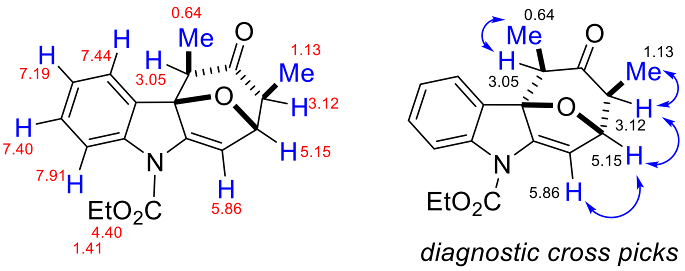

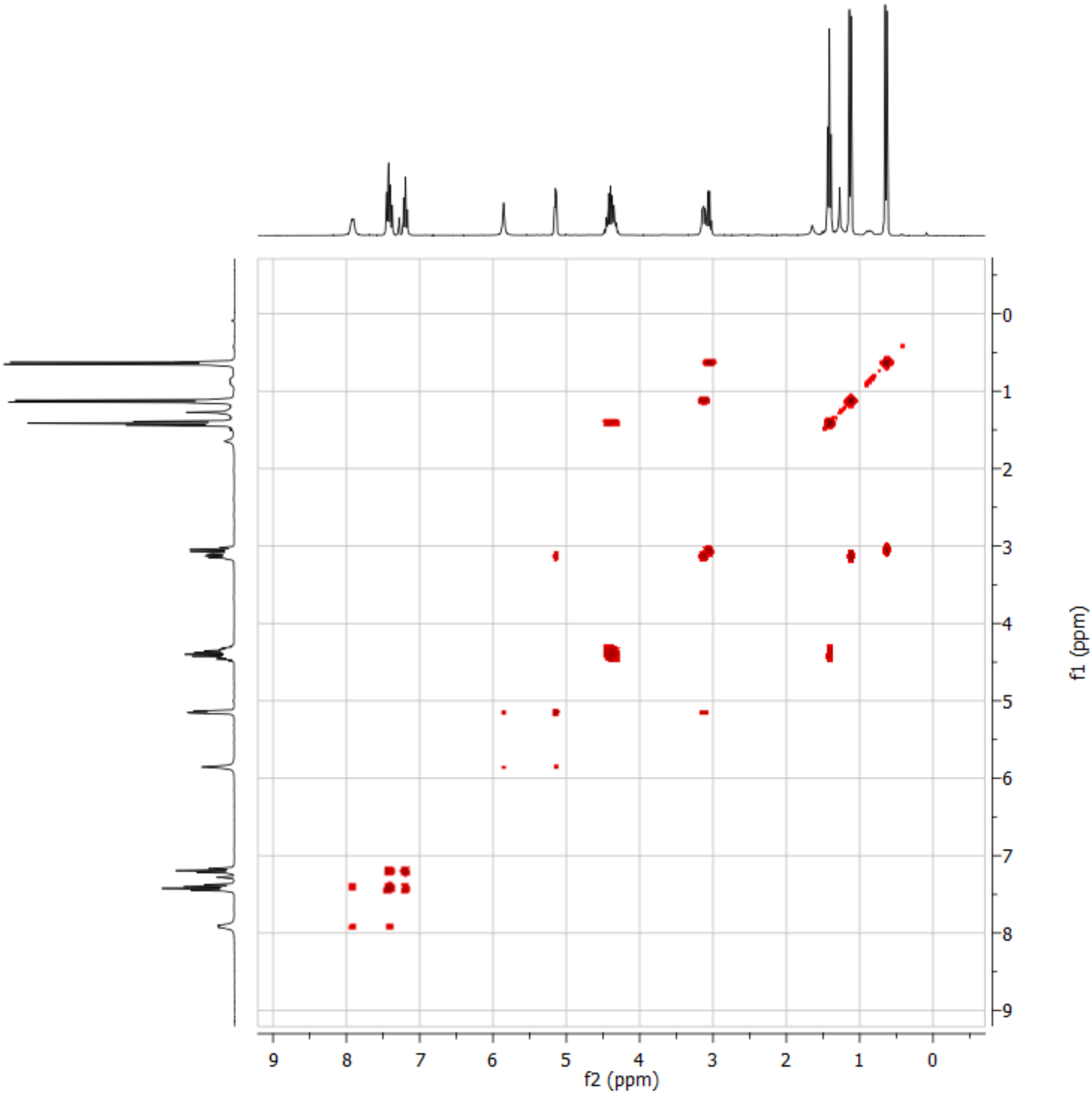

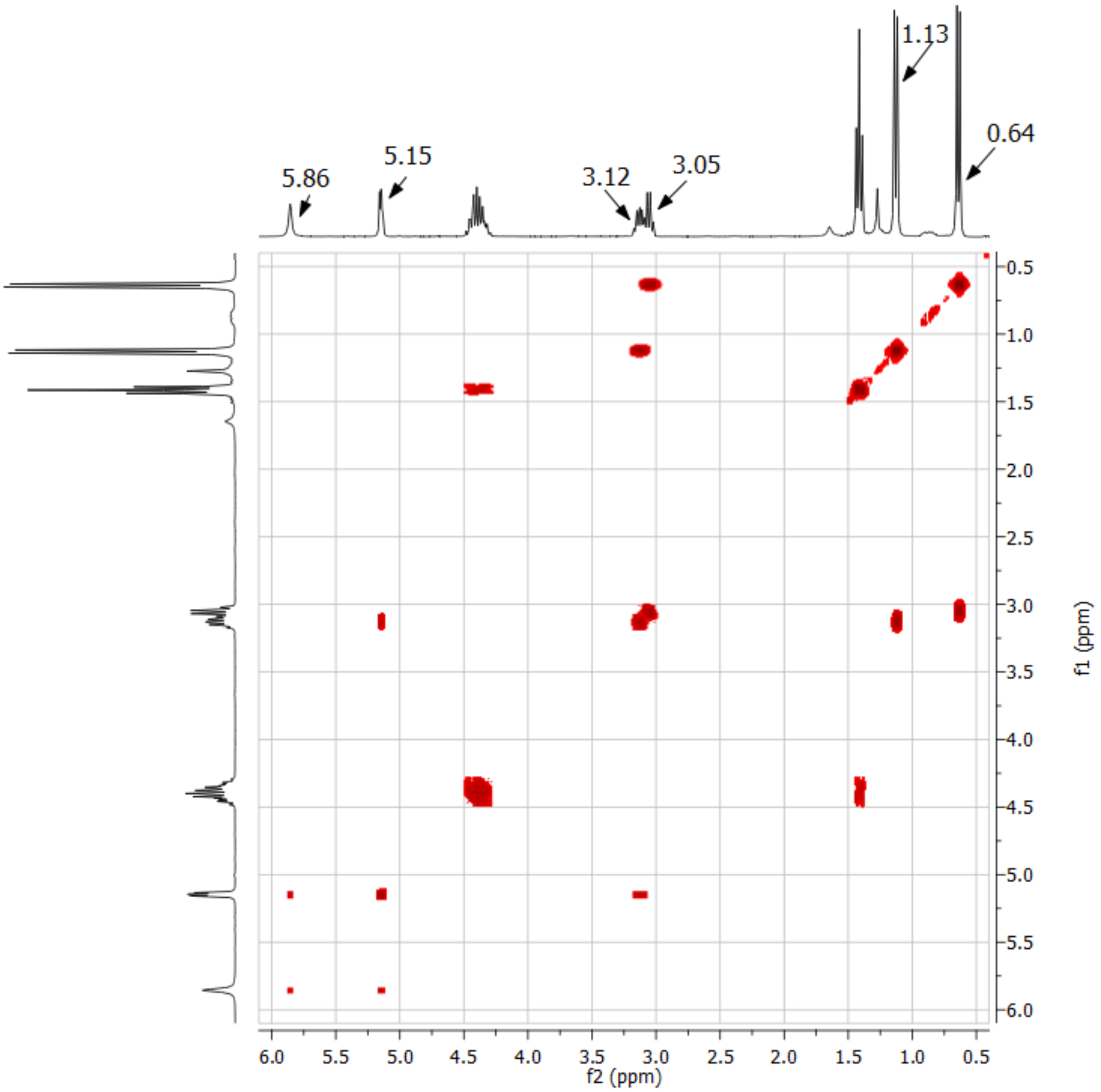
5.86
5.15
3.12
3.05
1.13
0.64
f1 (ppm)
f2 (ppm)

6f, HSQC in $CDCl_3$ at T = 300 K

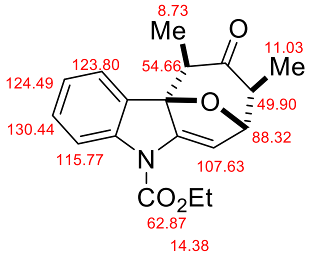

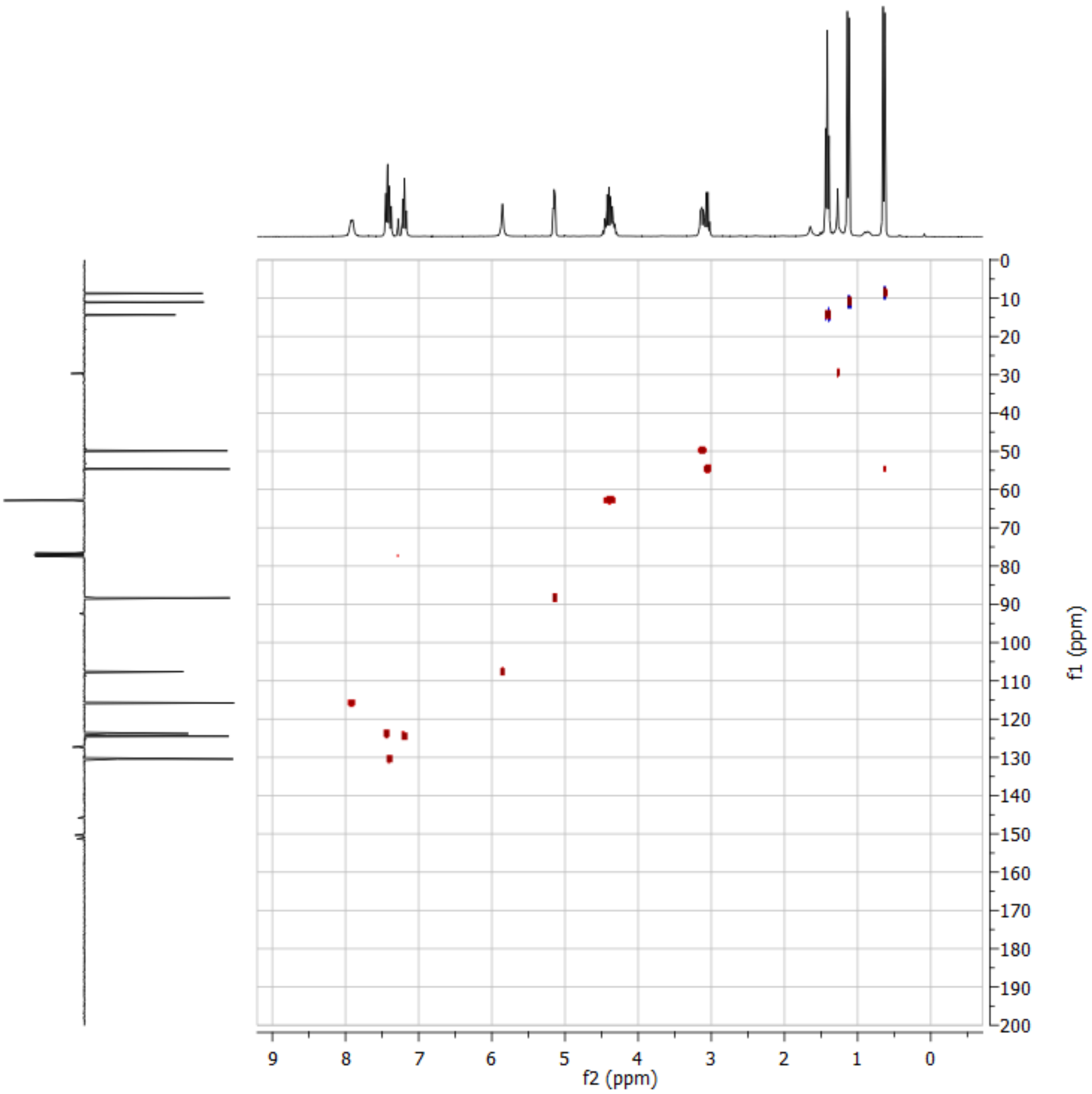

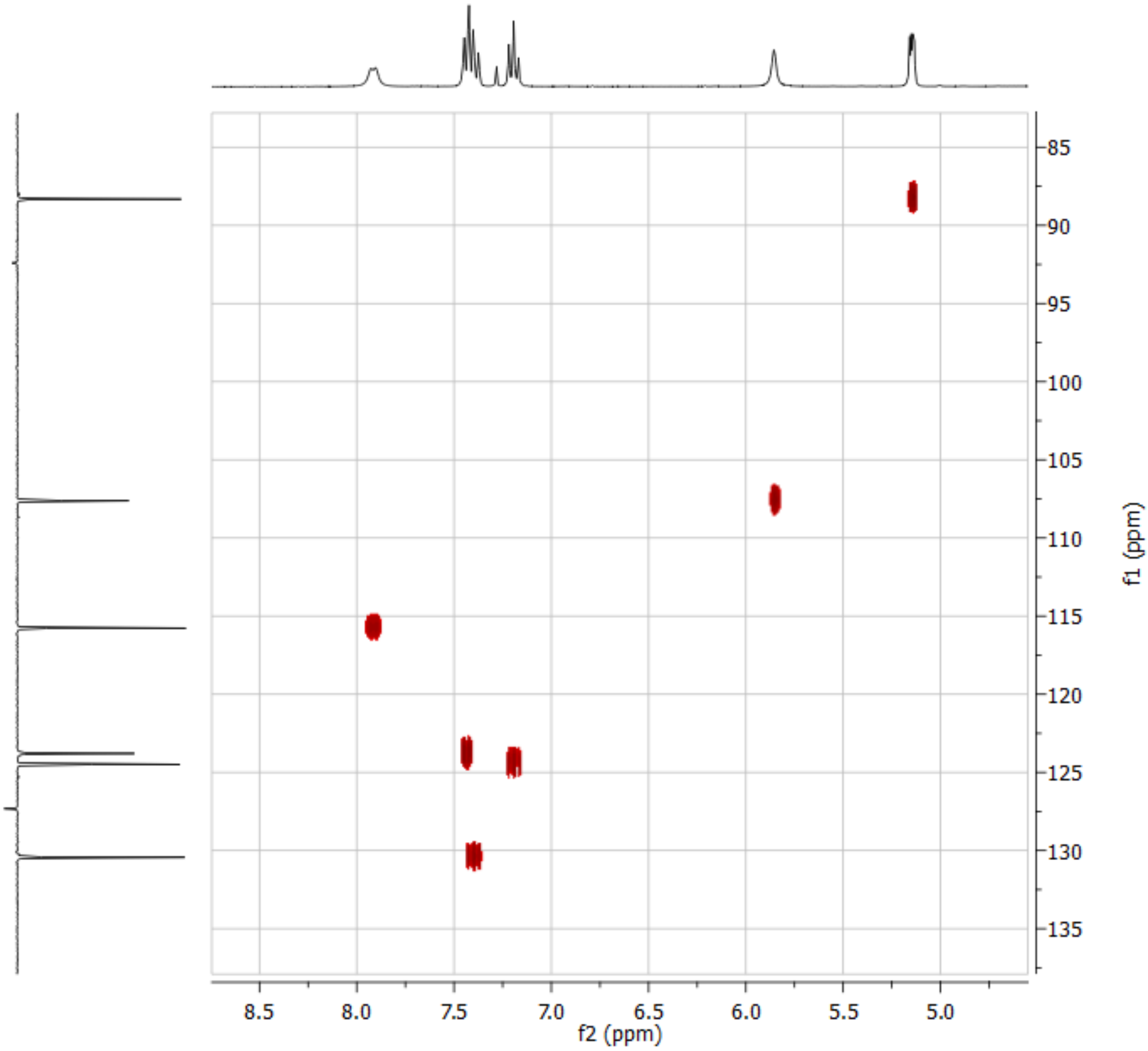

85
90
95
100
105
110
115
120
125
130
135
f1 (ppm)
8.5
8.0
7.5
7.0
6.5
6.0
5.5
5.0
f2 (ppm)

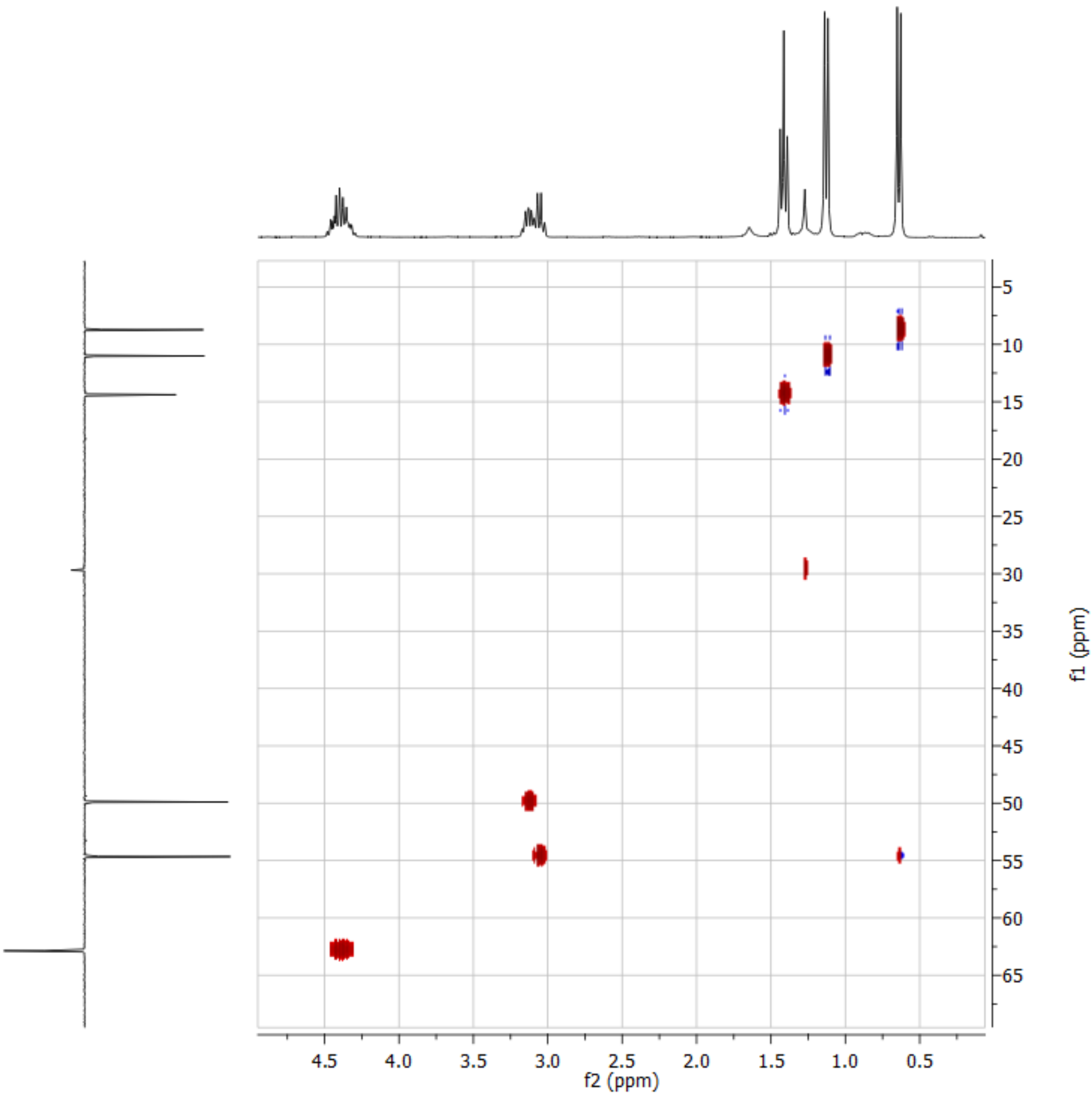

f1 (ppm)
f2 (ppm)

6f, NOESY in $CDCl_3$ at T = 300 K

For a better understanding of the diagnostic NOESY interactions, beside a picture reporting diagnostic cross picks with the use of arrows, we report a view of a MM2 minimized 3D model of **6f**.

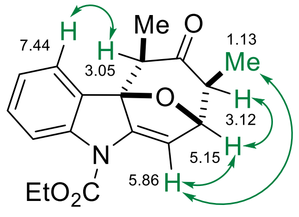

diagnostic cross picks

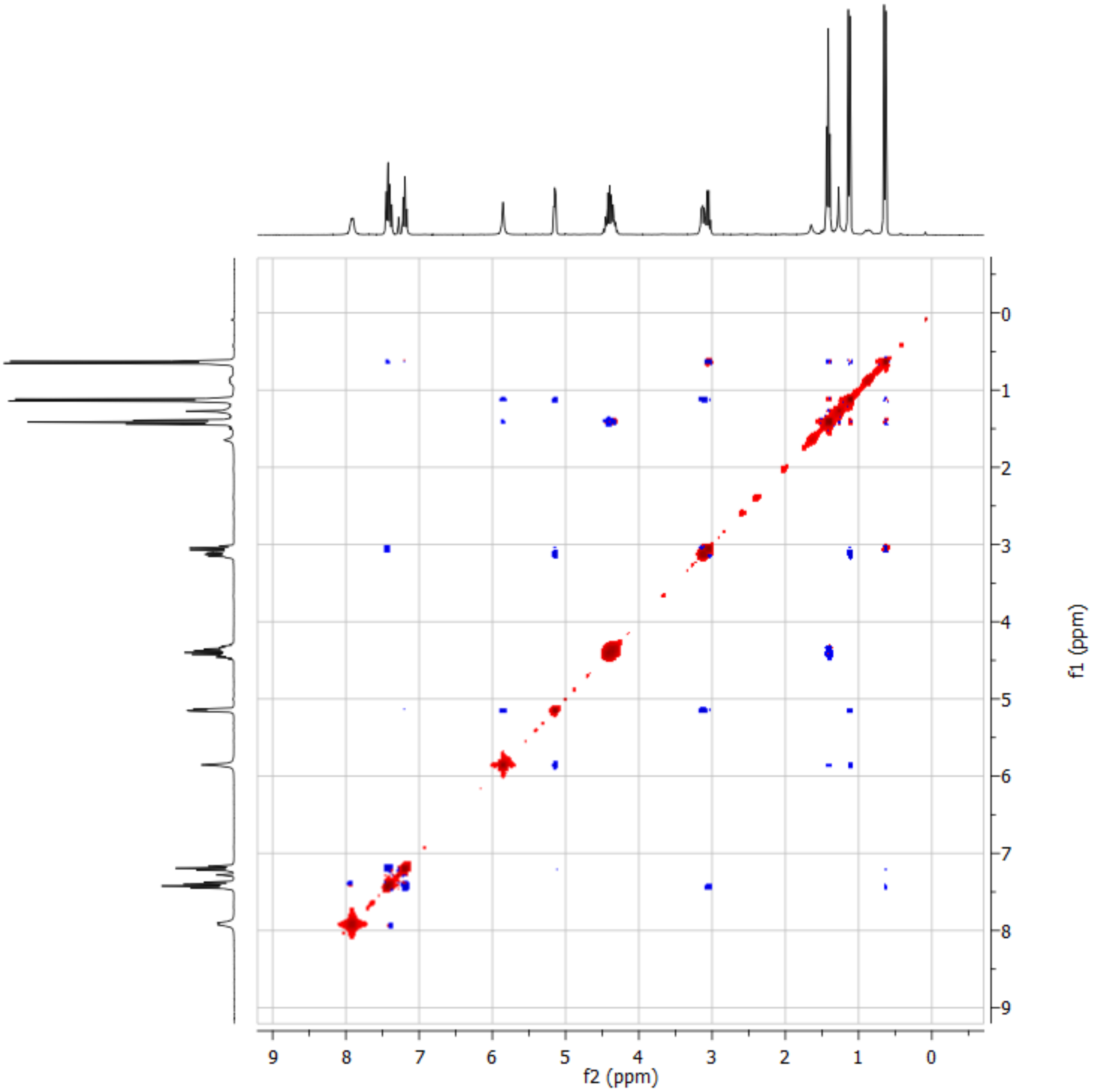

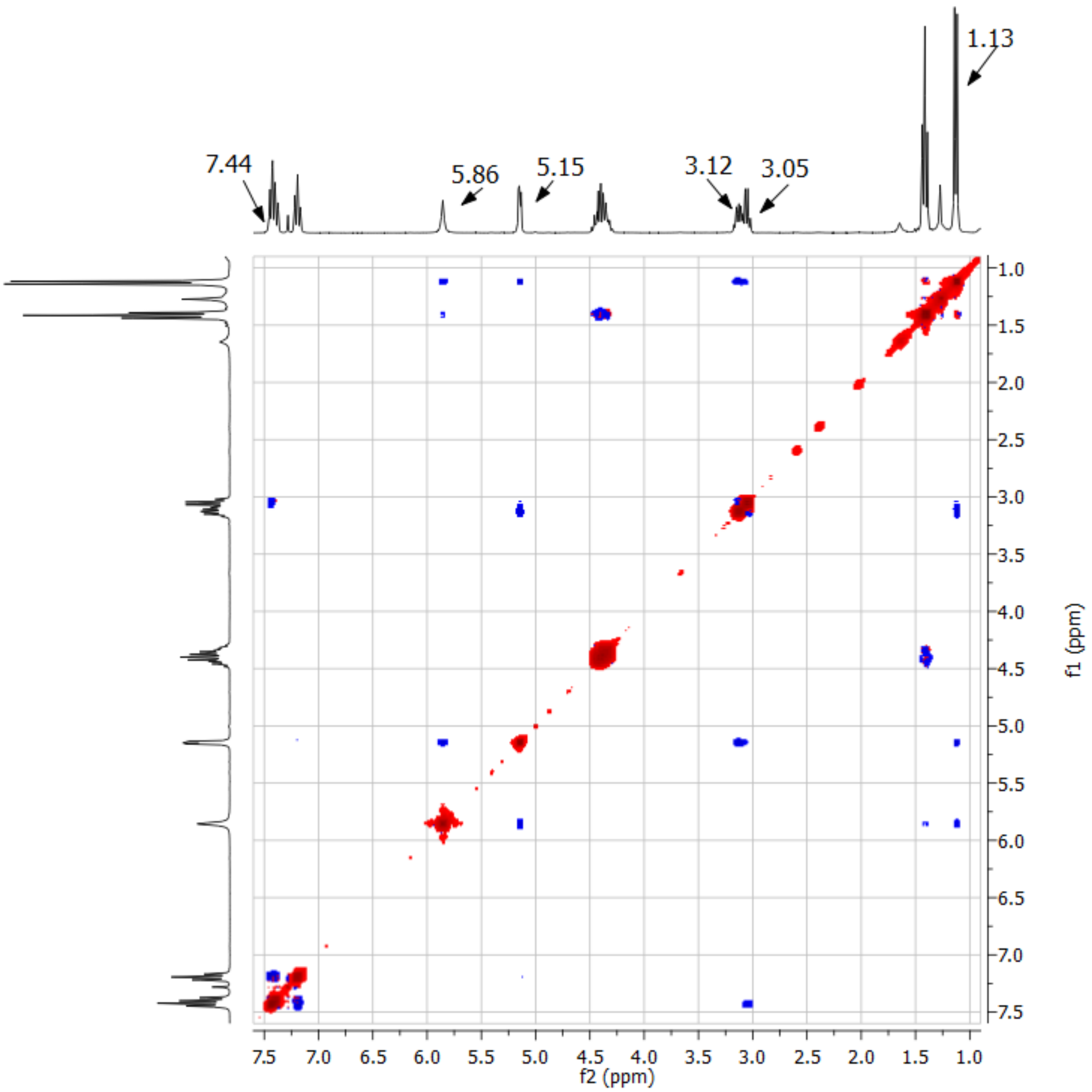
7.44
5.86
5.15
3.12
3.05
1.13
f2 (ppm)
f1 (ppm)

6f, MM2 minimized 3D structure

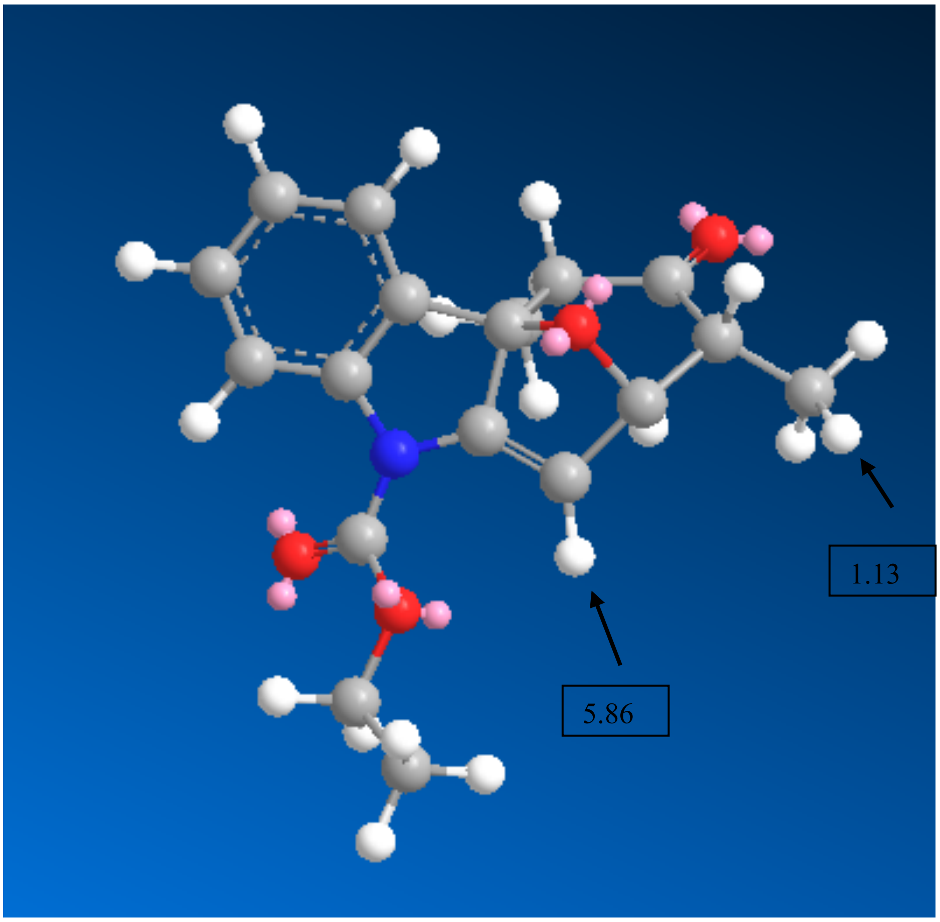

Figure S6: MM2 minimized 3D structure of **6f.**

6h, COSY in $CDCl_3$ at T = 300 K

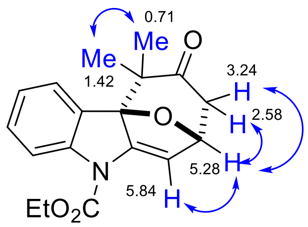

diagnostic cross picks

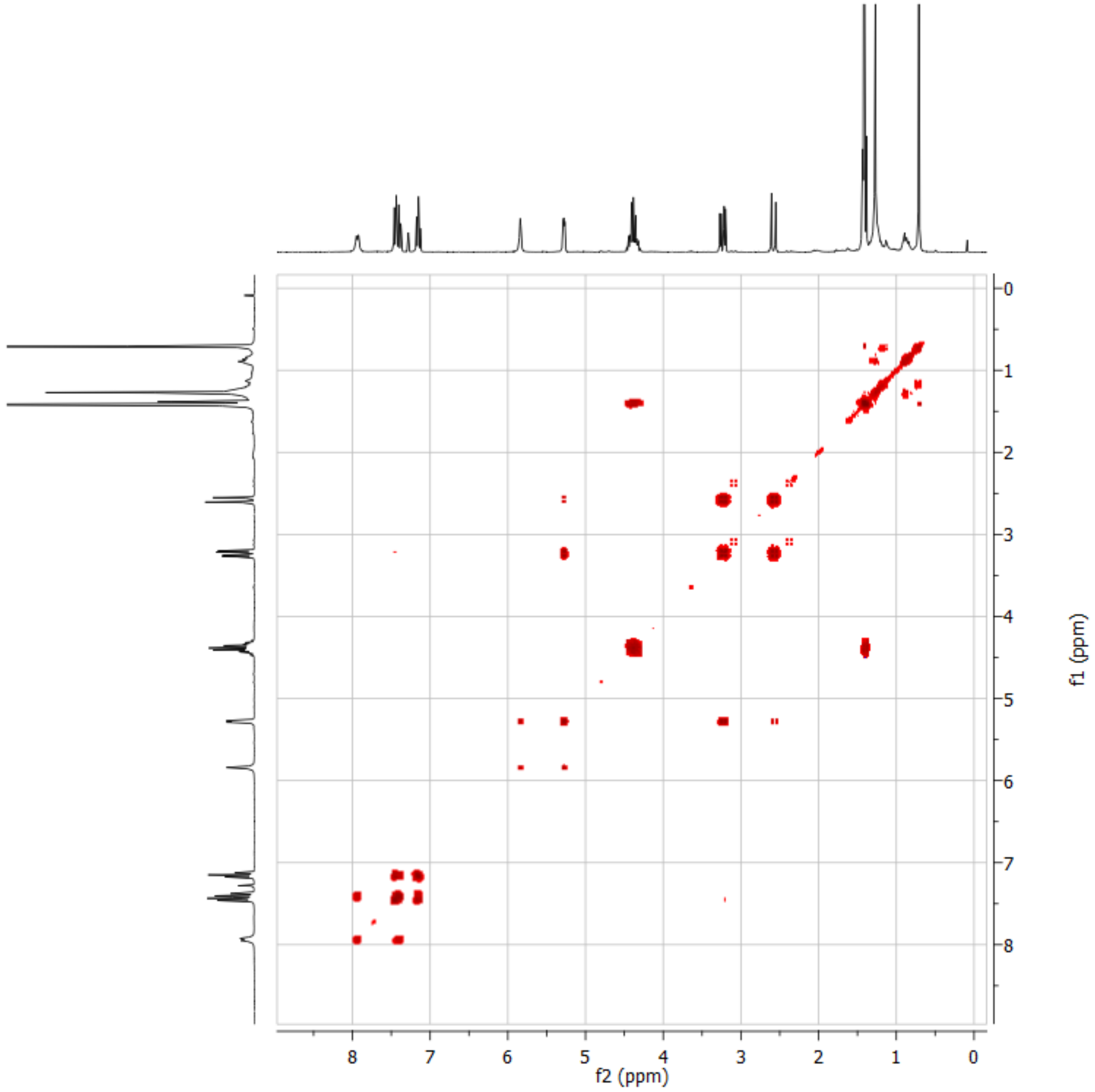

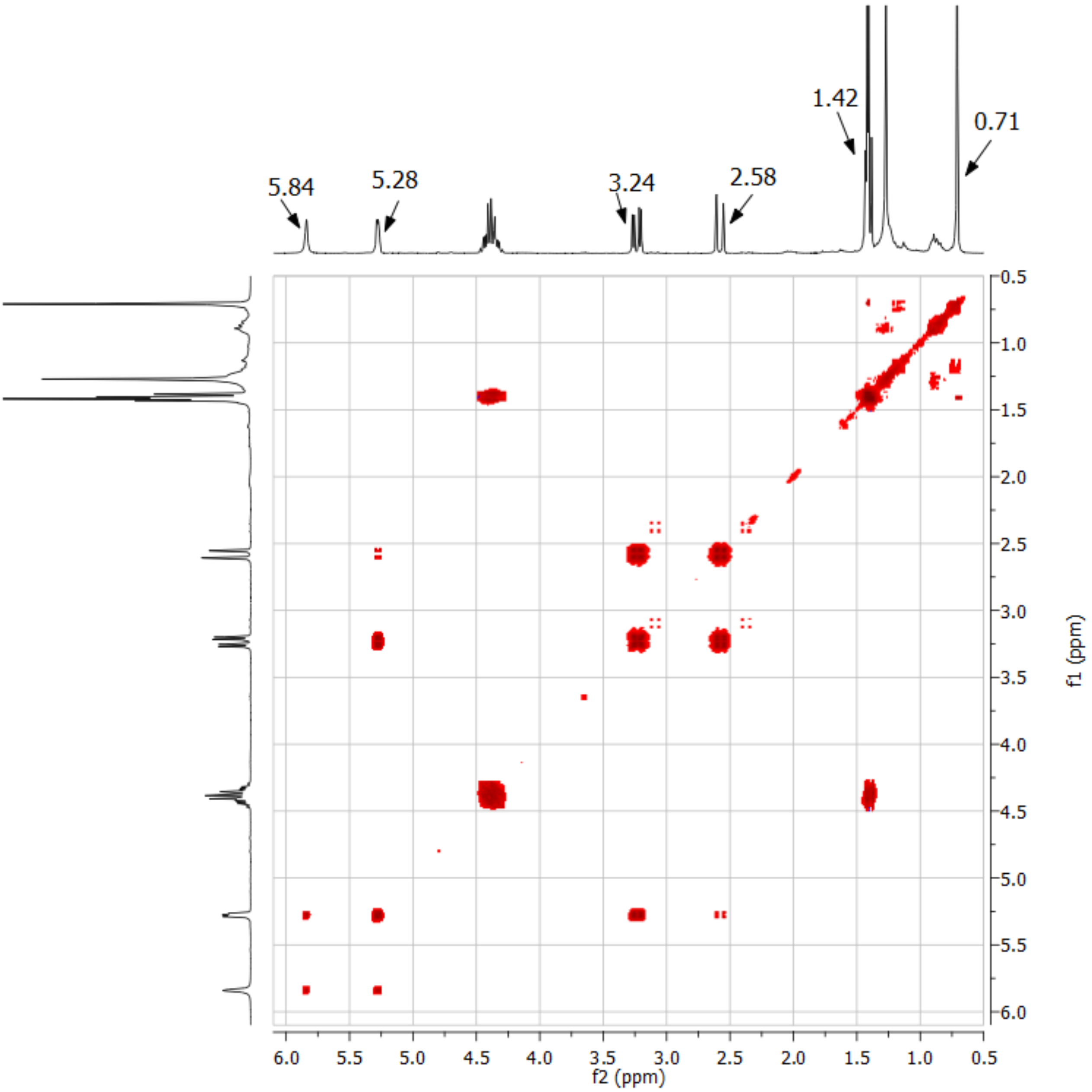

5.84
5.28
3.24
2.58
1.42
0.71
f2 (ppm)
f1 (ppm)

8, COSY in C_6D_6 at T = 300 K

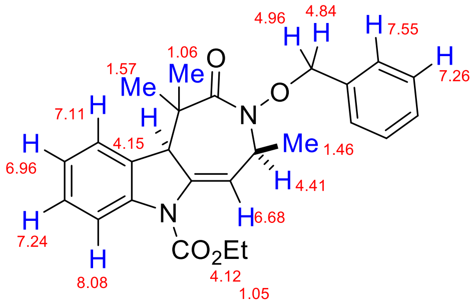

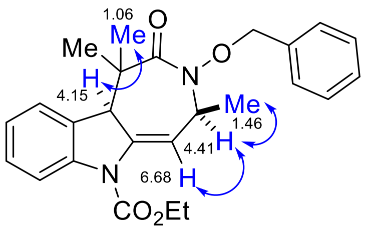

diagnostic cross picks

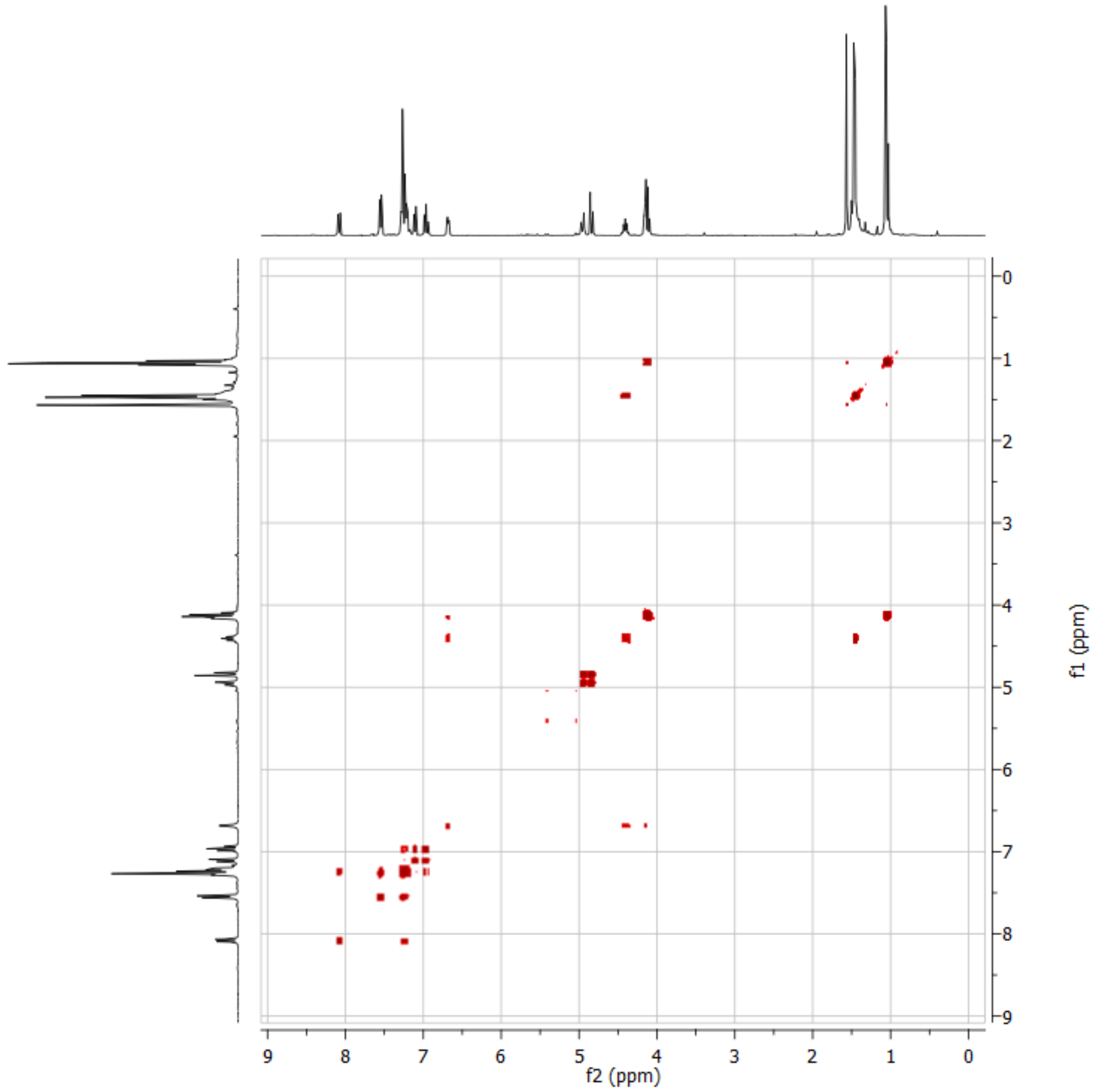

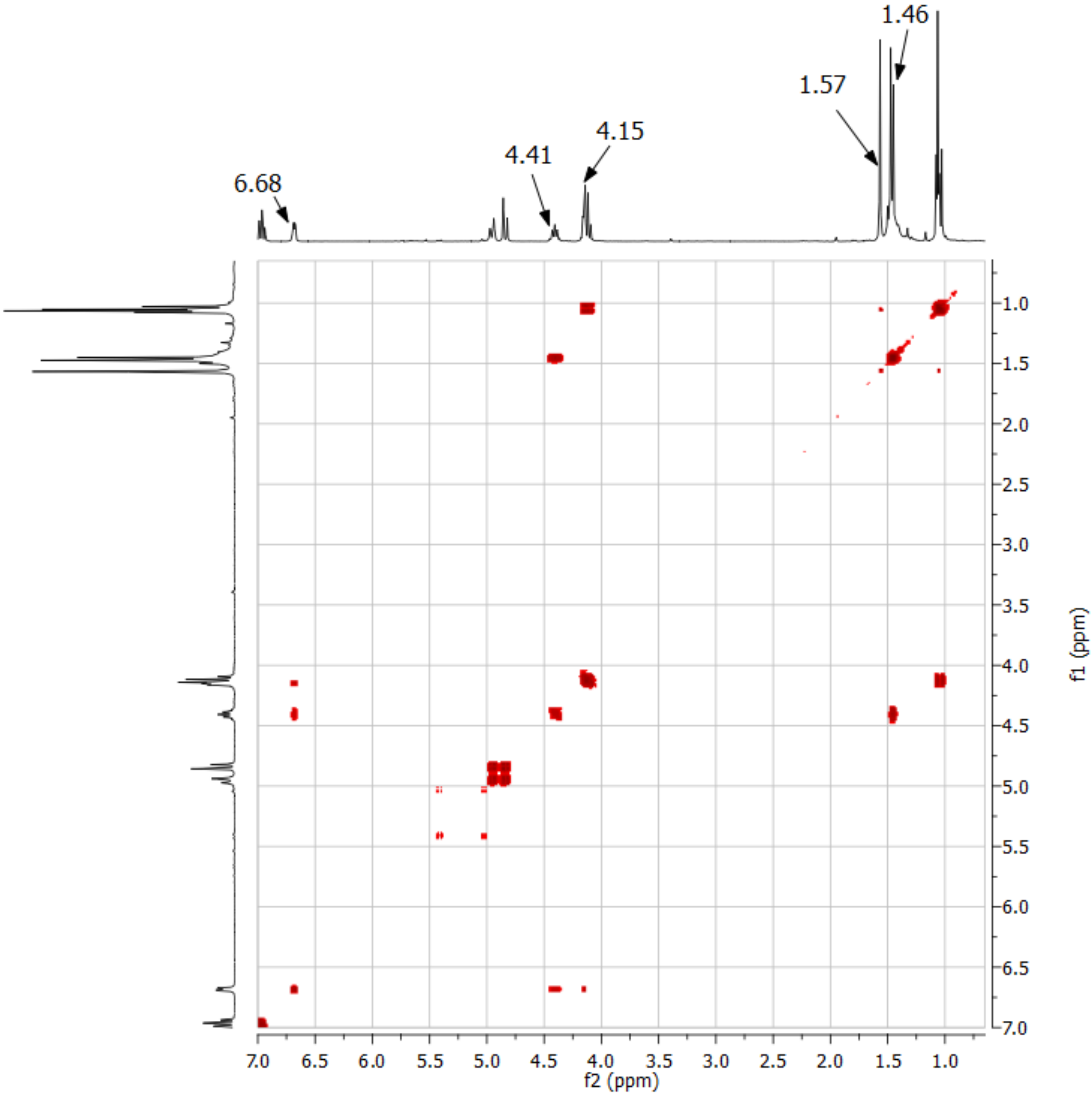

6.68
4.41
4.15
1.57
1.46
f2 (ppm)
f1 (ppm)

8, HSQC in C_6D_6 at T = 300 K

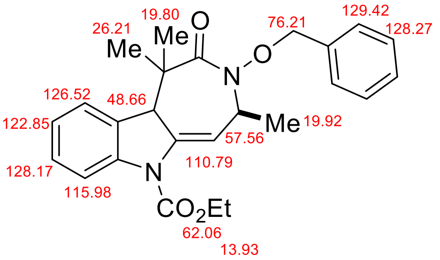

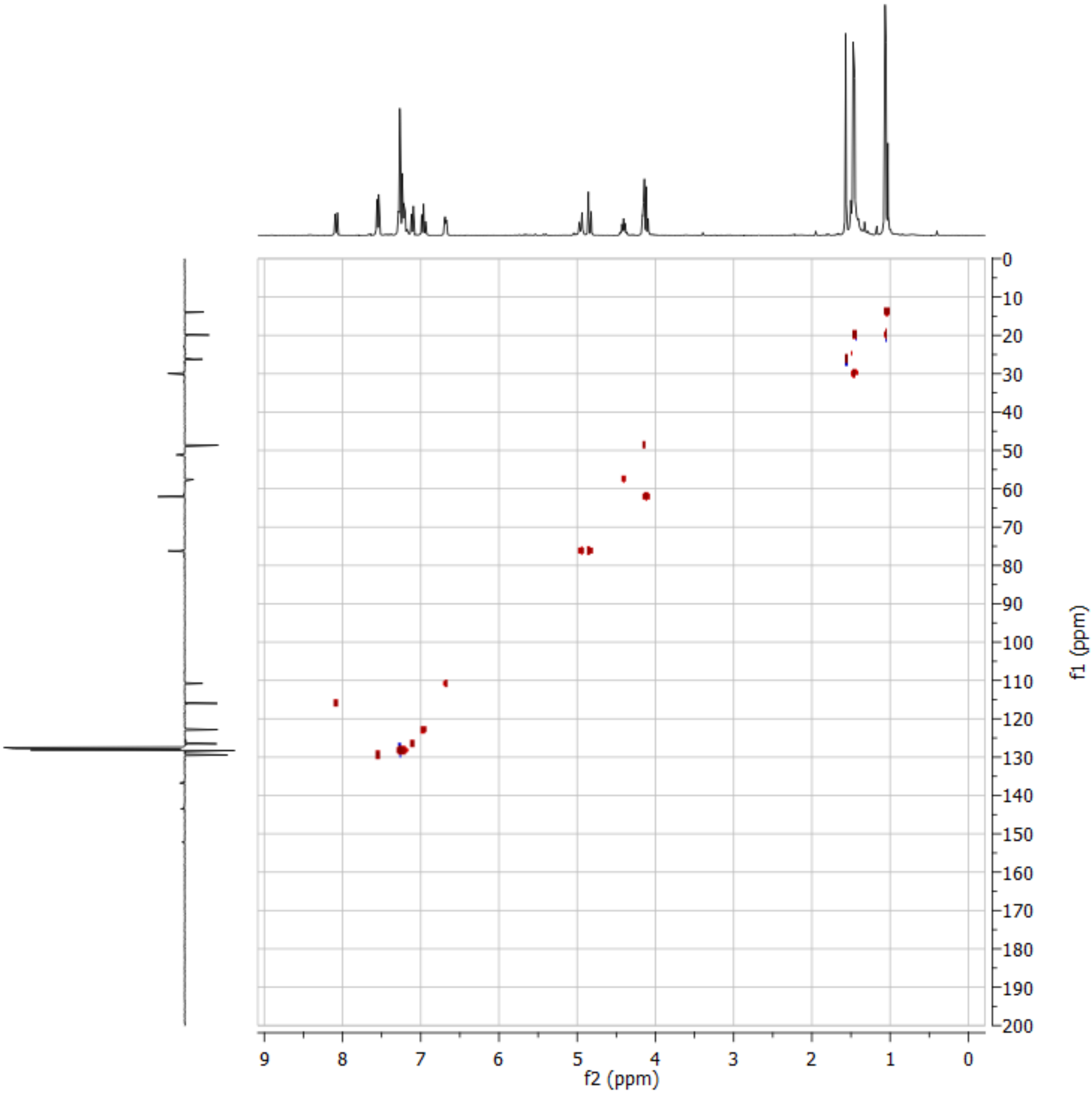

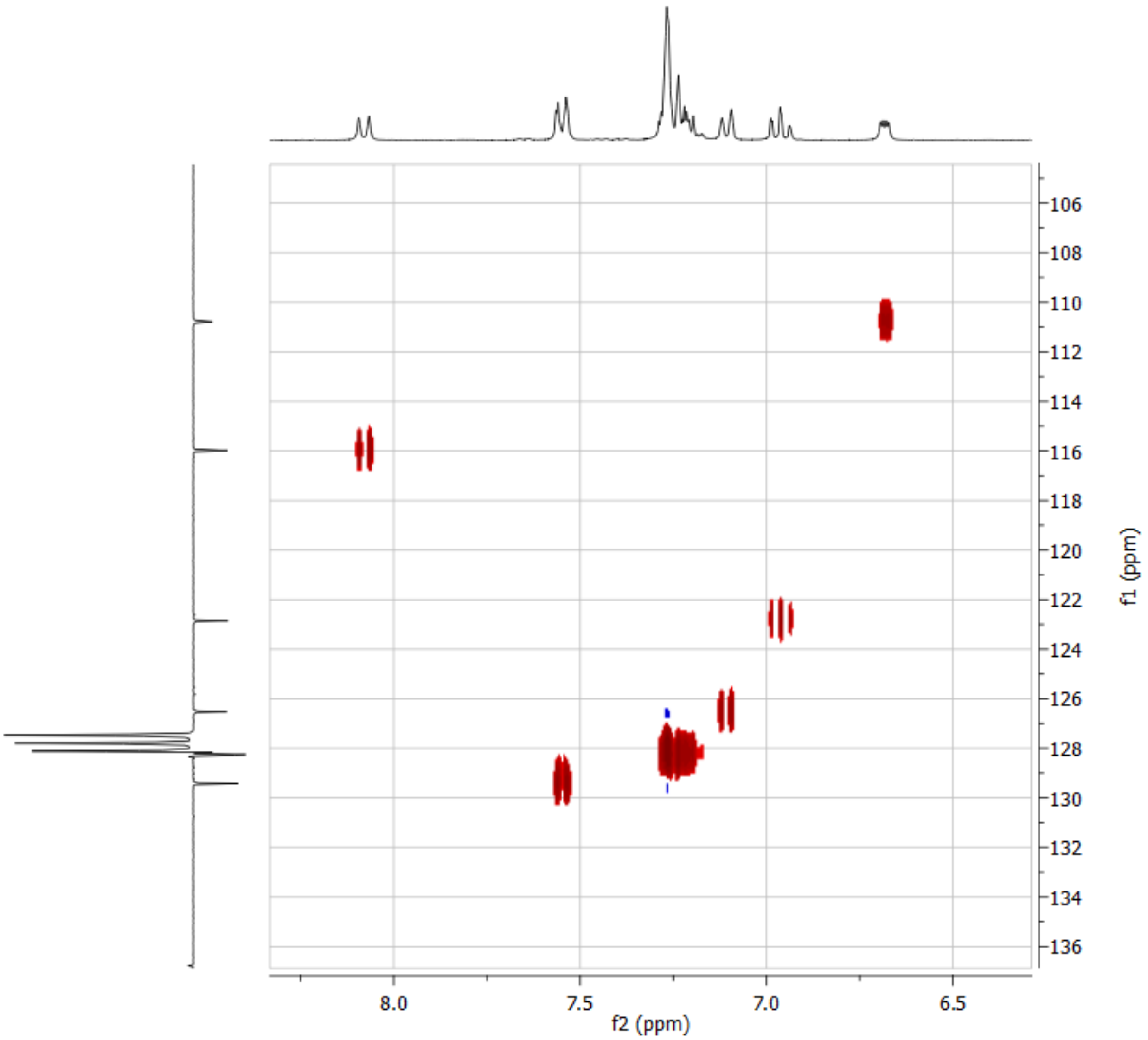
106
108
110
112
114
116
118
120
122
124
126
128
130
132
134
136
f1 (ppm)
8.0
7.5
7.0
6.5
f2 (ppm)

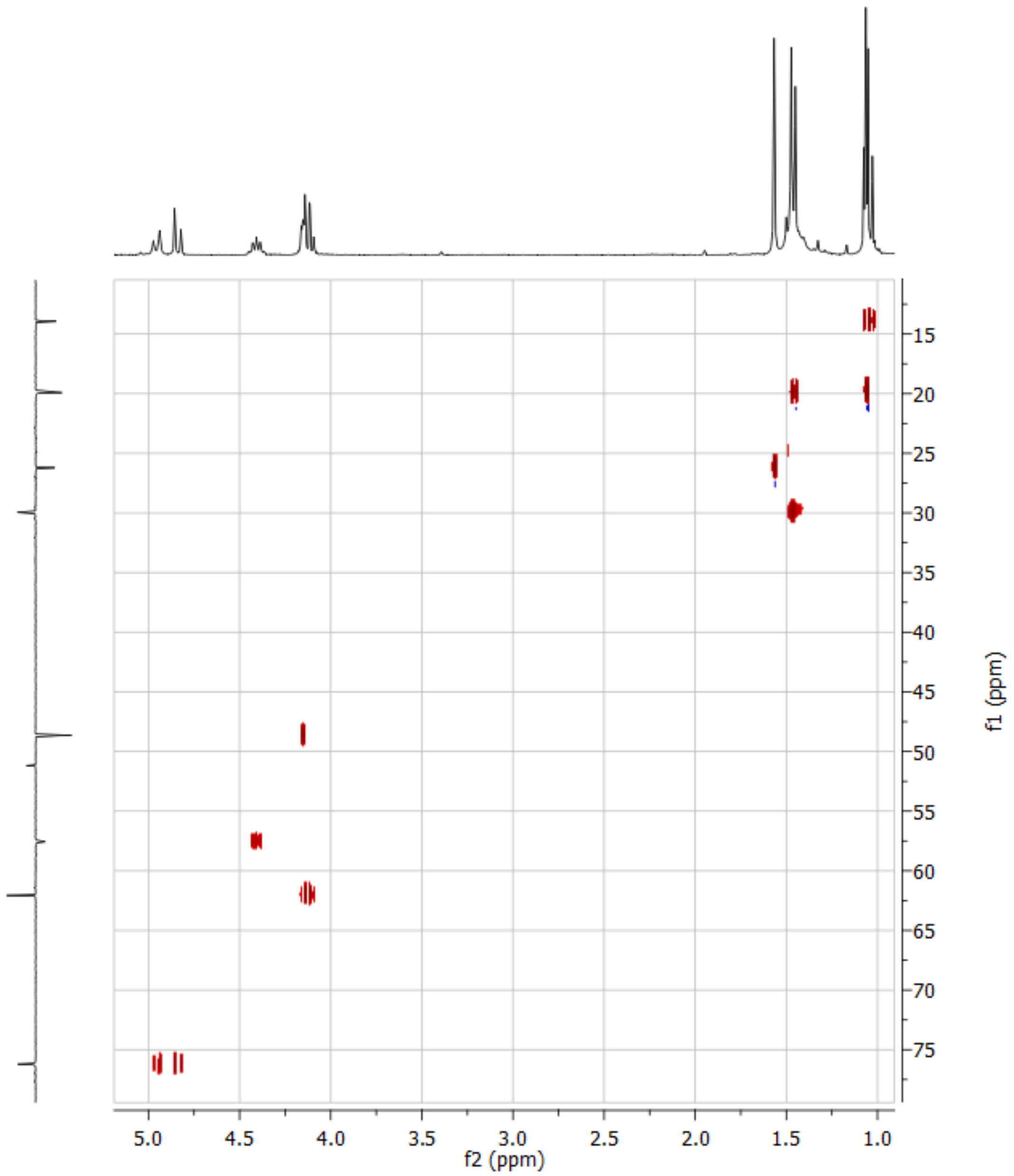

5.0
4.5
4.0
3.5
3.0
2.5
2.0
1.5
1.0
f2 (ppm)
15
20
25
30
35
40
45
50
55
60
65
70
75
f1 (ppm)

8, NOESY in C_6D_6 at T = 300 K

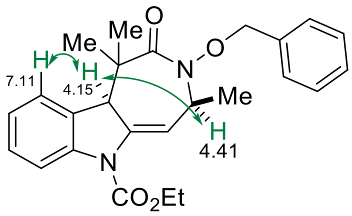

diagnostic cross picks

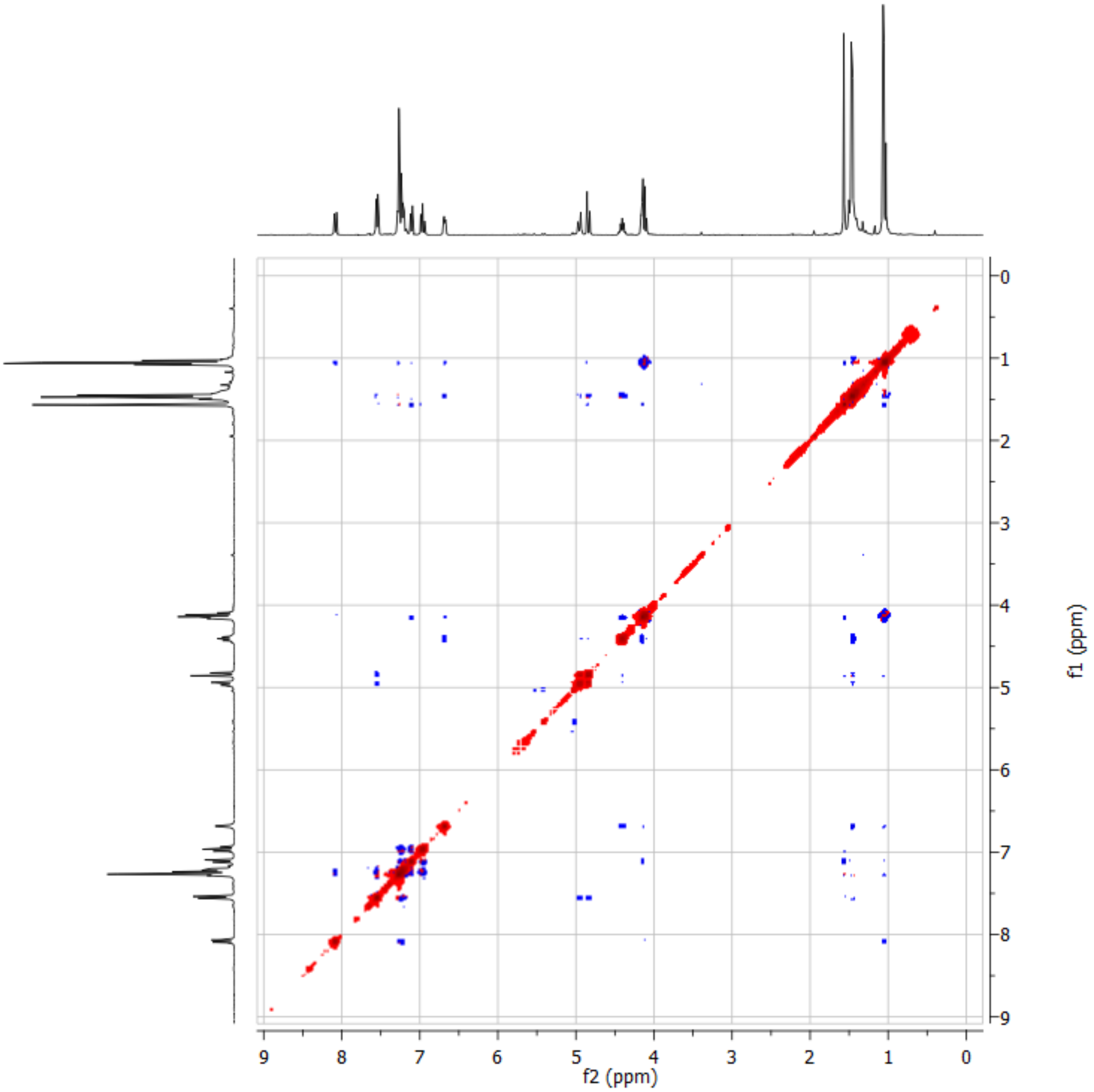

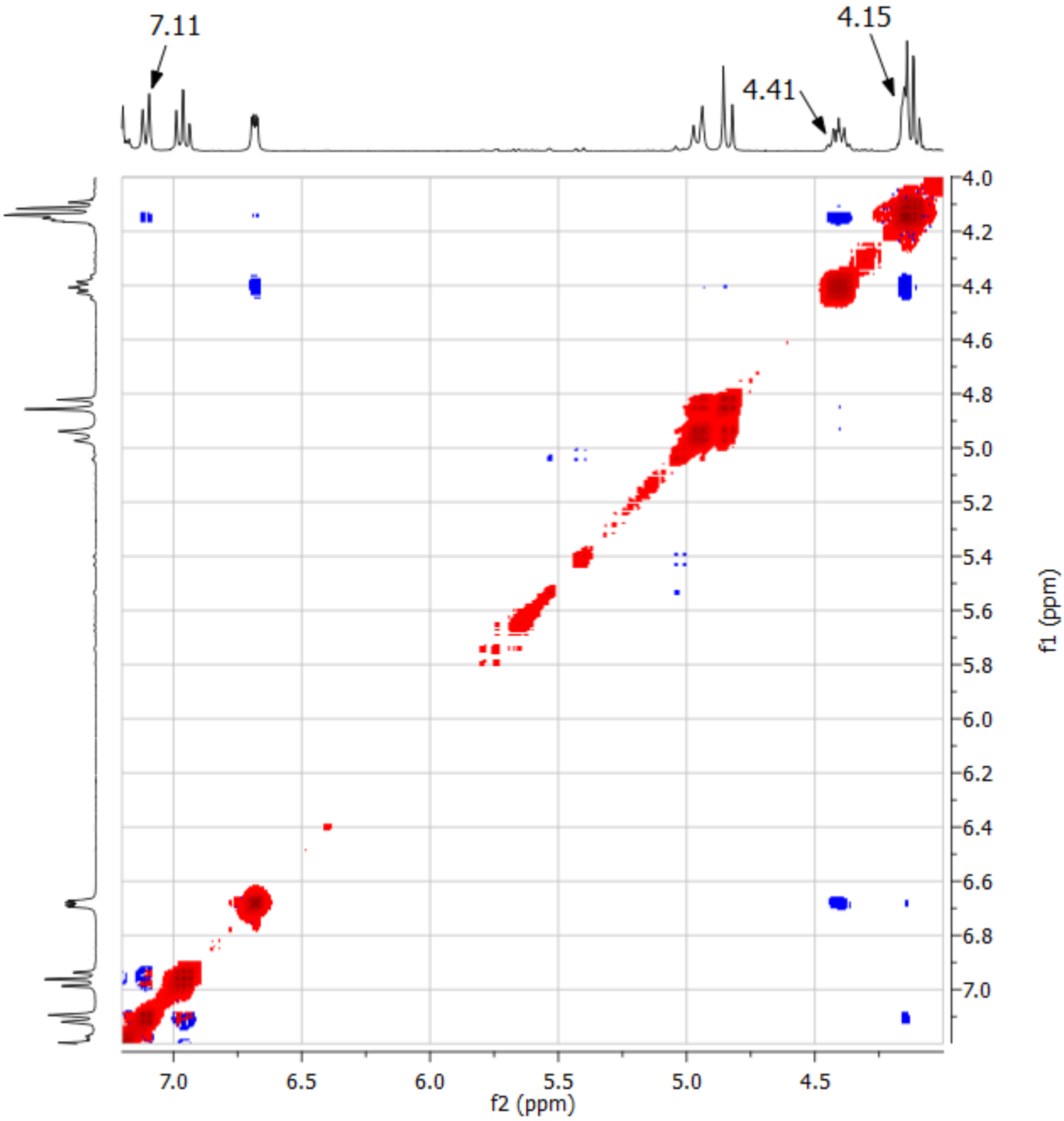

7.11
4.41
4.15
4.0
4.2
4.4
4.6
4.8
5.0
5.2
5.4
5.6
5.8
6.0
6.2
6.4
6.6
6.8
7.0
f1 (ppm)
7.0
6.5
6.0
5.5
5.0
4.5
f2 (ppm)

8, HMBC in C_6D_6 at T = 300 K

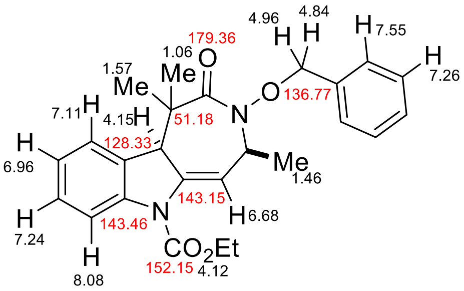

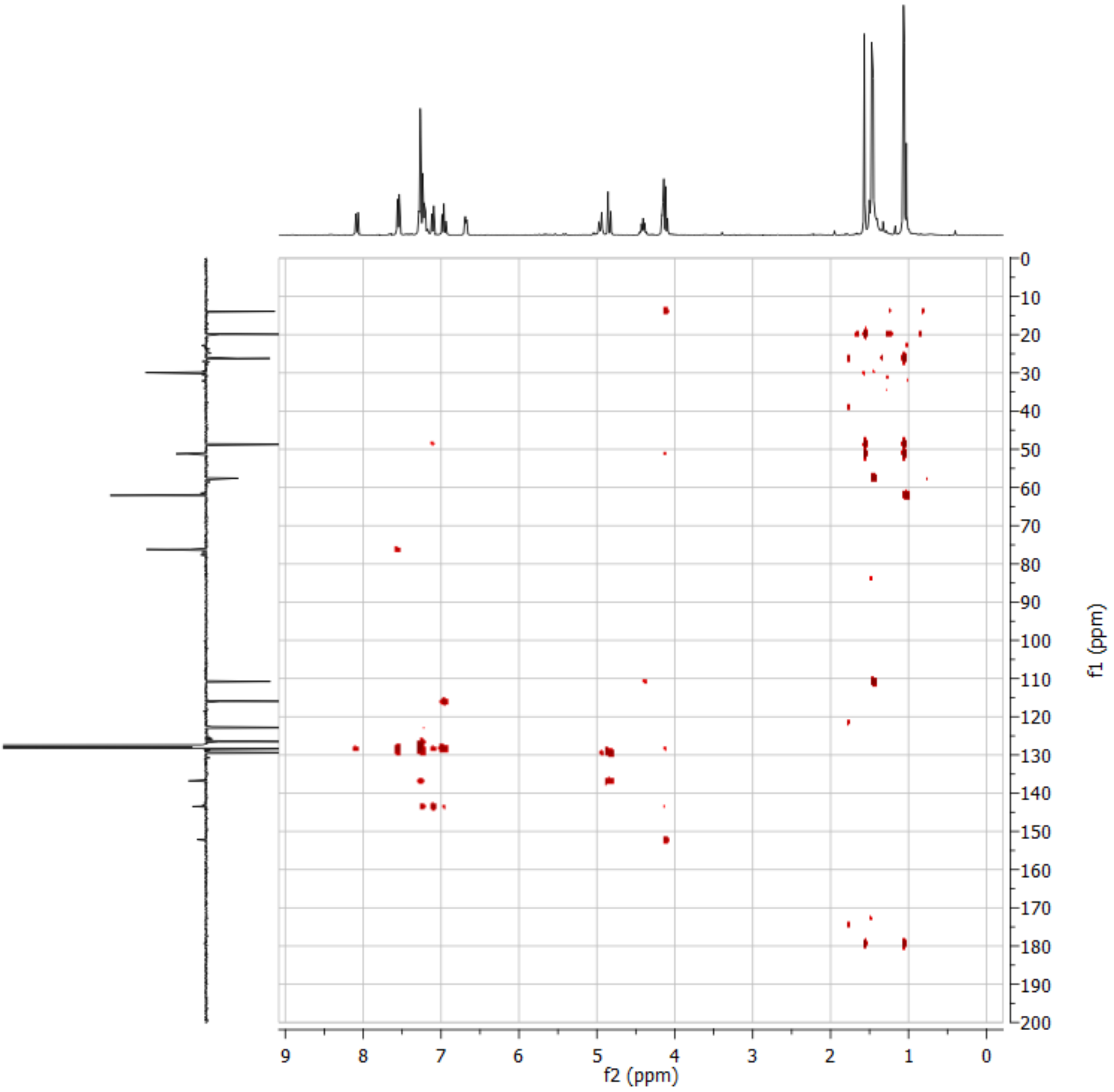

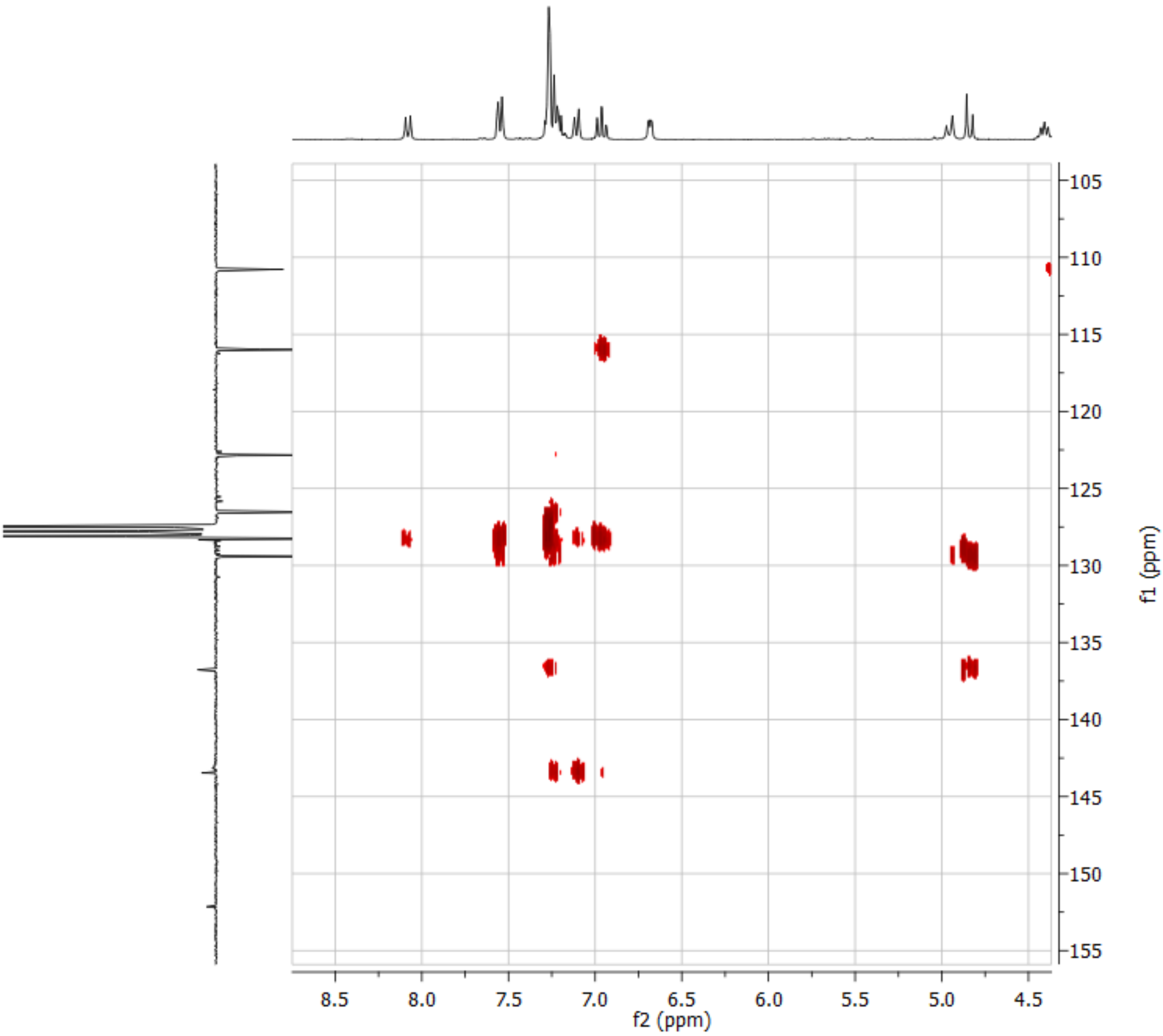
f2 (ppm)
f1 (ppm)
8.5
8.0
7.5
7.0
6.5
6.0
5.5
5.0
4.5
105
110
115
120
125
130
135
140
145
150
155

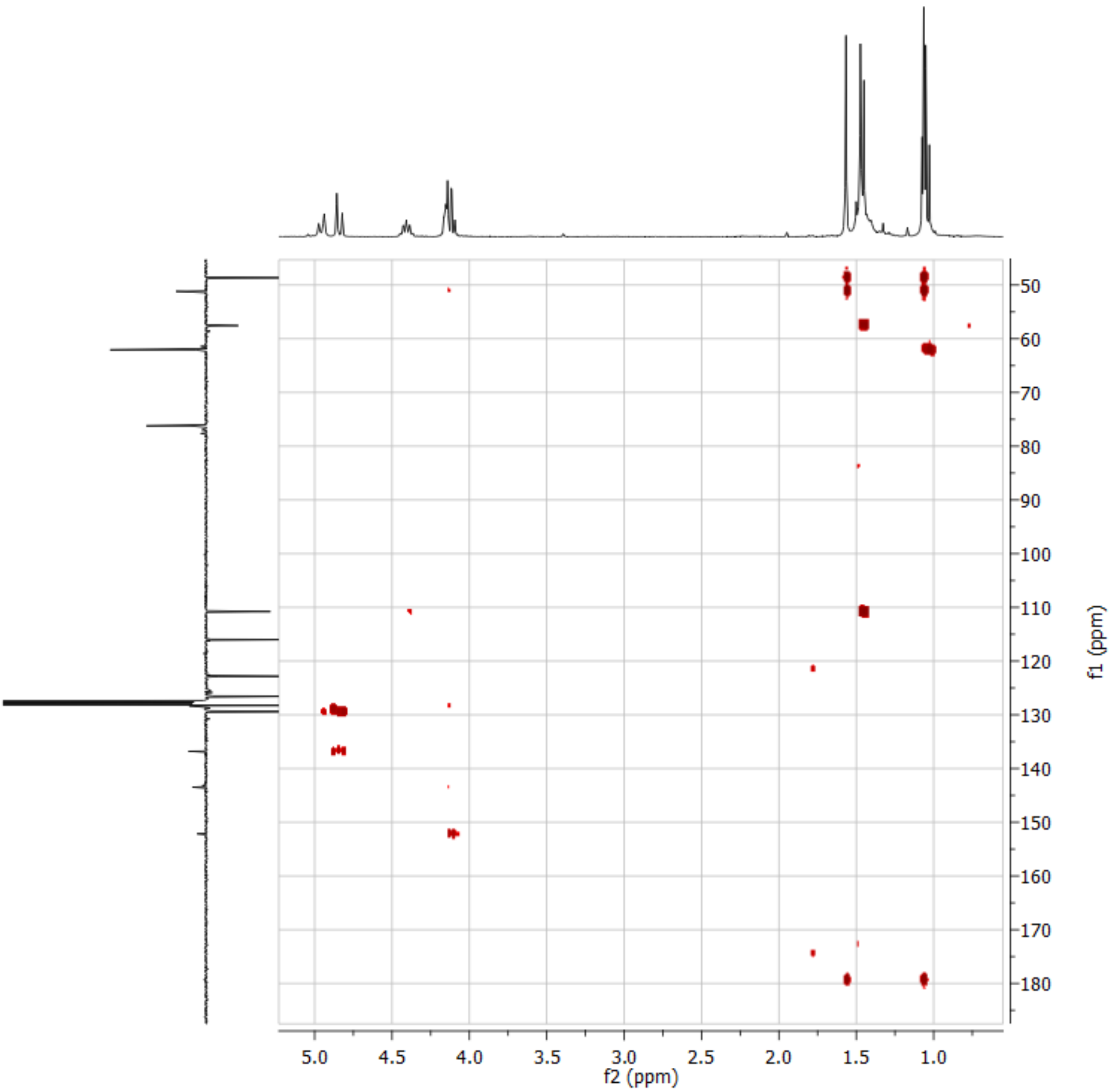

50
60
70
80
90
100
110
120
130
140
150
160
170
180
f1 (ppm)
5.0
4.5
4.0
3.5
3.0
2.5
2.0
1.5
1.0
f2 (ppm)

9, HMBC in C_6D_6 at T = 300 K

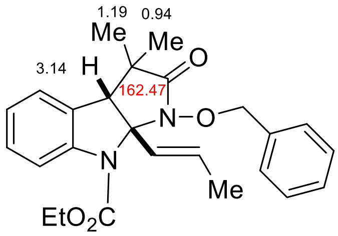

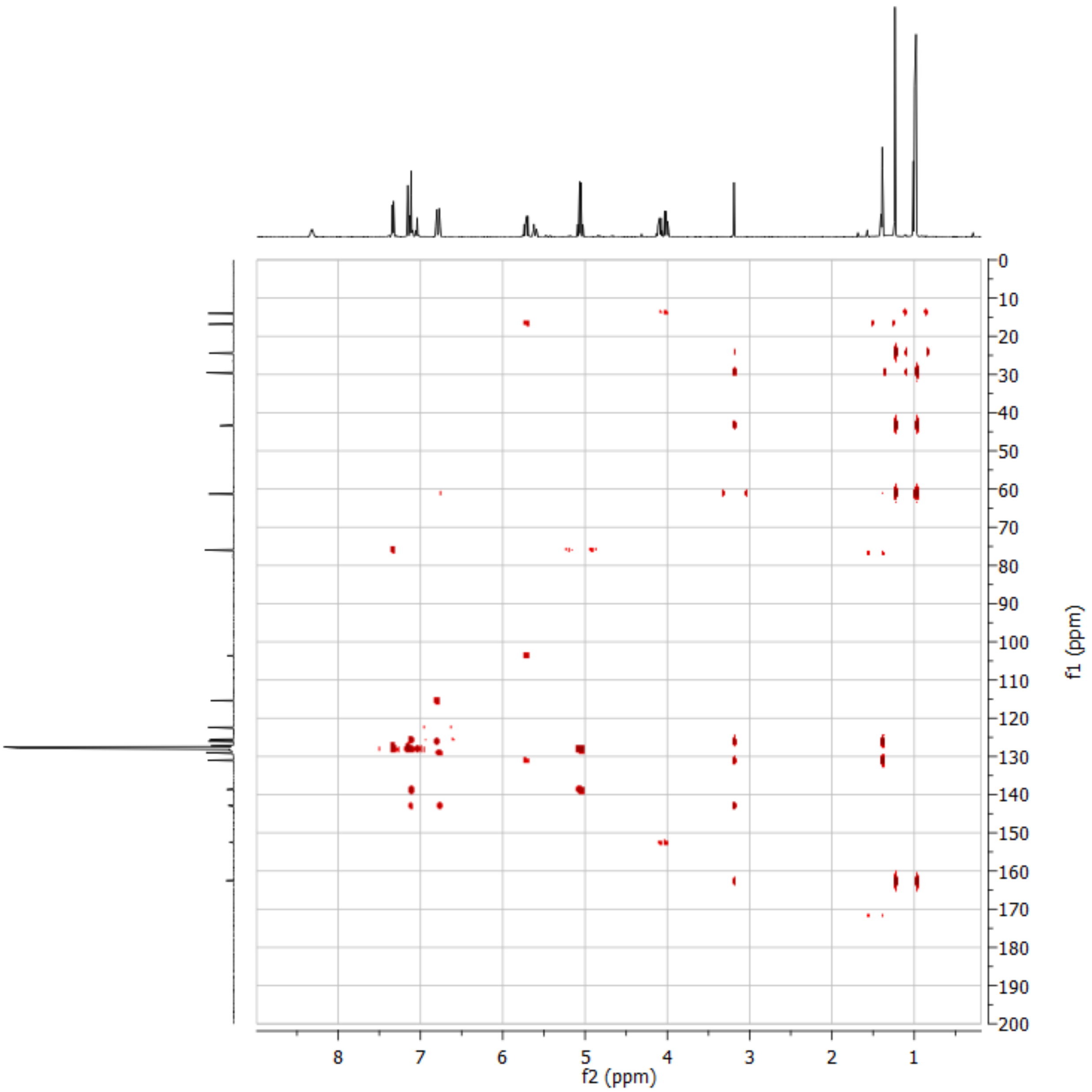

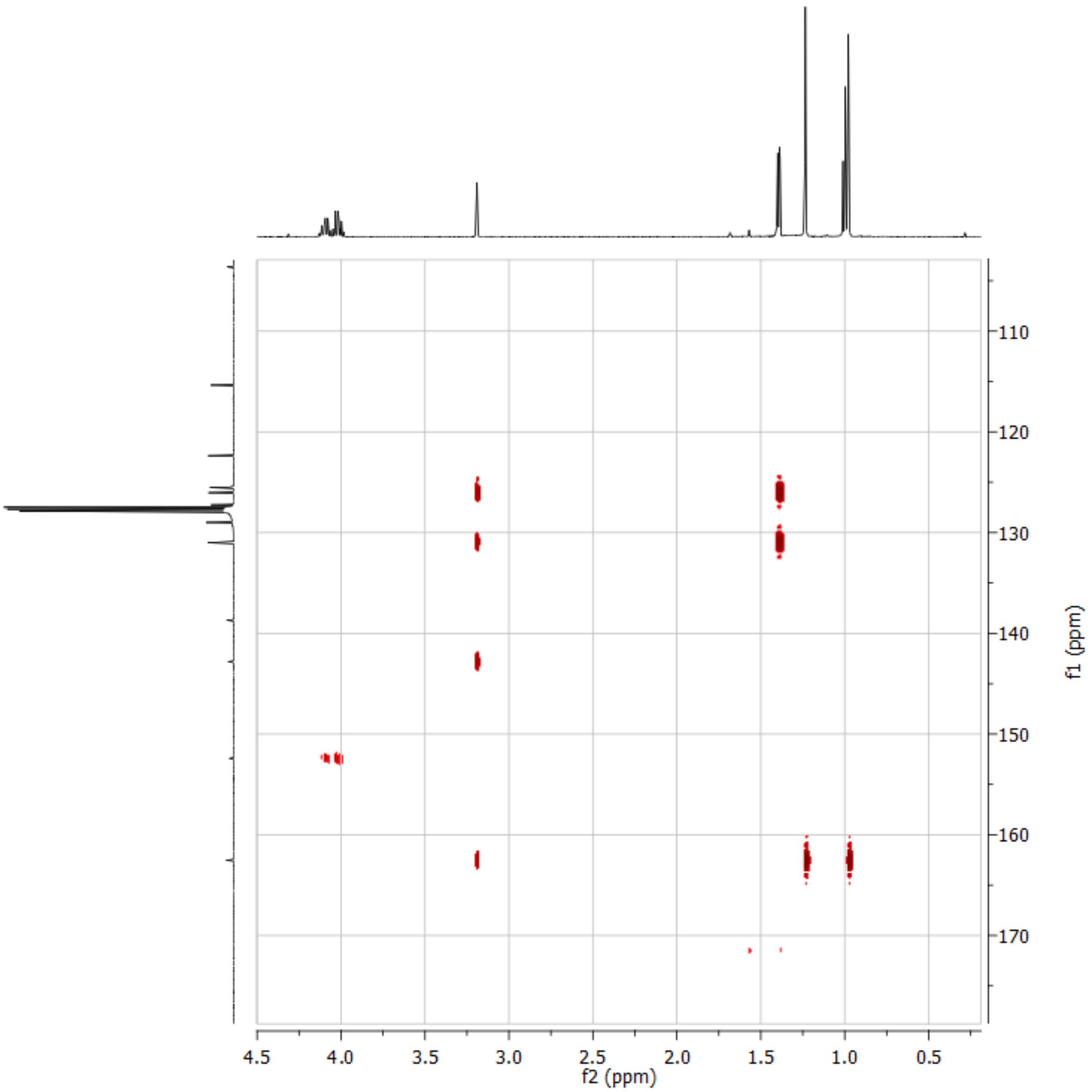
110
120
130
140
150
160
170
f1 (ppm)
4.5
4.0
3.5
3.0
2.5
2.0
1.5
1.0
0.5
f2 (ppm)

10, COSY in C_6D_6 at T = 300 K

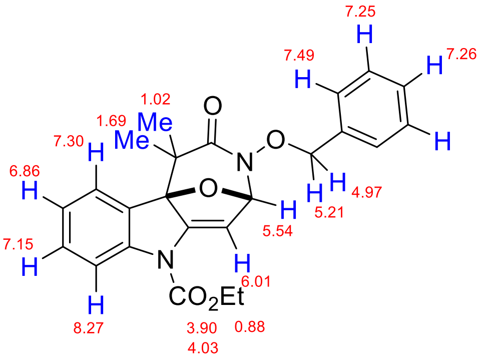

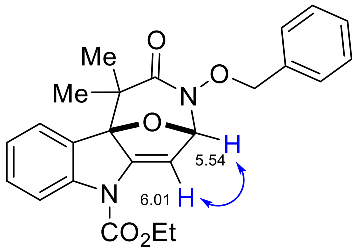

diagnostic cross pick

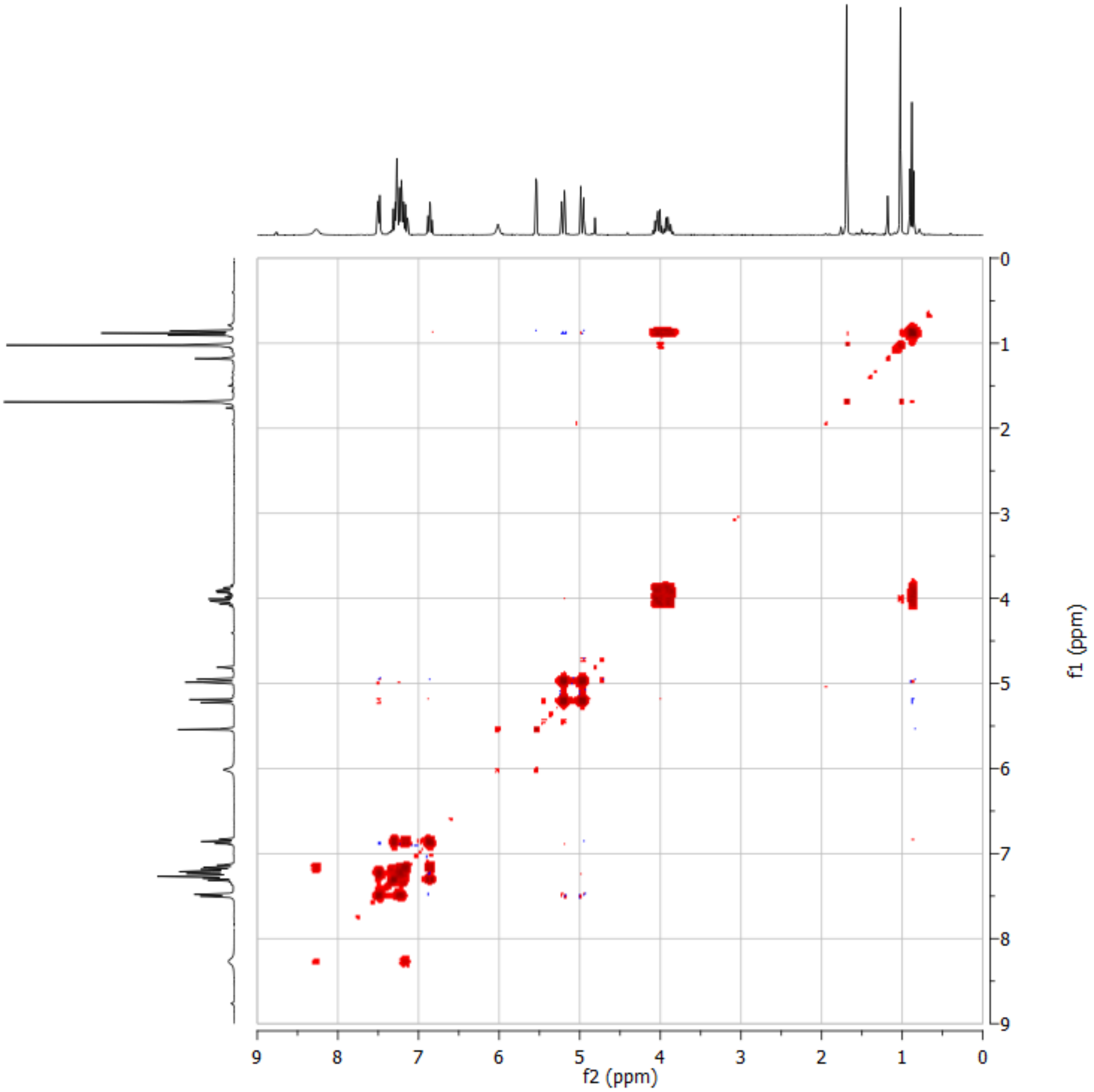

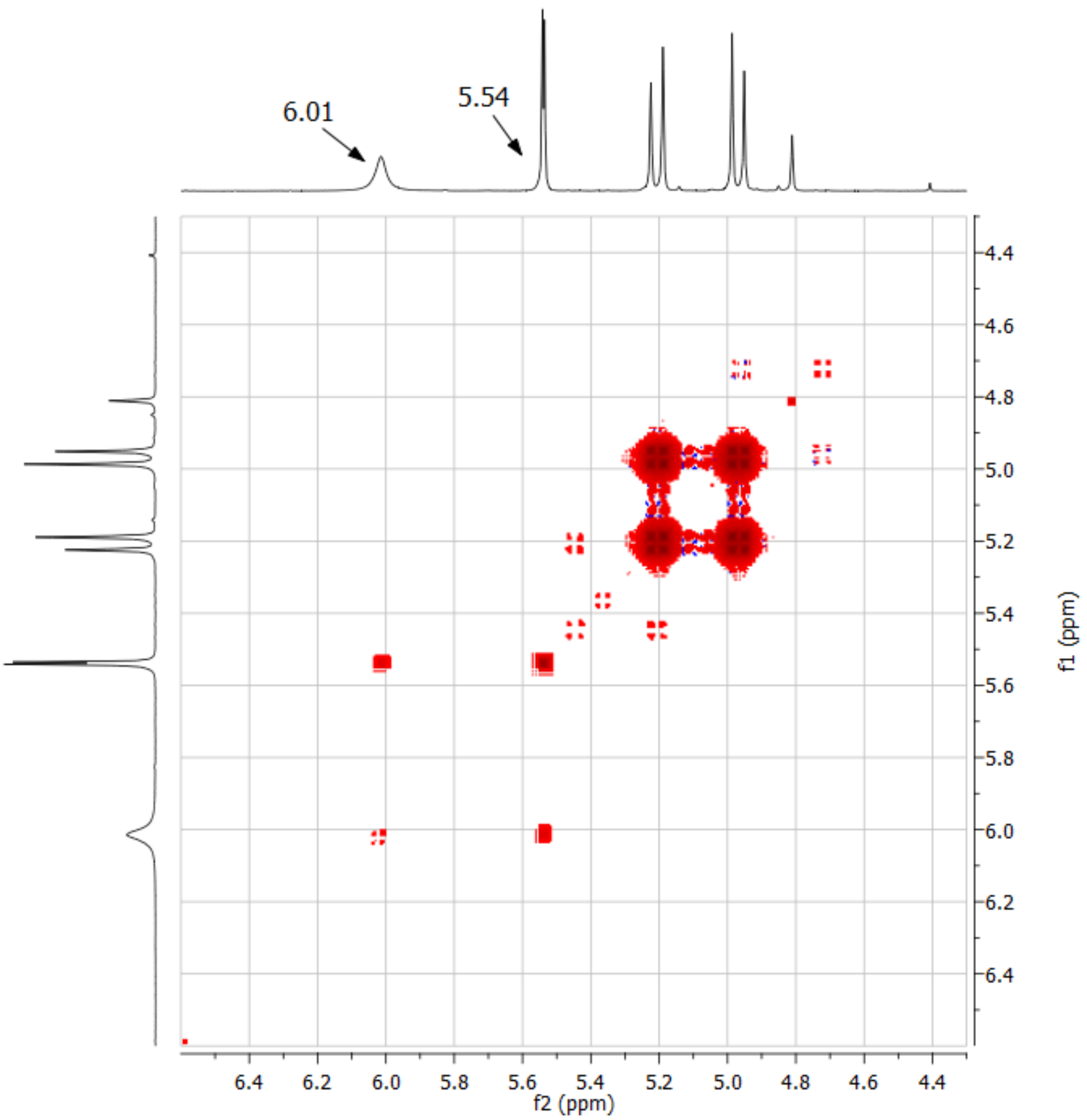
6.01
5.54
6.4
6.2
6.0
5.8
5.6
5.4
5.2
5.0
4.8
4.6
4.4
f2 (ppm)
f1 (ppm)

10, HSQC in C_6D_6 at T = 300 K

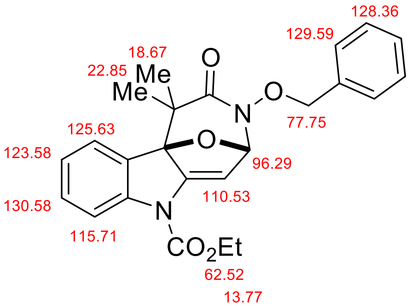

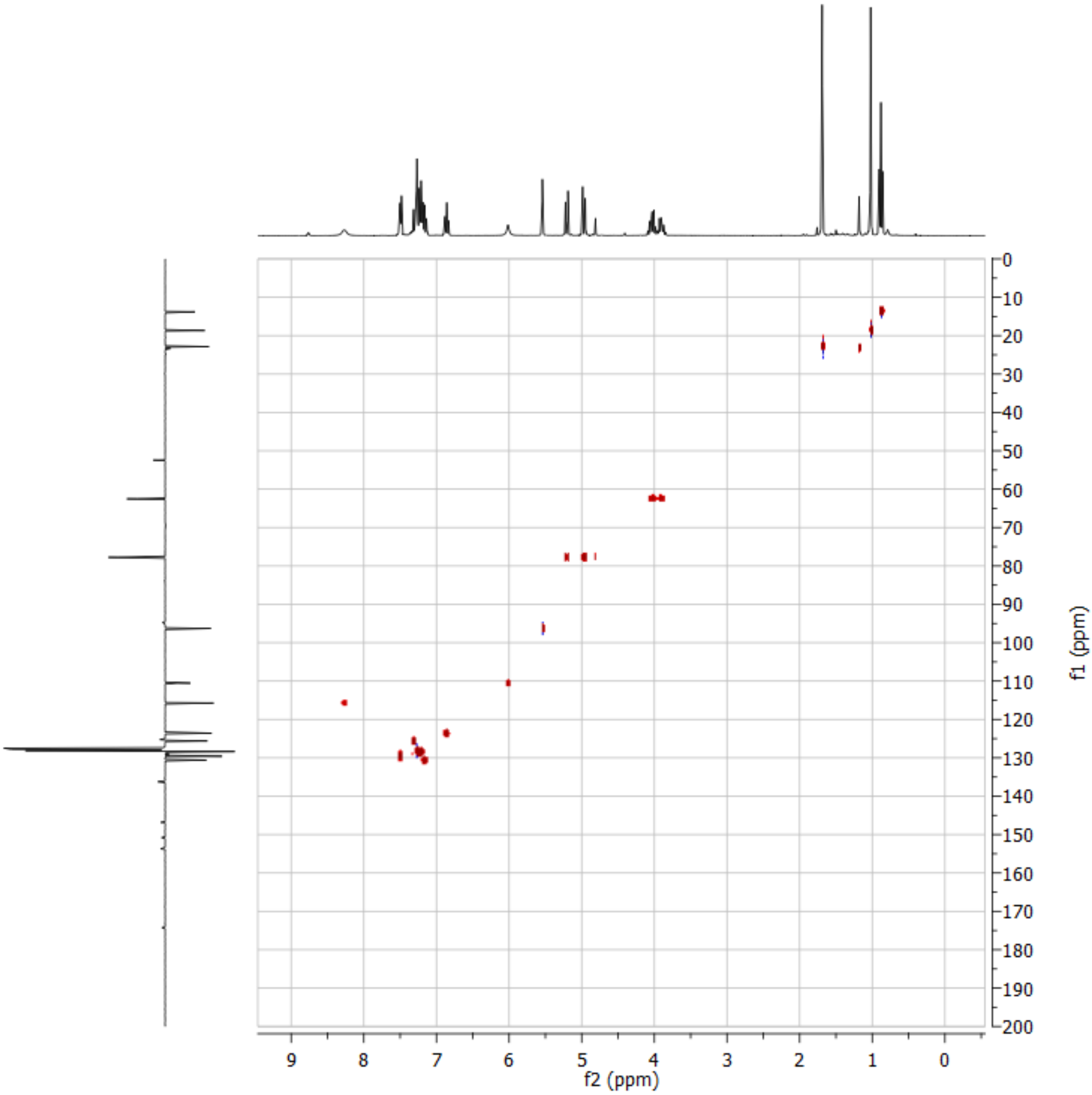

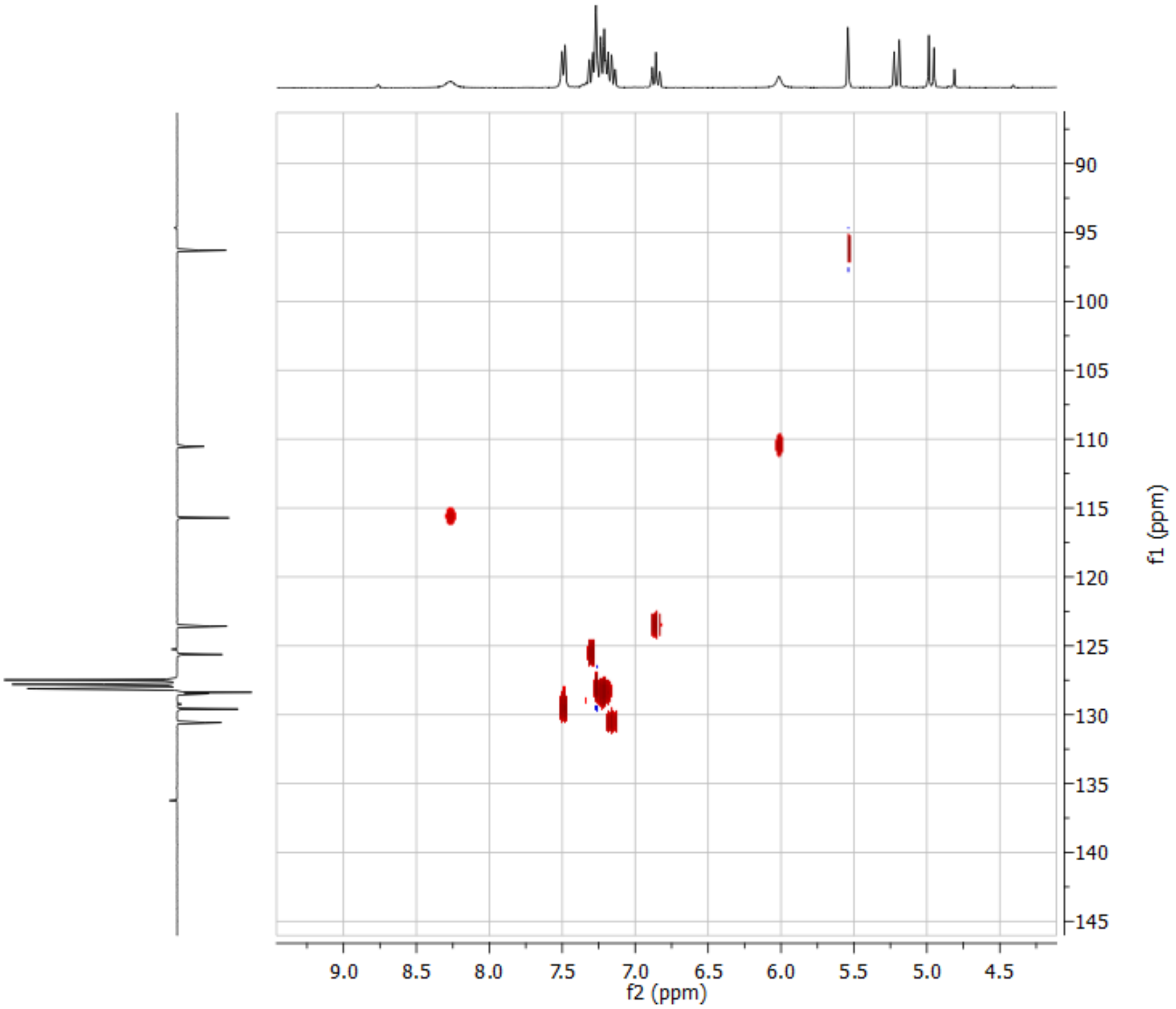

90
95
100
105
110
115
120
125
130
135
140
145
f1 (ppm)
9.0
8.5
8.0
7.5
7.0
6.5
6.0
5.5
5.0
4.5
f2 (ppm)

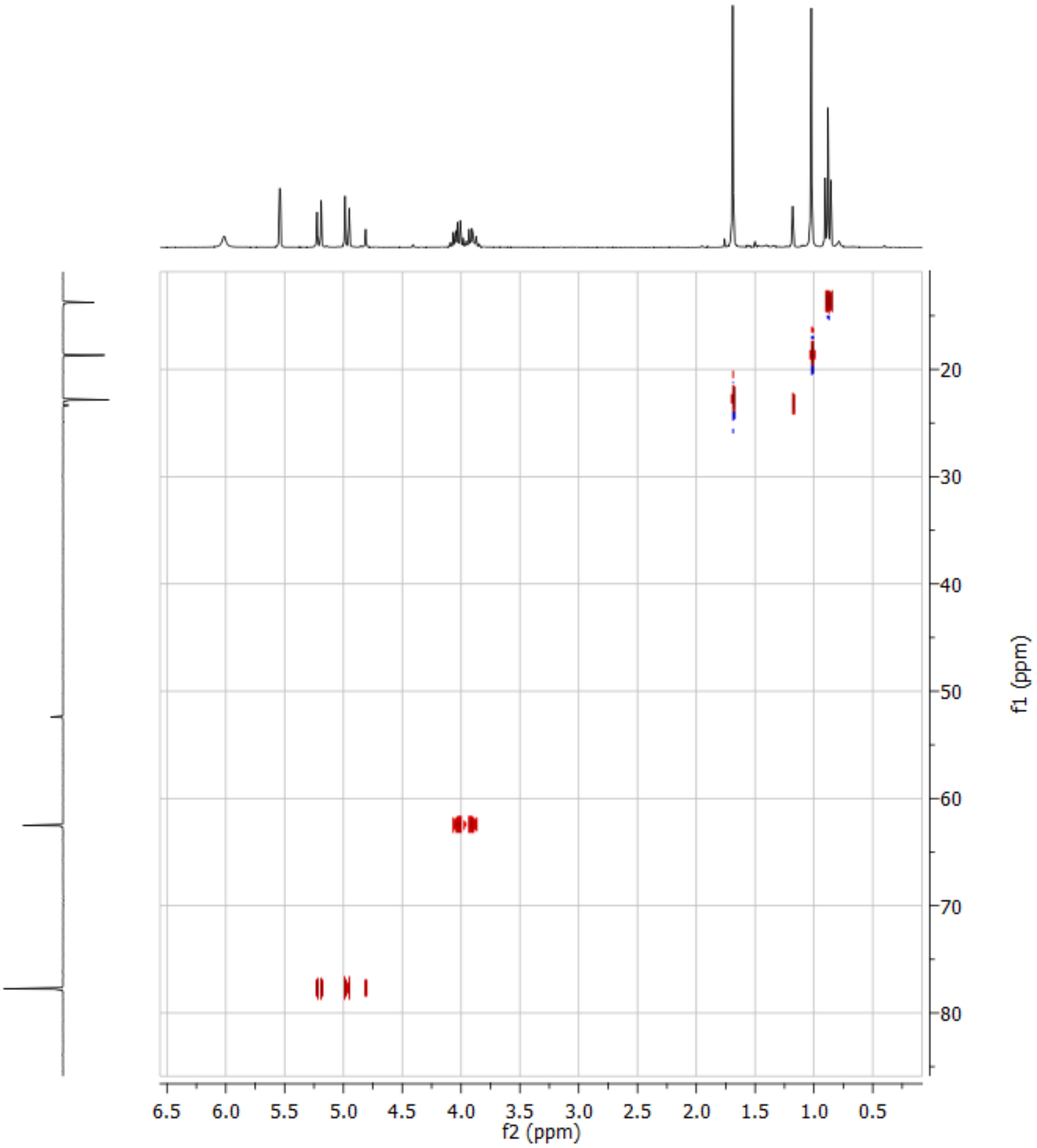

f1 (ppm)
20
30
40
50
60
70
80
6.5
6.0
5.5
5.0
4.5
4.0
3.5
3.0
2.5
2.0
1.5
1.0
0.5
f2 (ppm)

10, NOESY in C_6D_6 at T = 300 K

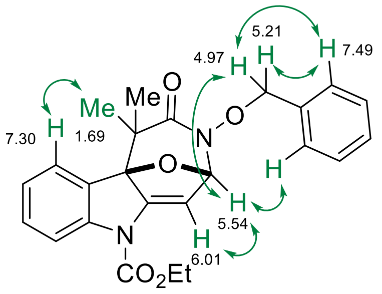

diagnostic cross picks

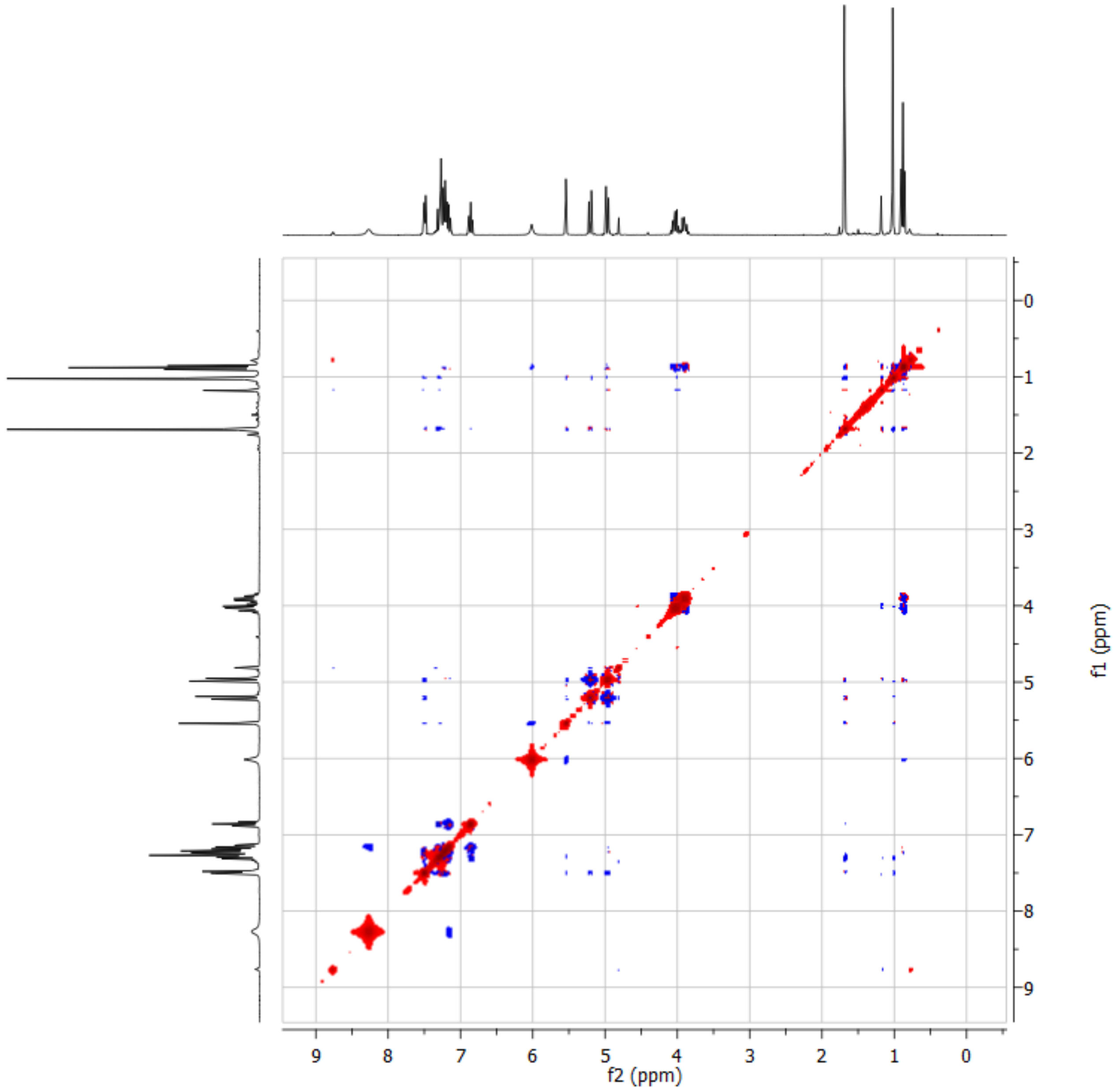

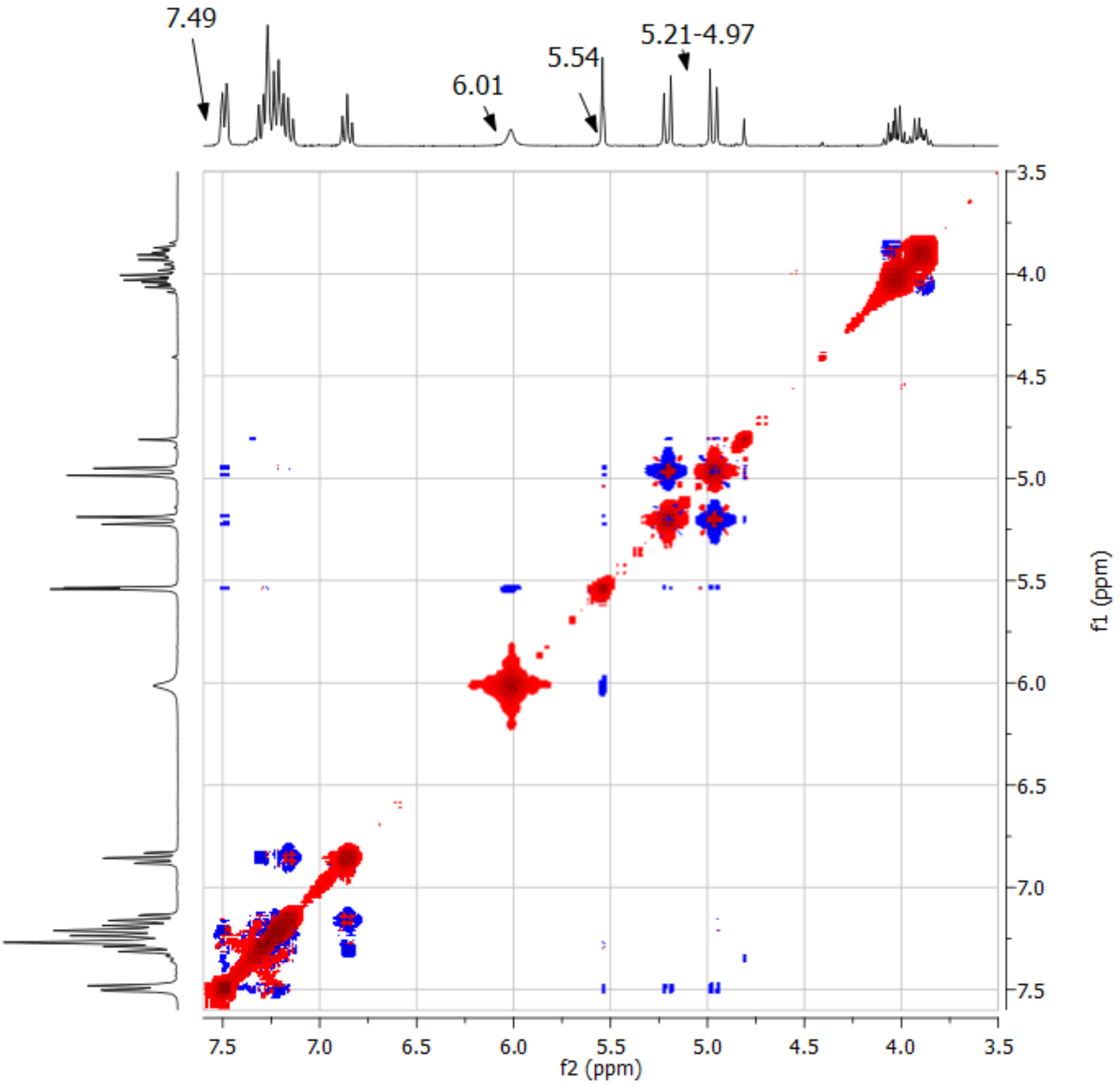

7.49
6.01
5.54
5.21-4.97
f2 (ppm)
f1 (ppm)

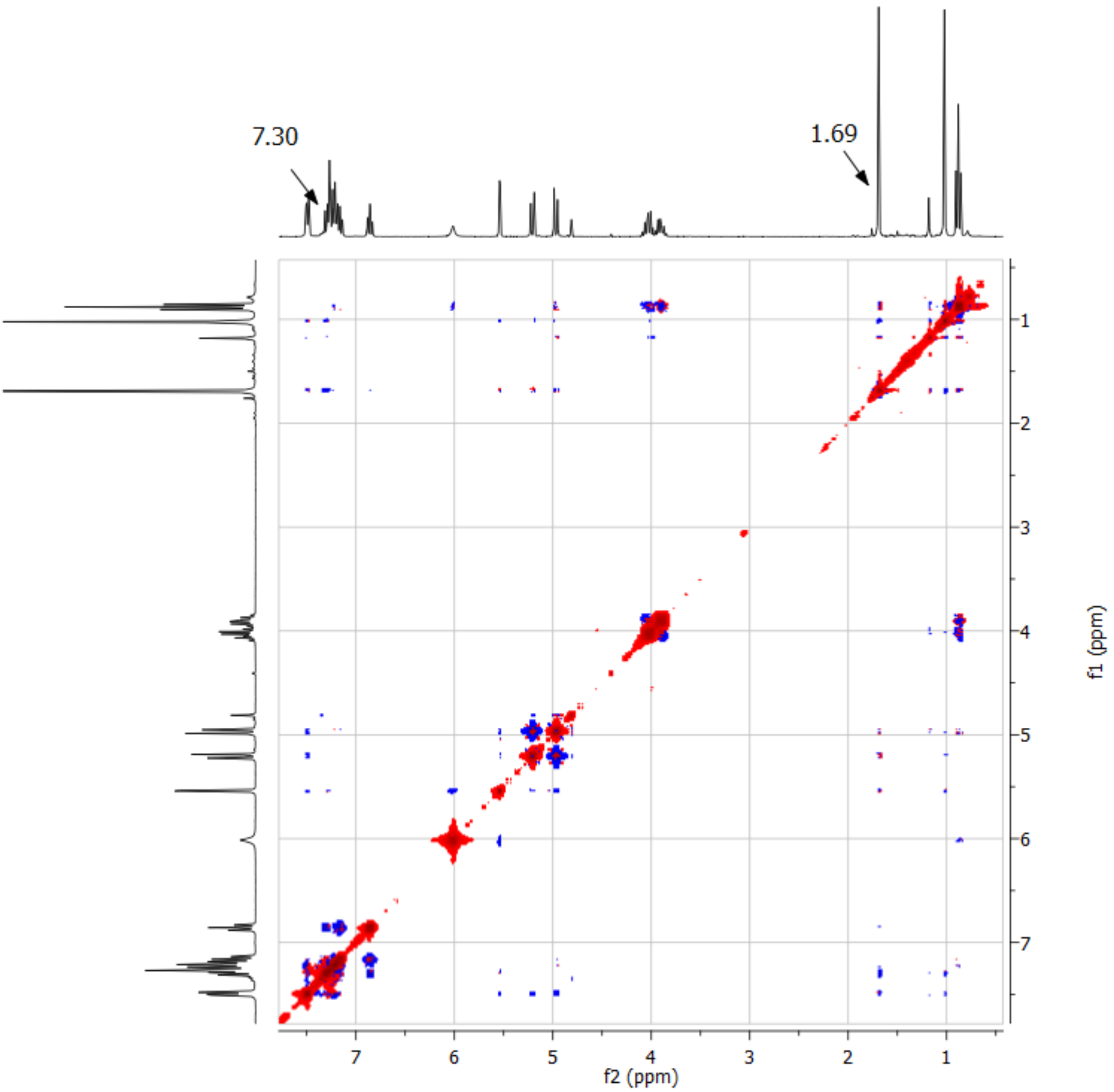
7.30
1.69
7
6
5
4
3
2
1
f2 (ppm)
f1 (ppm)

12, COSY in C_6D_6 at T = 300 K

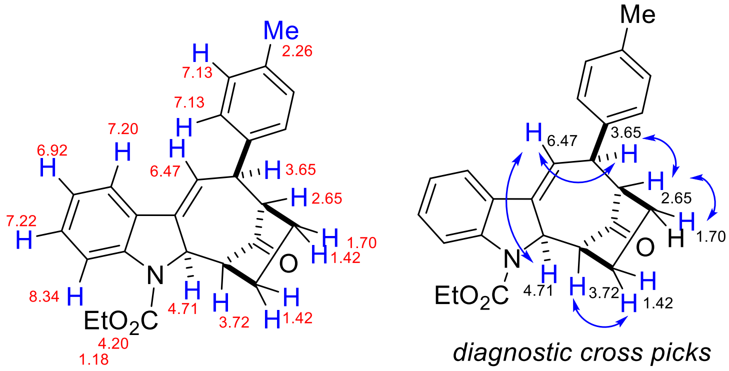

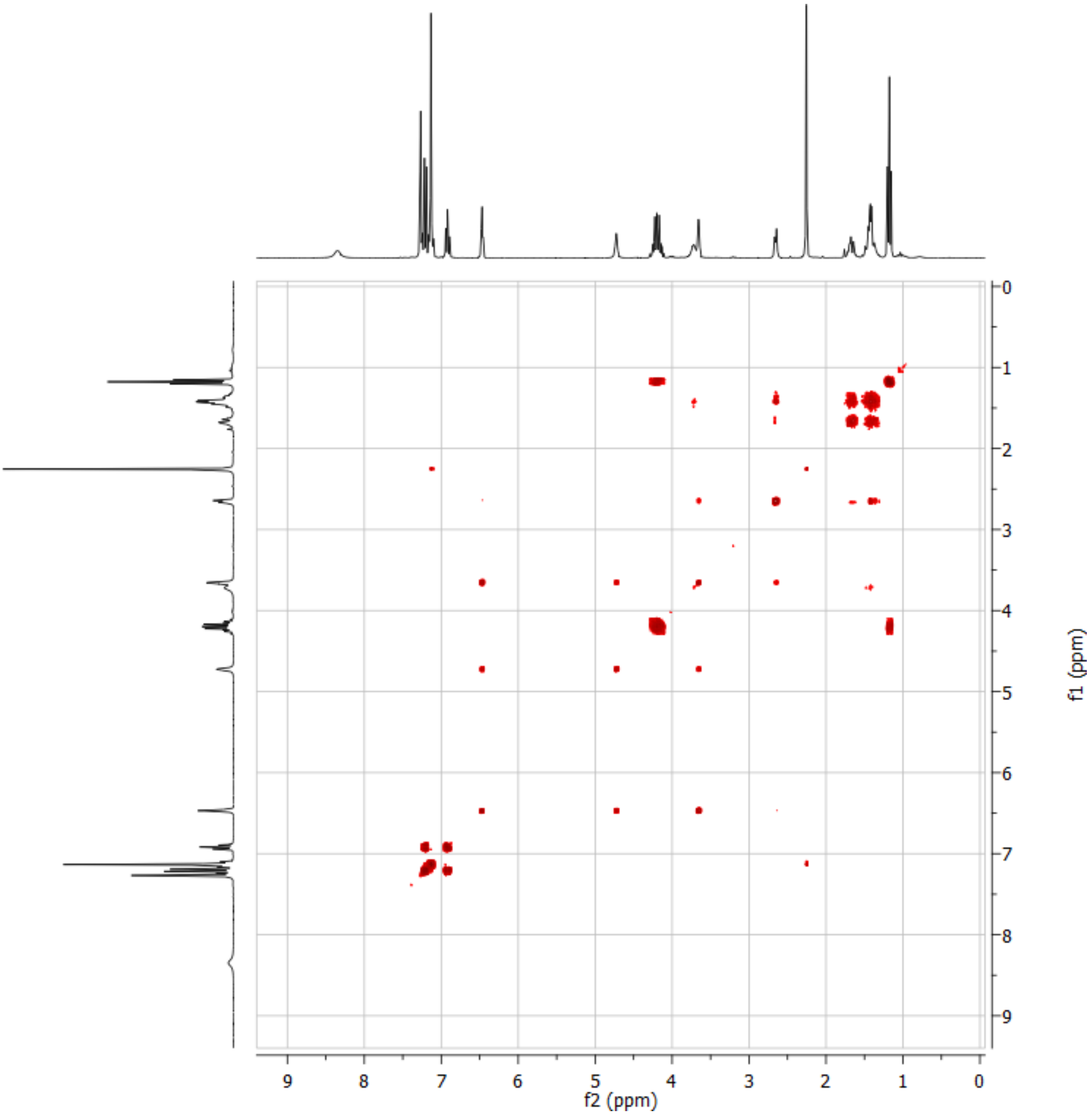

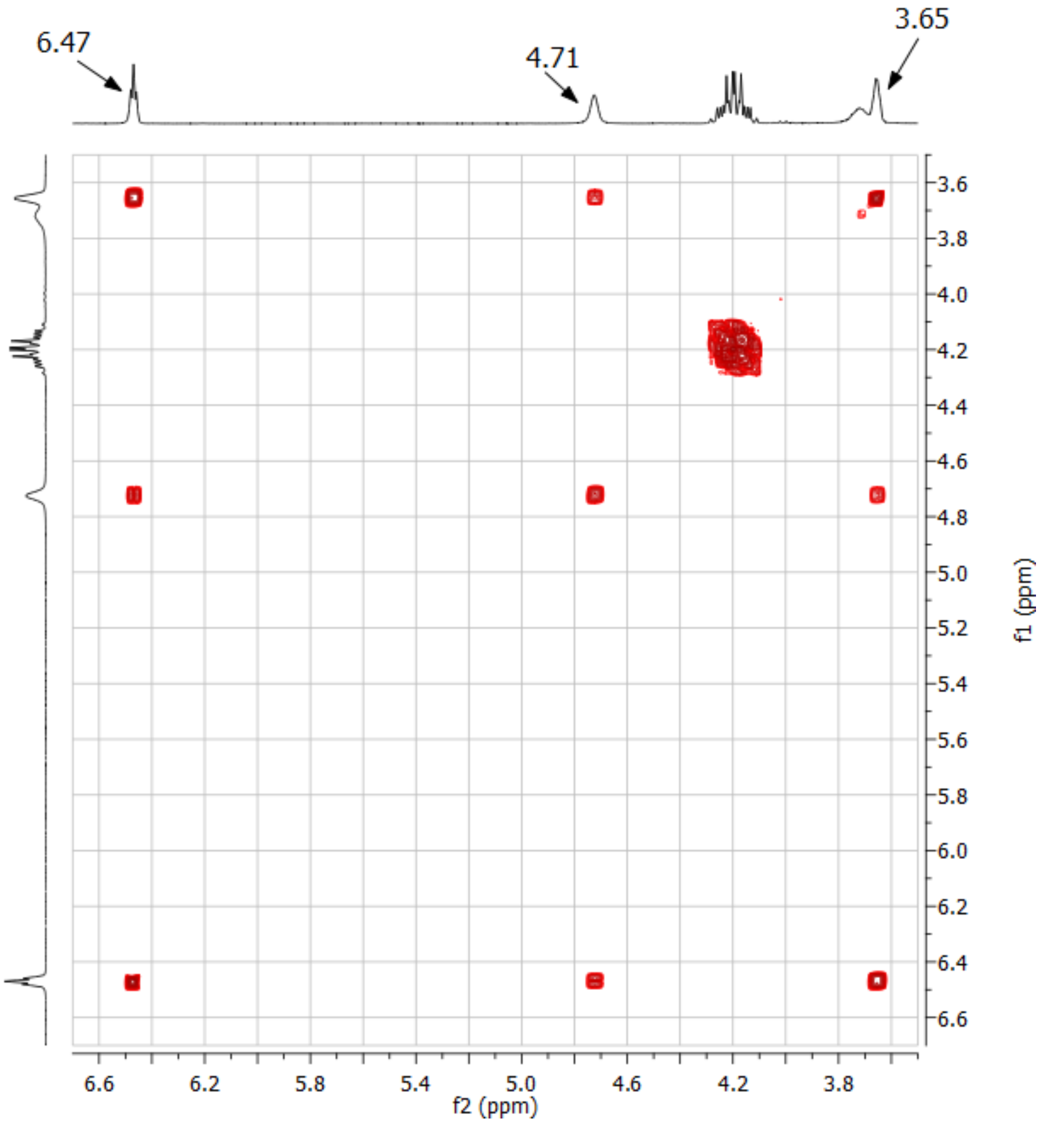

6.47
4.71
3.65
6.6
6.2
5.8
5.4
5.0
4.6
4.2
3.8
f2 (ppm)
3.6
3.8
4.0
4.2
4.4
4.6
4.8
5.0
5.2
5.4
5.6
5.8
6.0
6.2
6.4
6.6
f1 (ppm)

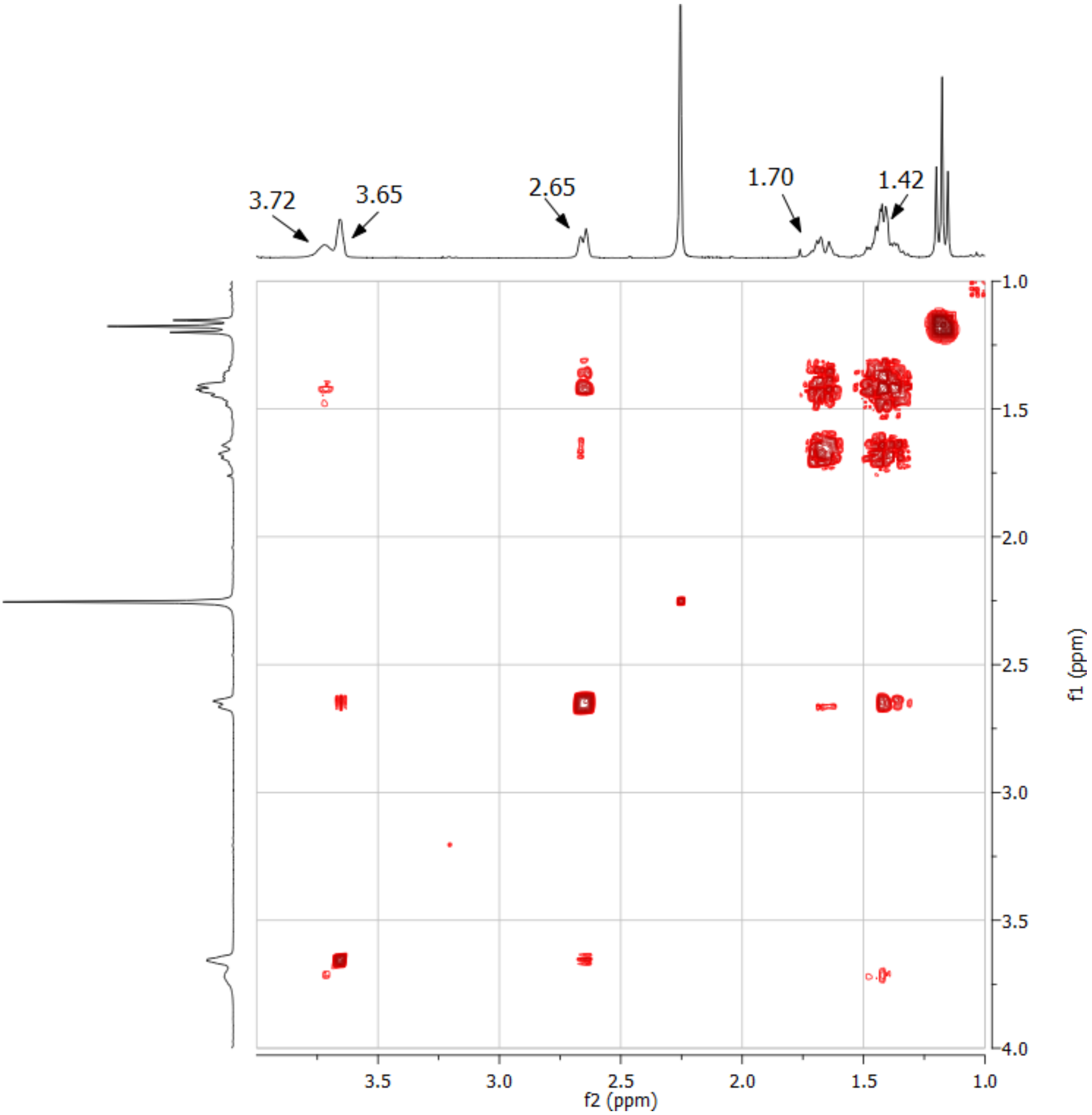

3.72
3.65
2.65
1.70
1.42
1.0
1.5
2.0
2.5
3.0
3.5
4.0
f1 (ppm)
3.5
3.0
2.5
2.0
1.5
1.0
f2 (ppm)

12, HSQC in C_6D_6 at T = 300 K

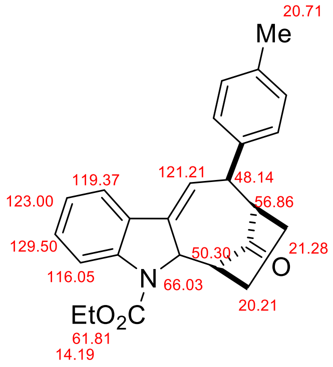

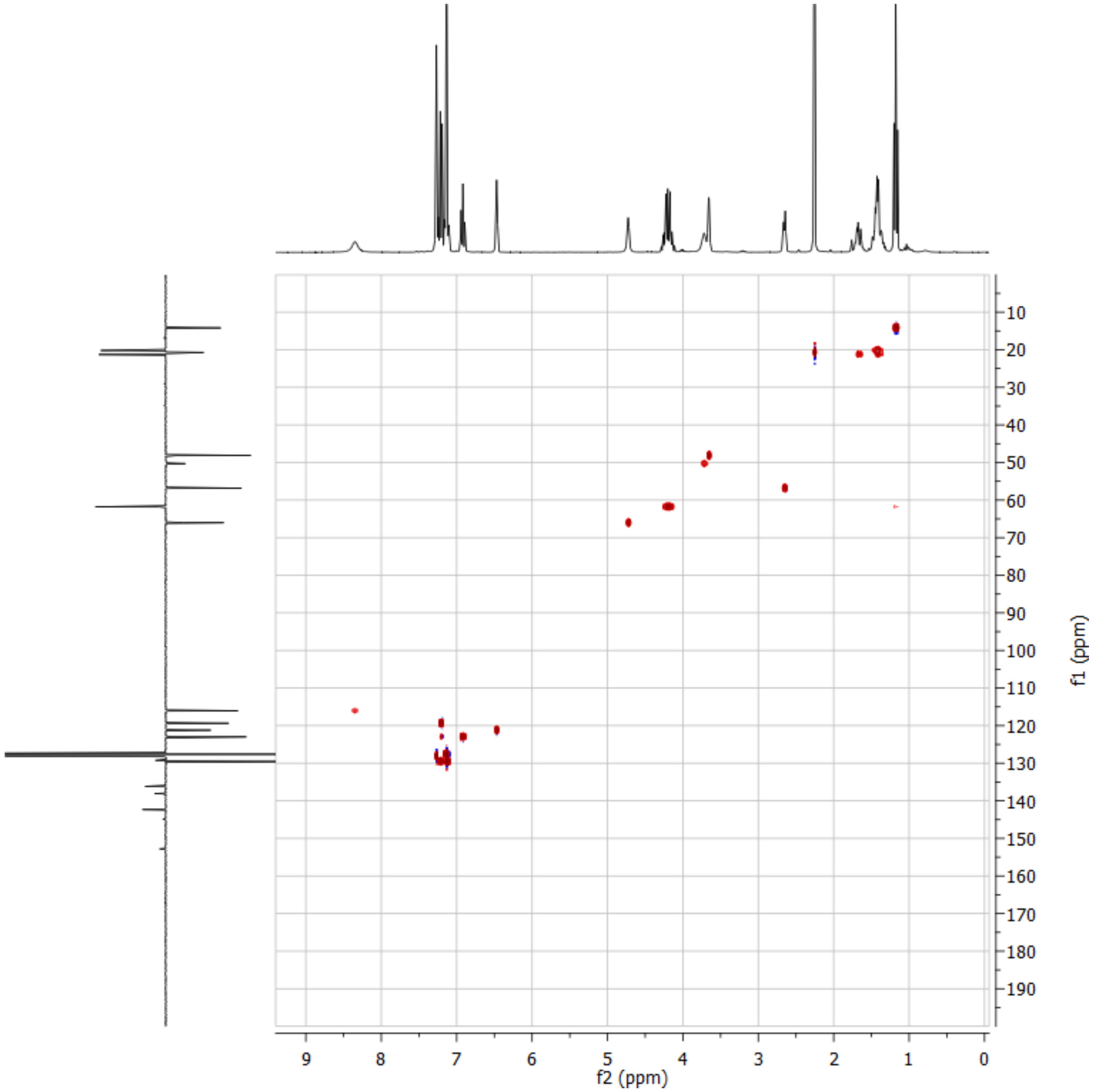

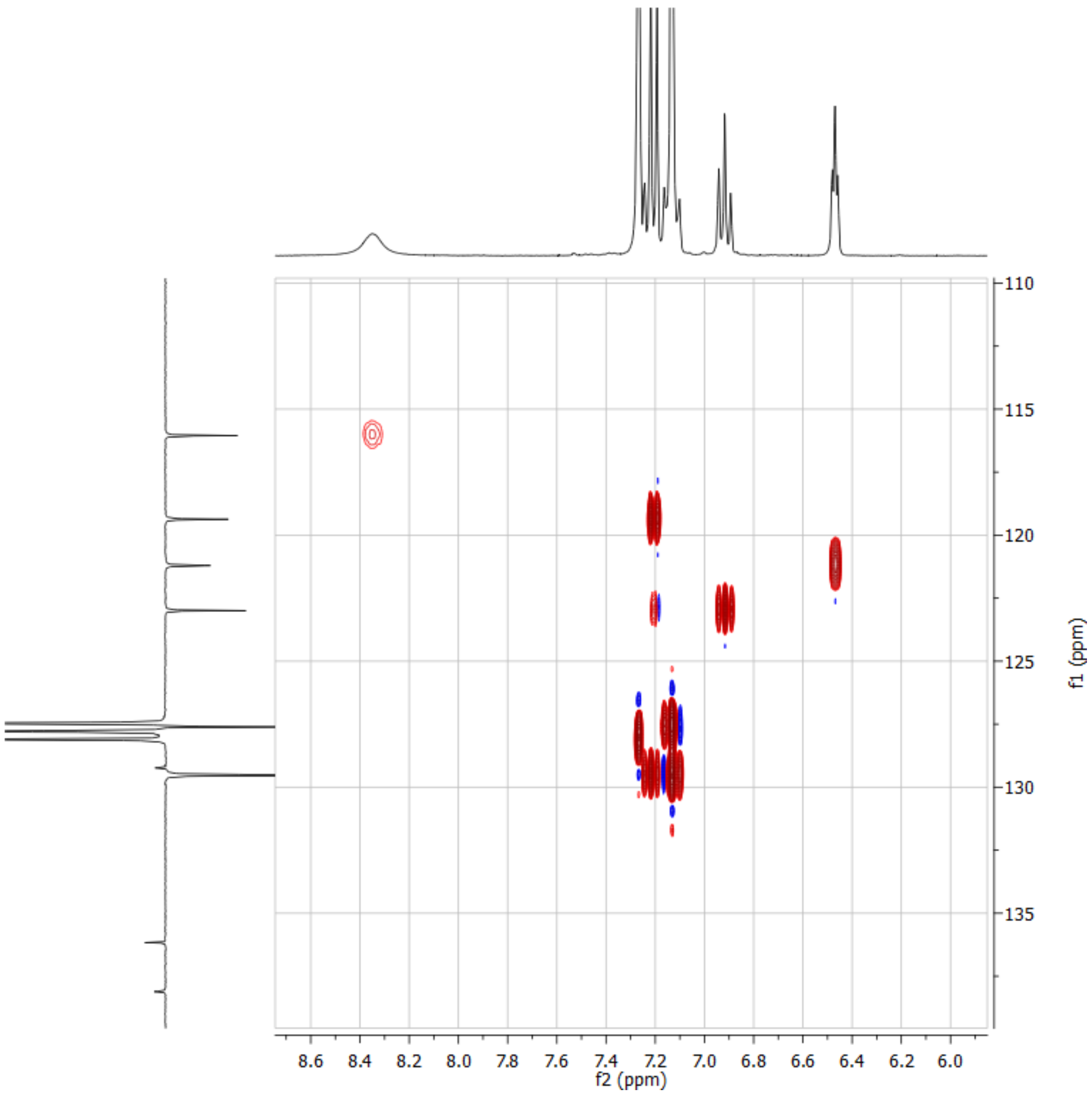
110
115
120
125
130
135
f1 (ppm)
8.6
8.4
8.2
8.0
7.8
7.6
7.4
7.2
7.0
6.8
6.6
6.4
6.2
6.0
f2 (ppm)

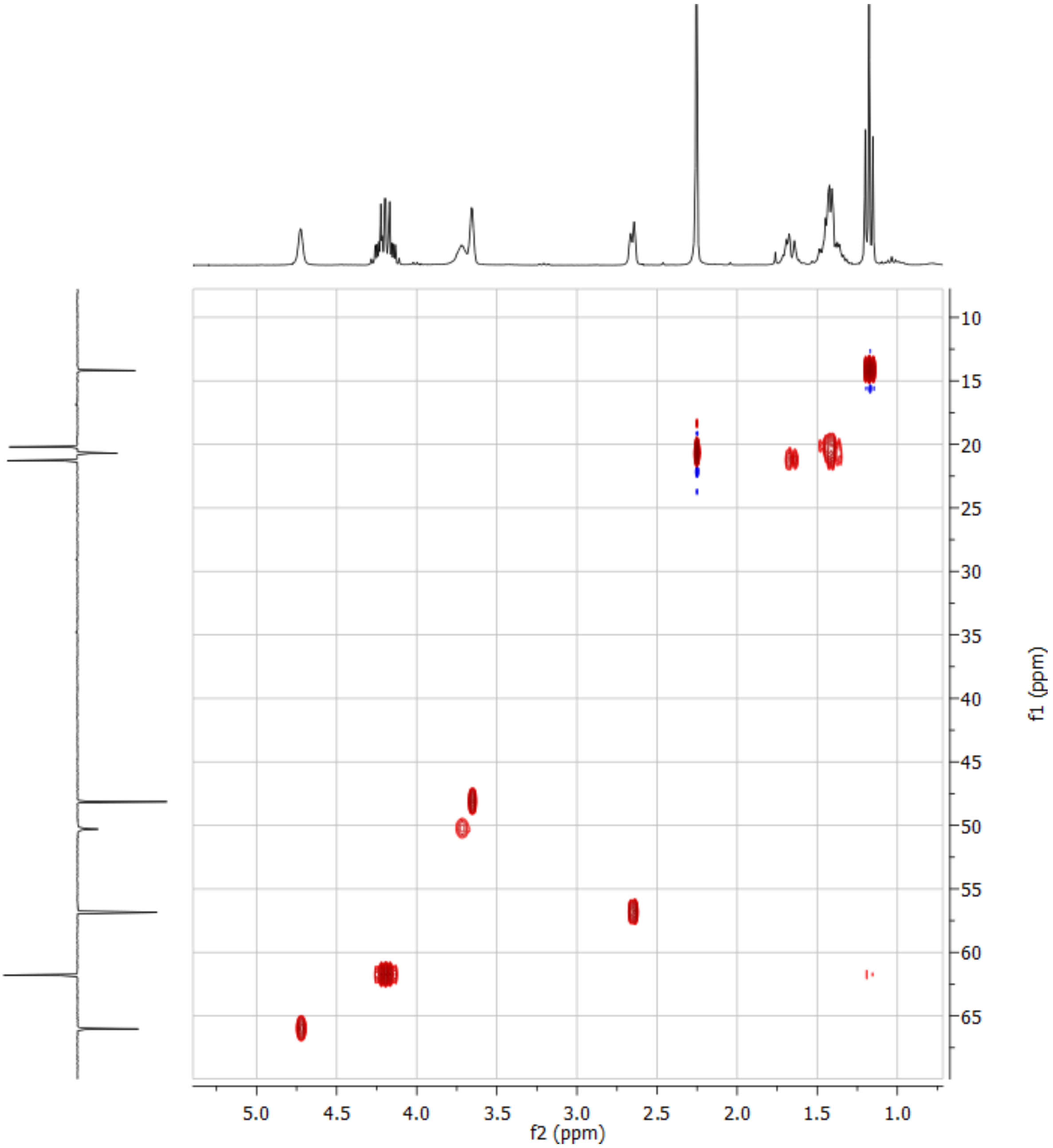

10
15
20
25
30
35
40
45
50
55
60
65
f1 (ppm)
5.0
4.5
4.0
3.5
3.0
2.5
2.0
1.5
1.0
f2 (ppm)

12, NOESY in C_6D_6 at T = 300 K

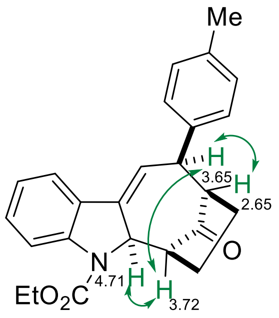

diagnostic cross picks

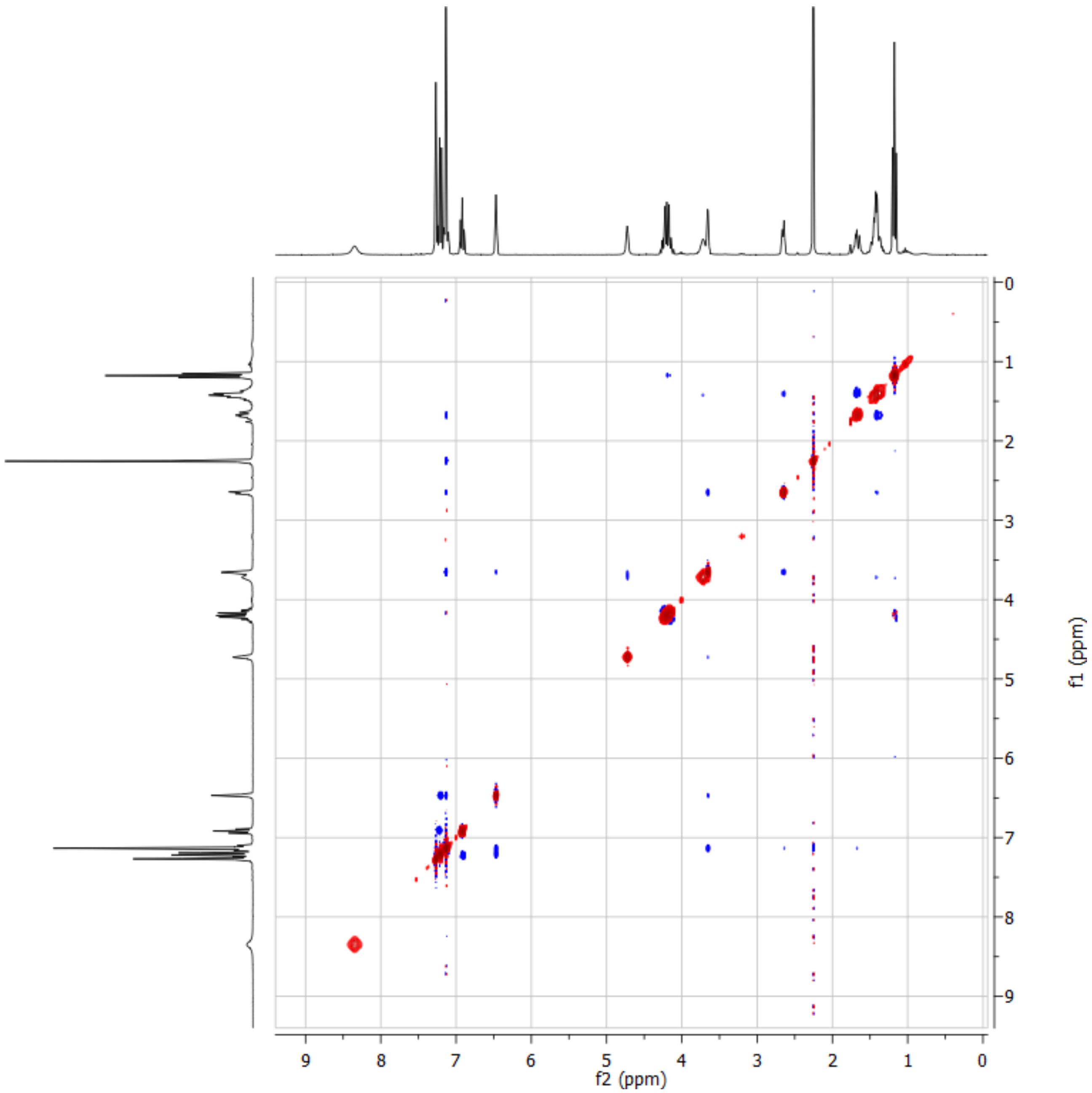

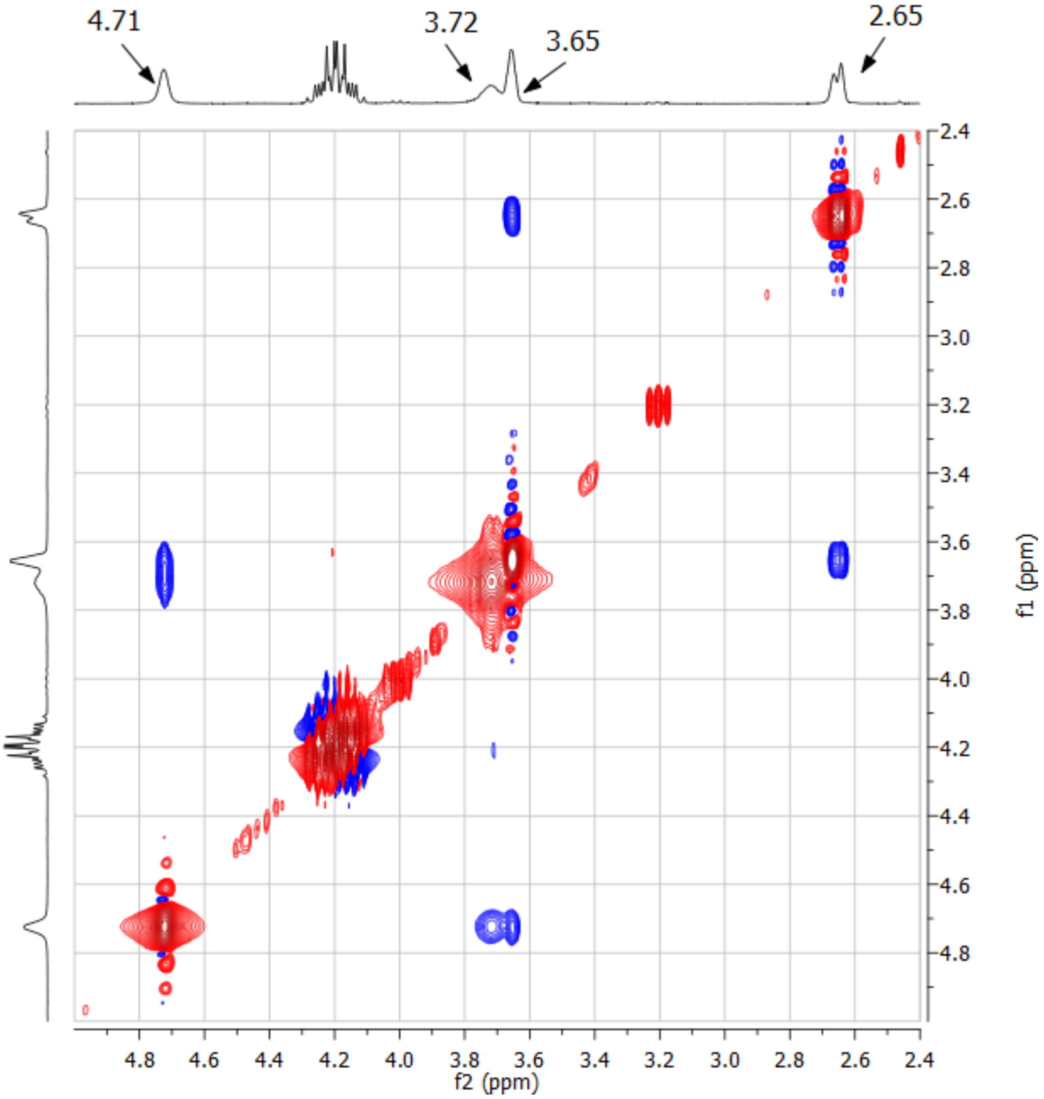

4.71
3.72
3.65
2.65
f2 (ppm)
f1 (ppm)

12, HMBC in C_6D_6 at T = 300 K

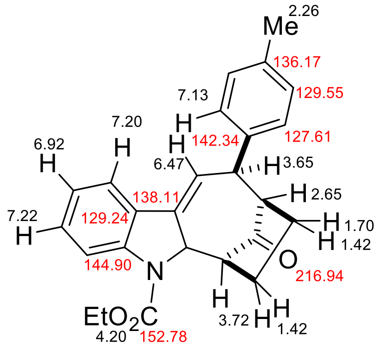

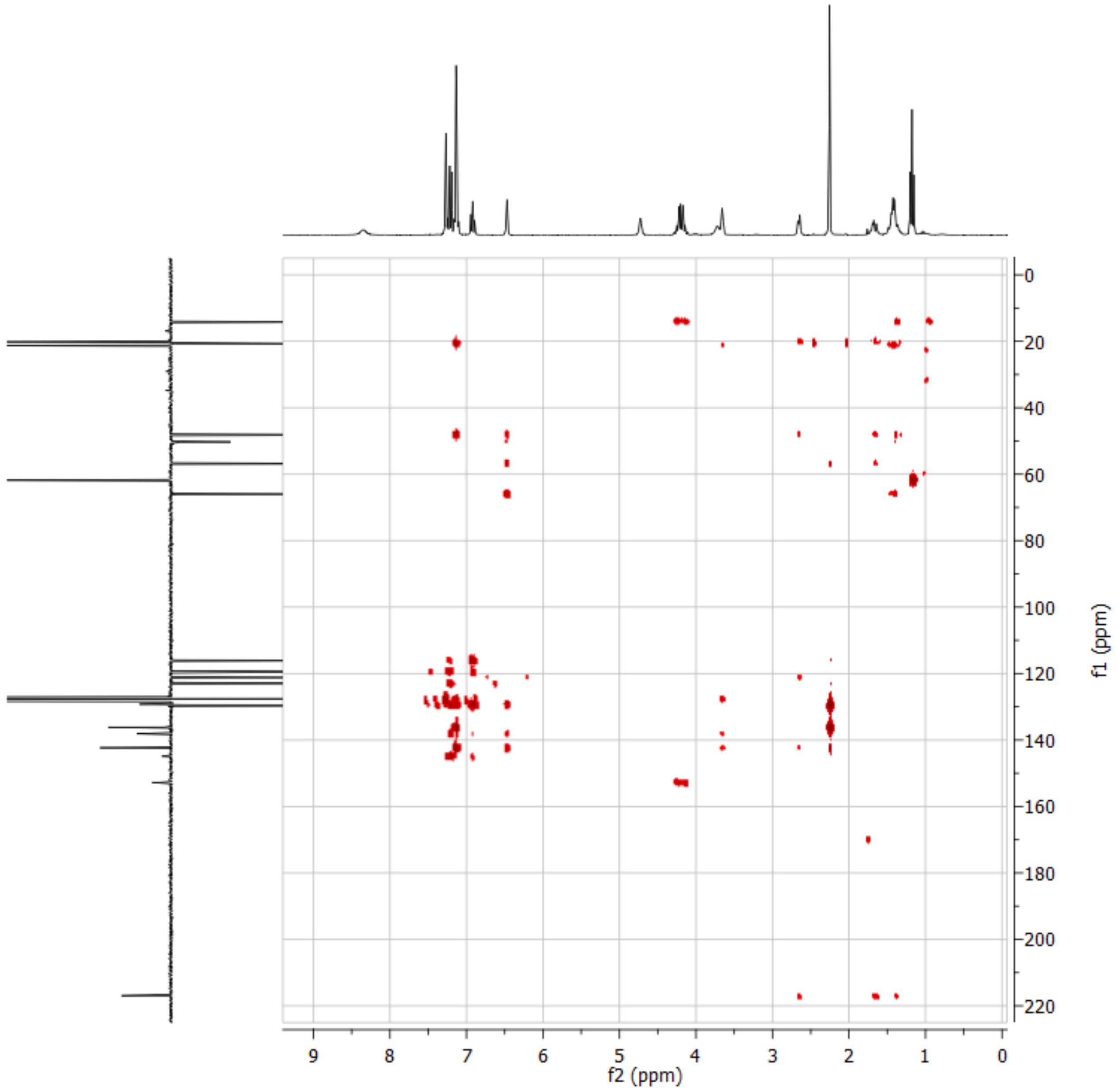

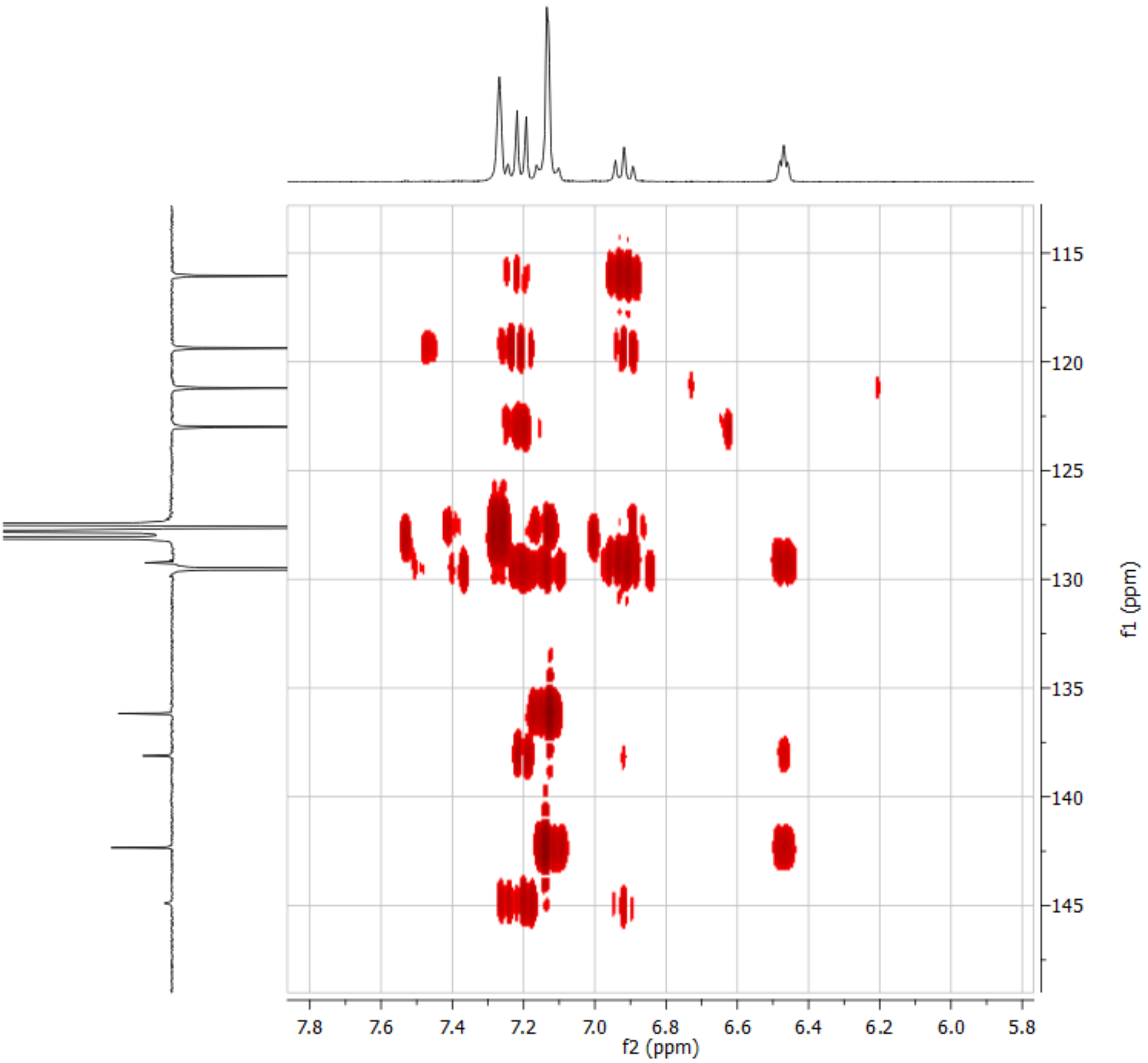

115
120
125
130
135
140
145
f1 (ppm)
7.8
7.6
7.4
7.2
7.0
6.8
6.6
6.4
6.2
6.0
5.8
f2 (ppm)

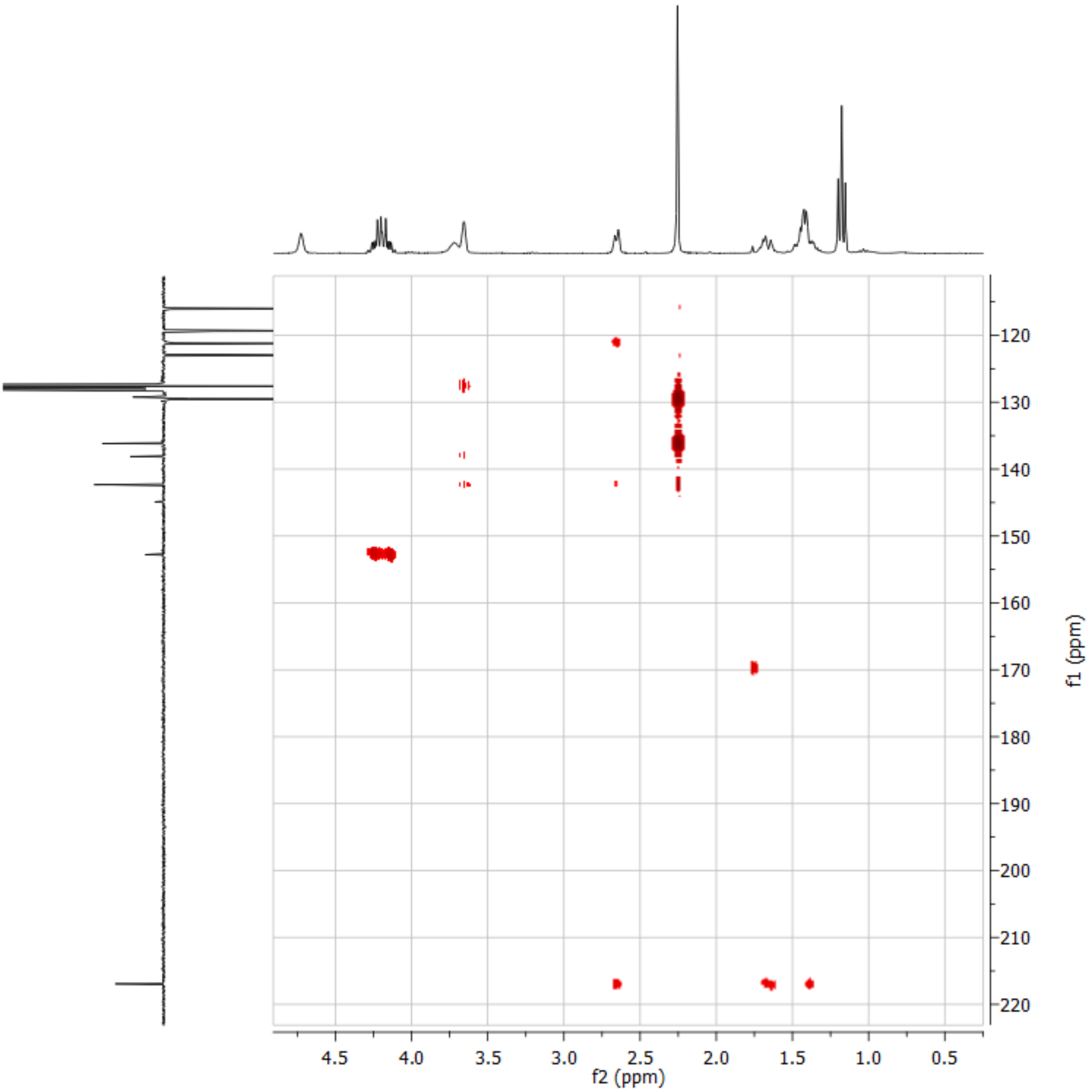

f1 (ppm)
120
130
140
150
160
170
180
190
200
210
220
4.5
4.0
3.5
3.0
2.5
2.0
1.5
1.0
0.5
f2 (ppm)

16, COSY in C_6D_6 at T = 300 K

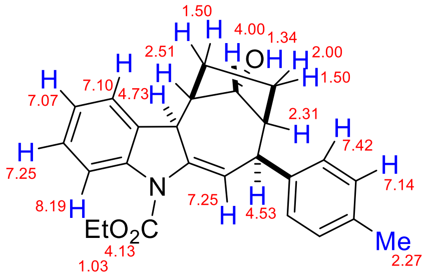

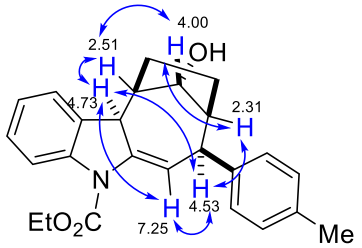

diagnostic cross picks

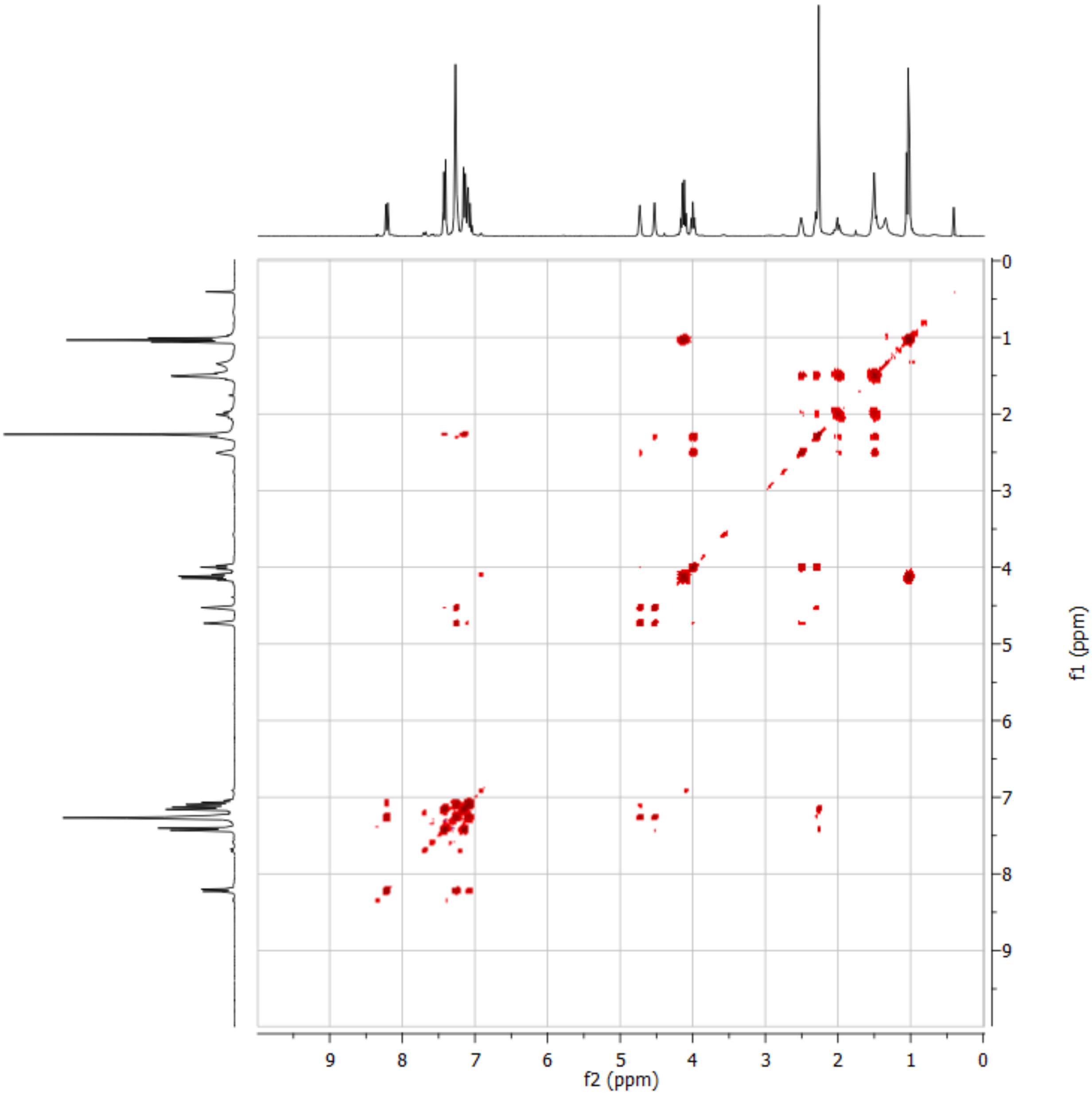

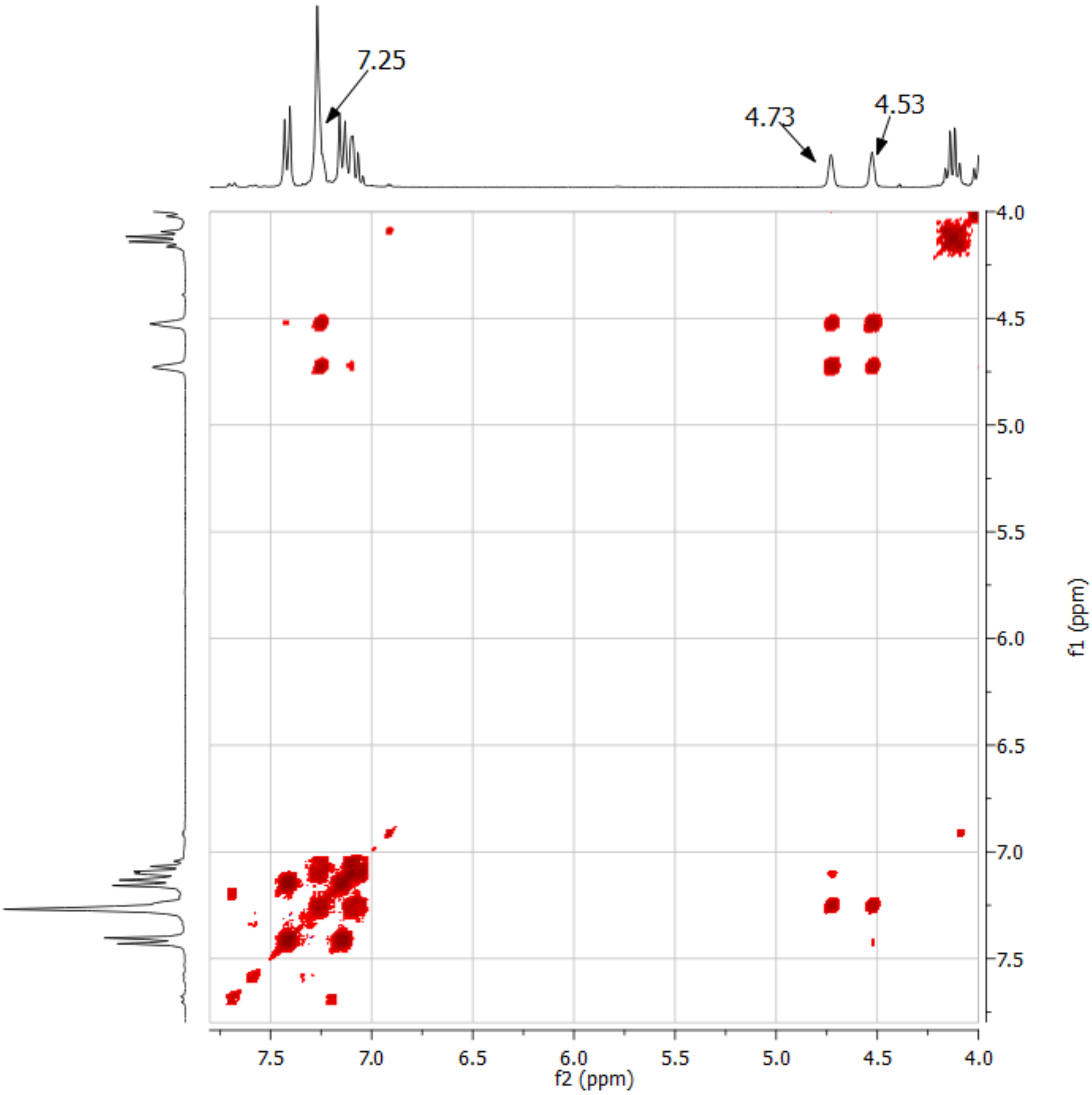
7.25
4.73
4.53
f2 (ppm)
f1 (ppm)
7.5
7.0
6.5
6.0
5.5
5.0
4.5
4.0

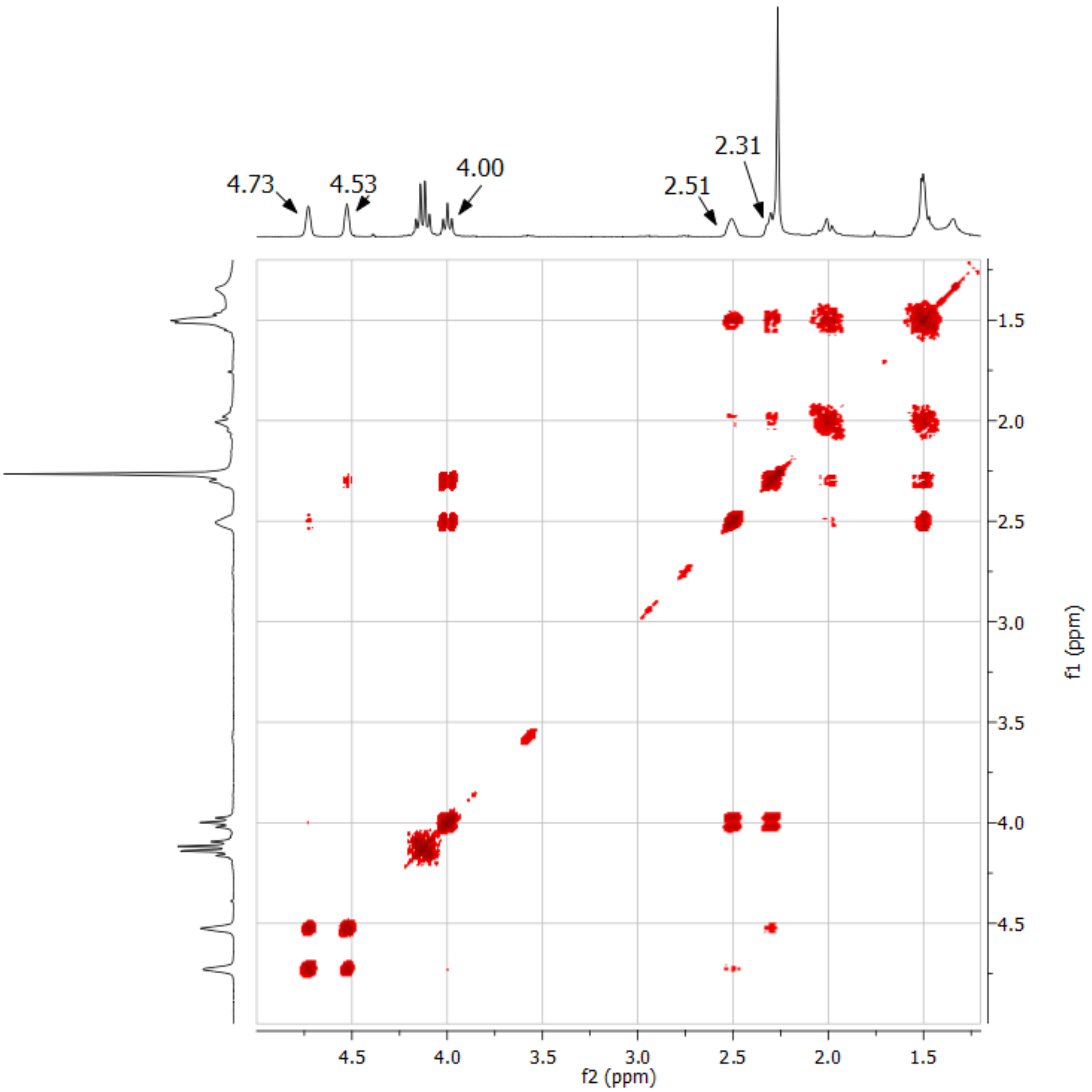
4.73
4.53
4.00
2.51
2.31
f1 (ppm)
f2 (ppm)

16, HSQC in C_6D_6 at T = 300 K

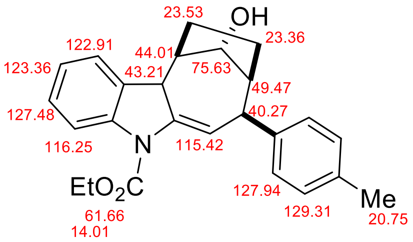

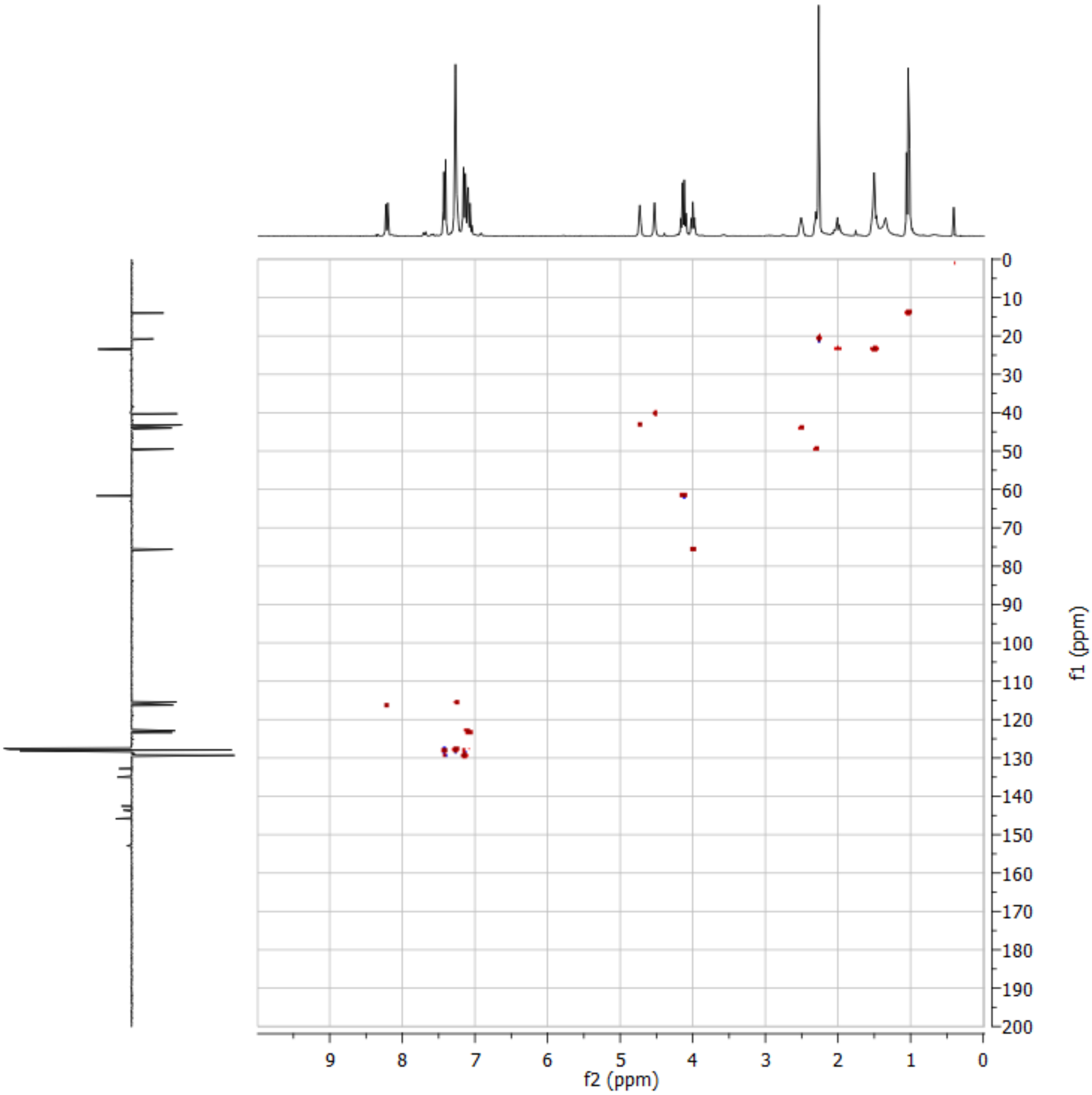

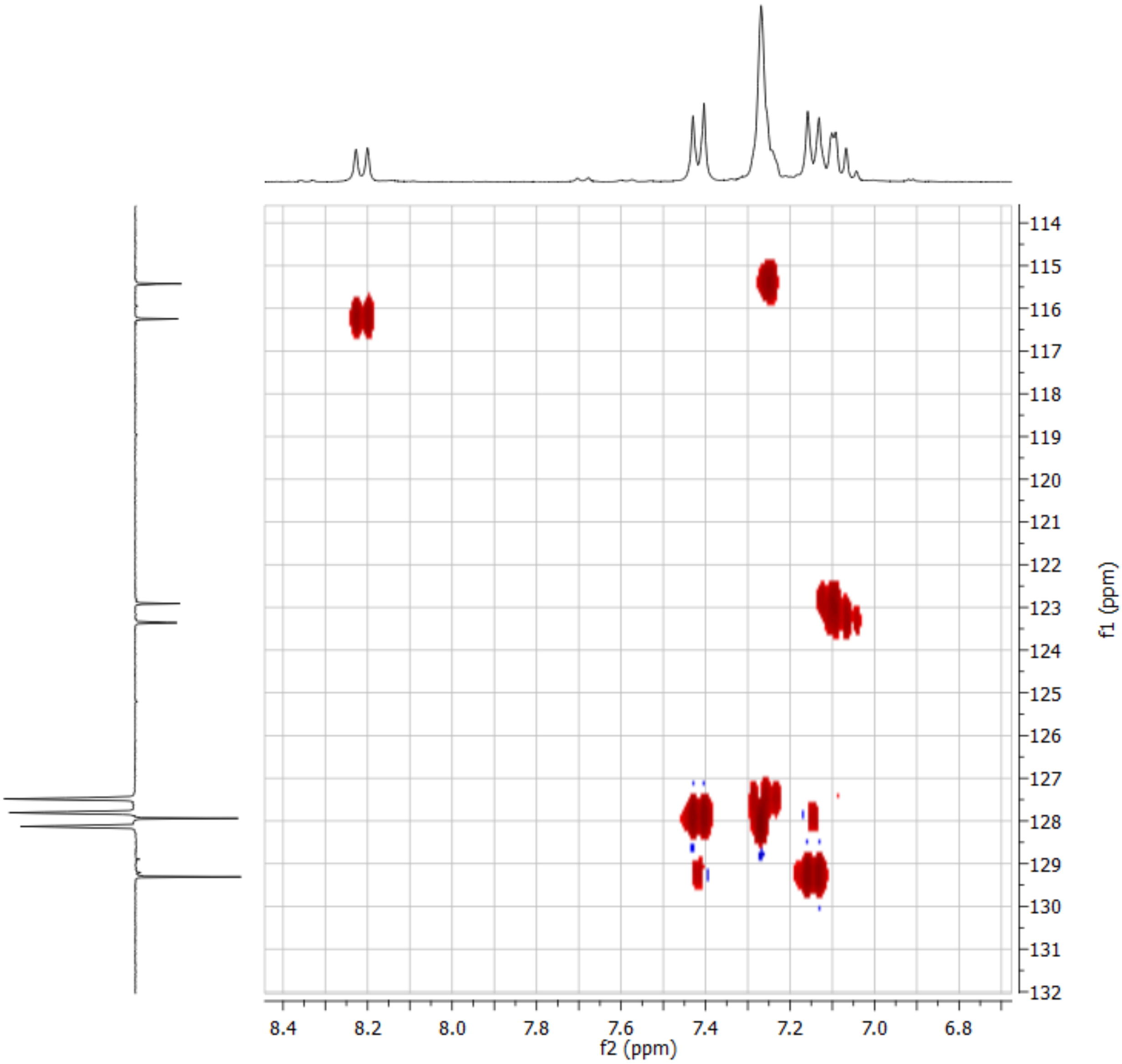
114
115
116
117
118
119
120
121
122
123
124
125
126
127
128
129
130
131
132
f1 (ppm)
8.4
8.2
8.0
7.8
7.6
7.4
7.2
7.0
6.8
f2 (ppm)

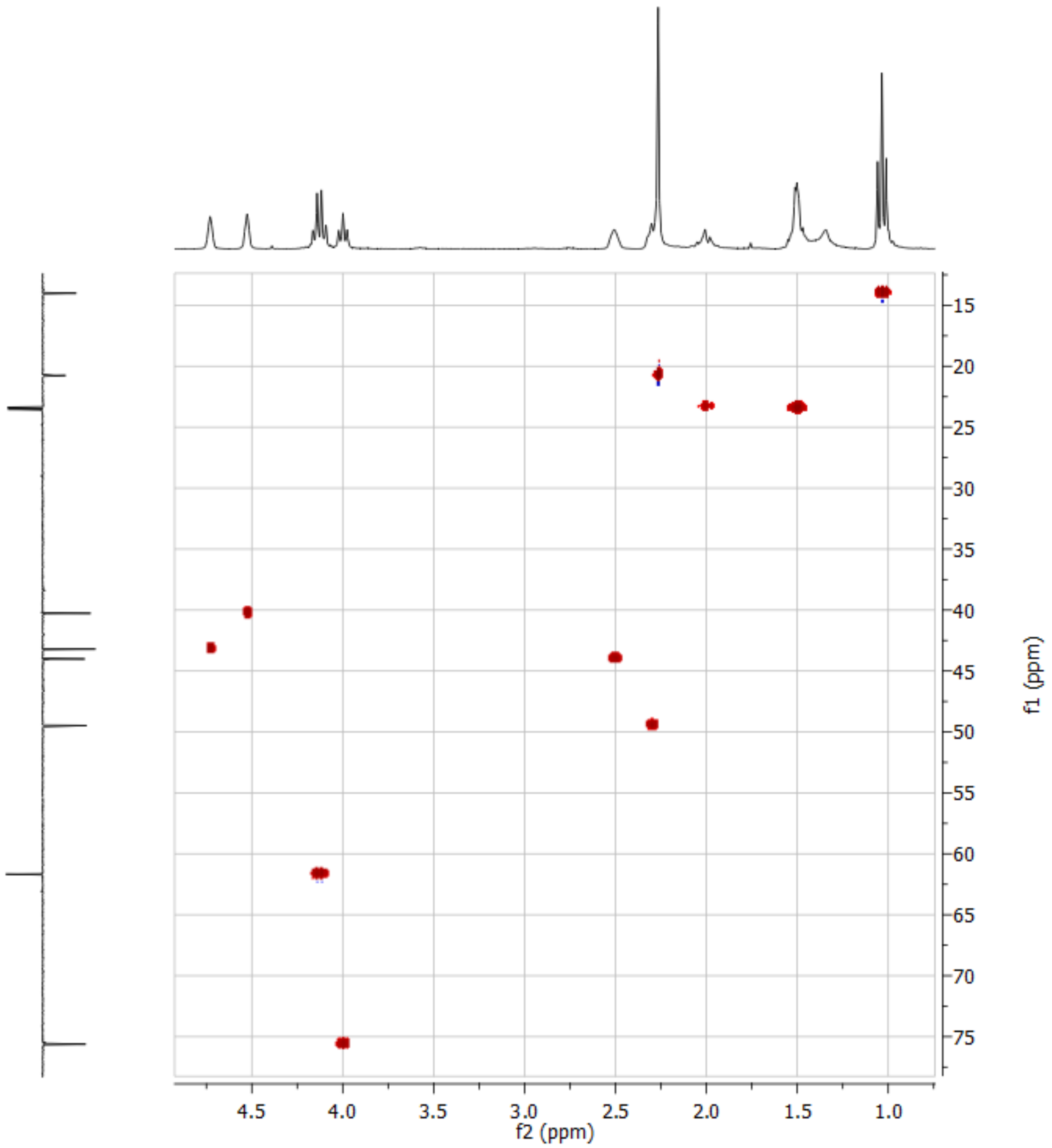

f1 (ppm)
f2 (ppm)

16, NOESY in C_6D_6 at T = 300 K

For a better understanding of the diagnostic NOESY interactions, beside a picture reporting diagnostic cross picks with the use of arrows, we report a view of a MM2 minimized 3D model of 1**6**.

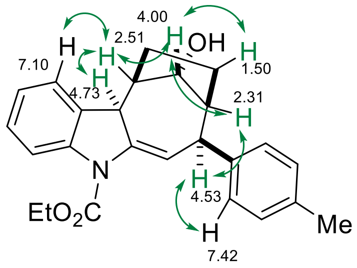

diagnostic cross picks

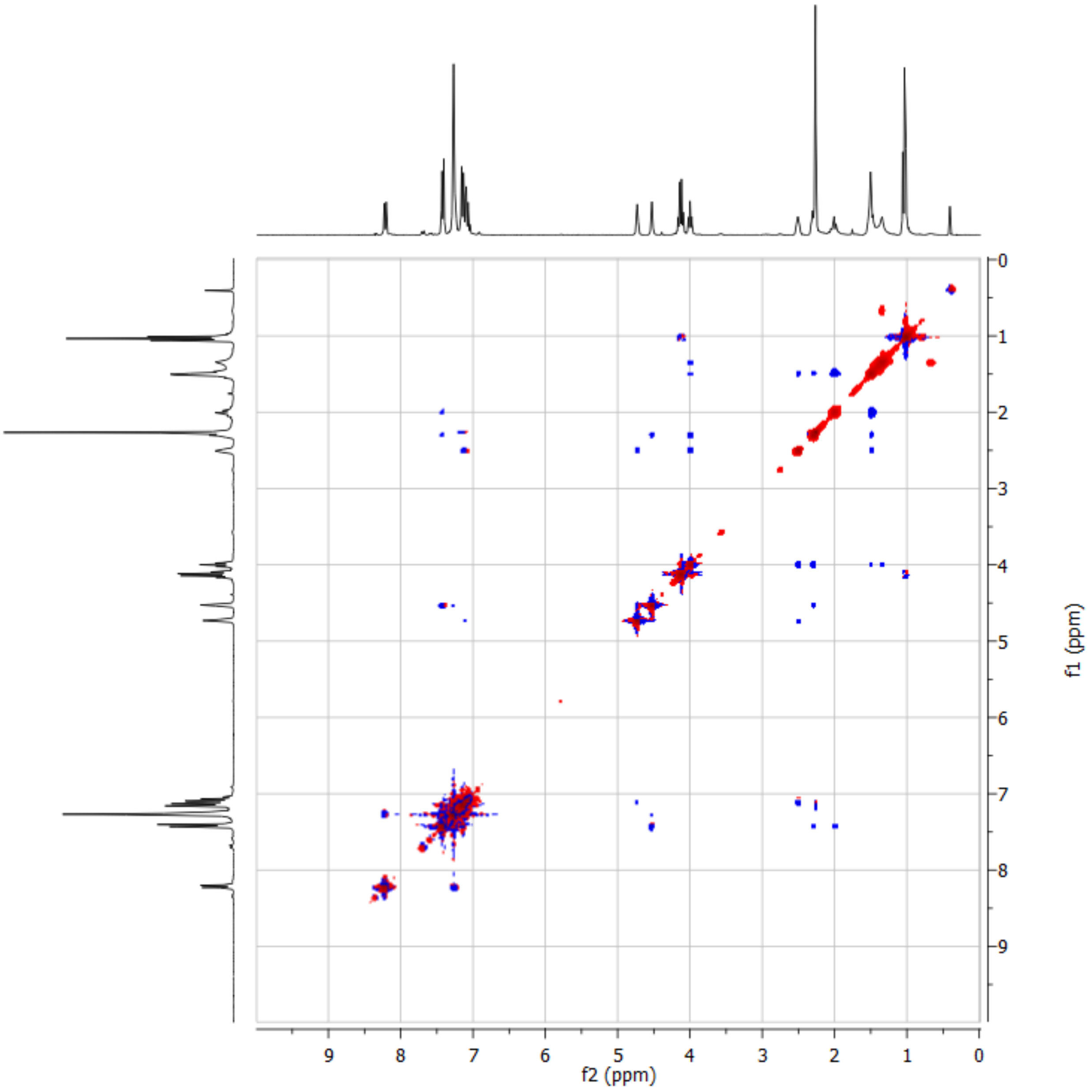

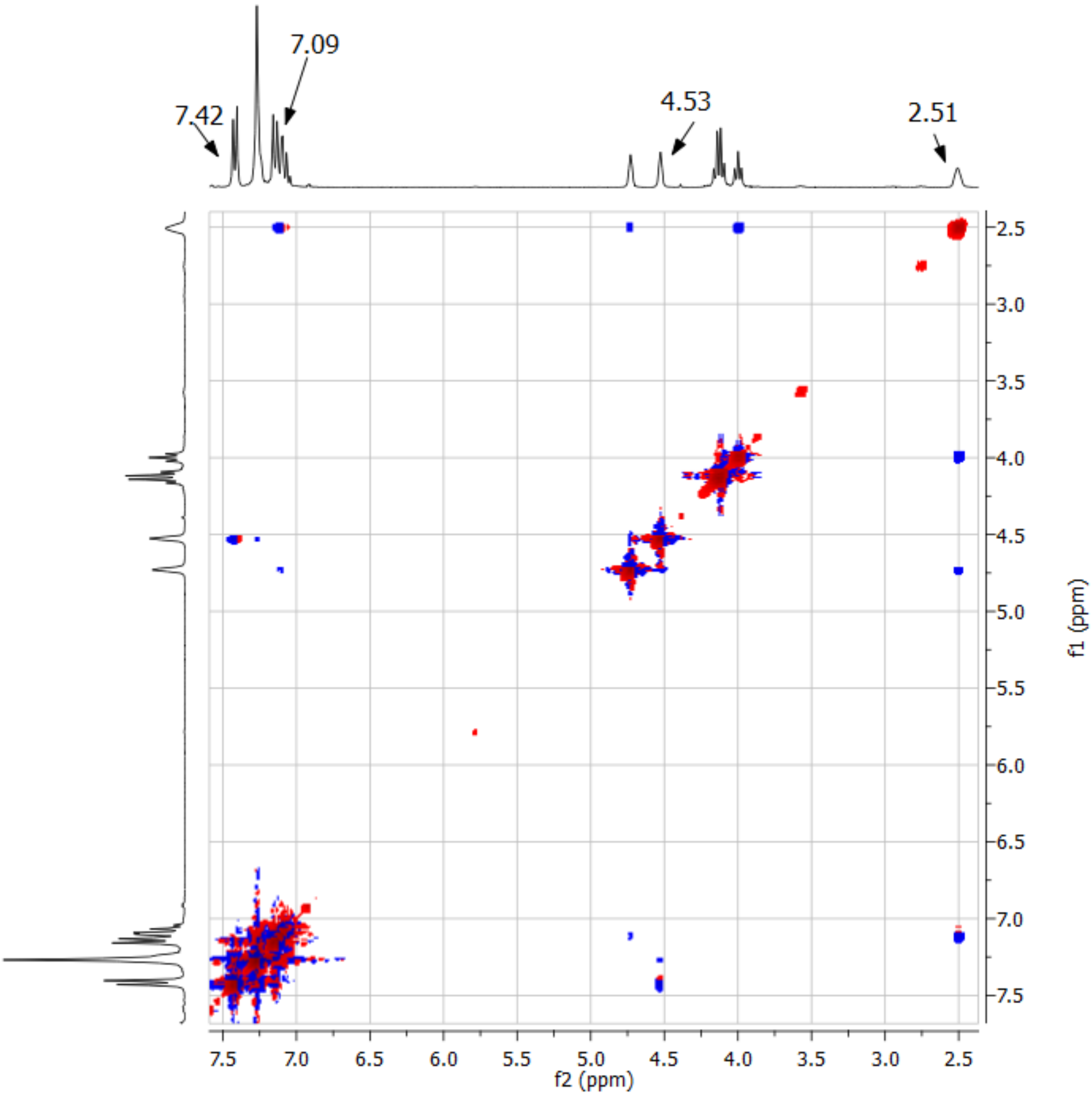

7.42
7.09
4.53
2.51
f2 (ppm)
f1 (ppm)

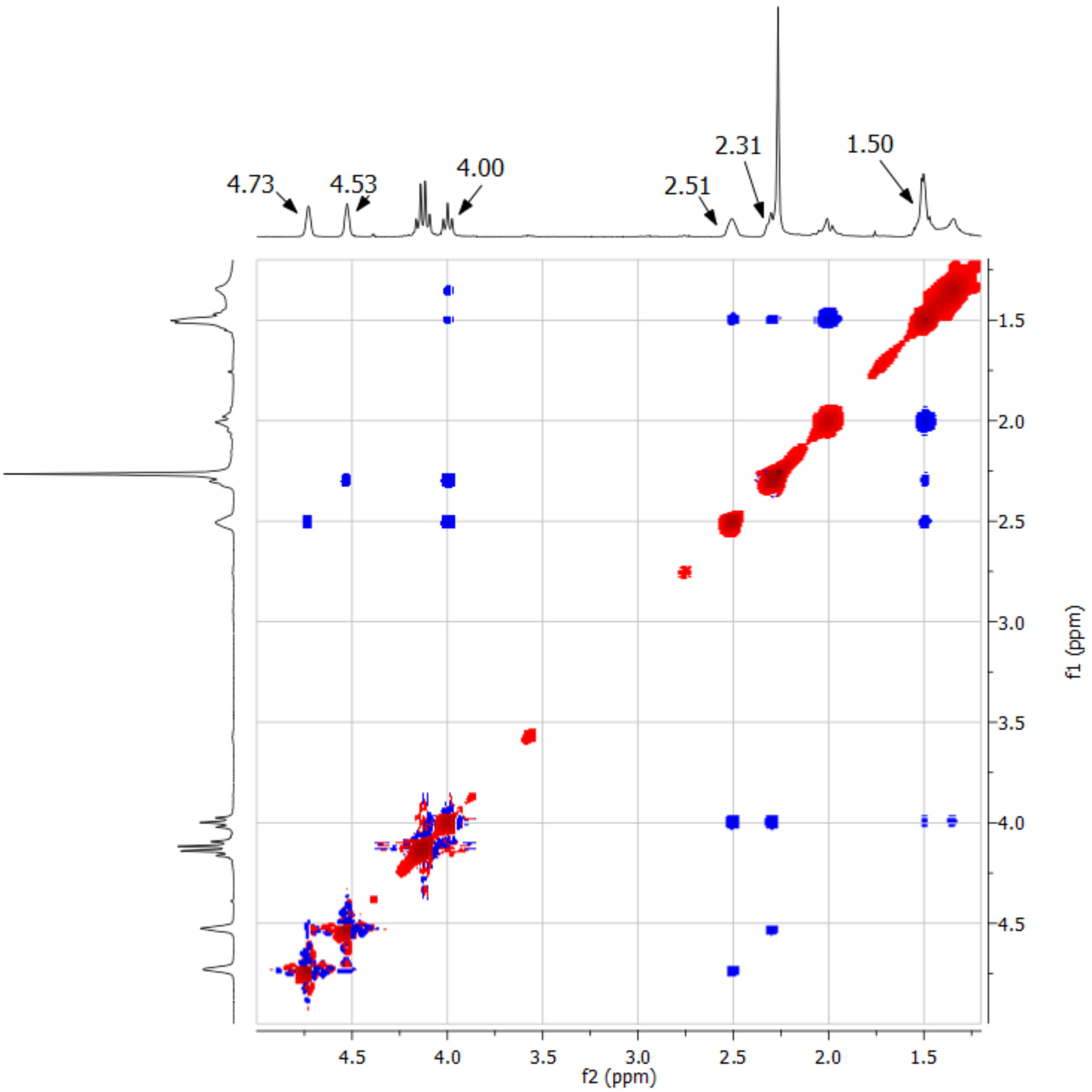
4.73
4.53
4.00
2.51
2.31
1.50
f2 (ppm)
f1 (ppm)
4.5
4.0
3.5
3.0
2.5
2.0
1.5

16, MM2 minimized 3D structure

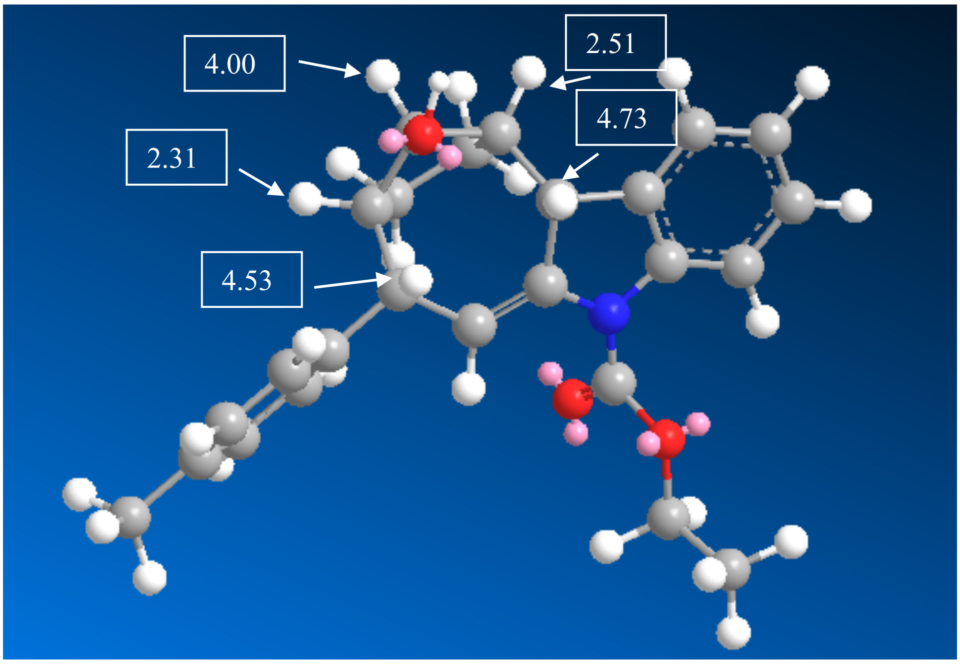

Figure S7: MM2 minimized 3D structure of **16.**

17, COSY in $CDCl_3$ at T = 300 K

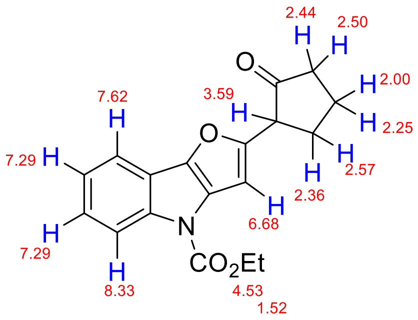

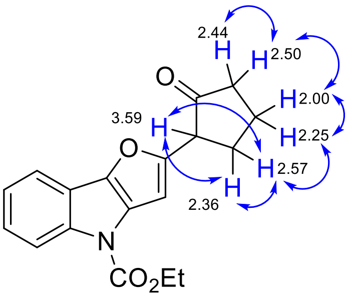

diagnostic cross picks

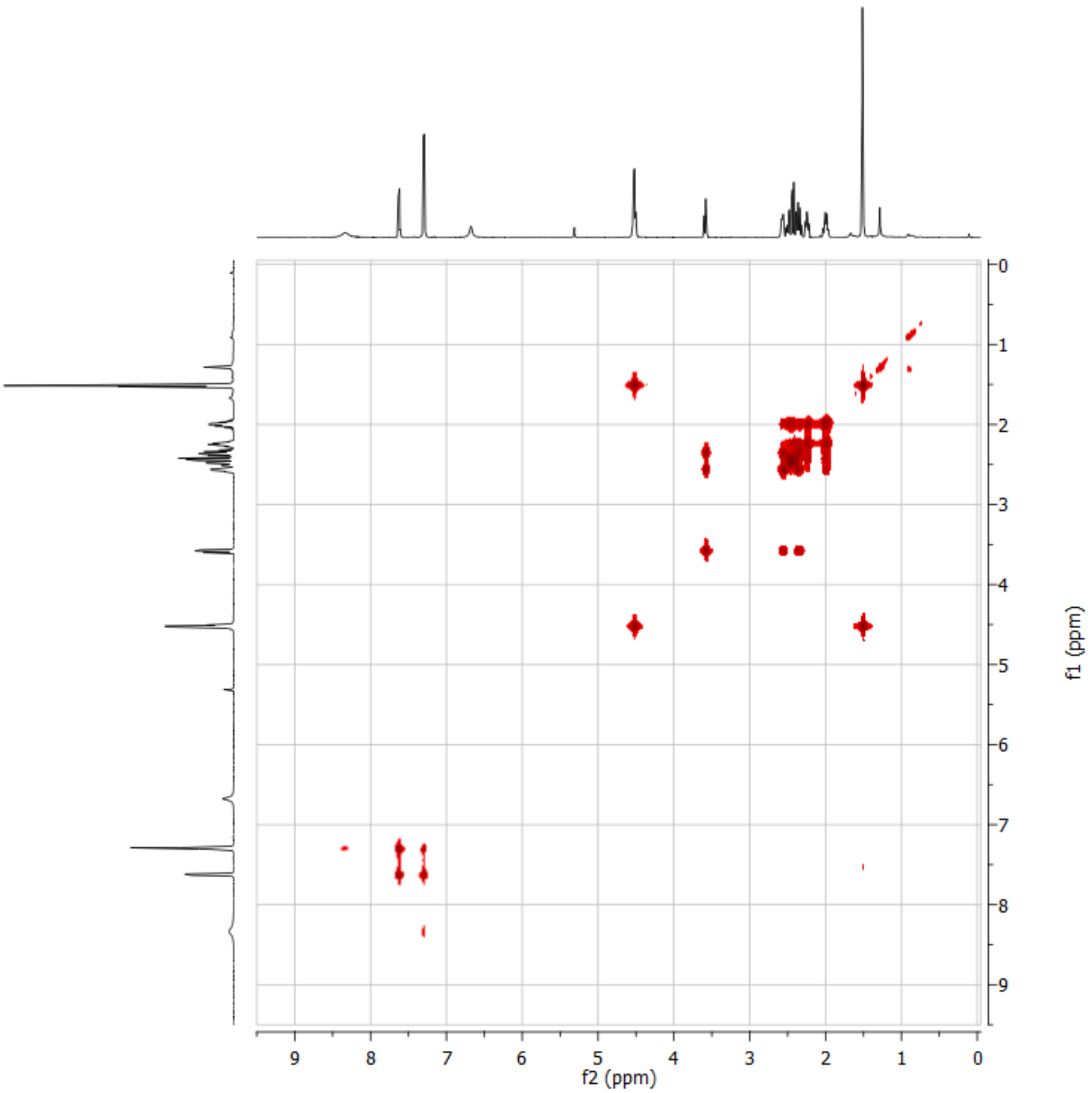

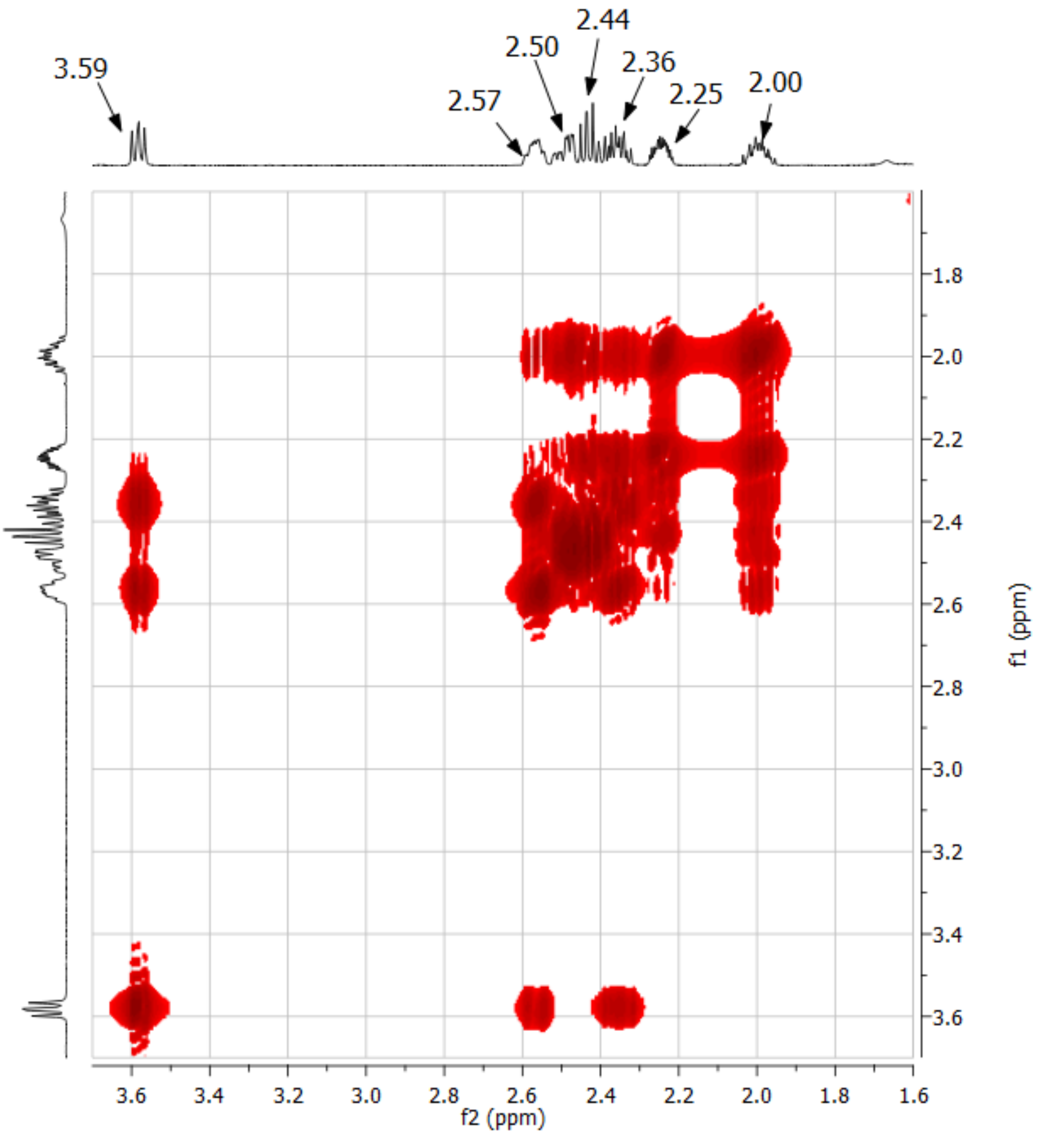
3.59
2.57
2.50
2.44
2.36
2.25
2.00
3.6
3.4
3.2
3.0
2.8
2.6
2.4
2.2
2.0
1.8
1.6
f2 (ppm)
1.8
2.0
2.2
2.4
2.6
2.8
3.0
3.2
3.4
3.6
f1 (ppm)

17, HSQC in $CDCl_3$ at T = 300 K

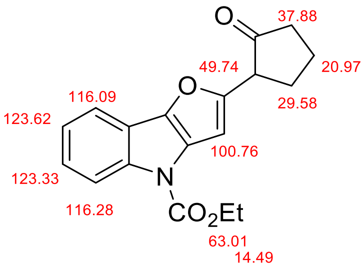

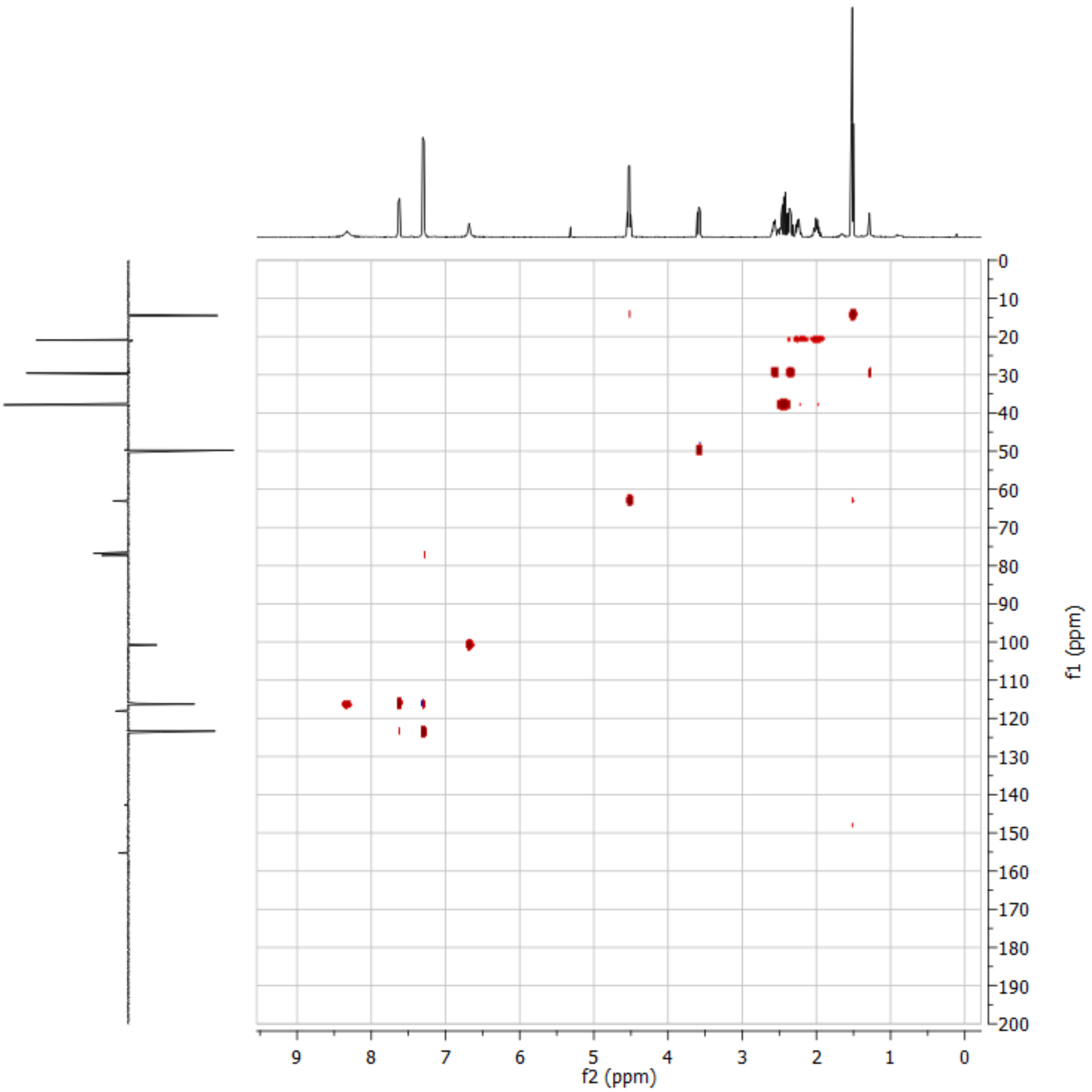

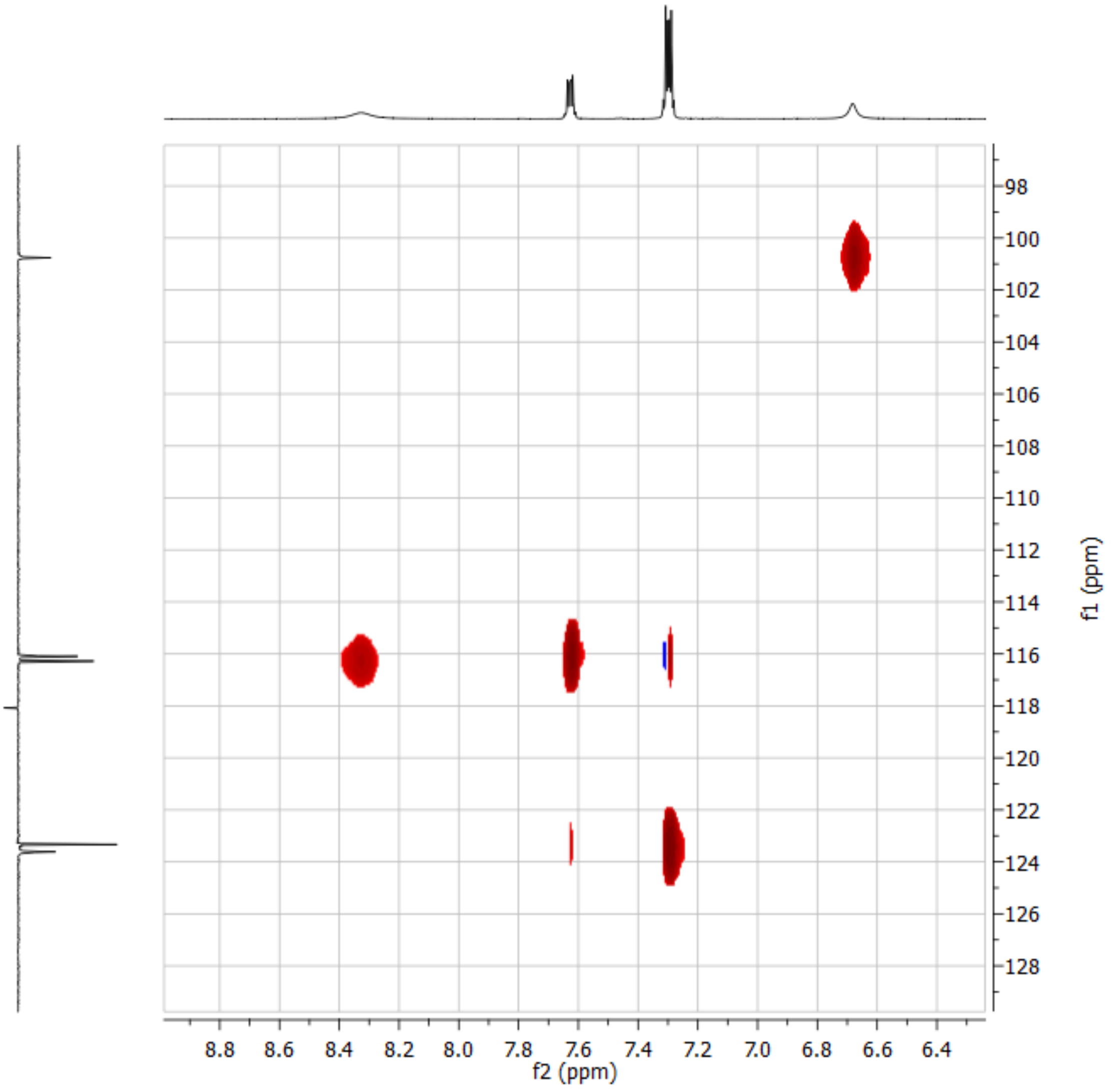
98
100
102
104
106
108
110
112
114
116
118
120
122
124
126
128
f1 (ppm)
8.8
8.6
8.4
8.2
8.0
7.8
7.6
7.4
7.2
7.0
6.8
6.6
6.4
f2 (ppm)

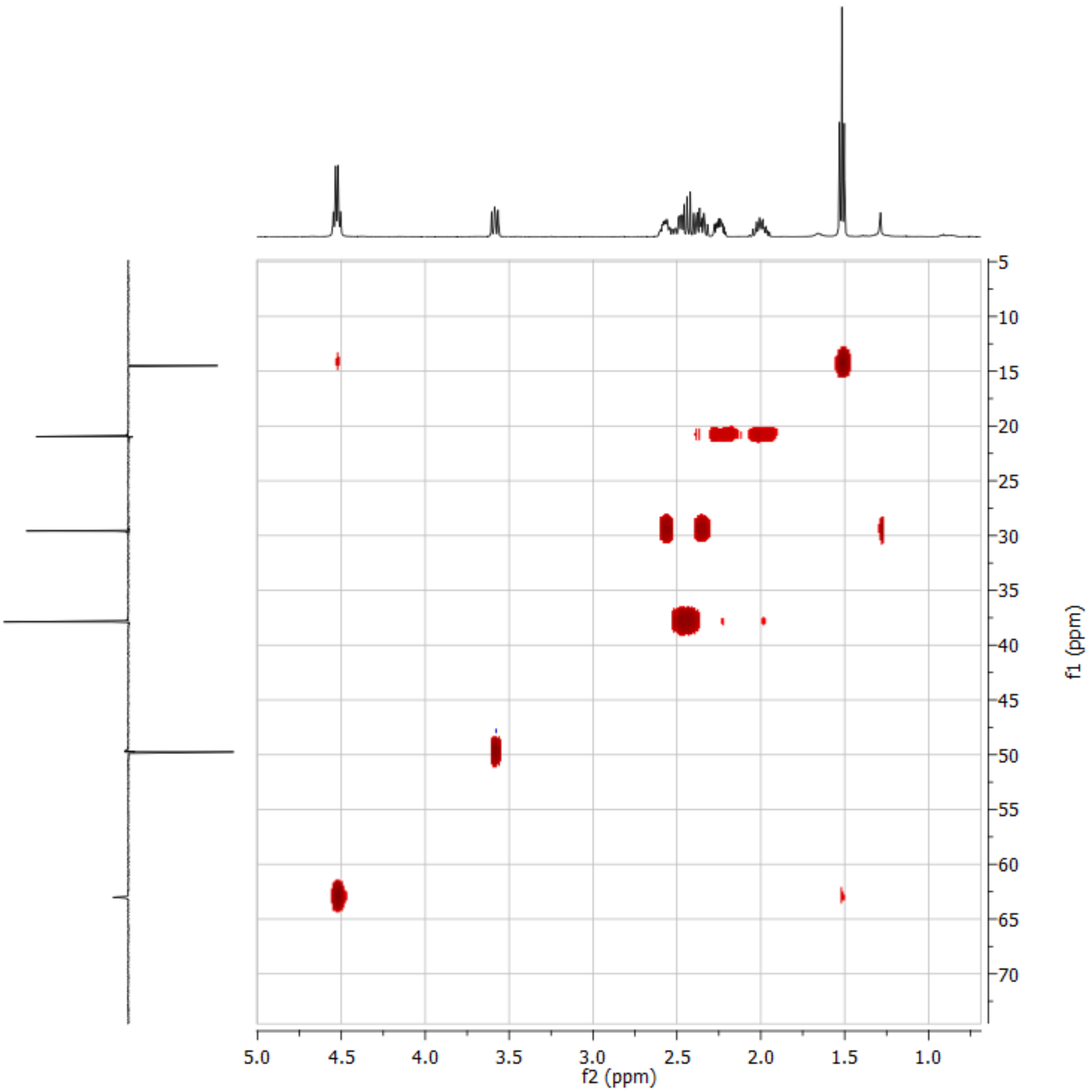

f2 (ppm)
f1 (ppm)

17, NOESY in $CDCl_3$ at T = 300 K

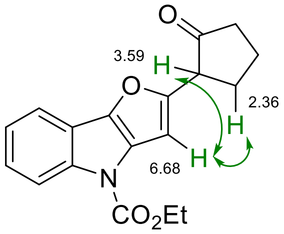

diagnostic cross picks

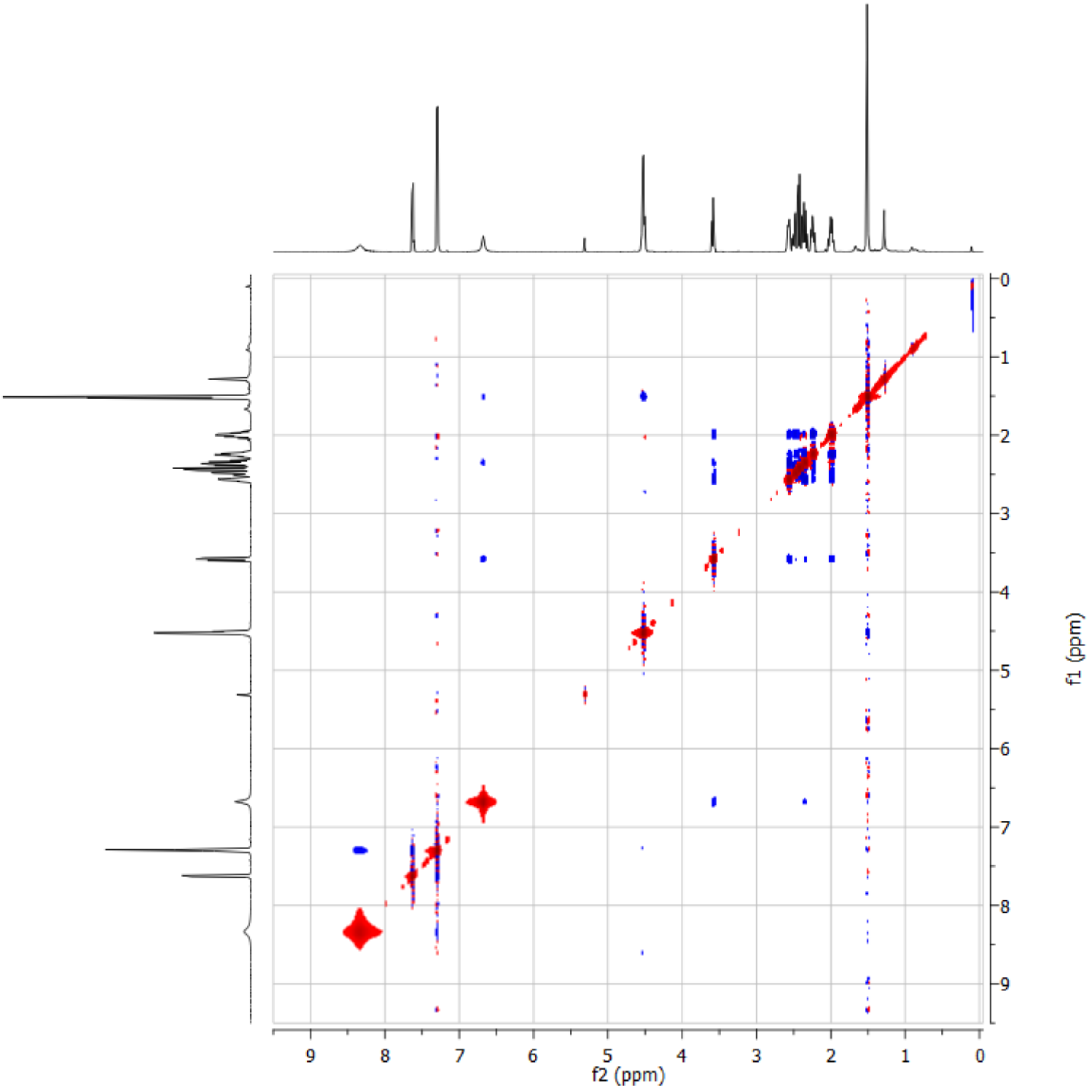

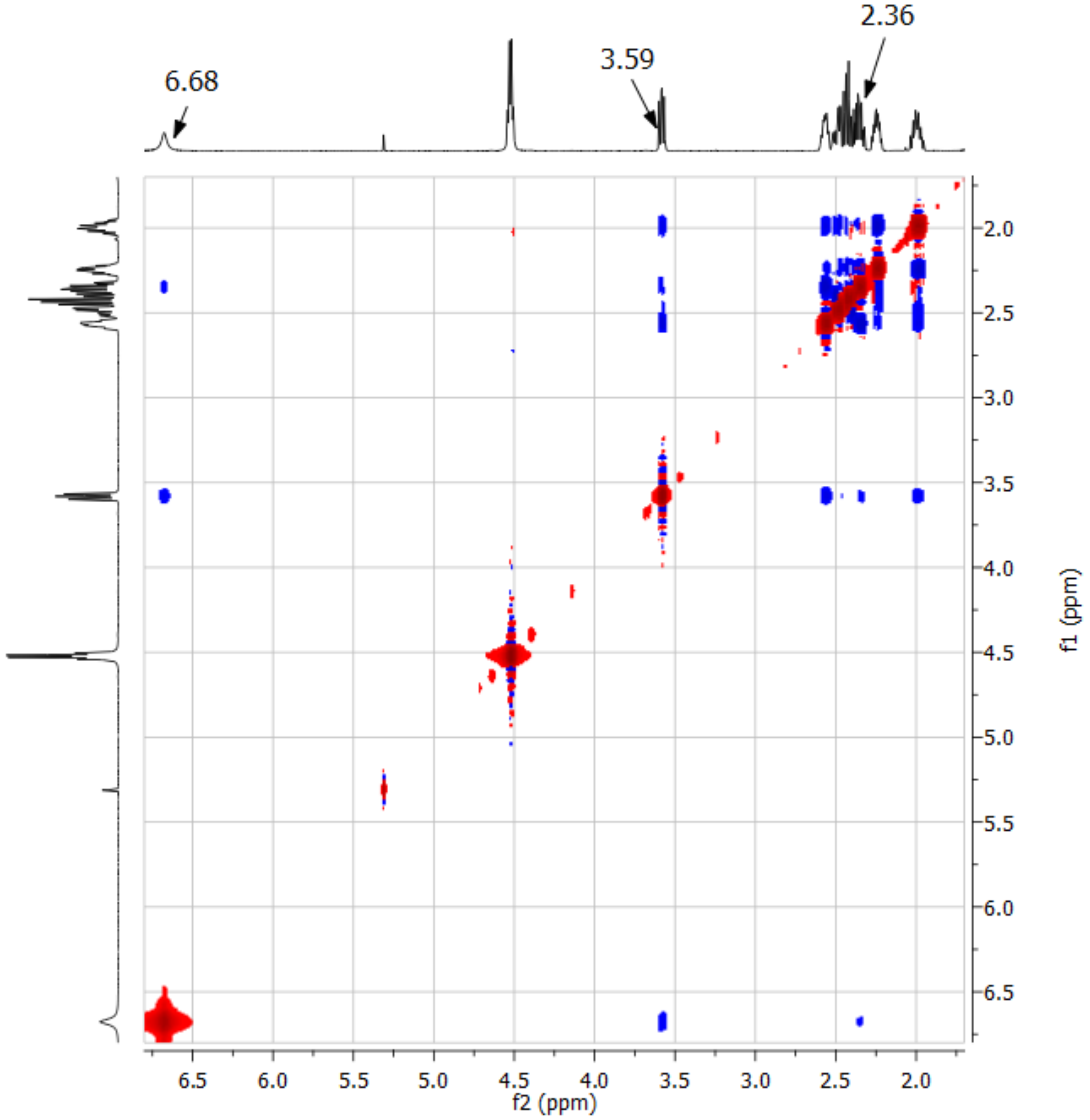

6.68
3.59
2.36
f2 (ppm)
f1 (ppm)

Crystallographic data for 6a

Crystal structure determinations.

Single crystals of 6a suitable for X-ray diffraction were grown by *slow evaporation* at low temperature (5°C) from ethyl acetate. A selected crystal (approximate dimensions 0.48 x 0.42 x 0.26 mm^3) was placed onto the tip of a glass capillary and mounted on a Bruker SMART-APEX II diffractometer for a data collection at 293(2) K. A preliminary orientation matrix and unit cell parameters were obtained from reflections harvested from three sets of 12 frames each, collected with a frame time of 20 seconds.

The X-ray data collection at 293 K was carried out using graphite-monochromated MoKα radiation (λ = 0.71073 Å) with a frame time of 10 seconds and a detector distance of 50.9 mm. Four set of frames were collected with 0.50° steps in ω at four different φ settings and a detector position of -30° in 2θ. The intensity data were corrected for absorption by using SADABS.[1] No *decay correction* was applied. Determination of the integrated intensities and unit cell refinement were performed using the SAINT[1] program. The structure was solved by direct methods (SIR2004)[2] and refined by full-matrix least squares on F^2 against all data (SHELX 2014)[3] with the WINGX interface.[4]

All hydrogens were found from difference Fourier synthesis and refined as "riding" on the adjacent carbon with individual isotropic temperature factor 1.2 or 1.5 (H-methyl group) times the value of the equivalent temperature factor of the parent atom. Non-H atoms were refined with full occupancy and anisotropic displacement parameters.

The diagram was drawn using ORTEPIII program.[5] Crystal data and structure determination results are summarized in Table S1.

Full crystallographic data have been deposited with the Cambridge Crystallographic Data Centre (CCDC No. 1964975). A copy of the data can be obtained free of charge on application to CCDC, 12 Union Road, Cambridge CB2 IEZ, UK (Fax: +44 1223 336 033; e-mail: deposit@ccdc.cam.ac.uk).

1. Bruker. *SADABS* and *SAINT* **2009**, Bruker AXS Inc., Madison, Wisconsin, USA.
2. Burla, M. C.; Caliandro, R.; Camalli, M.; Carrozzini, B.; Cascarano, G. L.; De Caro, L.; Giacovazzo, C.; Polidori, G.; Spagna, R. *J. Appl. Crystallogr.* **2005**, *38* (2), 381–388.
3. Sheldrick, G. M. *Acta Crystallogr. Sect. C Struct. Chem.* **2015**, *71*, 3–8.
4. Farrugia, L. J. *J. Appl. Crystallogr.* **2012**, *45* (4), 849–854.
5. M. N. Burnett and C. K. Johnson, ORTEP-III Report ORNL-6895. Oak Ridge National Laboratory, Tennessee, USA, 1996.

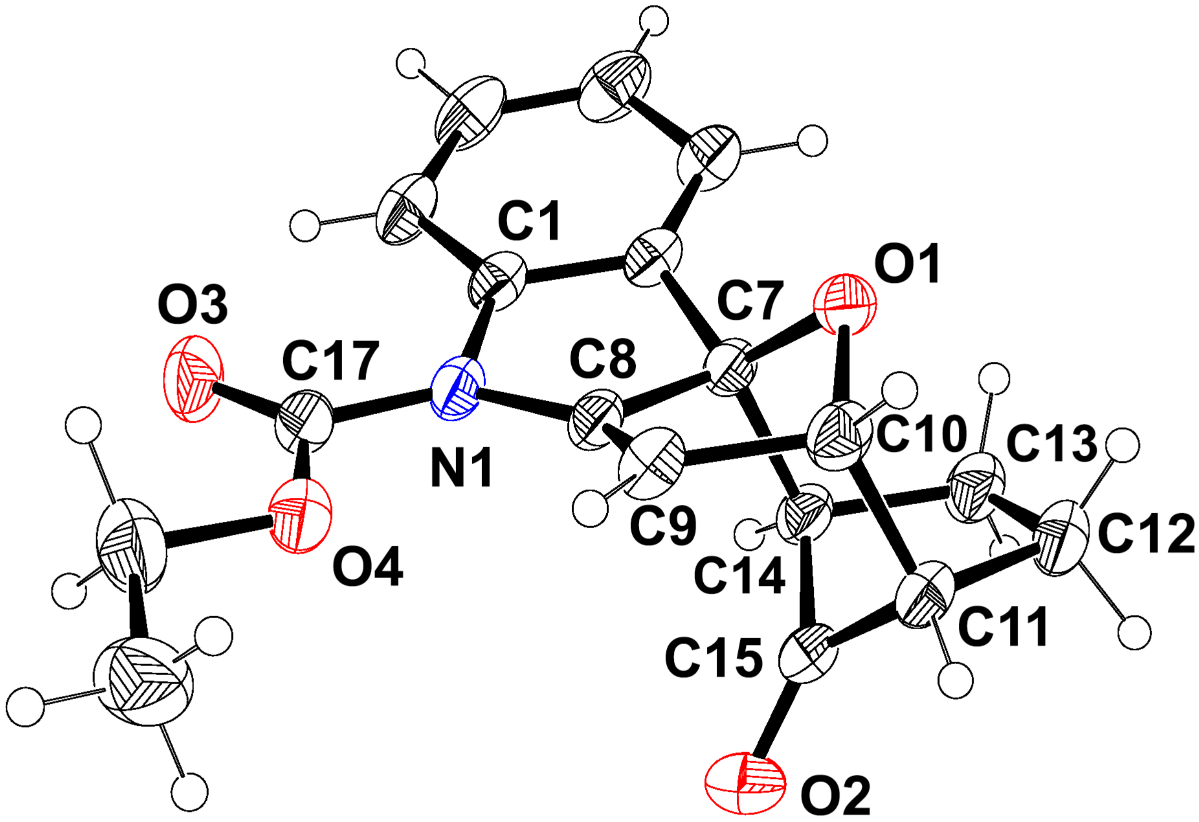

Figure S8: The molecular structure of compound **6a** showing the numbering schemes for some nonhydrogen atoms. Displacement ellipsoids are plotted at the 40% probability level.

Table S1. Summary of X-ray single crystal diffraction refinement results for **6a**.

Identification code	**6a**
Empirical formula	$C_{18}H_{17}NO_4$
Formula weight	311.33
Temperature (K)	296(2)
Wavelength (Å)	0.71073
Crystal system	Monoclinic
Space group	*C*2/*c*
Unit cell dimensions	*a* = 15.549 (8) Å
	b = 11.015 (6) Å β = 95.236 (8)°
	c = 17.360 (9) Å
Volume (Å^3)	2961(3)
Z	8
Density (calculated) (g/cm^3)	1.3969
Absorption coefficient μ (mm^{-1})	0.099
F(000)	1312
T_{min}, T_{max}	0.660, 0.746
Theta range for data collection	2.3 to 31.6°
Index ranges	-21<=h<=22, -16<=k<=16, -25<=l<=24
(sin θ/λ)$_{max}$ (Å^{-1})	0.737
Reflections collected	21326
Independent reflections	4584 [R_{int} = 0.023]
Observed reflections [I>2sigma(I)]	3512
Completeness	99.6%
Data / restraints / parameters	4584 / 0 / 260

Goodness-of-fit on F^2	1.056
Final R indices [I>2sigma(I)]	R_1 = 0.0469, wR_2 = 0.1252
R indices (all data)	R_1 = 0.0643, wR_2 = 0.1399
Largest diff. peak and hole (e.Å^{-3})	0.297 and -0.241

^{1}H NMR (300 MHz, C_6D_6)

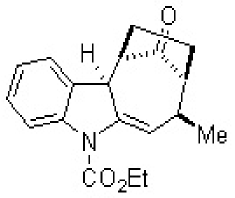

3a

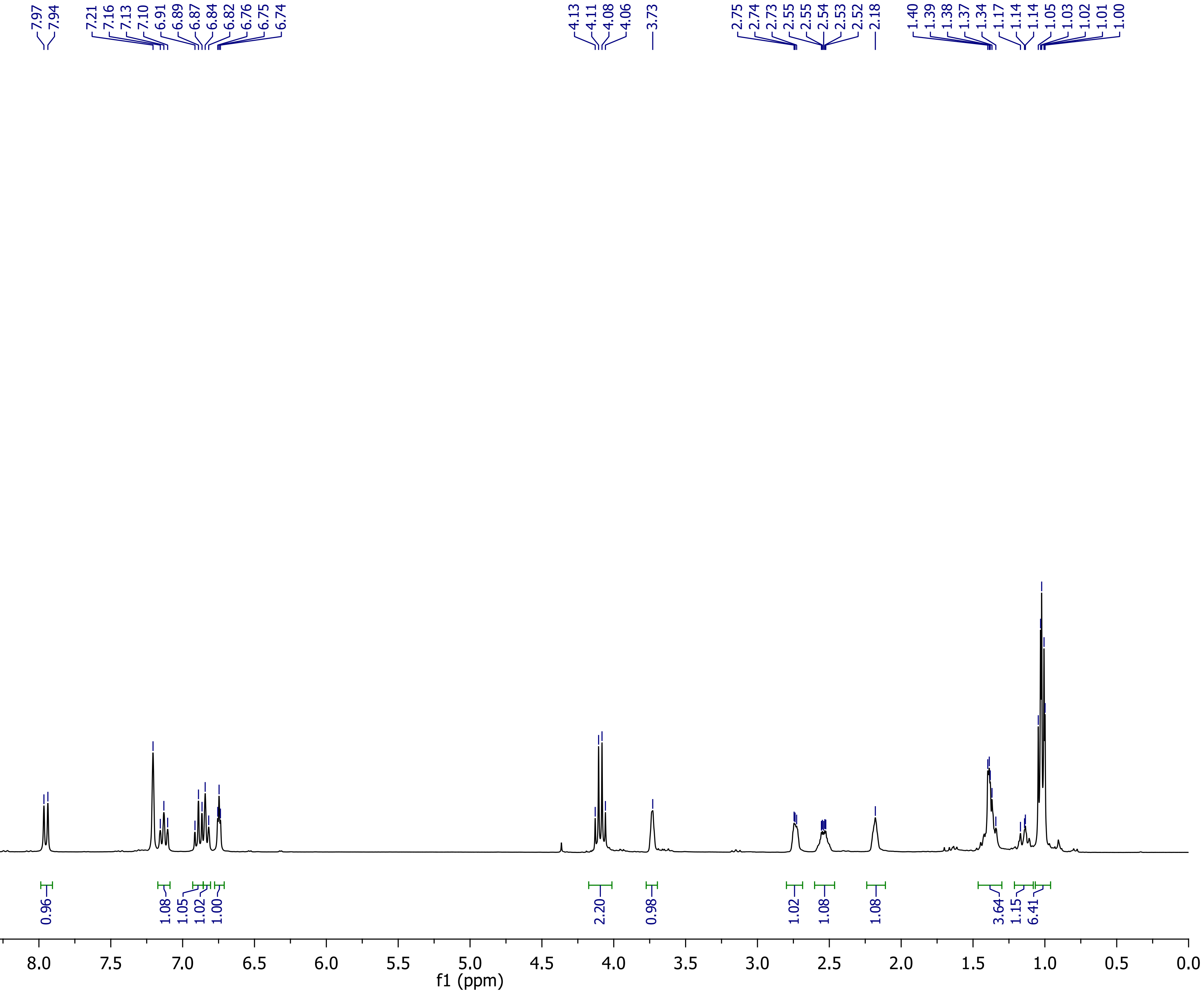

^{13}C NMR (126 MHz, C_6D_6)

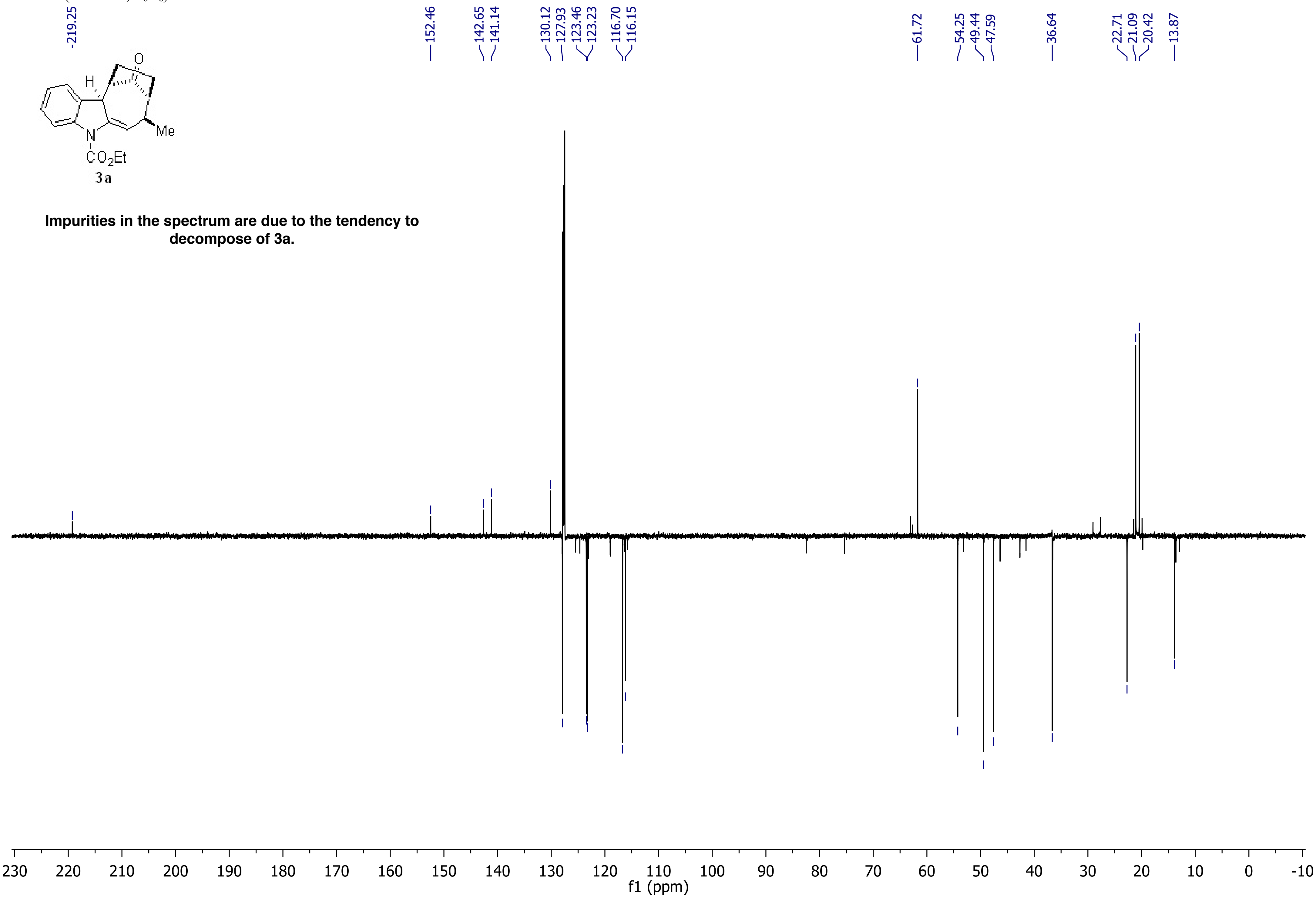

3a

Impurities in the spectrum are due to the tendency to decompose of 3a.

^{1}H NMR (300 MHz, $CDCl_3$)

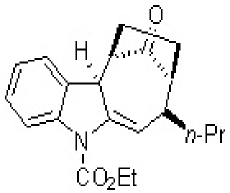

3b

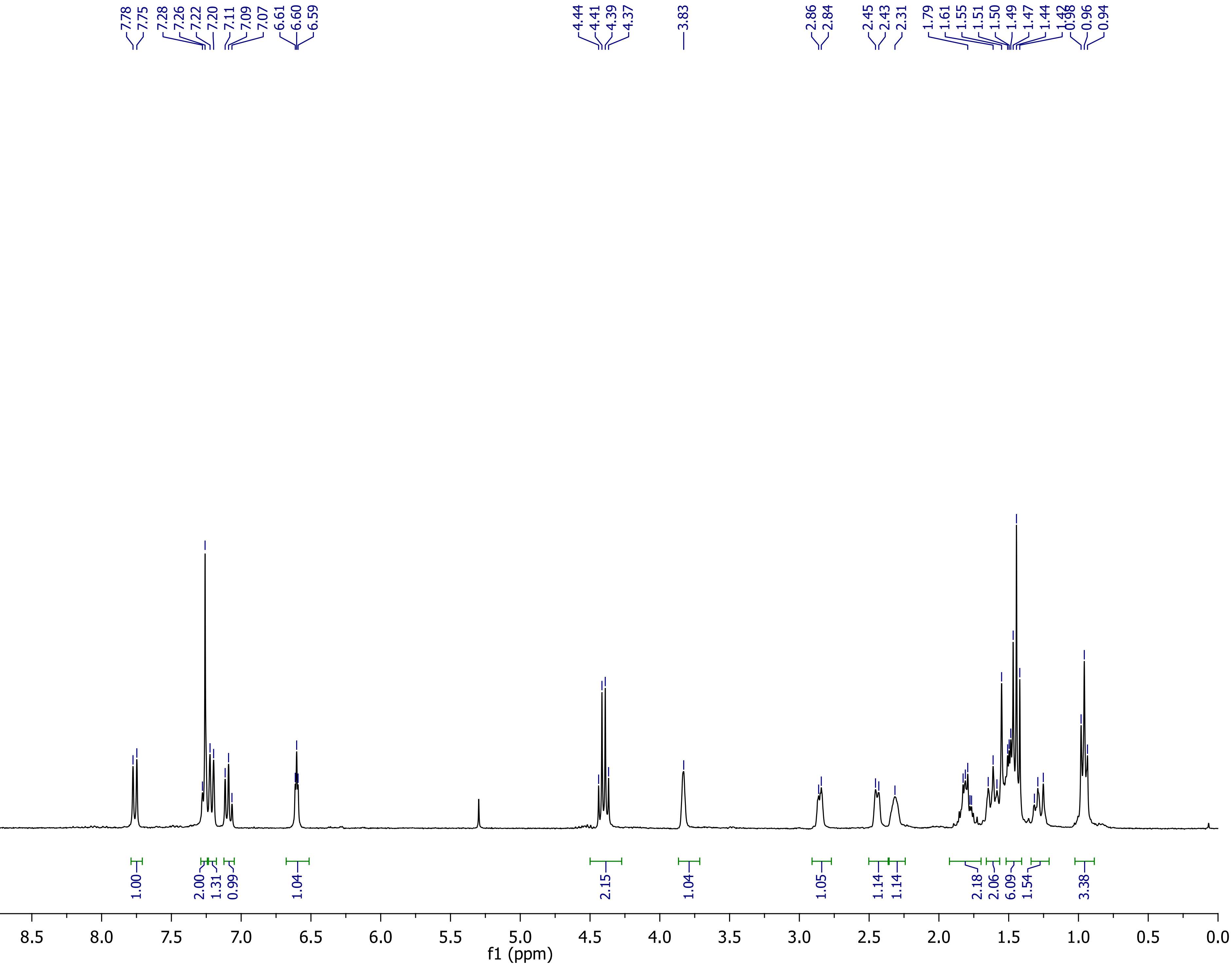

^{13}C NMR (126 MHz, C_6D_6)

3b

Impurities in the spectrum are due to the tendency to decompose of 3b.

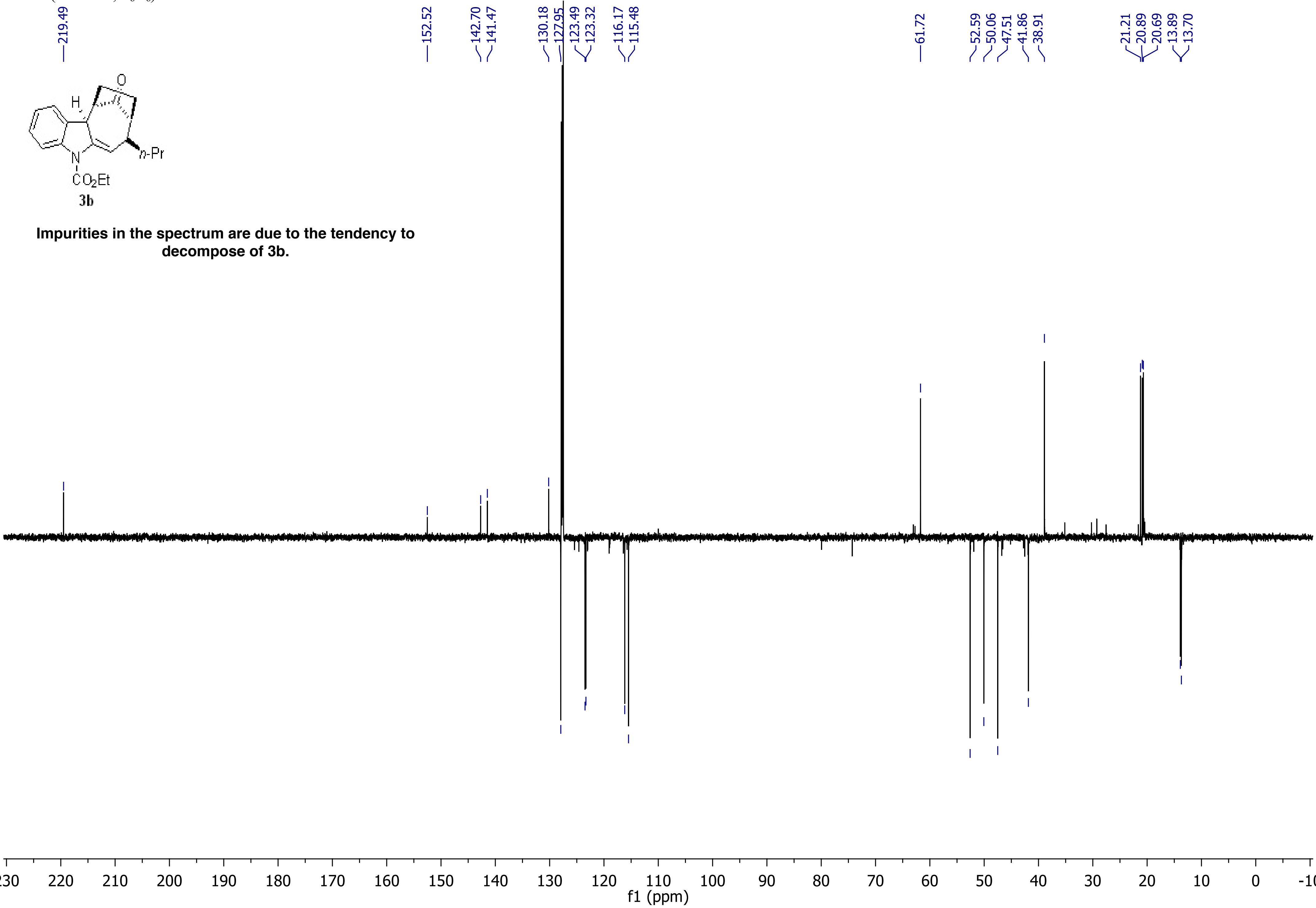

^{1}H NMR (500 MHz, C_6D_6)

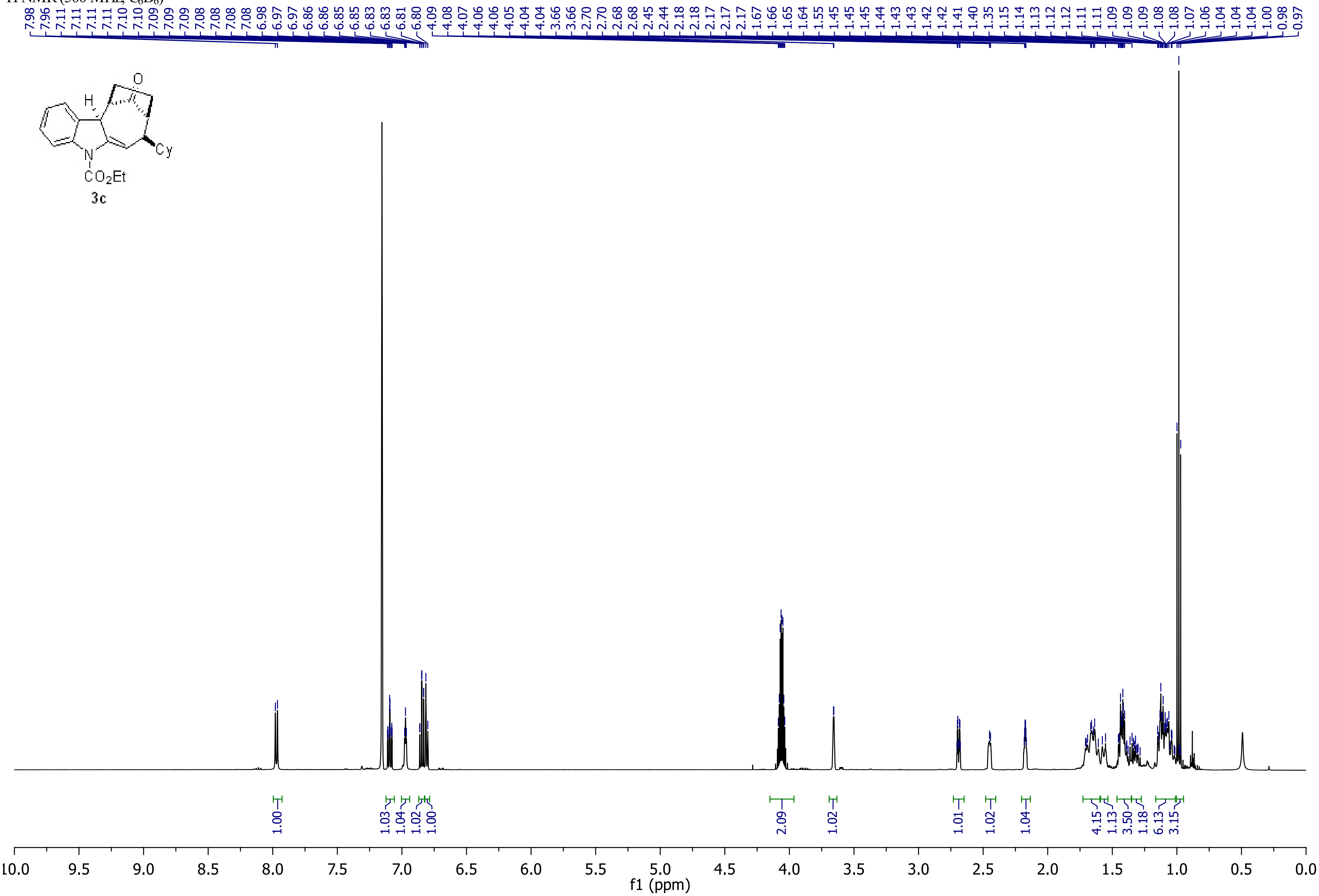

^{13}C NMR (126 MHz, C_6D_6)

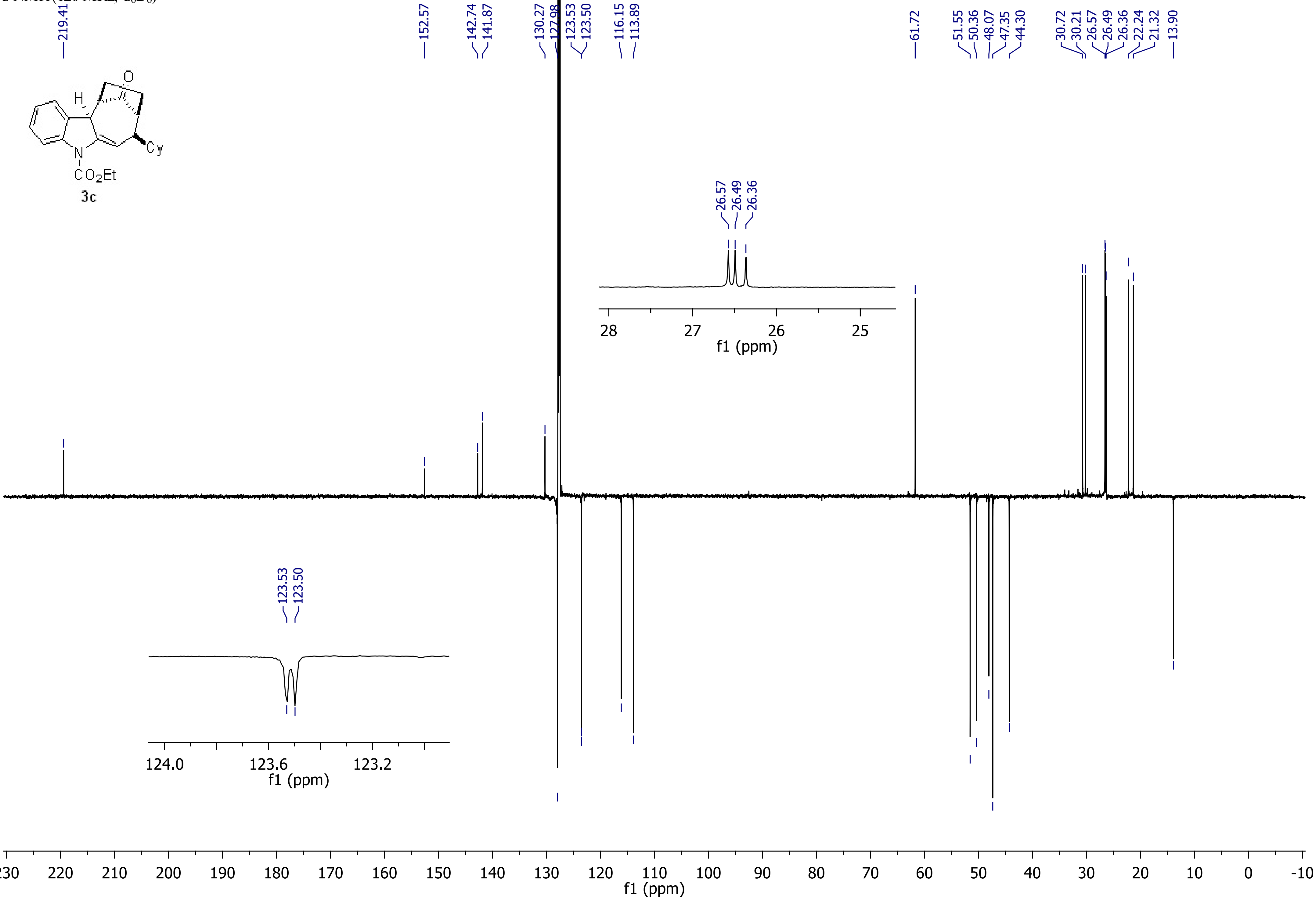

3c

^{1}H NMR (300 MHz, C_6D_6)

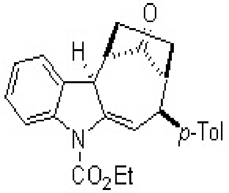

3d

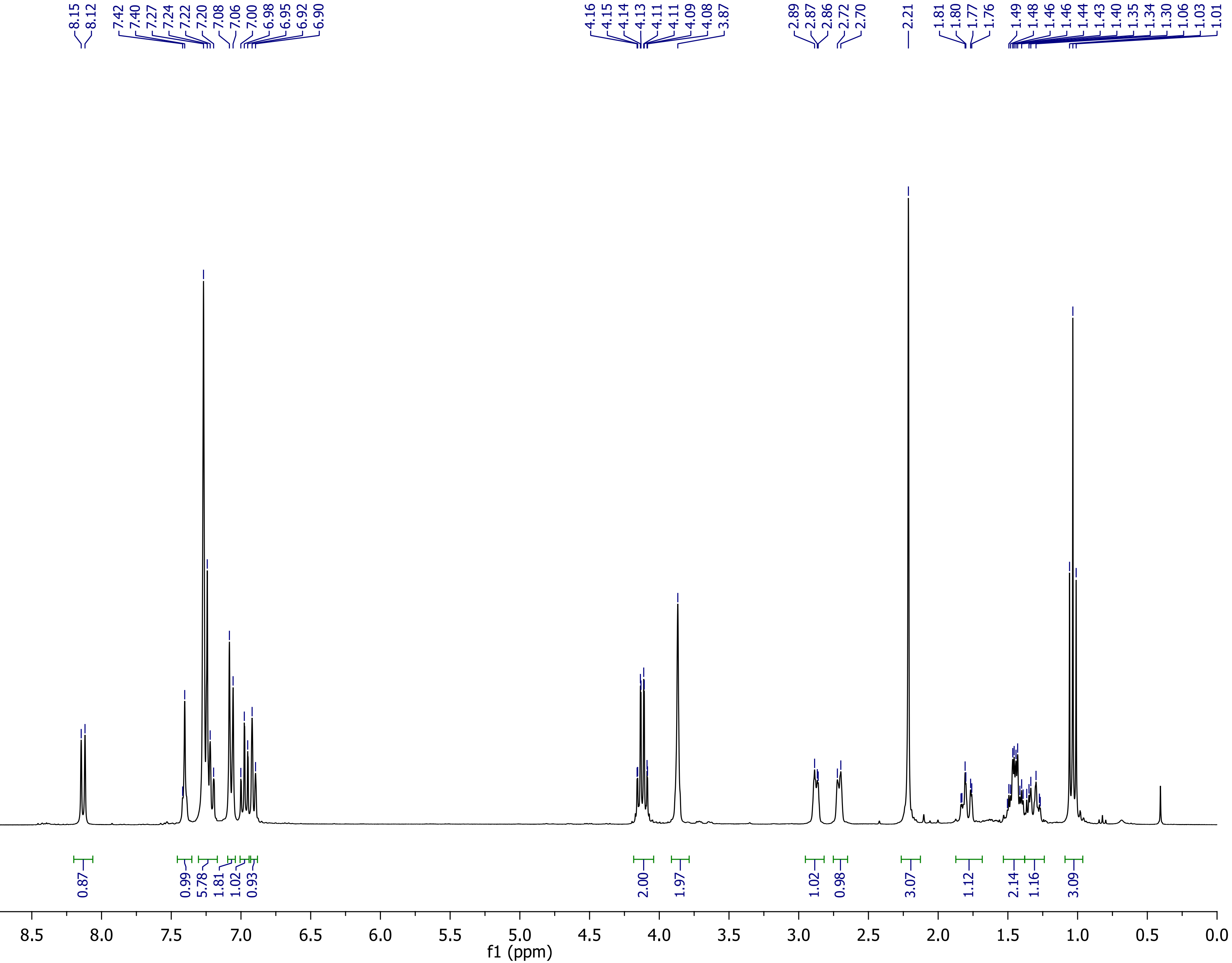

^{13}C NMR (75 MHz, C_6D_6)

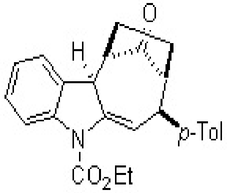

3d

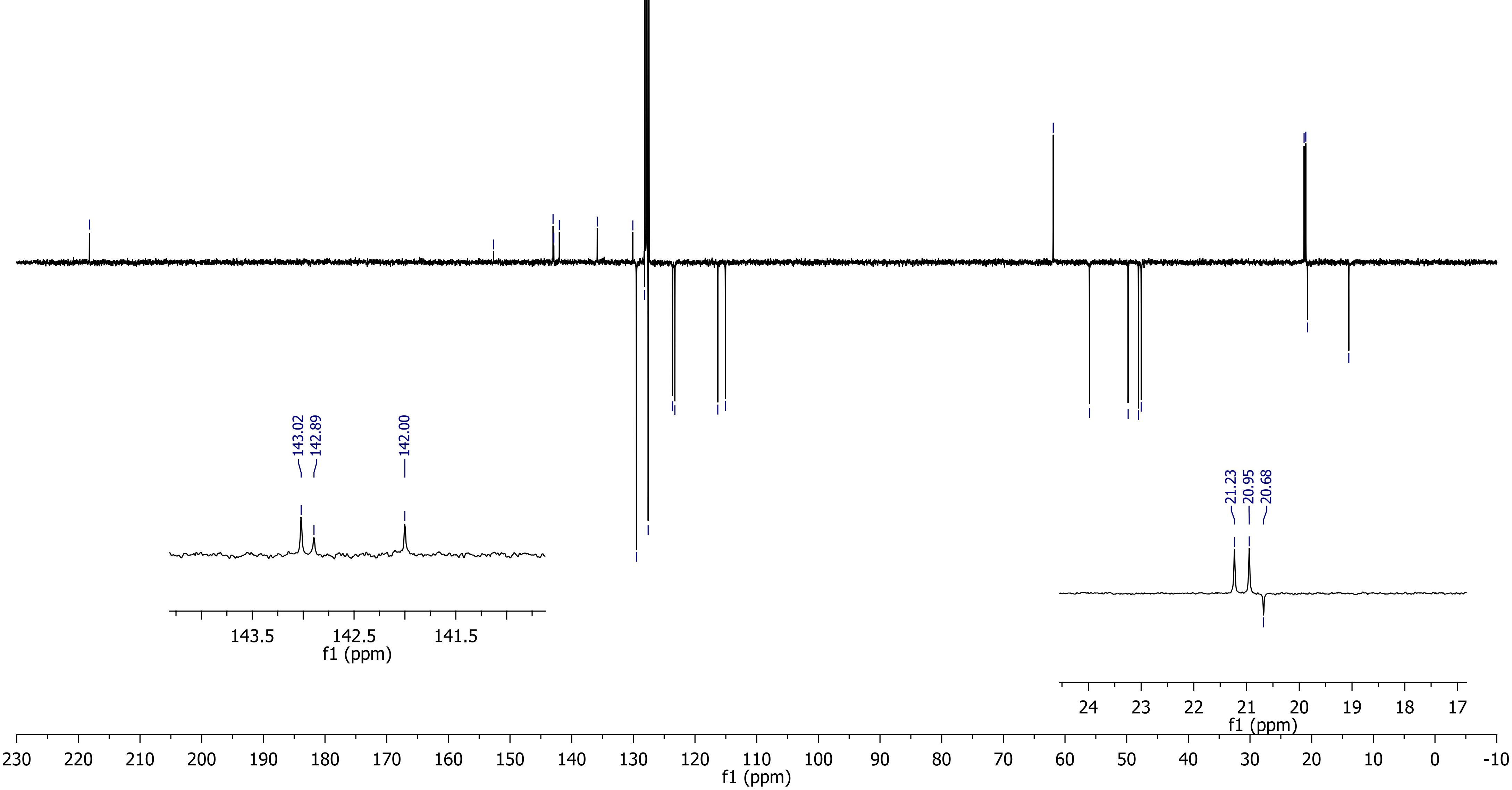

^{1}H NMR (300 MHz, C_6D_6)

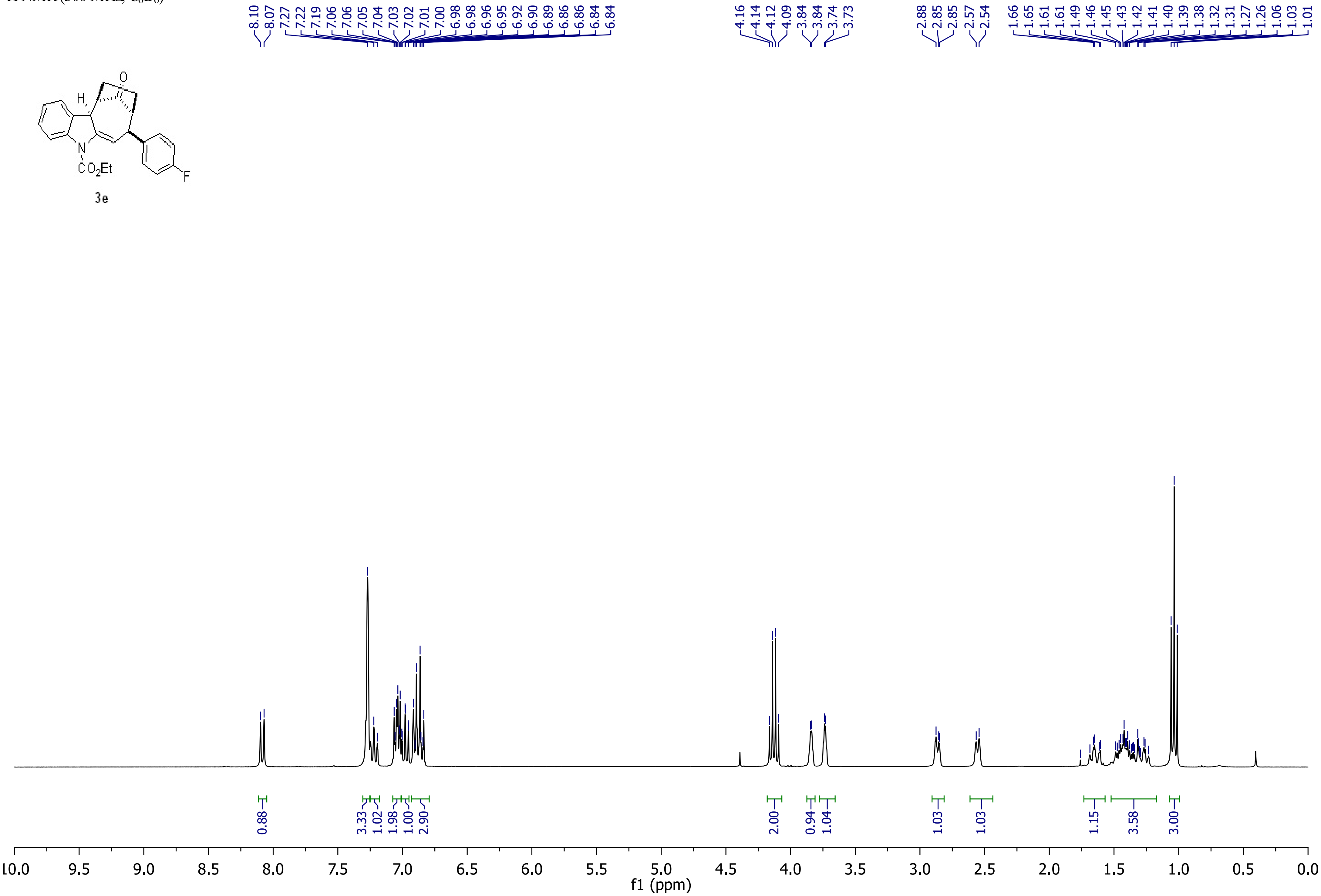

^{13}C NMR (75 MHz, C_6D_6)

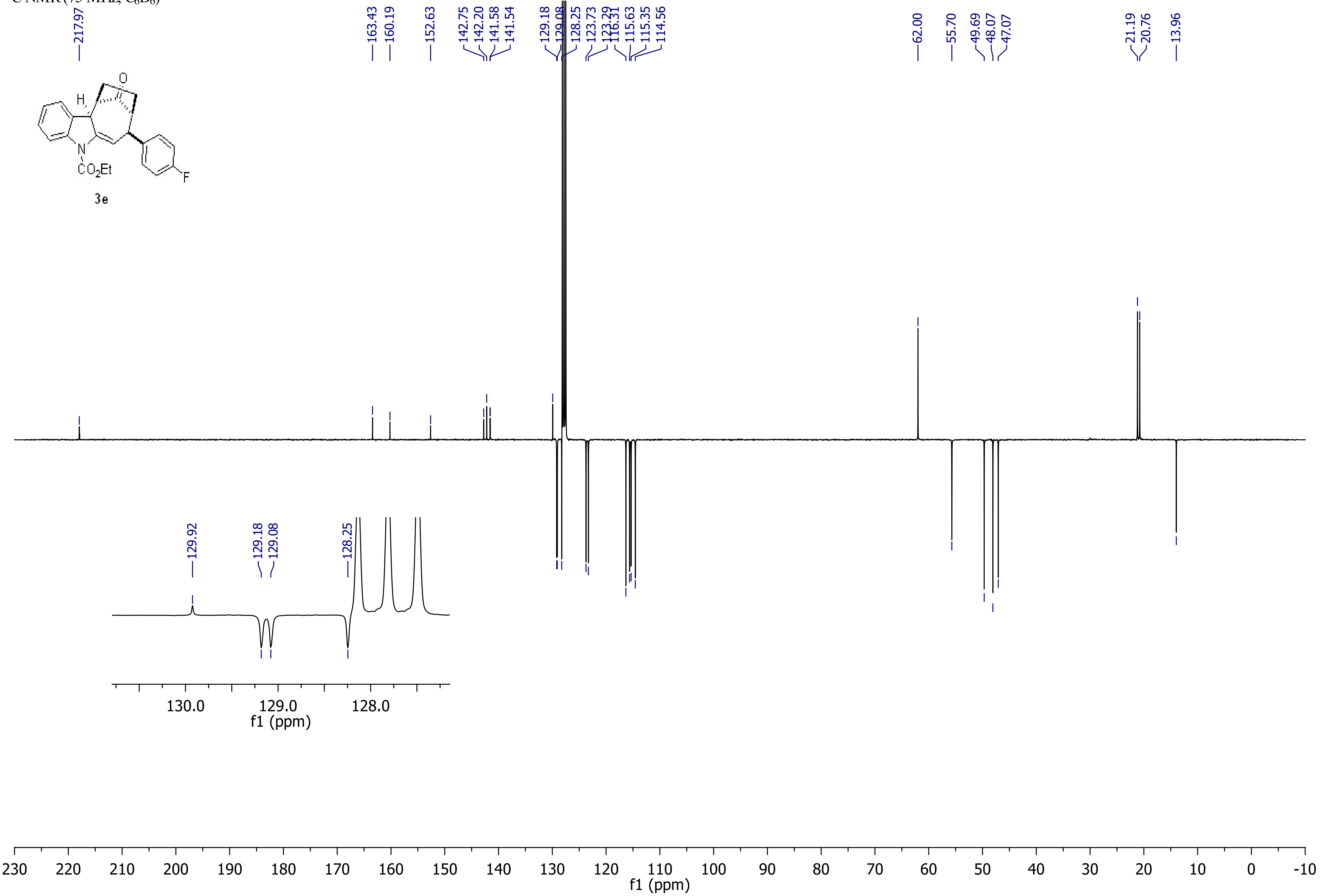

^{1}H NMR (300 MHz, C_6D_6)

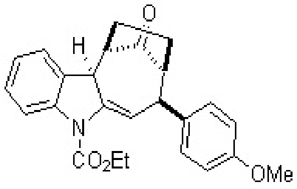

3f

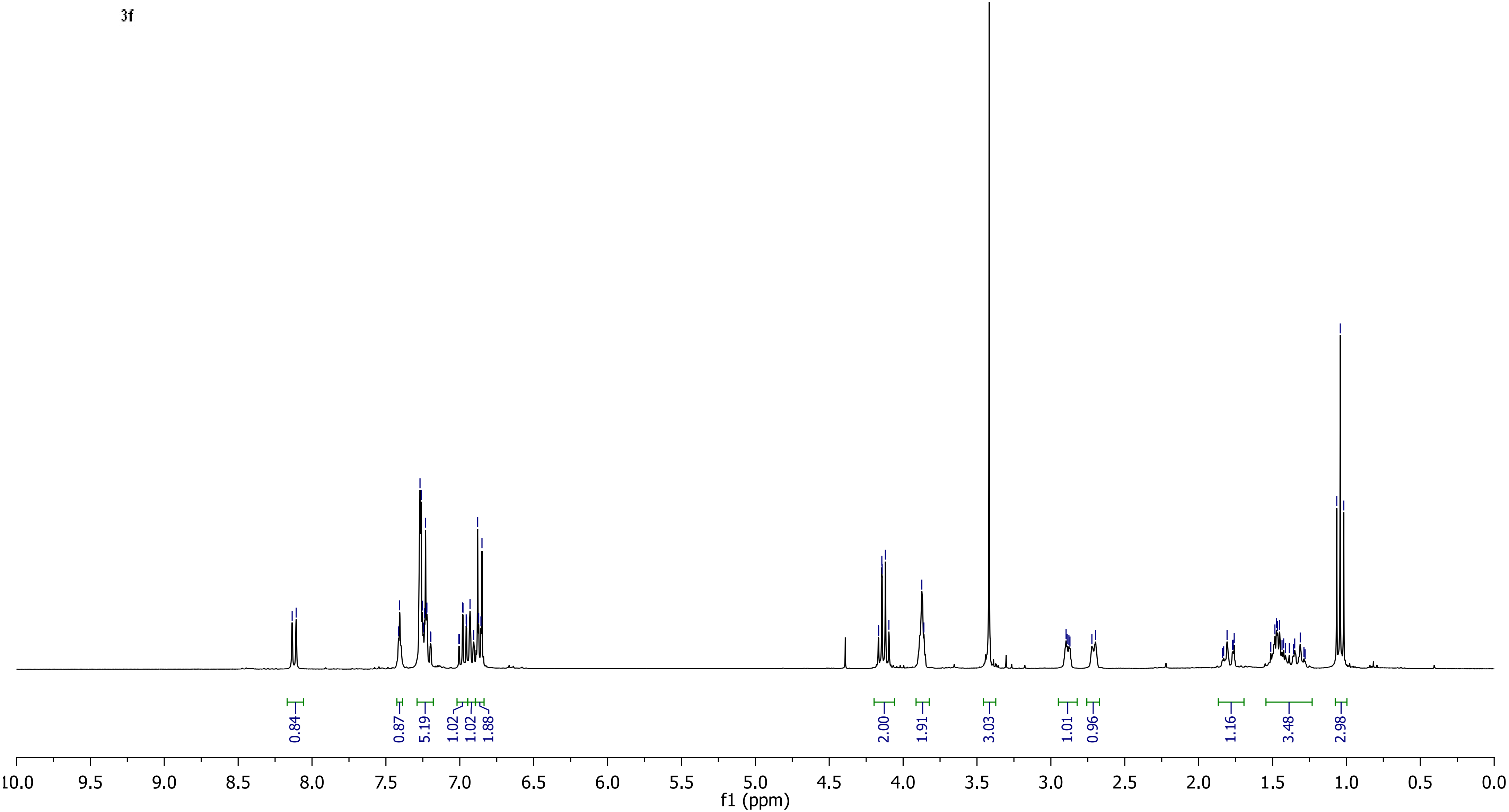

^{13}C NMR (75 MHz, C_6D_6)

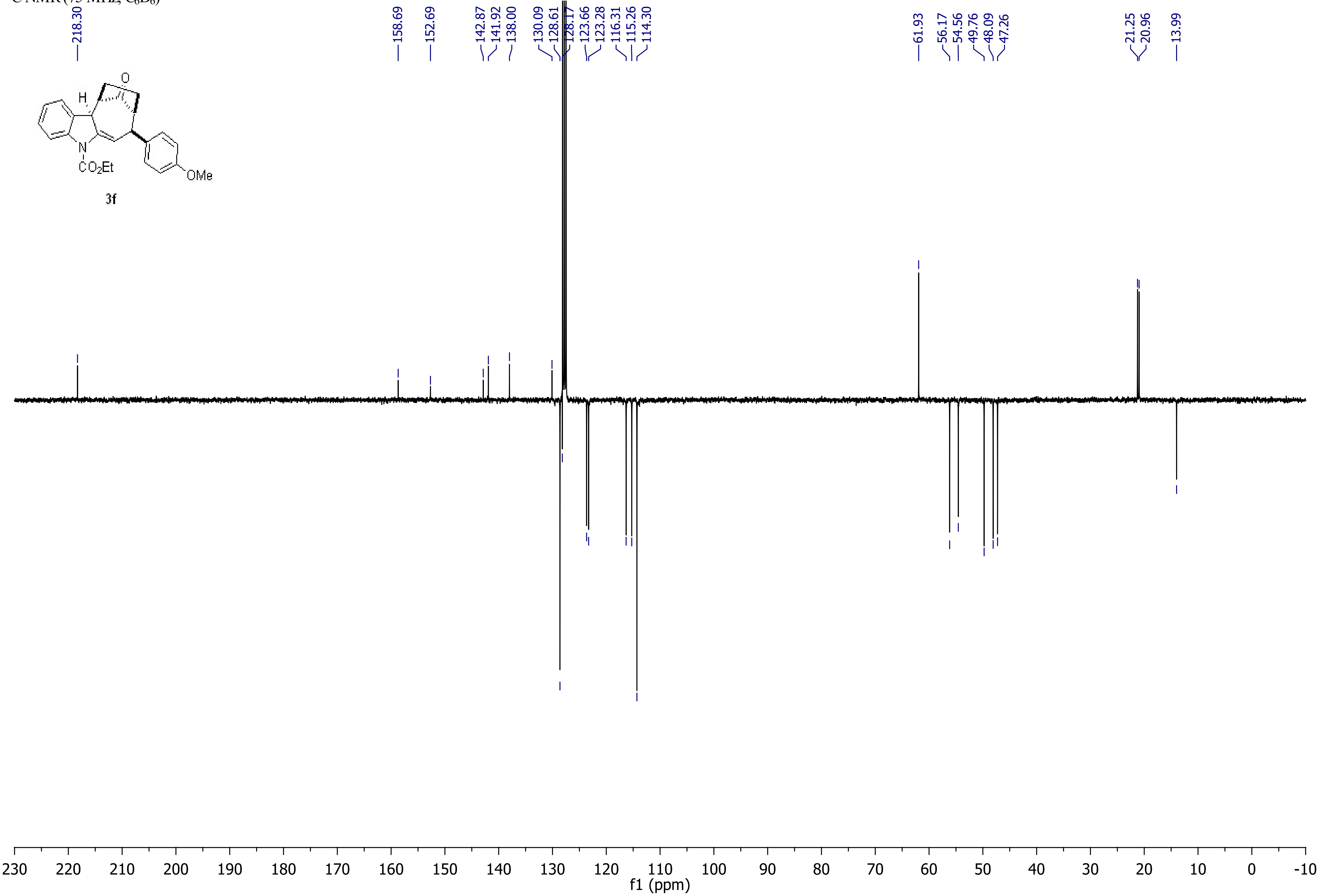

^{1}H NMR (300 MHz, C_6D_6)

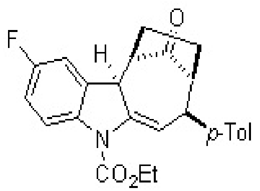

3g

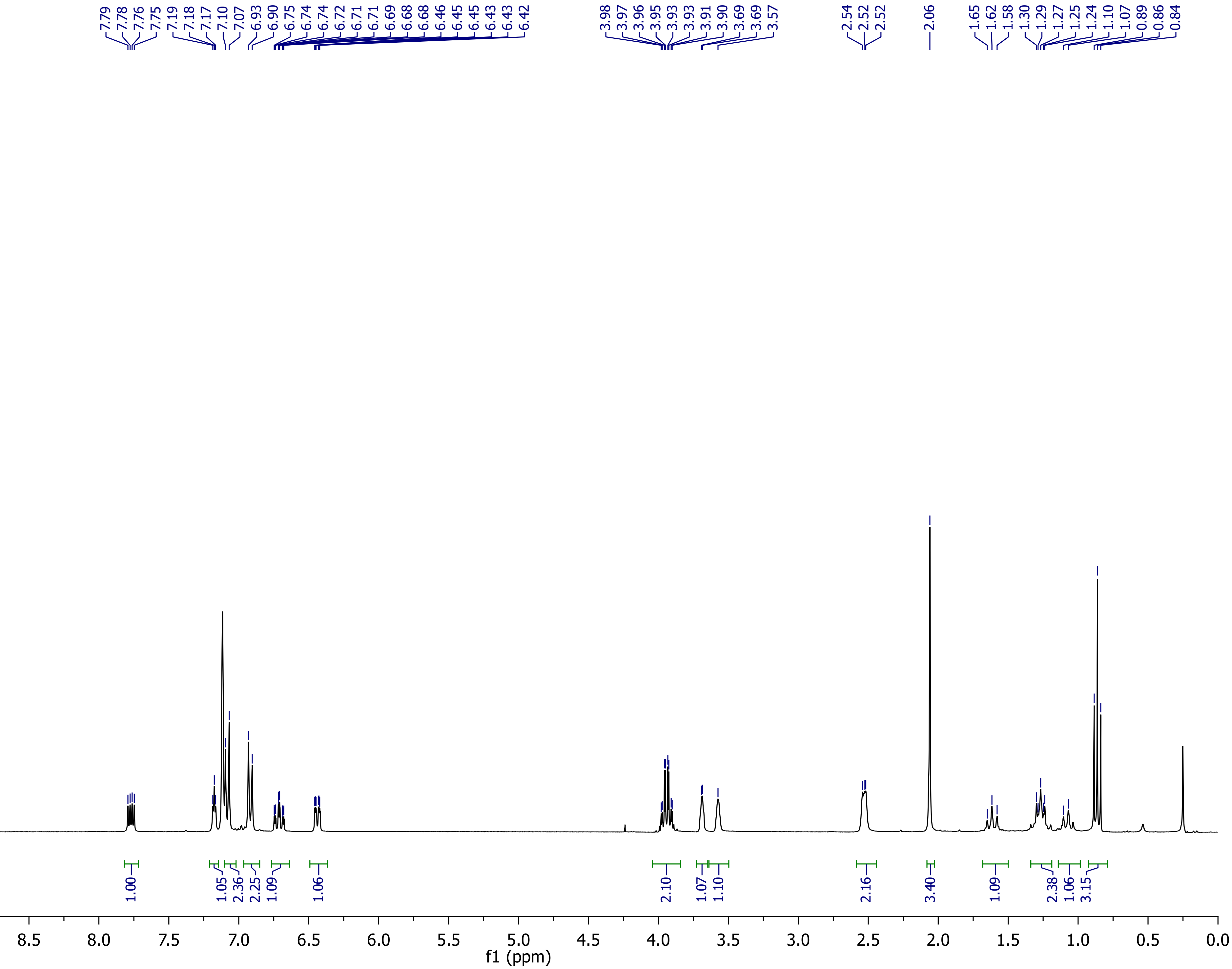

^{13}C NMR (75 MHz, C_6D_6)

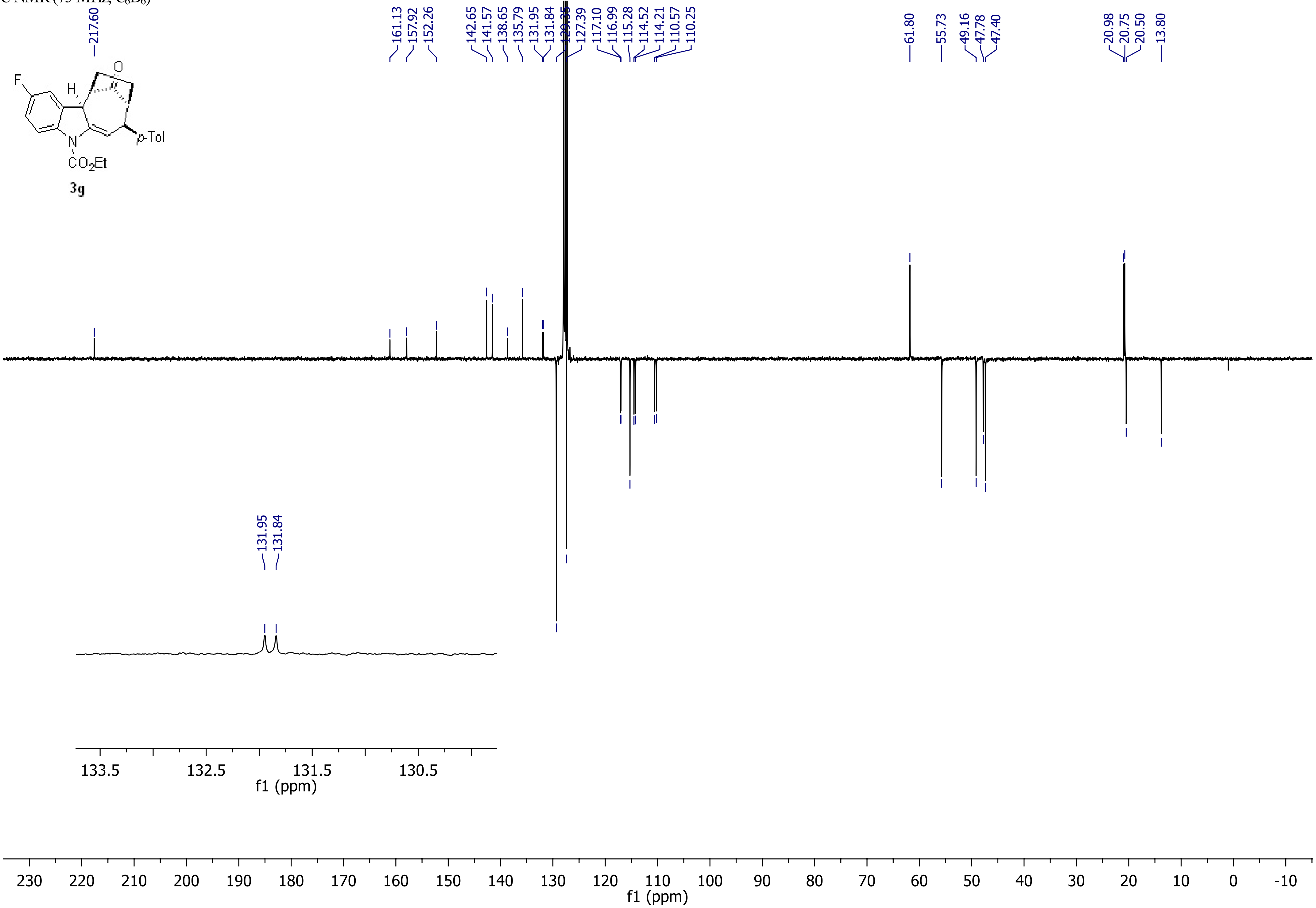

^{1}H NMR (300 MHz, C_6D_6)

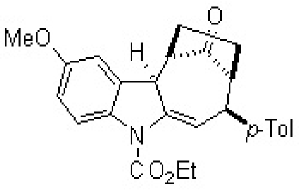

3h

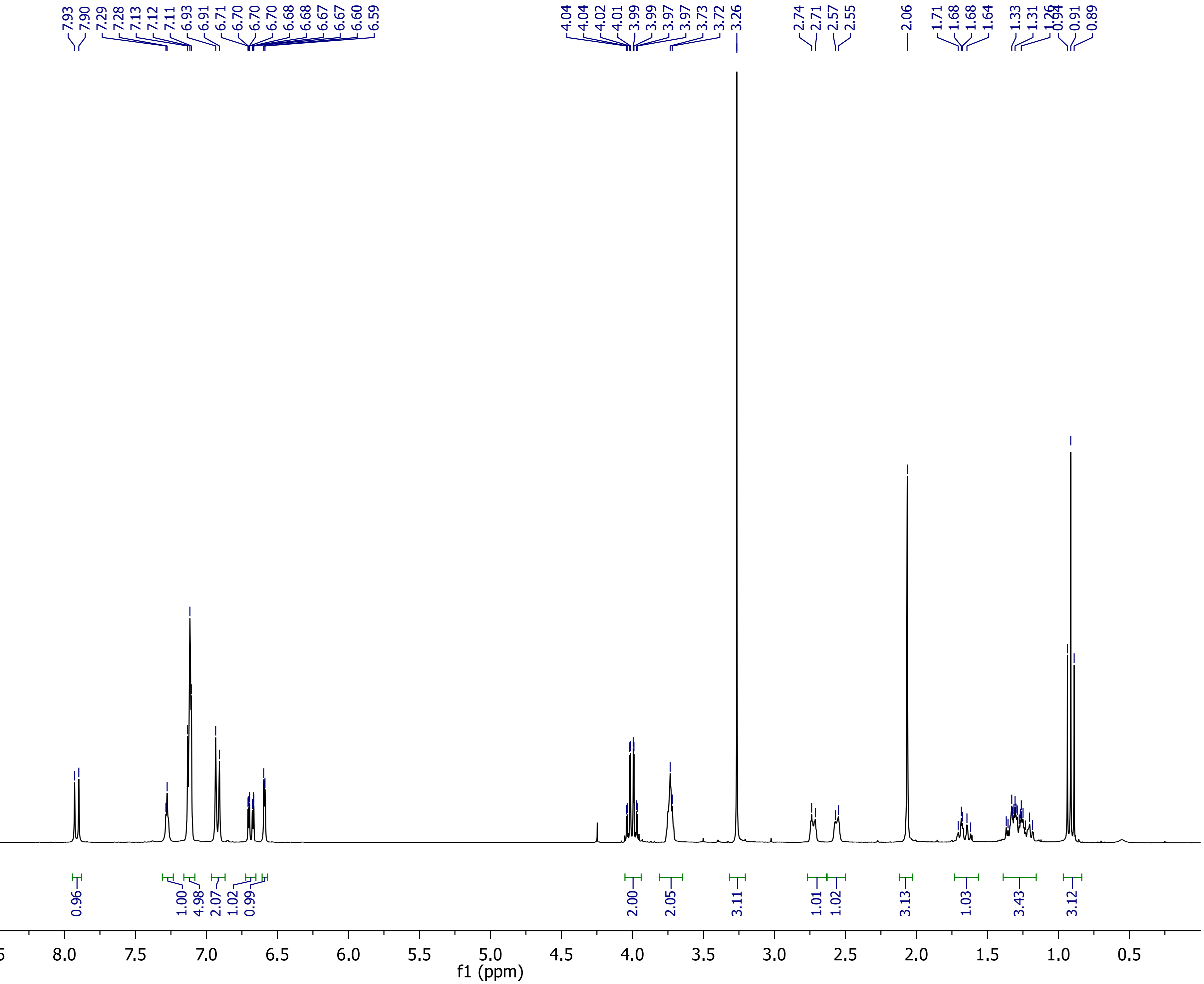

^{13}C NMR (75 MHz, C_6D_6)

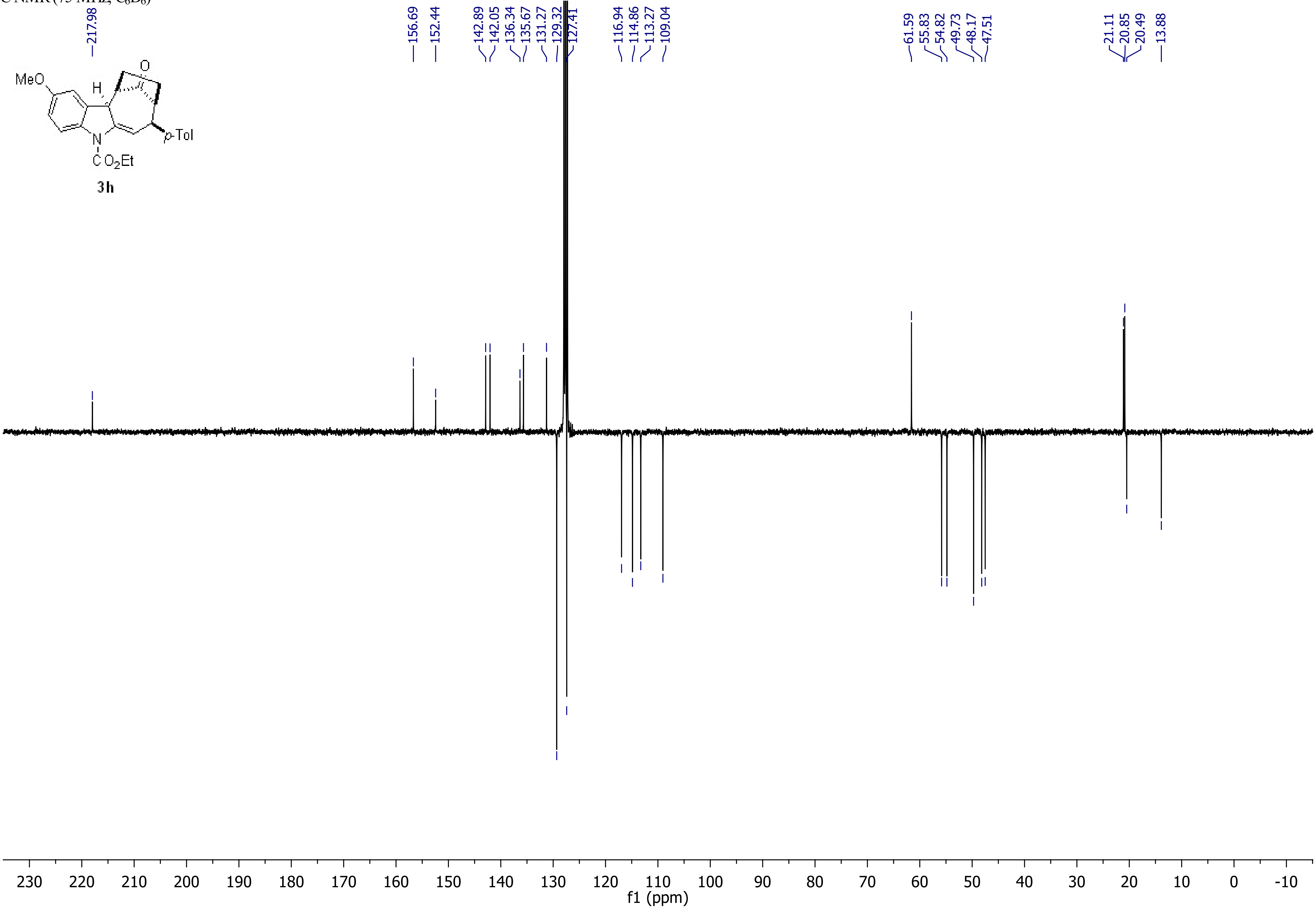

^{1}H NMR (500 MHz, C_6D_6)

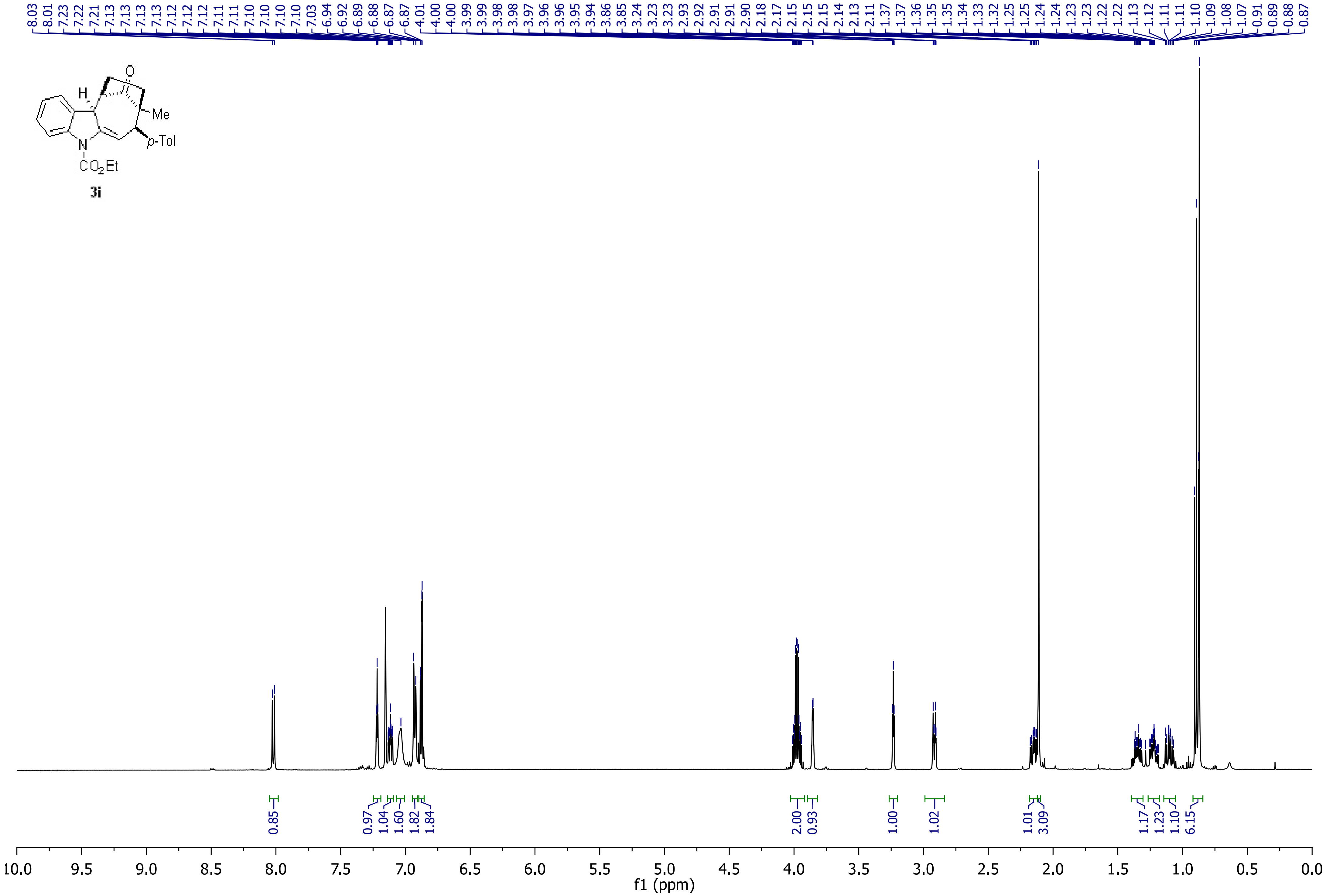

^{13}C NMR (126 MHz, C_6D_6)

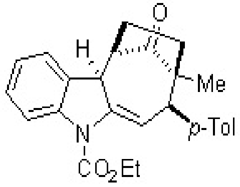

3i

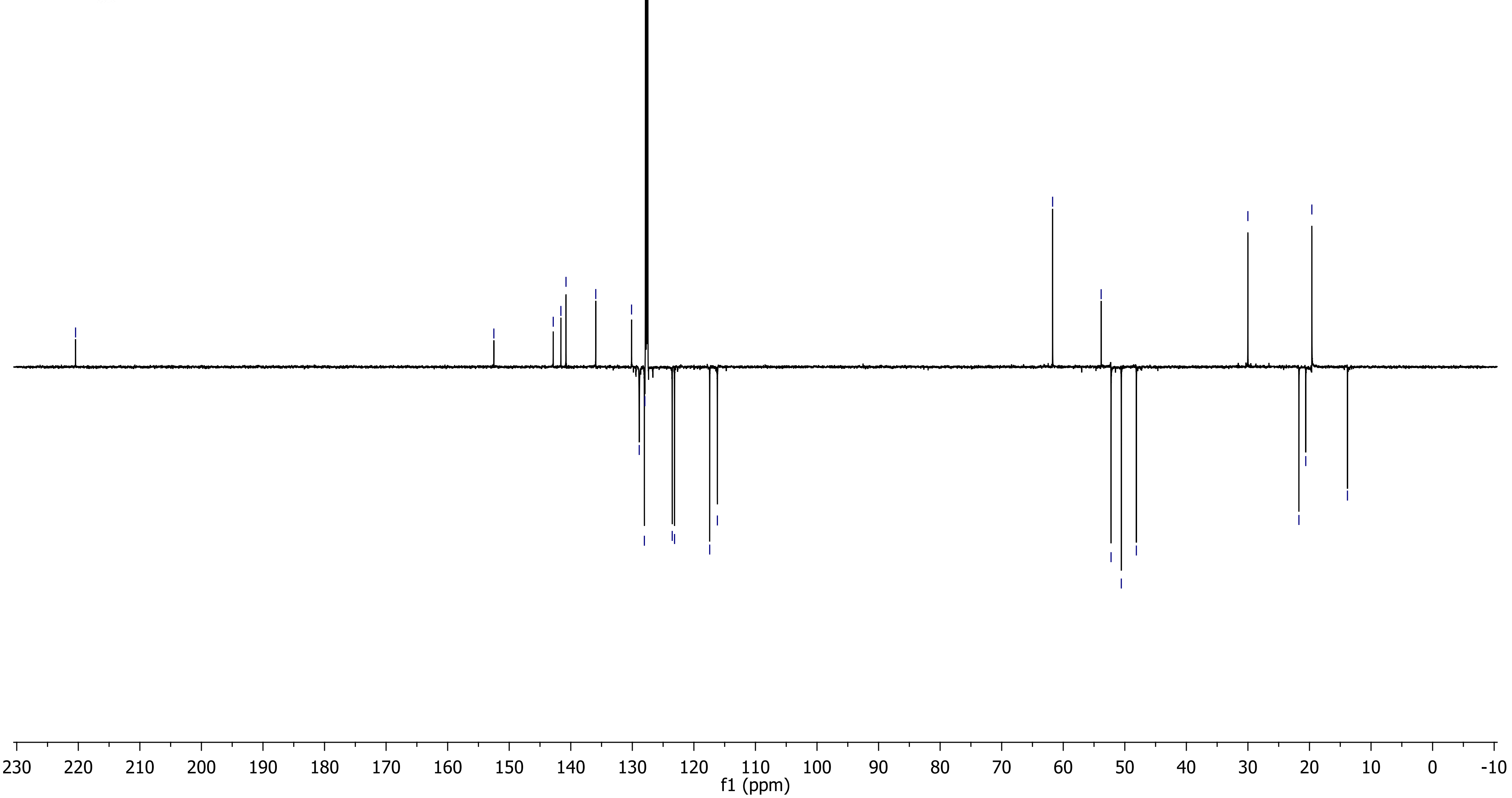

^{1}H NMR (300 MHz, C_6D_6)

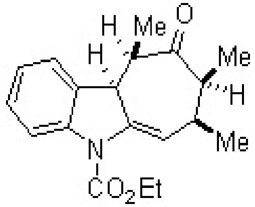

3j

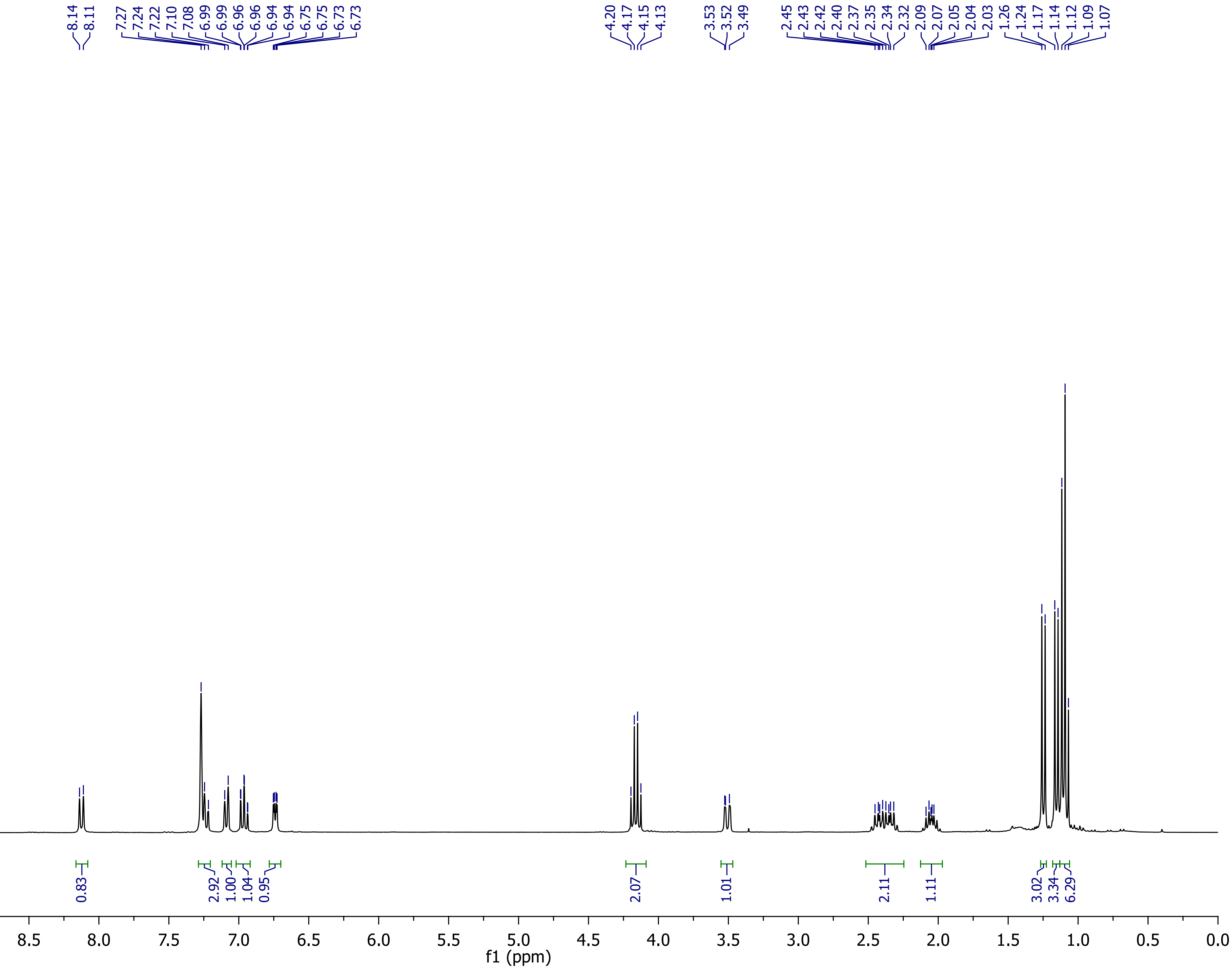

^{13}C NMR (75 MHz, C_6D_6)

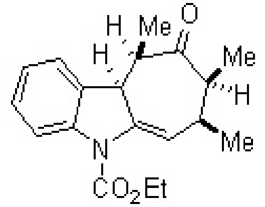

3j

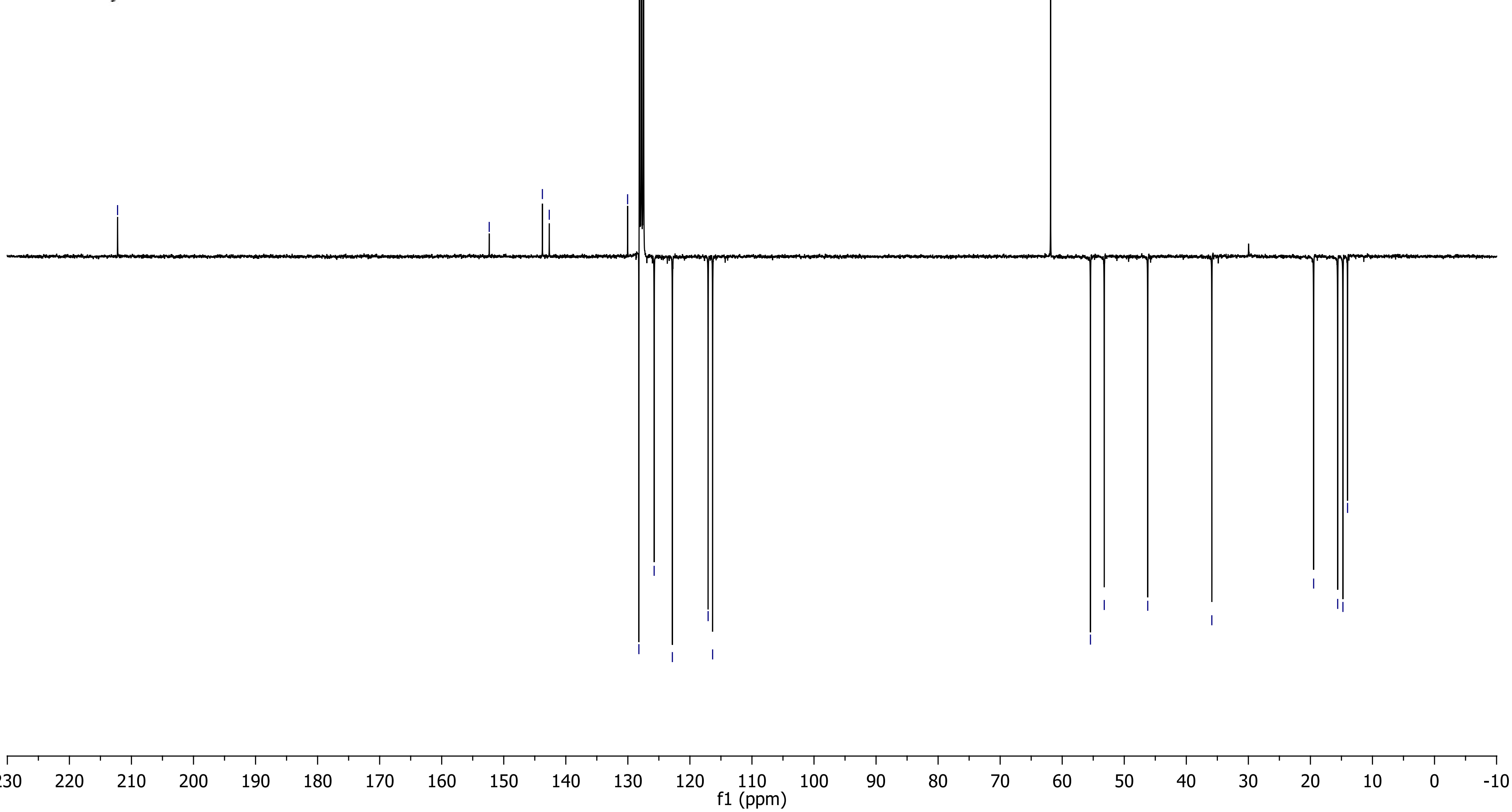

^{1}H NMR (300 MHz, C_6D_6)

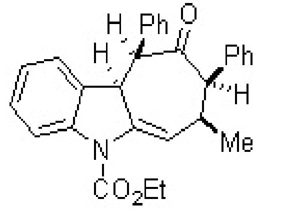

3k

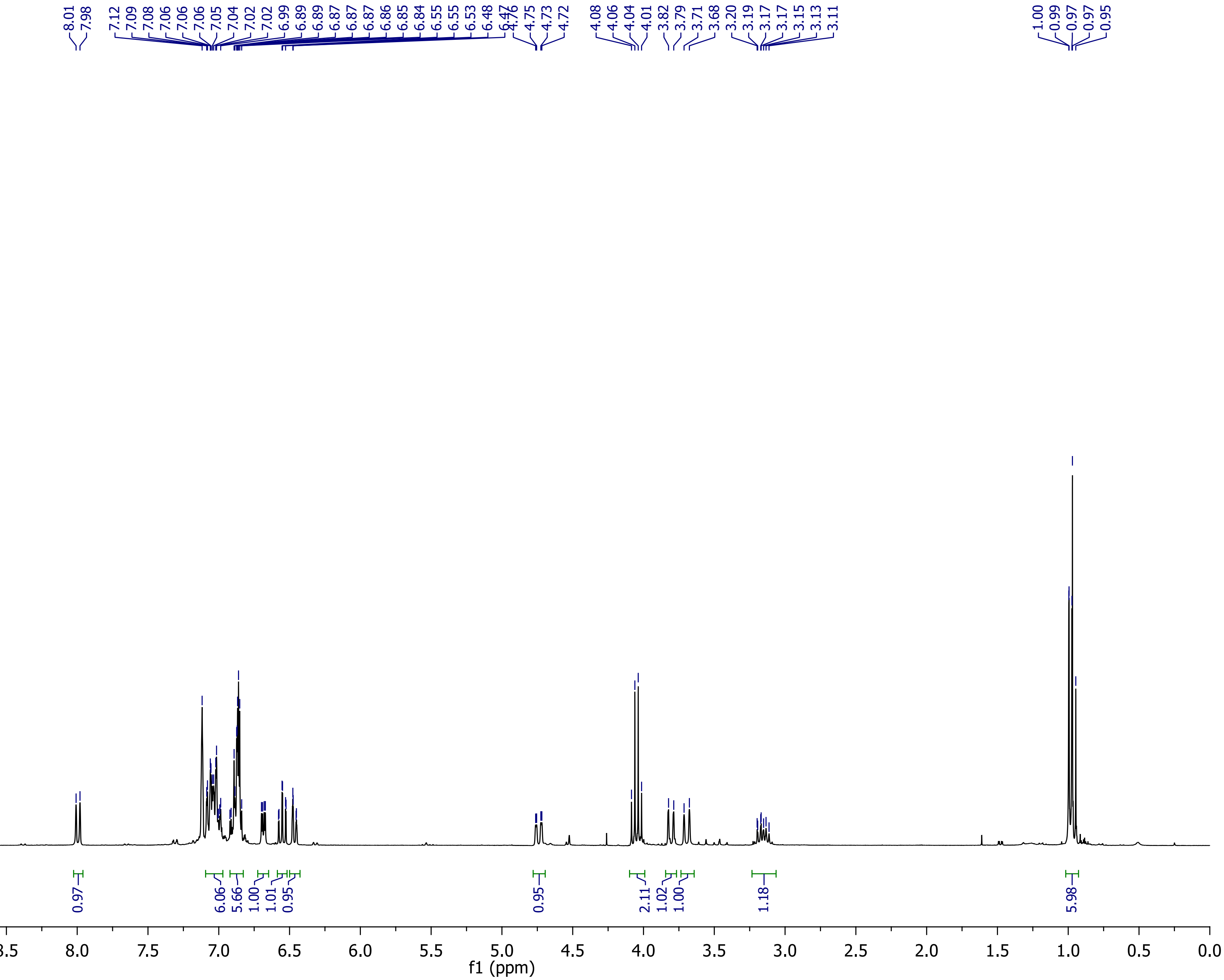

^{13}C NMR (75 MHz, C_6D_6)

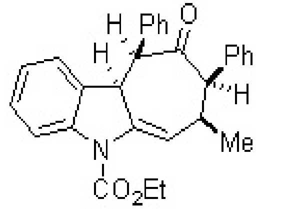

3k

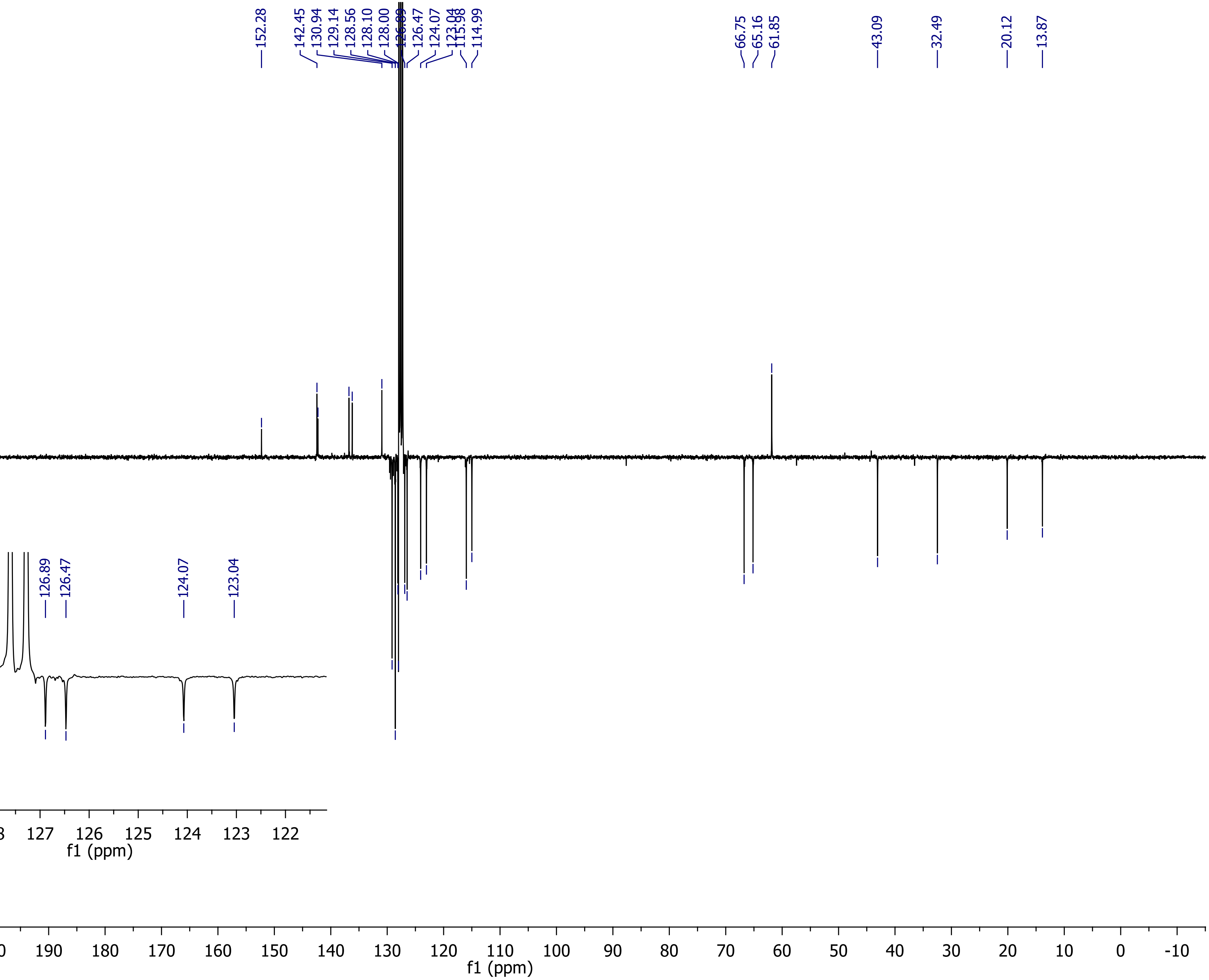

^{1}H NMR (300 MHz, C_6D_6)

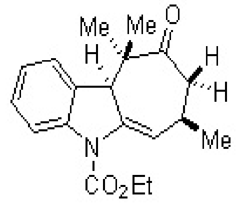

3l

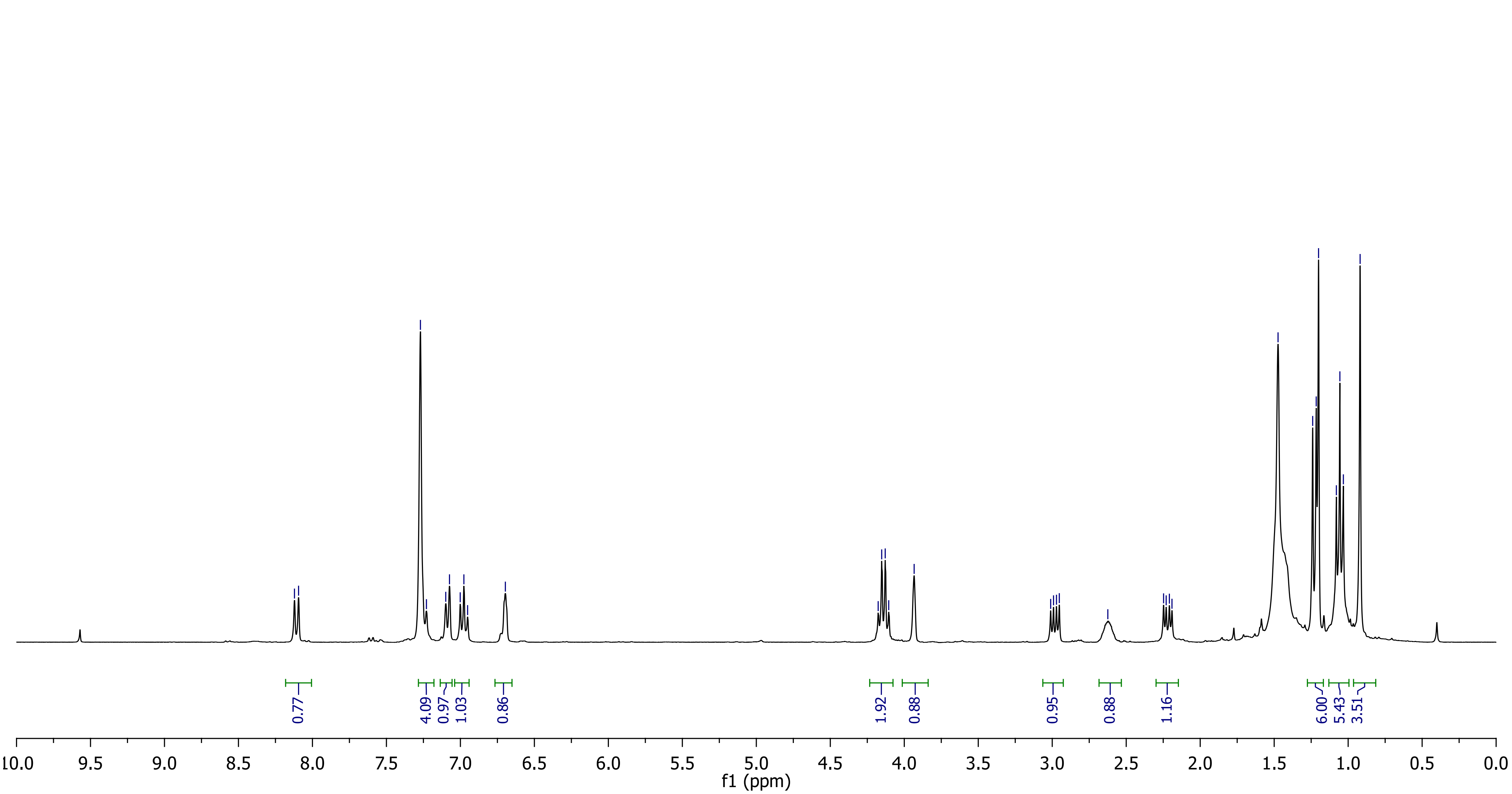

^{13}C NMR (75 MHz, C_6D_6) .

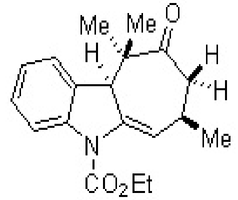

3l

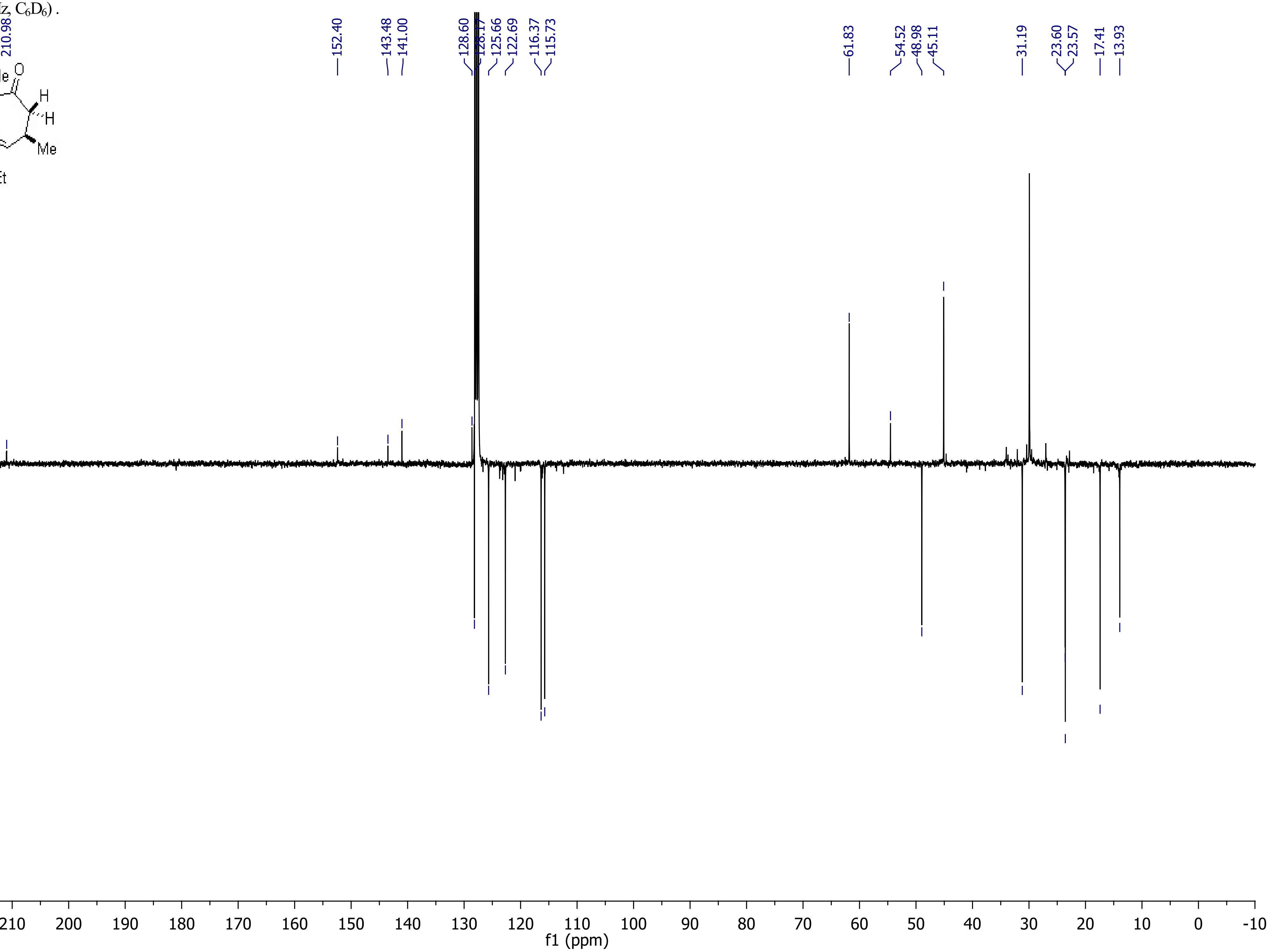

^{1}H NMR (300 MHz, C_6D_6)

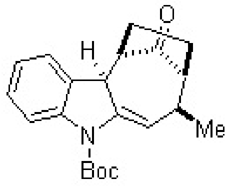

3m

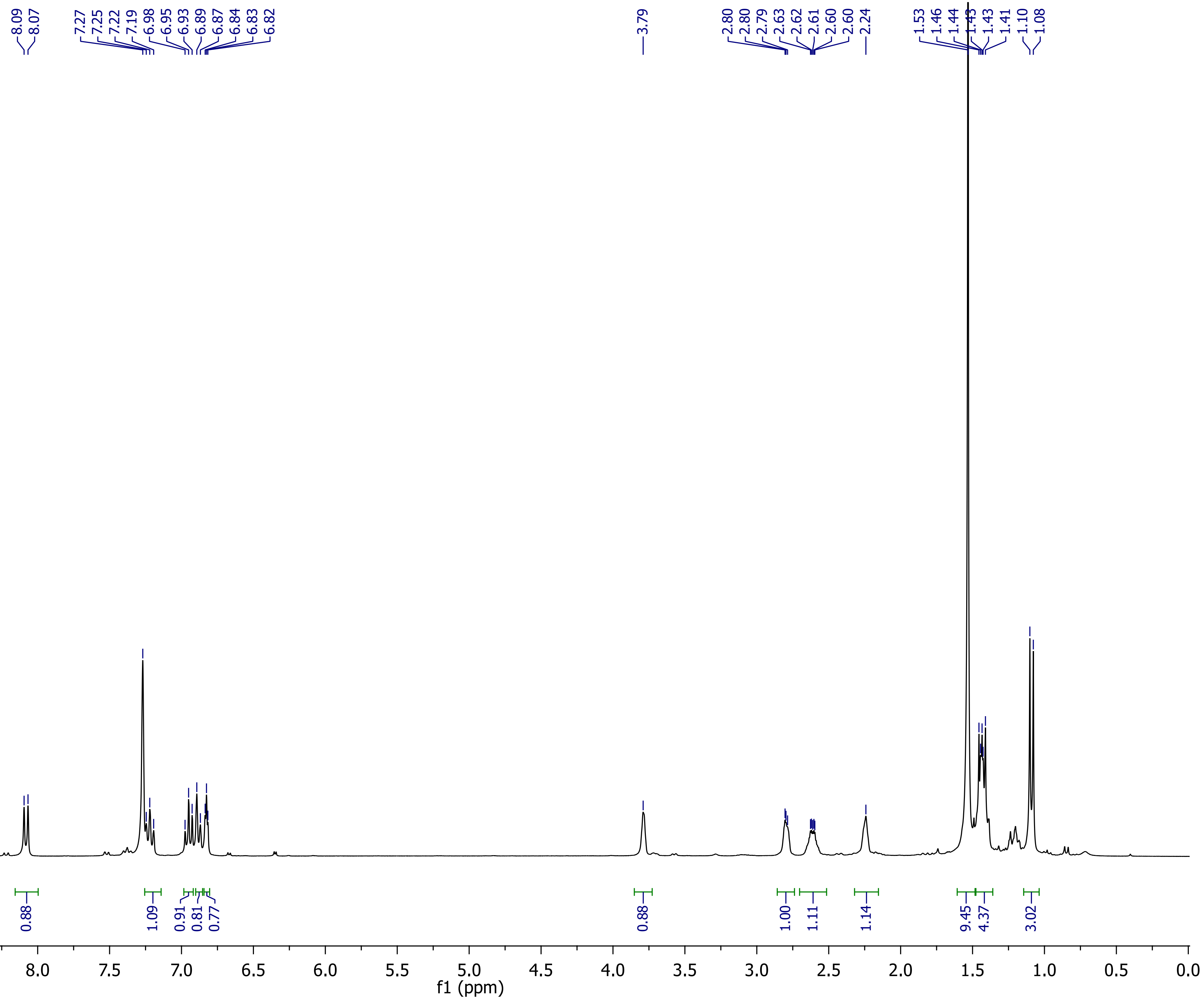

^{13}C NMR (75 MHz, C_6D_6)

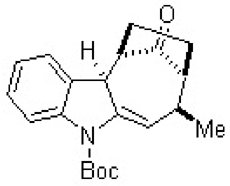

3m

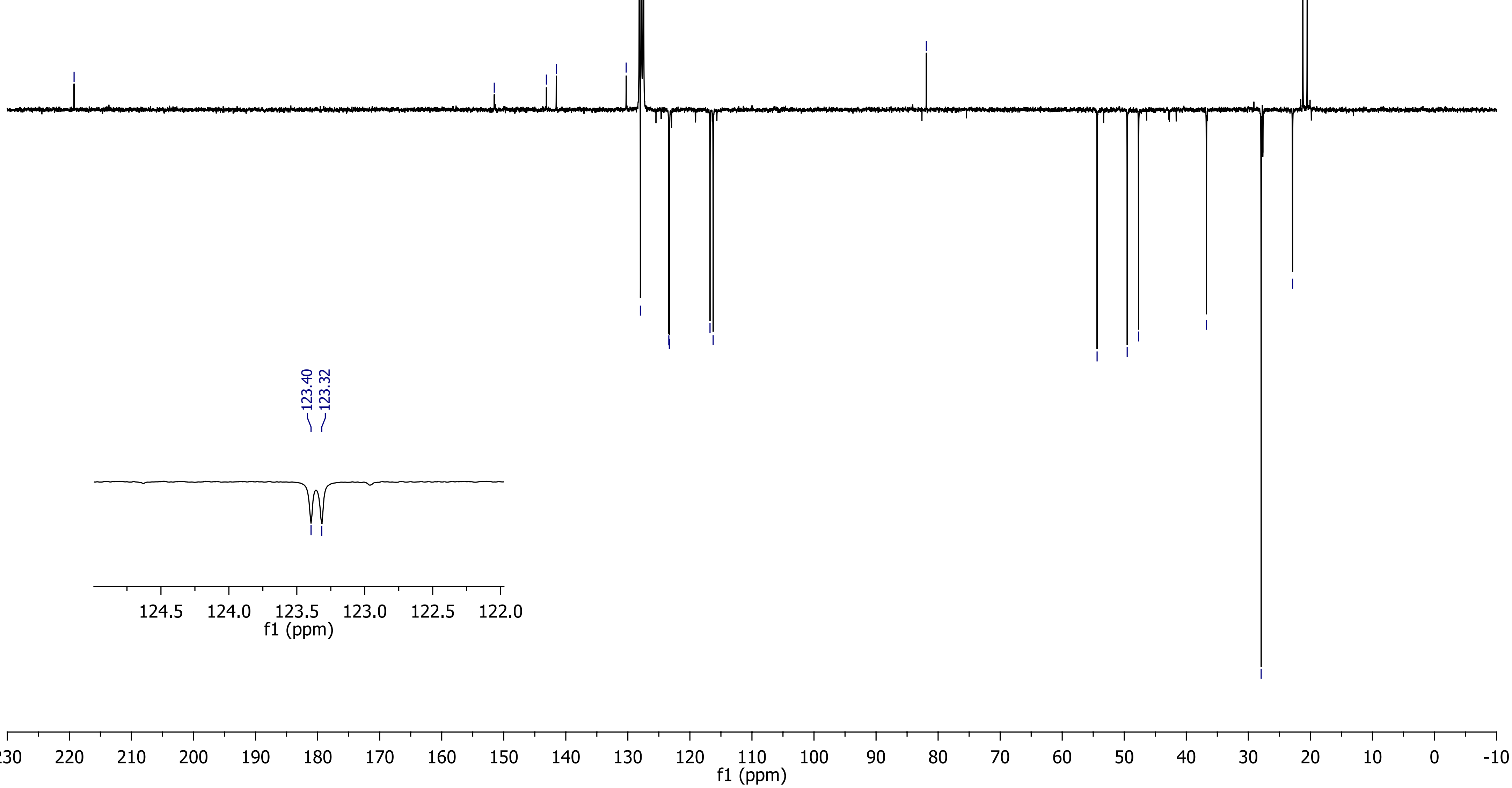

^{1}H NMR (300 MHz, CD_2Cl_2)

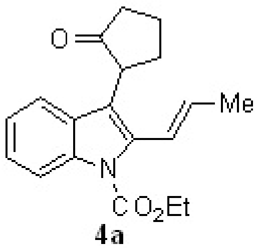

4a

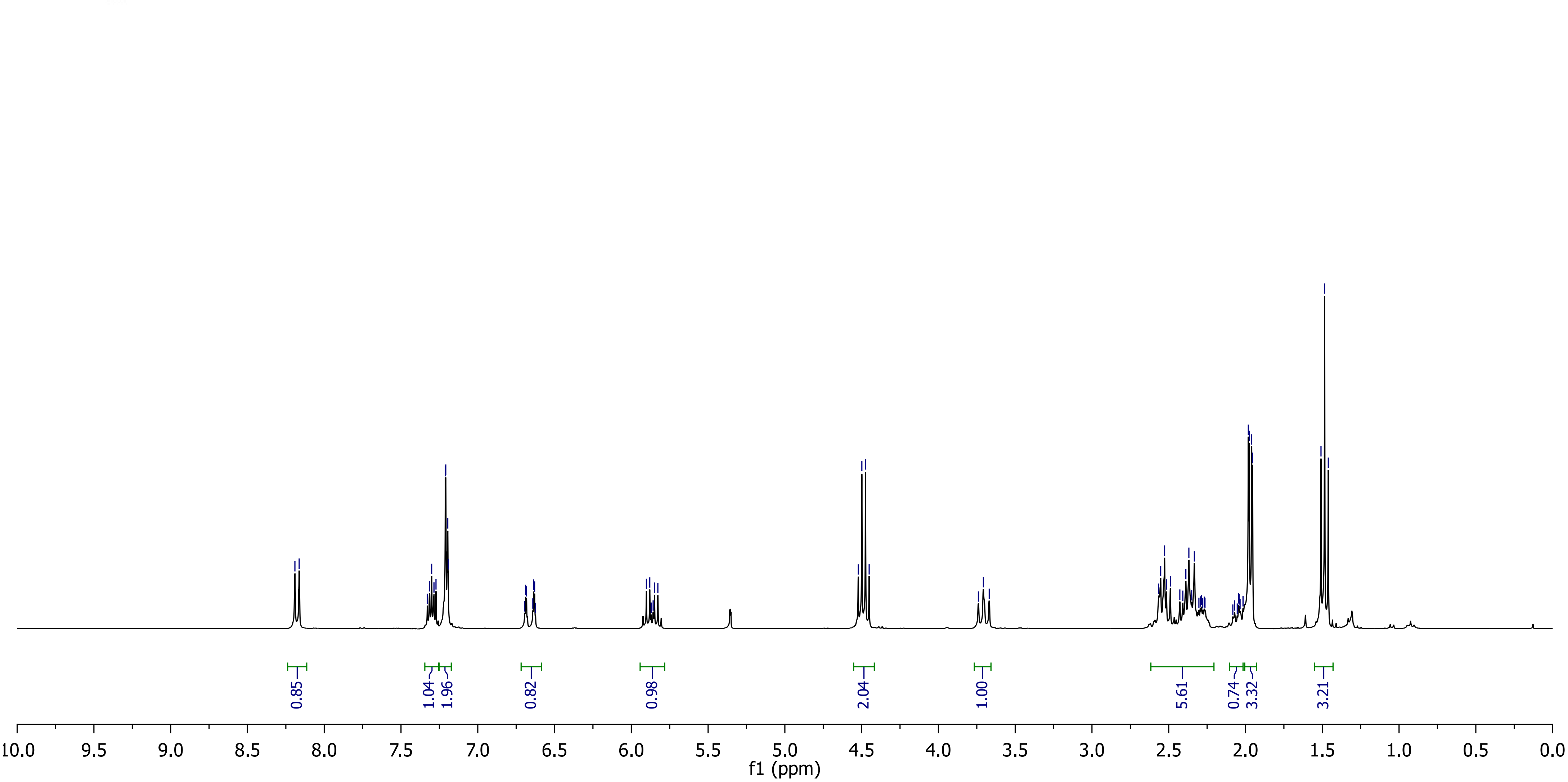

^{13}C NMR (75 MHz, CD_2Cl_2)

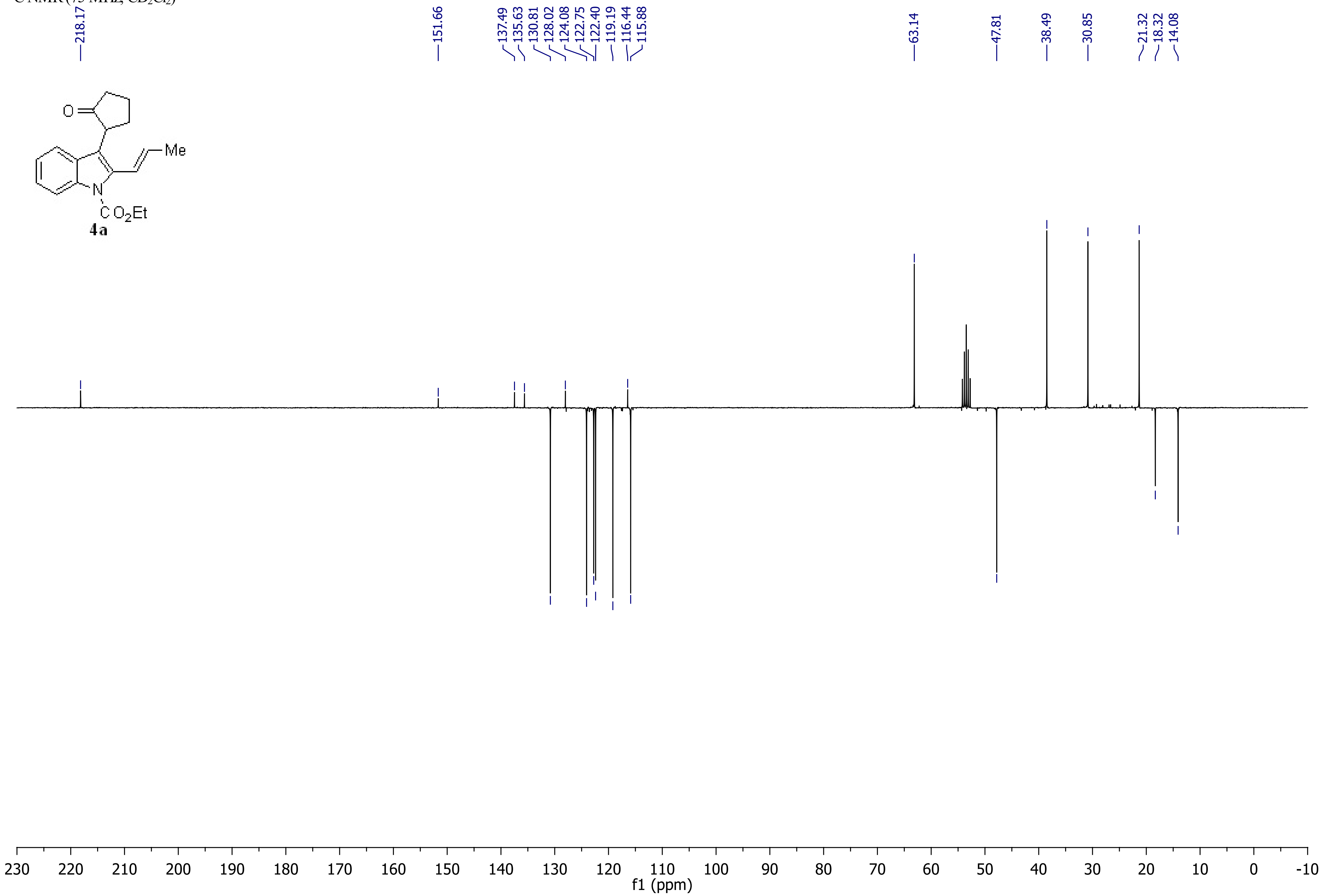

^{1}H NMR (300 MHz, $CDCl_3$)

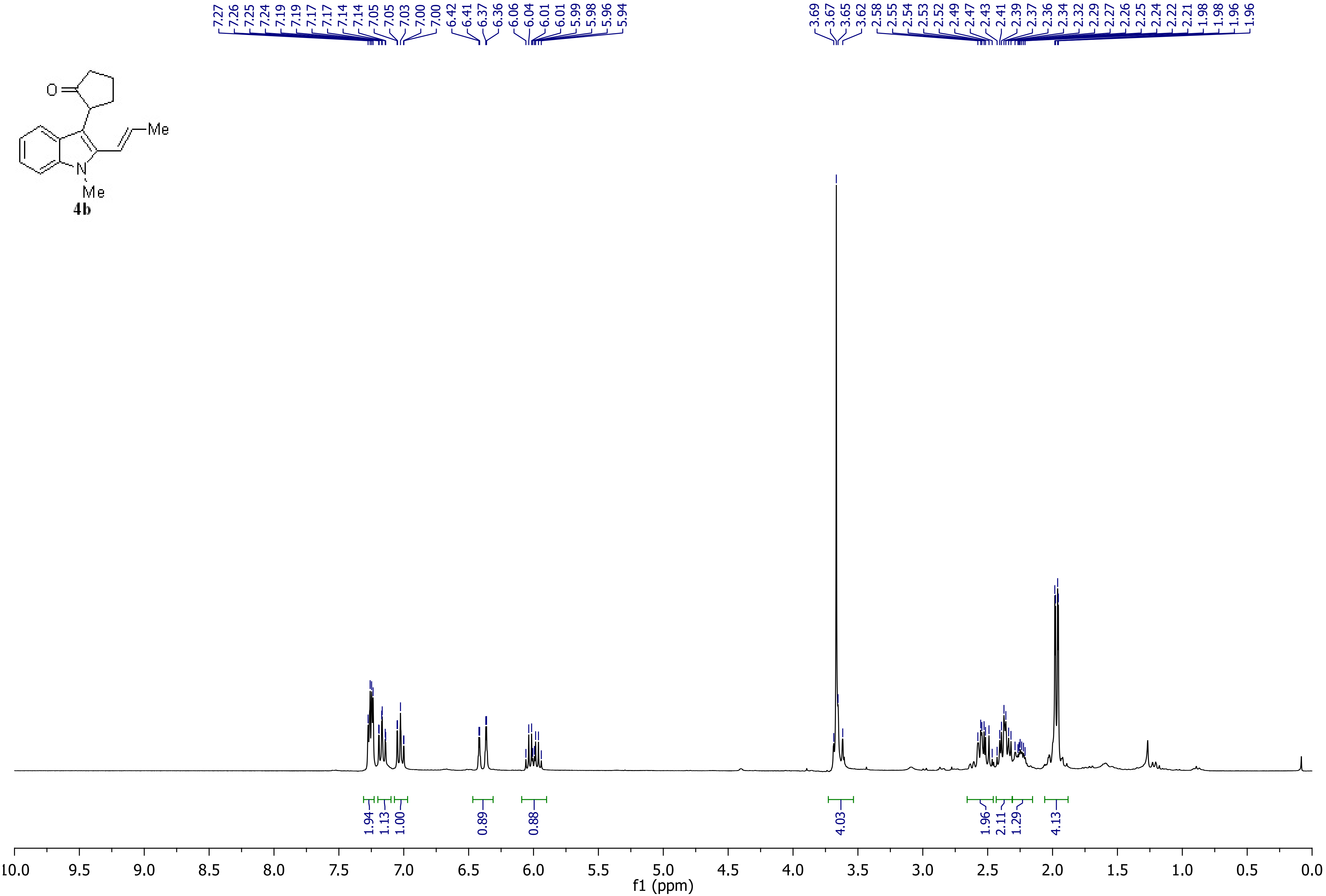

^{13}C NMR (75 MHz, $CDCl_3$)

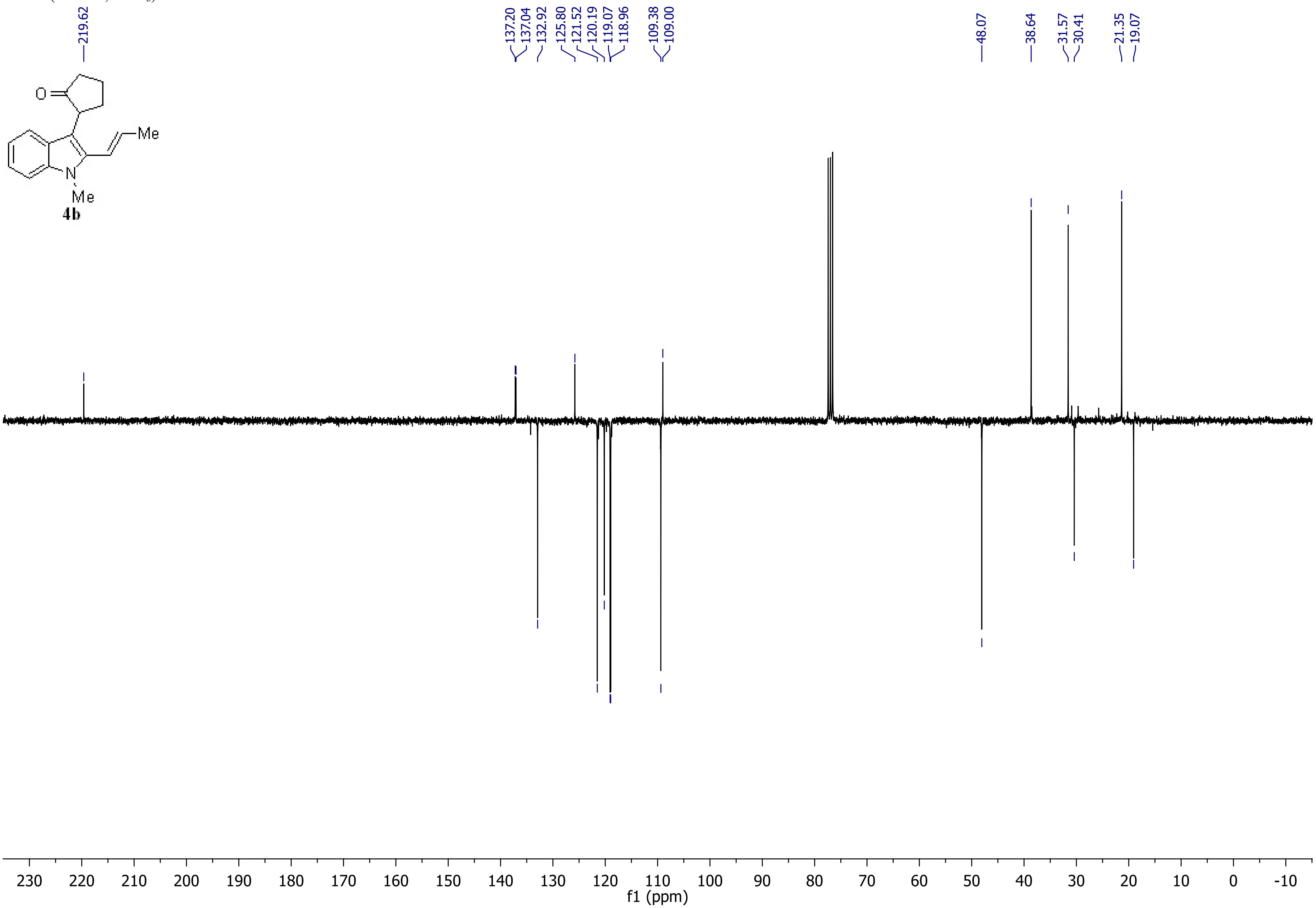

4b

^{1}H NMR (300 MHz, $CDCl_3$)

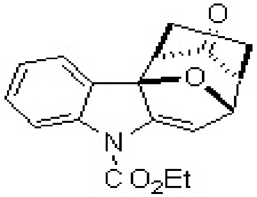

6 a

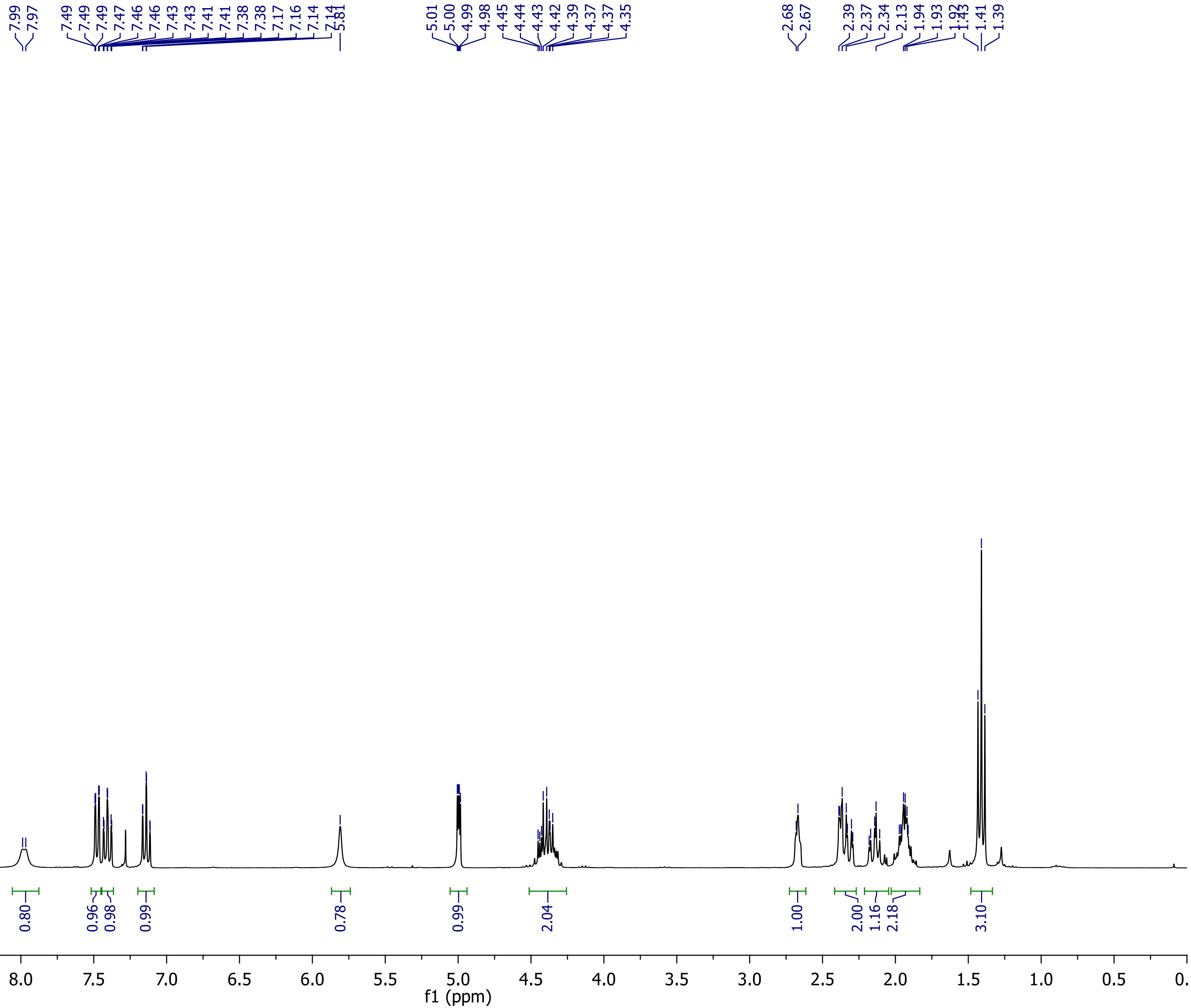

^{13}C NMR (75 MHz, $CDCl_3$)

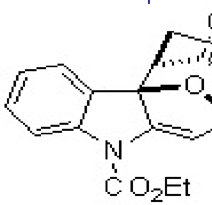

6 a

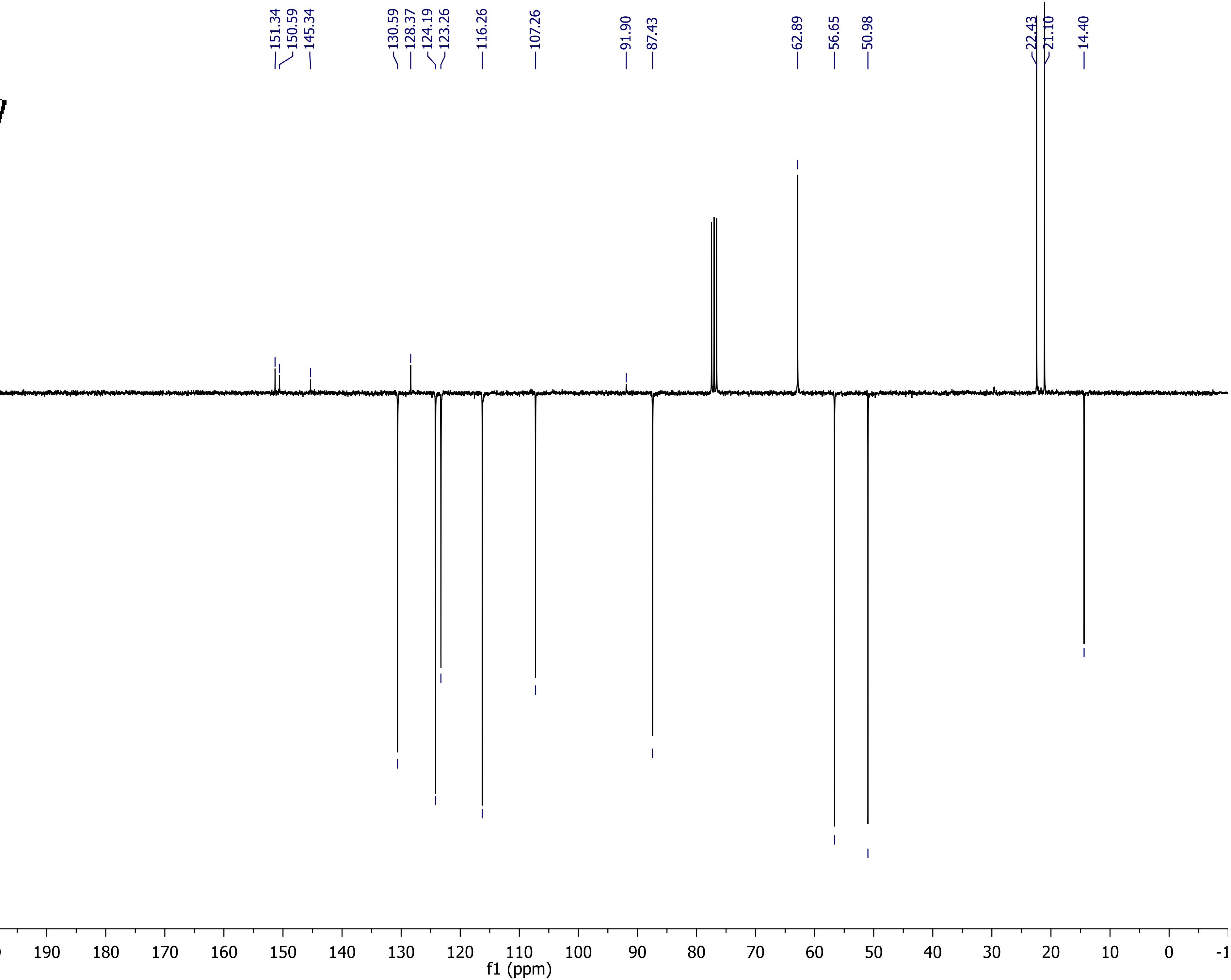

^{1}H NMR (300 MHz, C_6D_6)

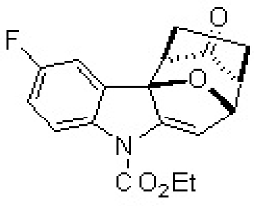

6b

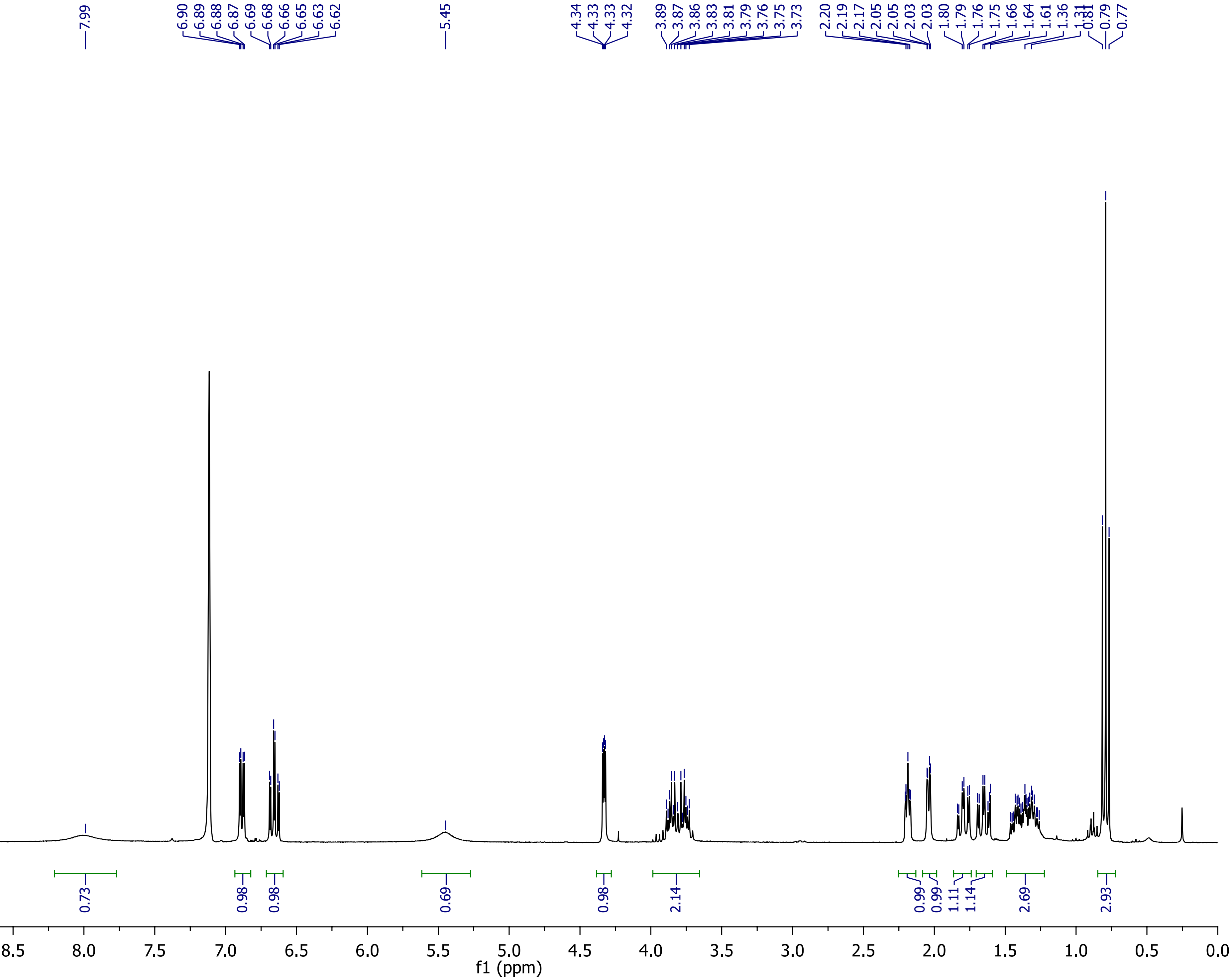

^{13}C NMR (75 MHz, C_6D_6)

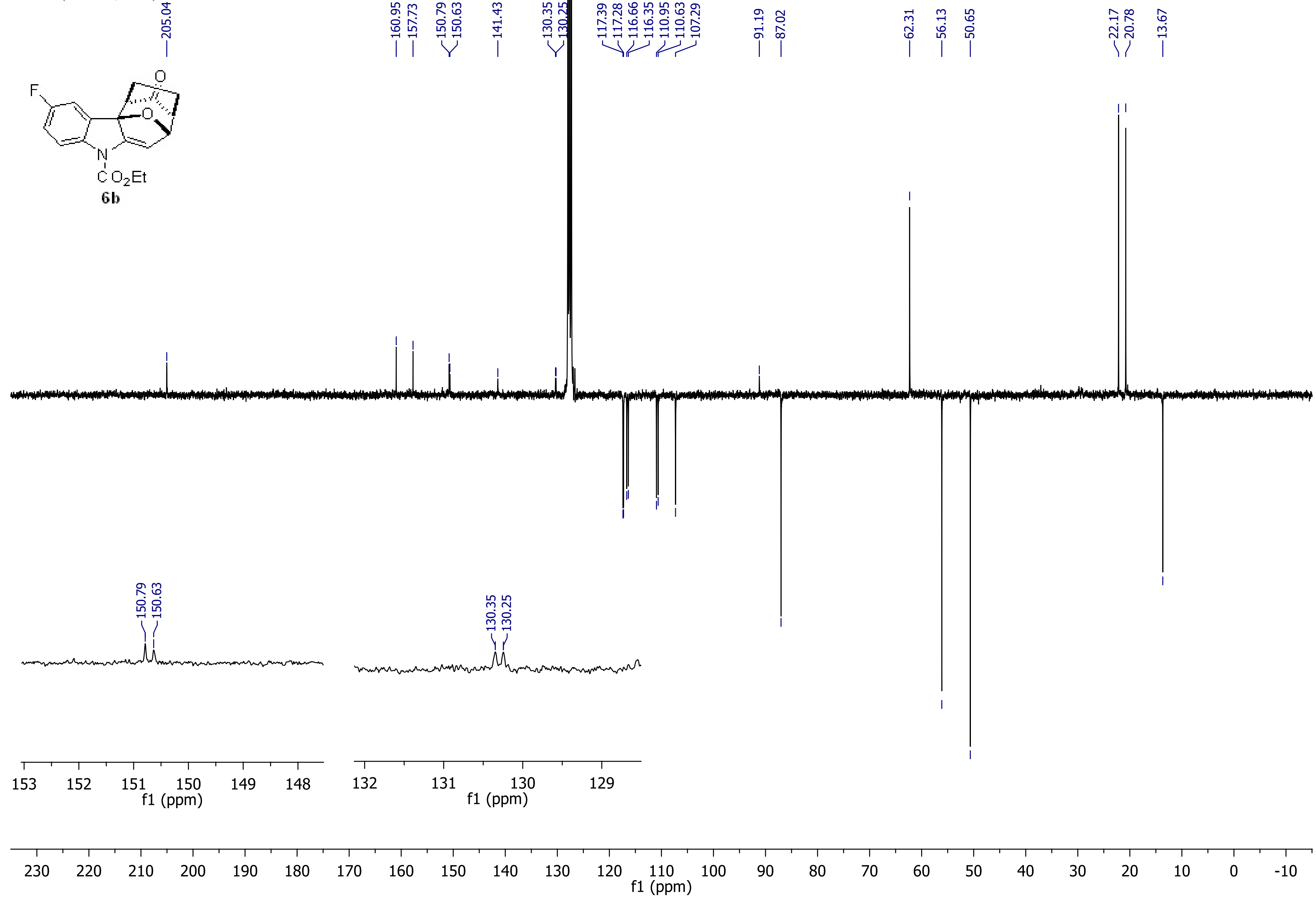

^{1}H NMR (300 MHz, C_6D_6)

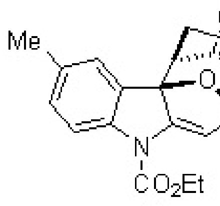

6c

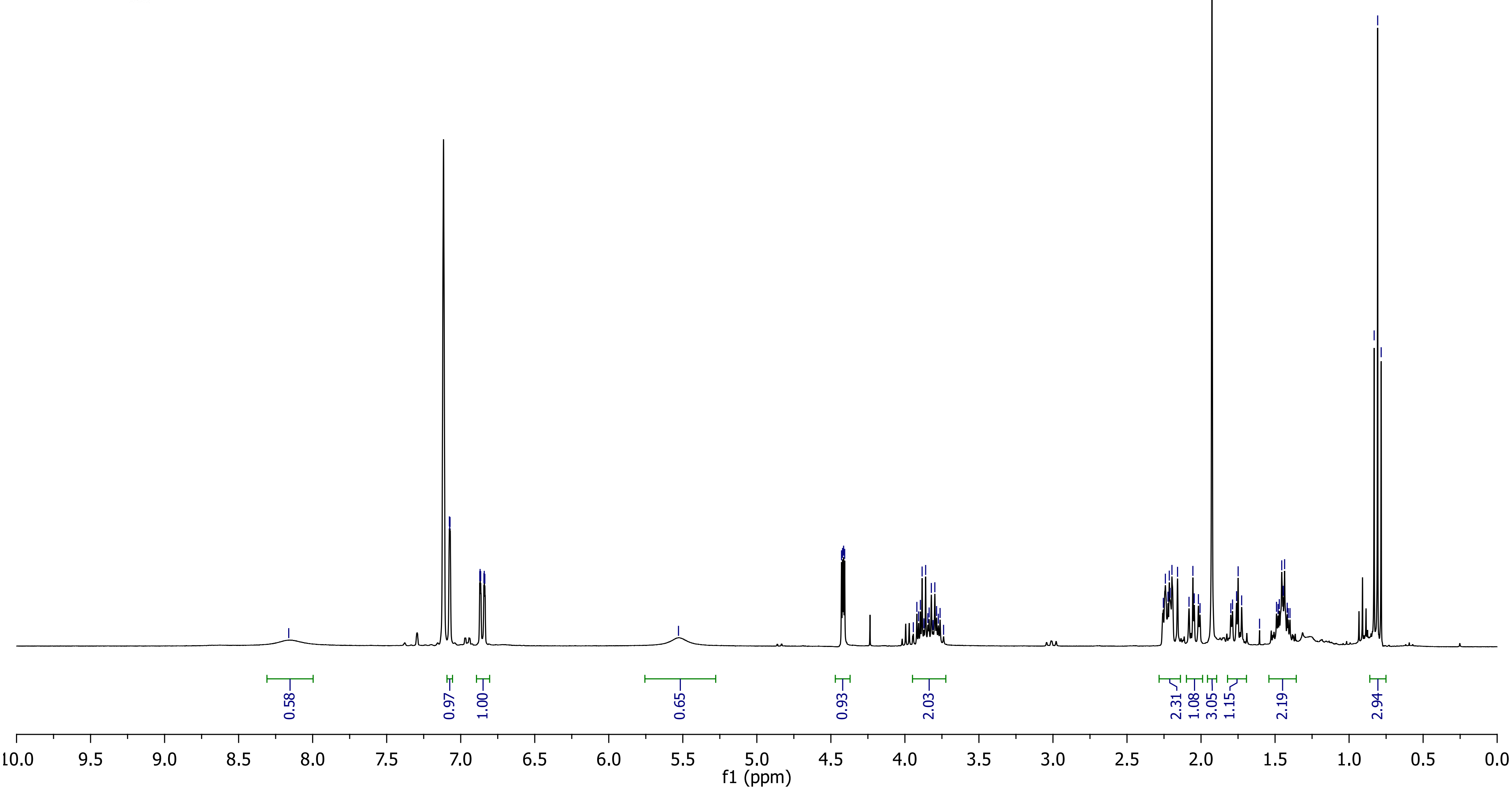

^{13}C NMR (75 MHz, C_6D_6)

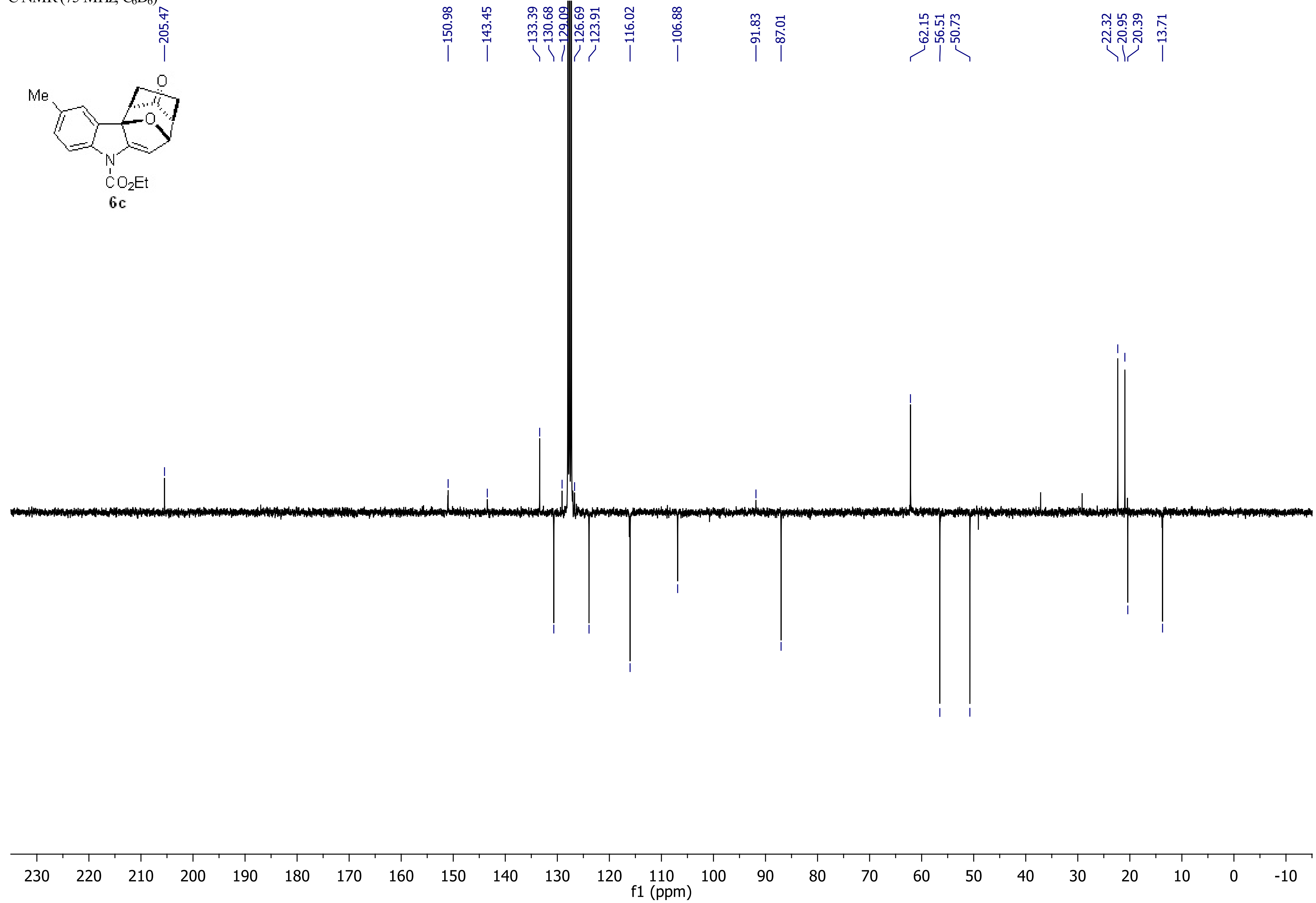

^{1}H NMR (300 MHz, $CDCl_3$)

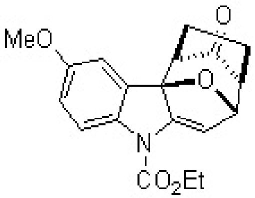

6d

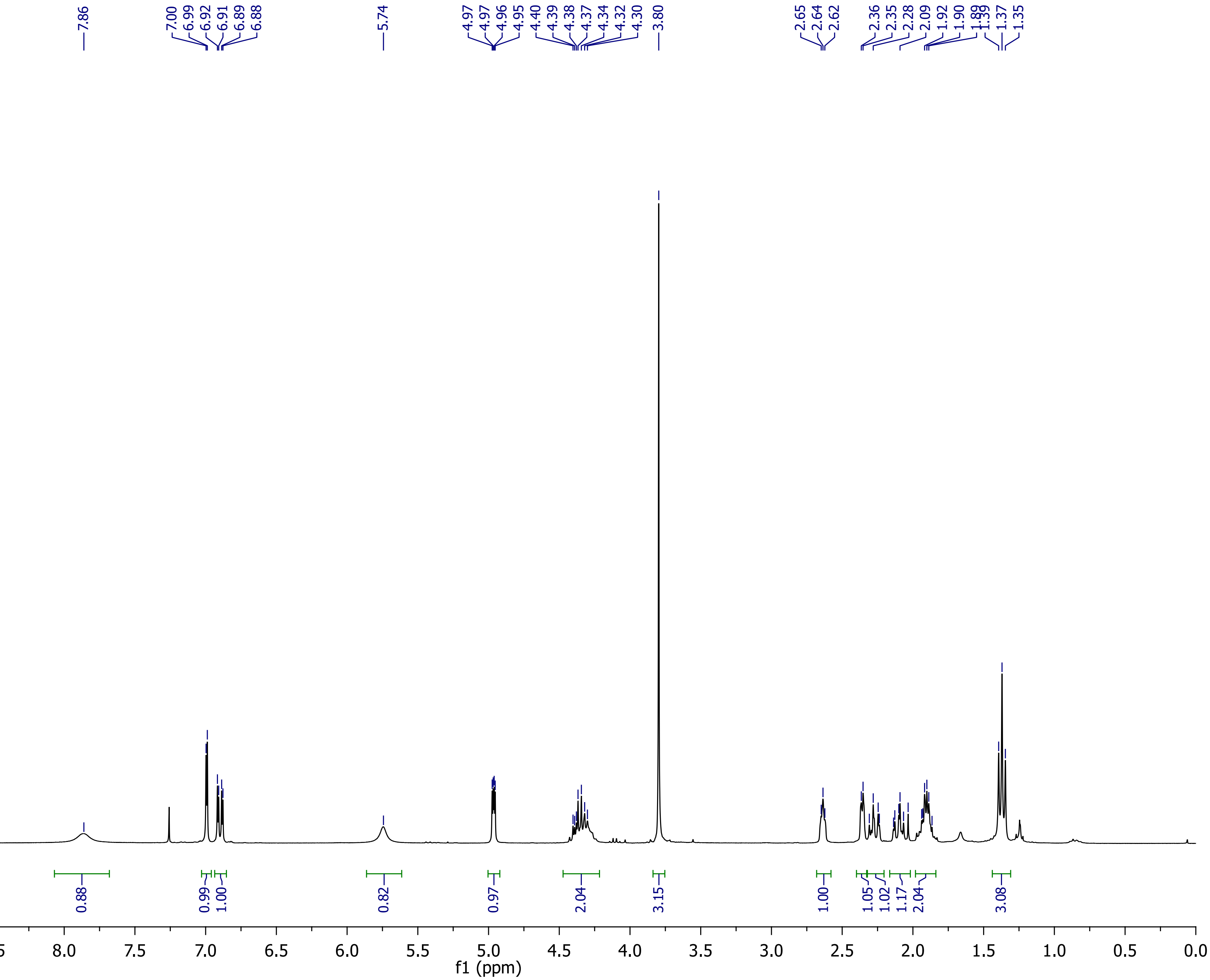

^{13}C NMR (75 MHz, $CDCl_3$)

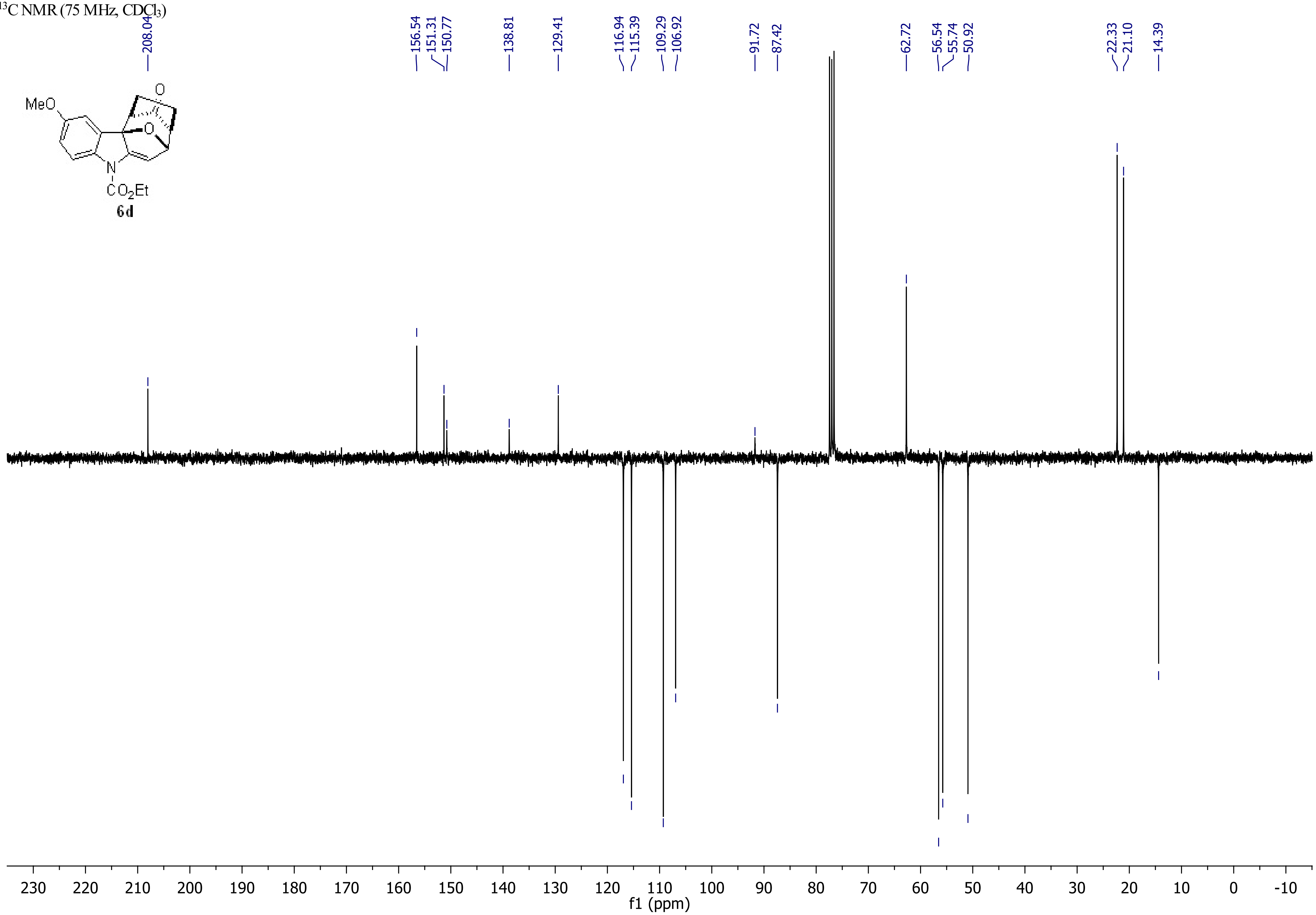

^{1}H NMR (300 MHz, $CDCl_3$)

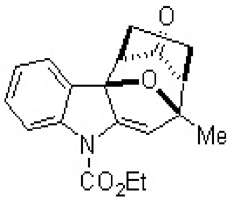

6e

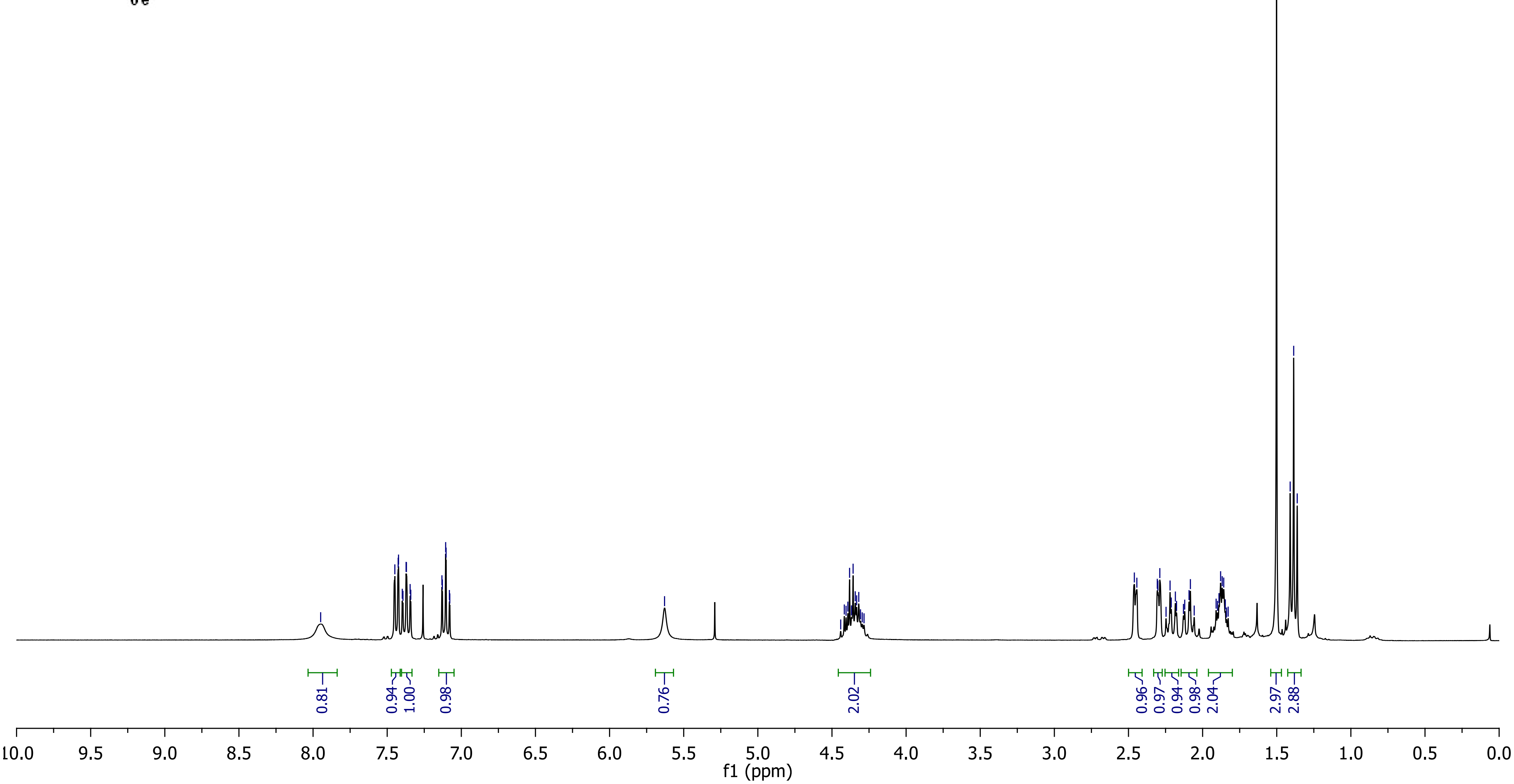

^{13}C NMR (75 MHz, $CDCl_3$)

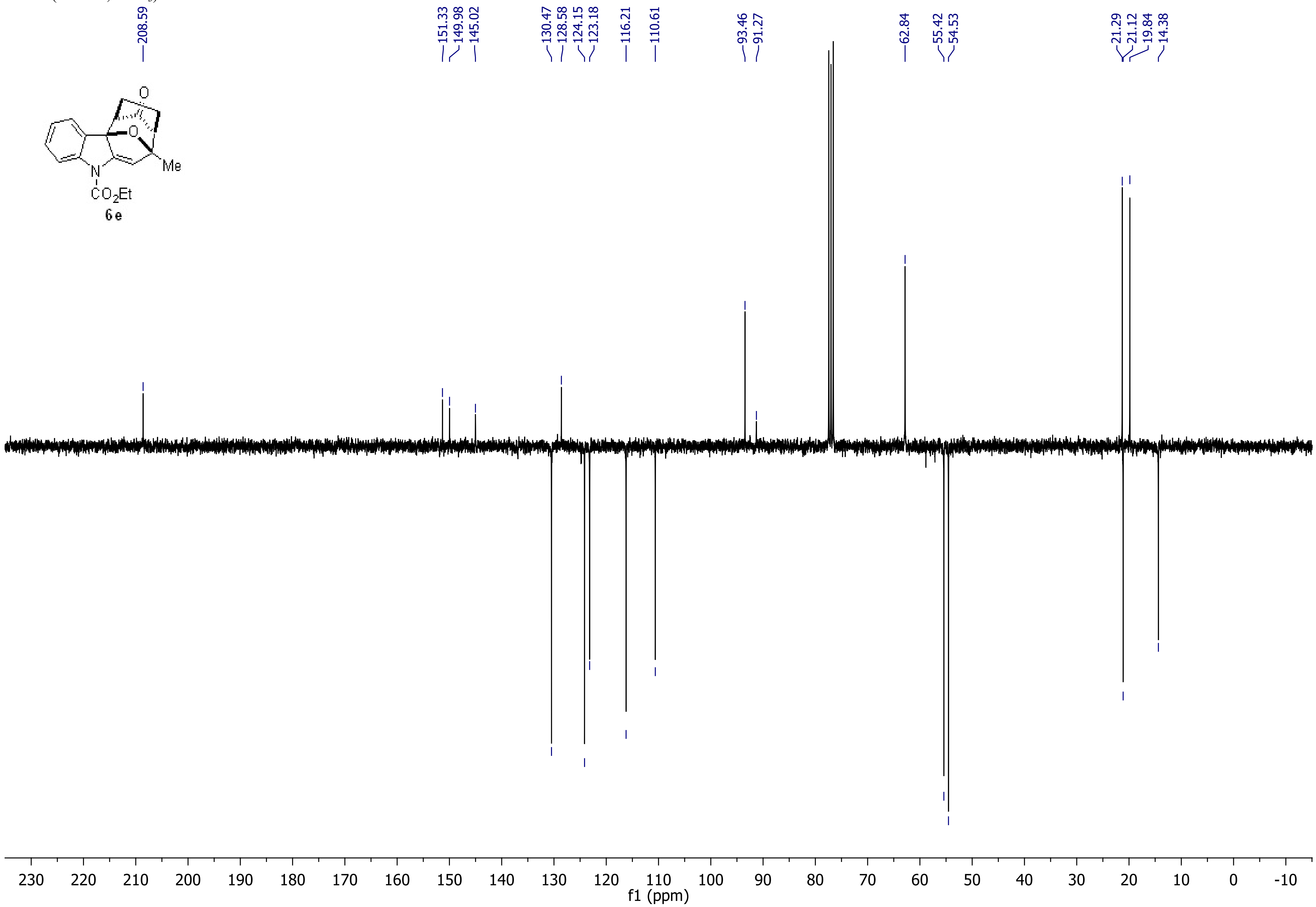

^{1}H NMR (300 MHz, $CDCl_3$)

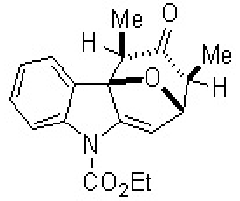

6f

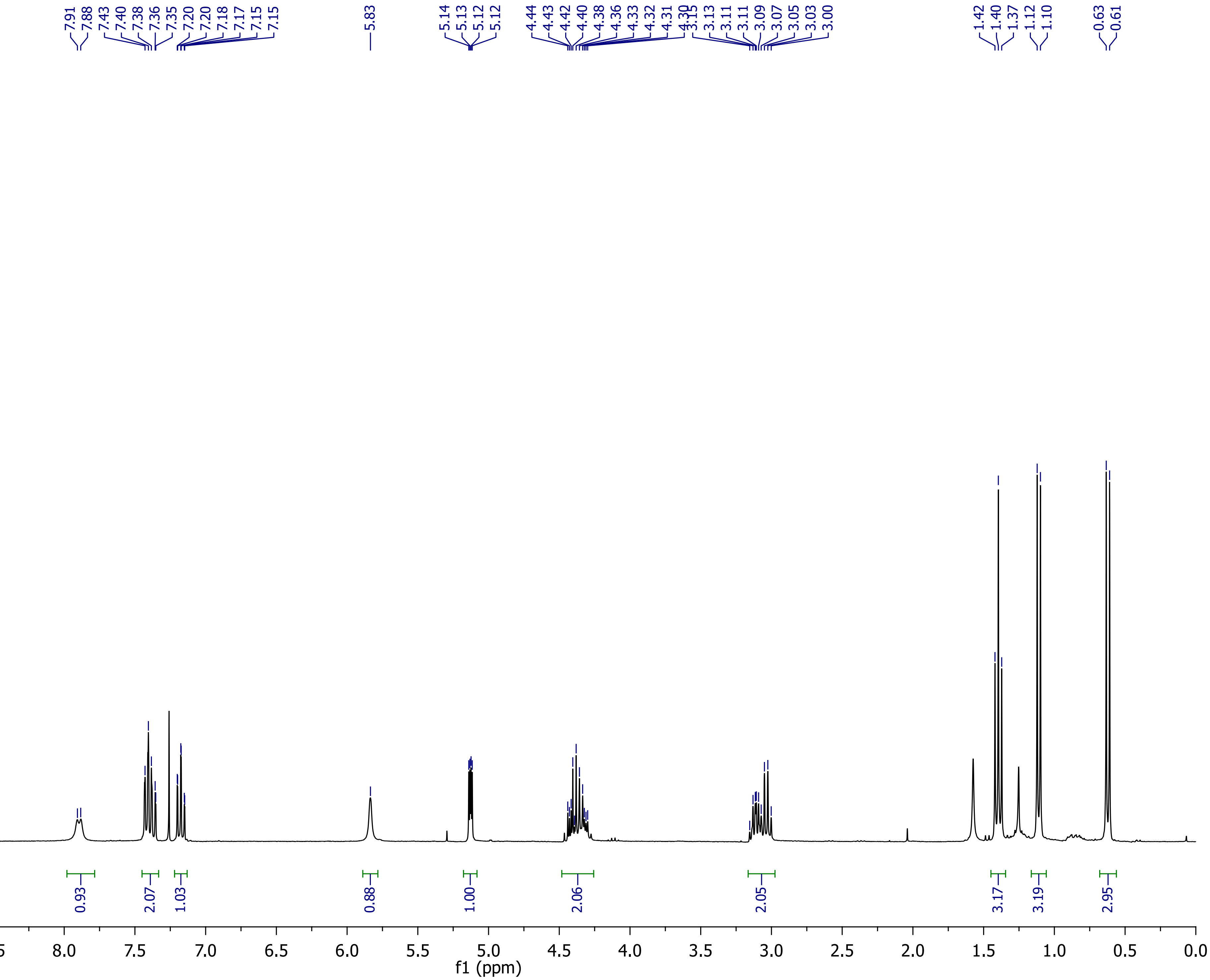

^{13}C NMR (75 MHz, CDCl$_3$)

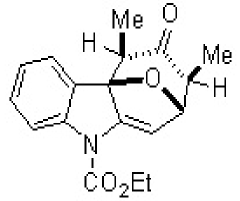

6f

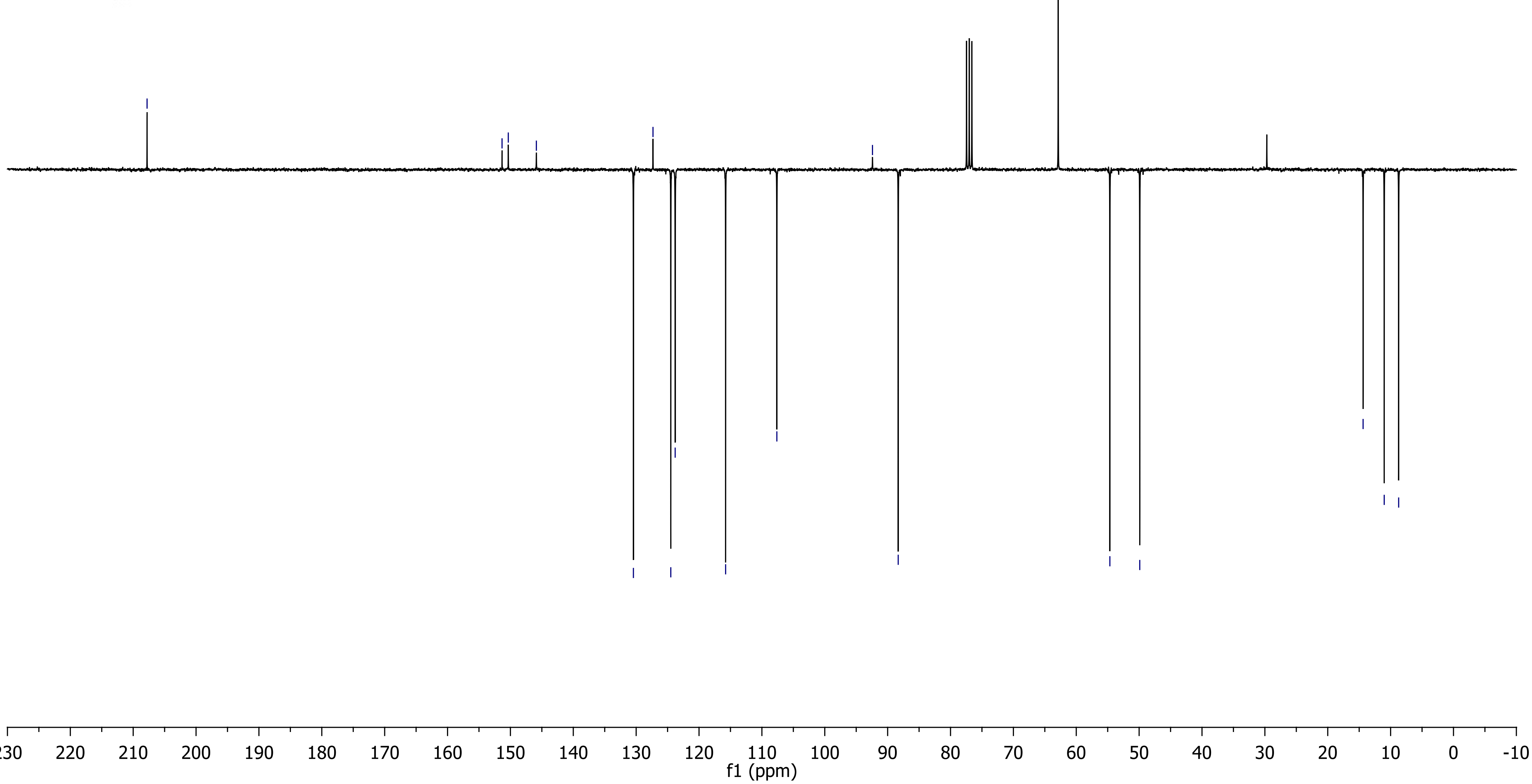

^{1}H NMR (300 MHz, $CDCl_3$)

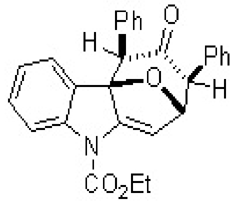

6g

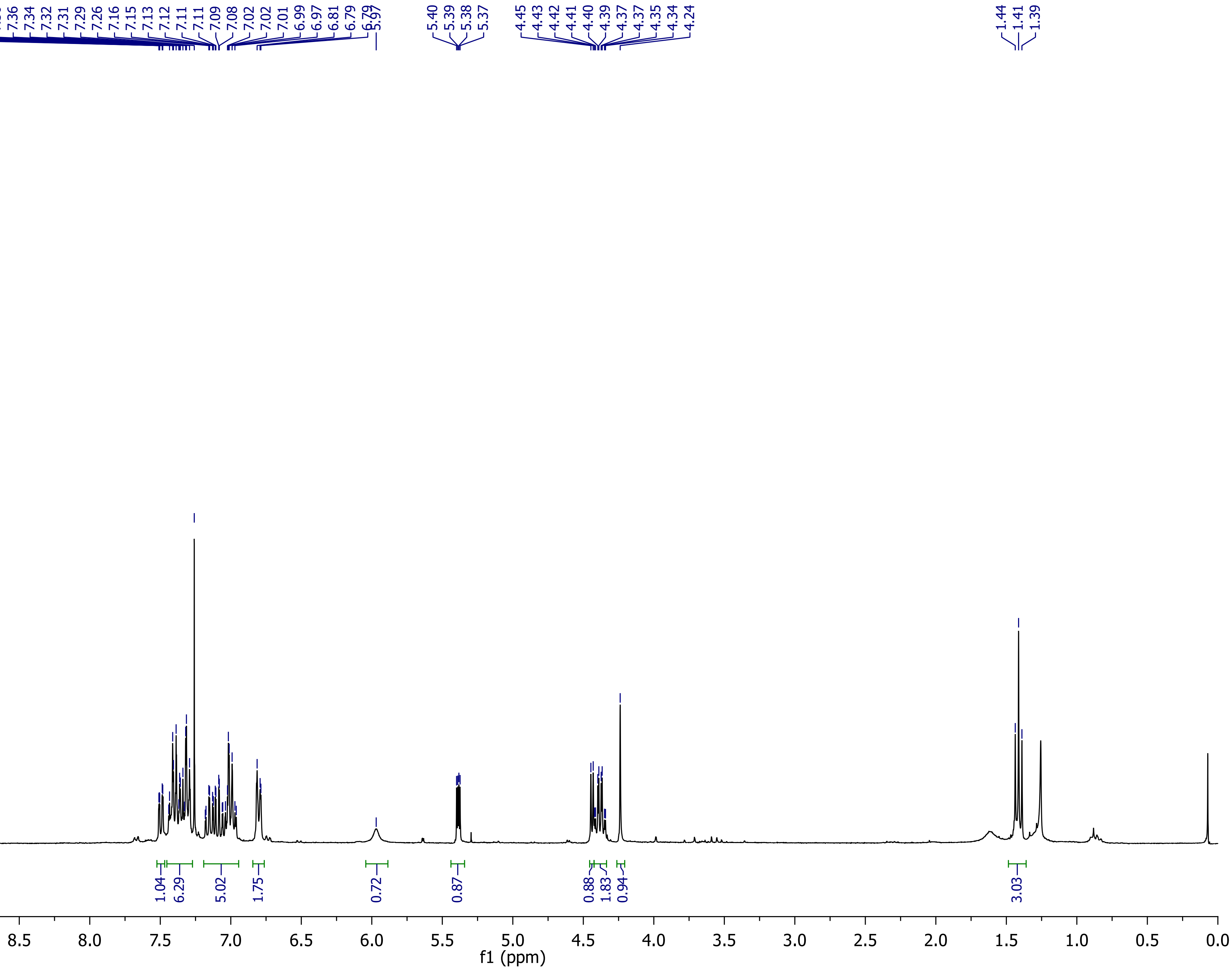

^{13}C NMR (75 MHz, CDCl$_3$)

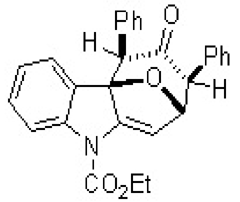

6g

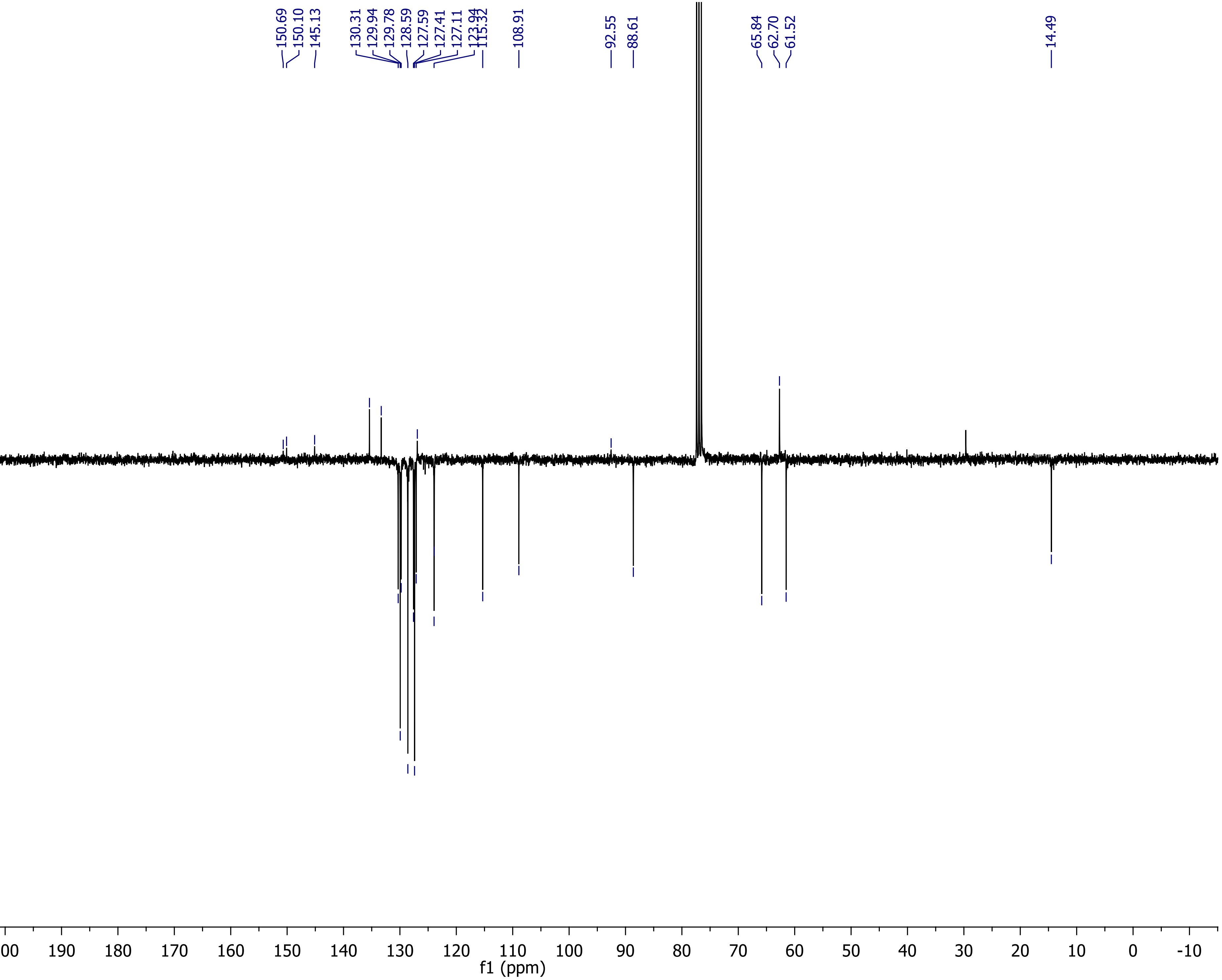

^{1}H NMR (300 MHz, $CDCl_3$)

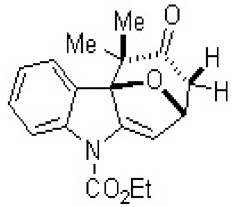

6h

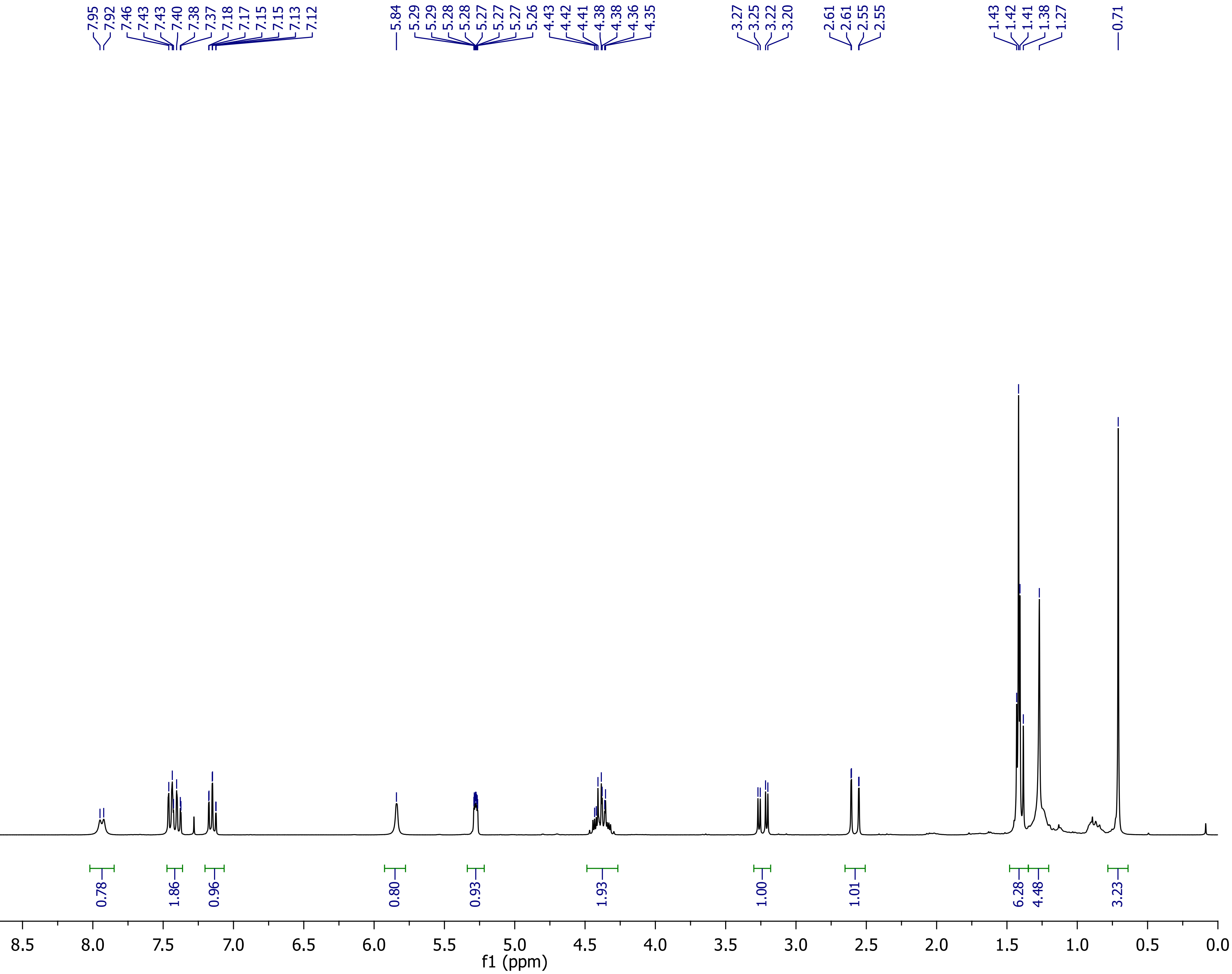

^{13}C NMR (75 MHz, $CDCl_3$)

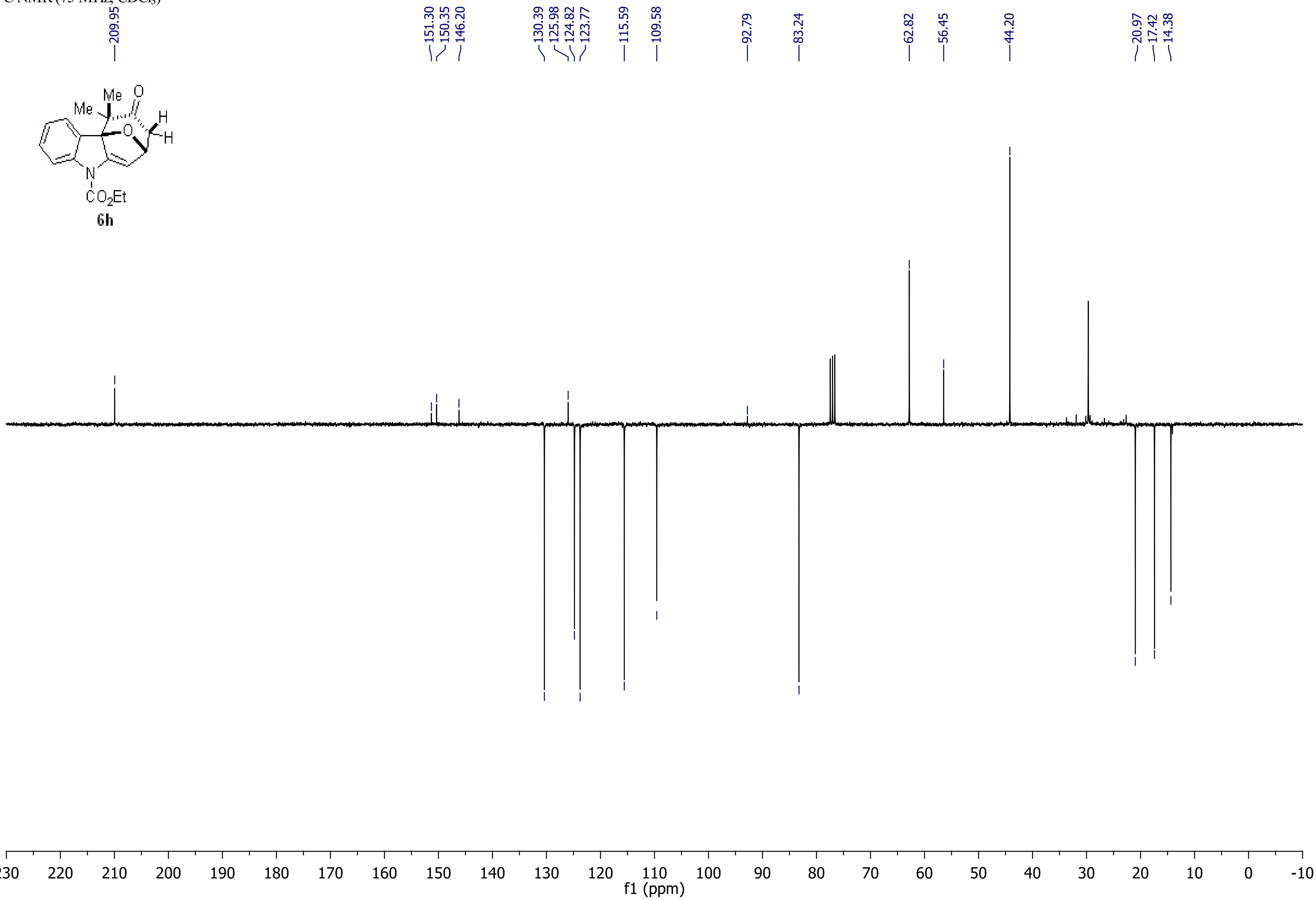

^{1}H NMR (300 MHz, C_6D_6)

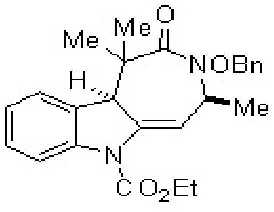

8

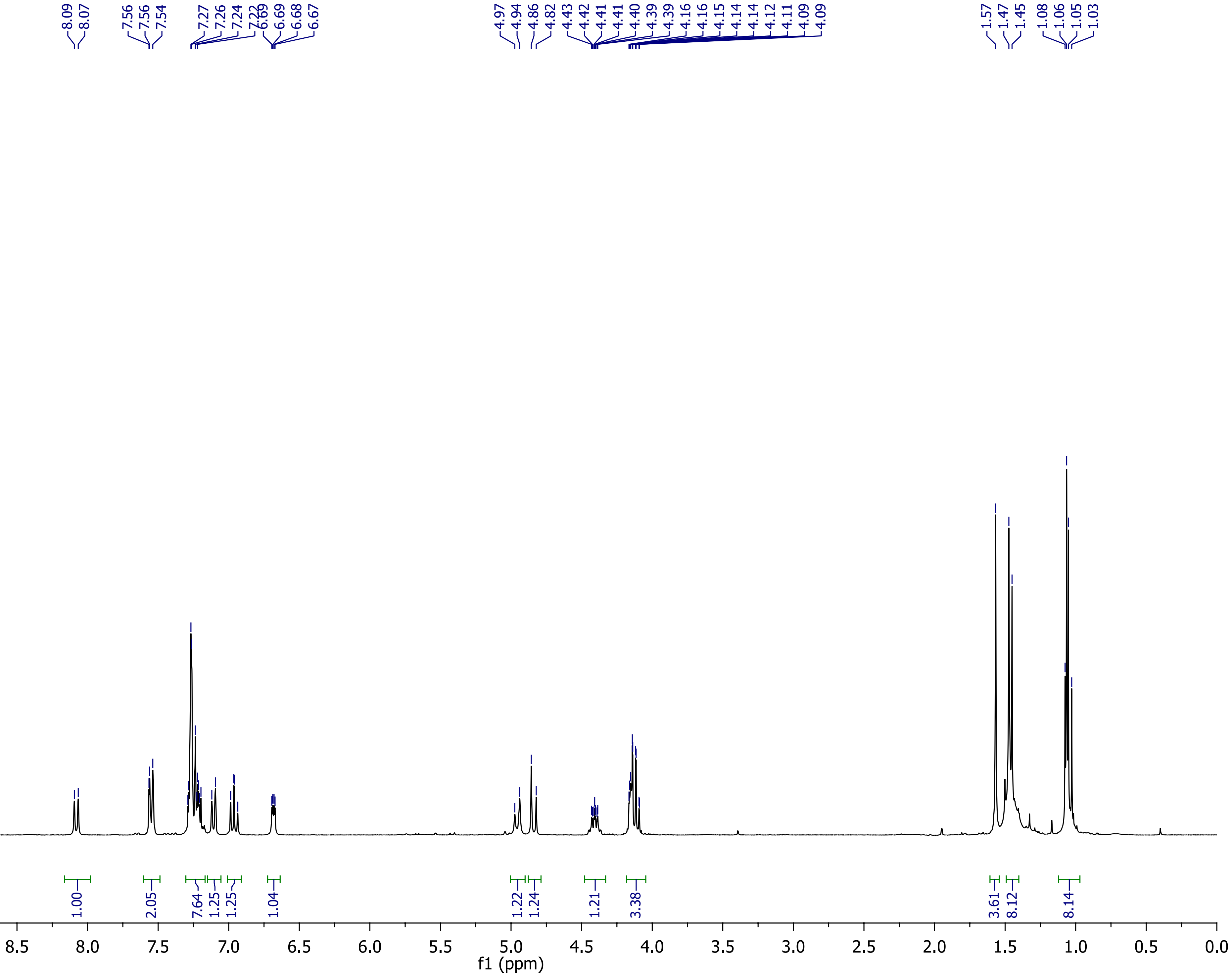

^{13}C NMR (75 MHz, C_6D_6)

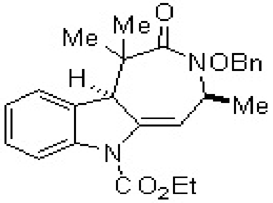

8

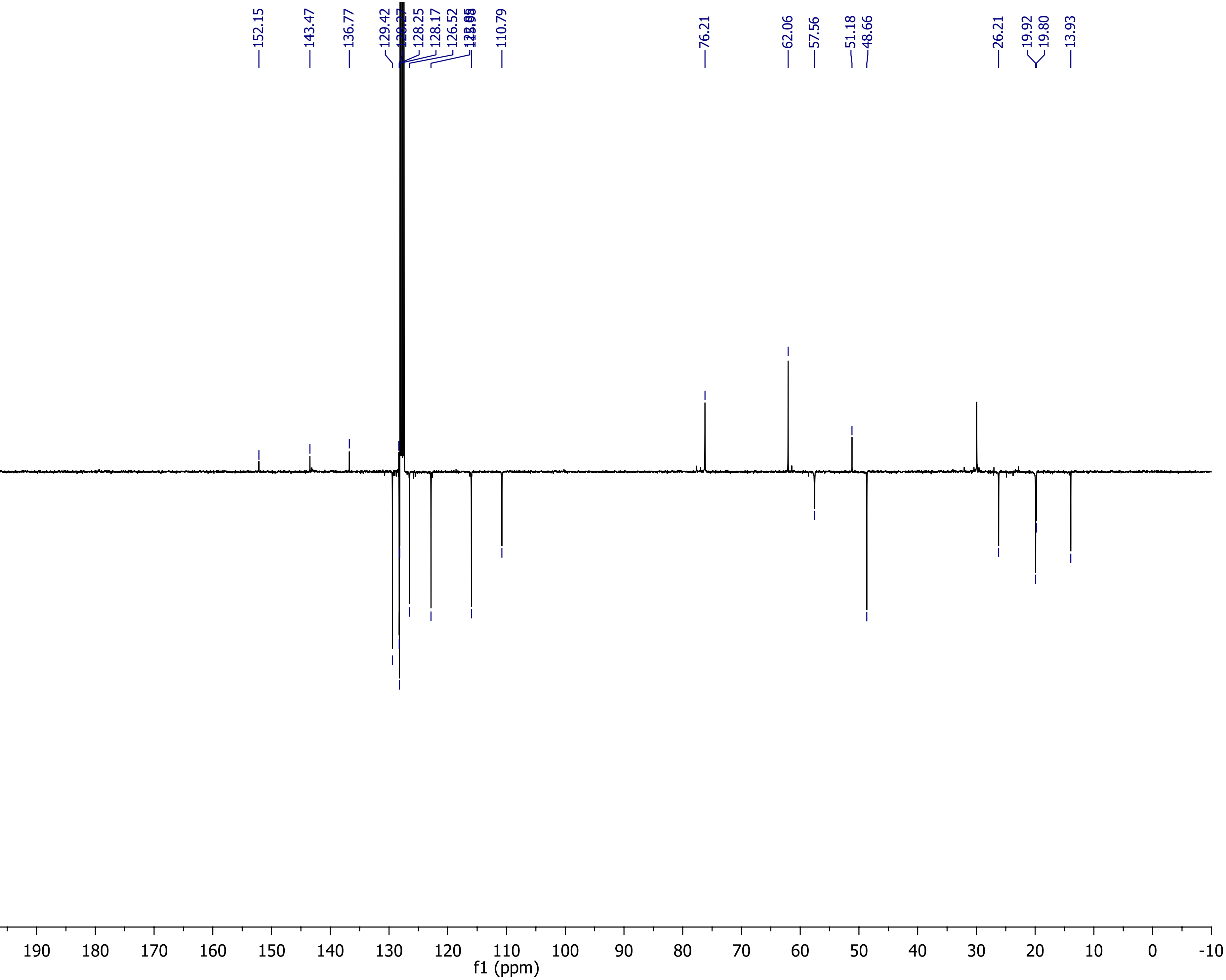

^{13}C NMR (75 MHz, C_6D_6)

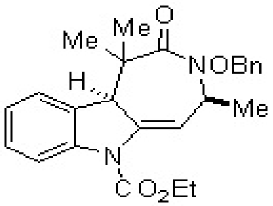

8

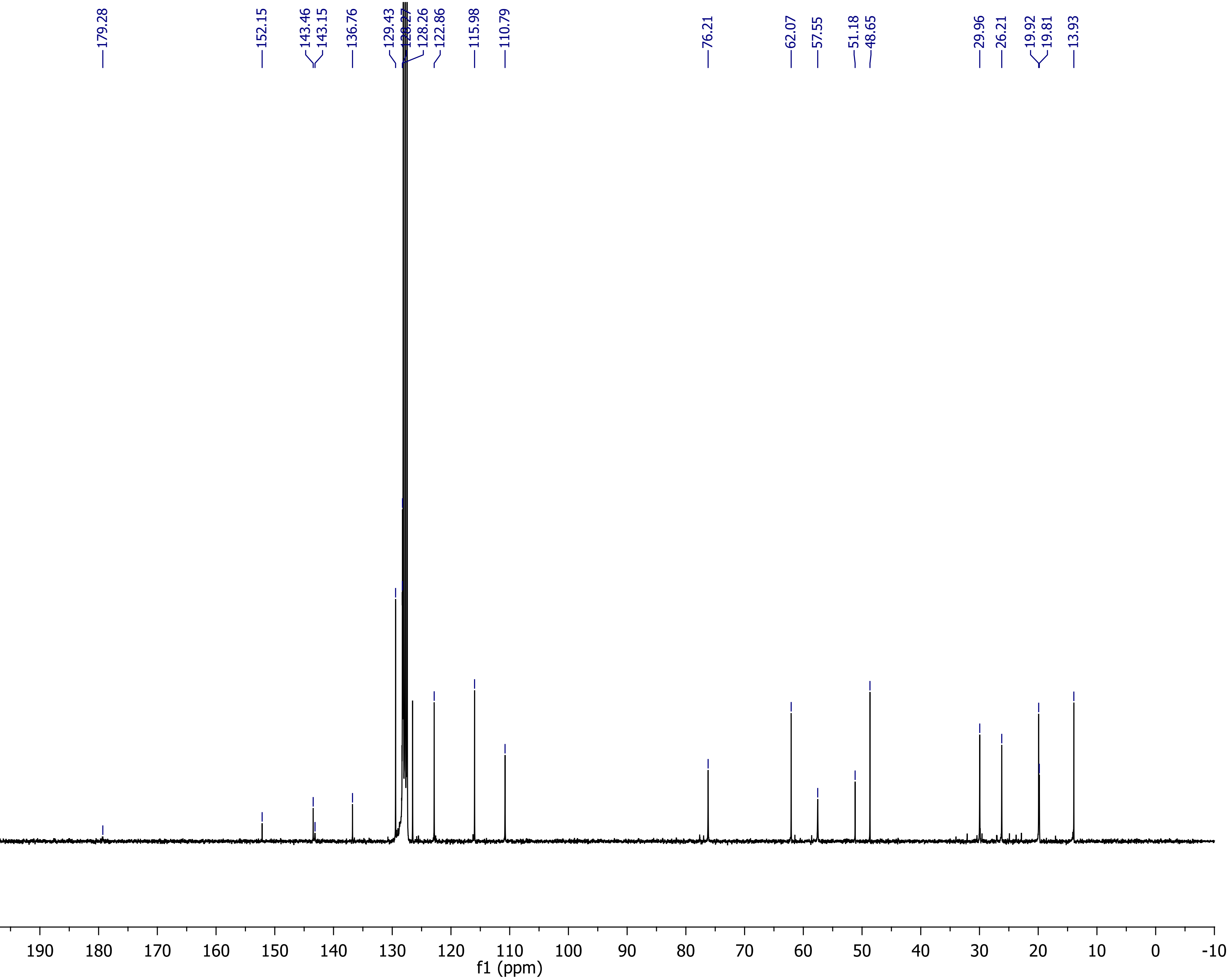

^{1}H NMR (300 MHz, C_6D_6)

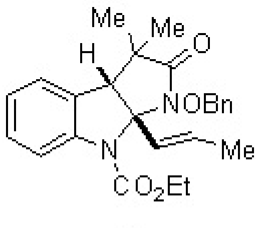

9

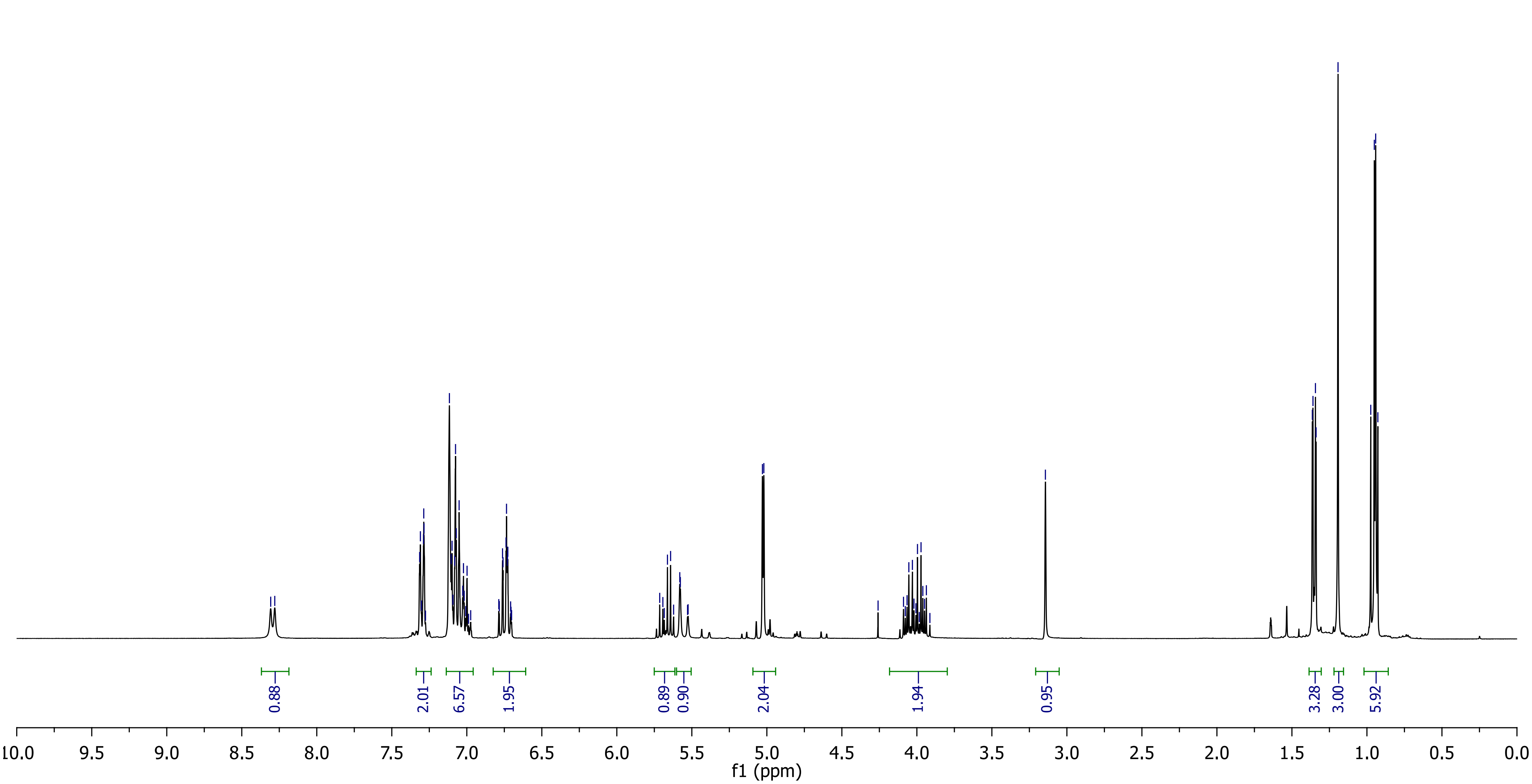

^{13}C NMR (75 MHz, C_6D_6)

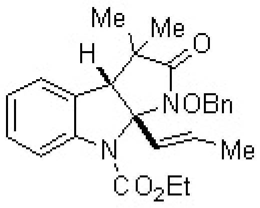

9

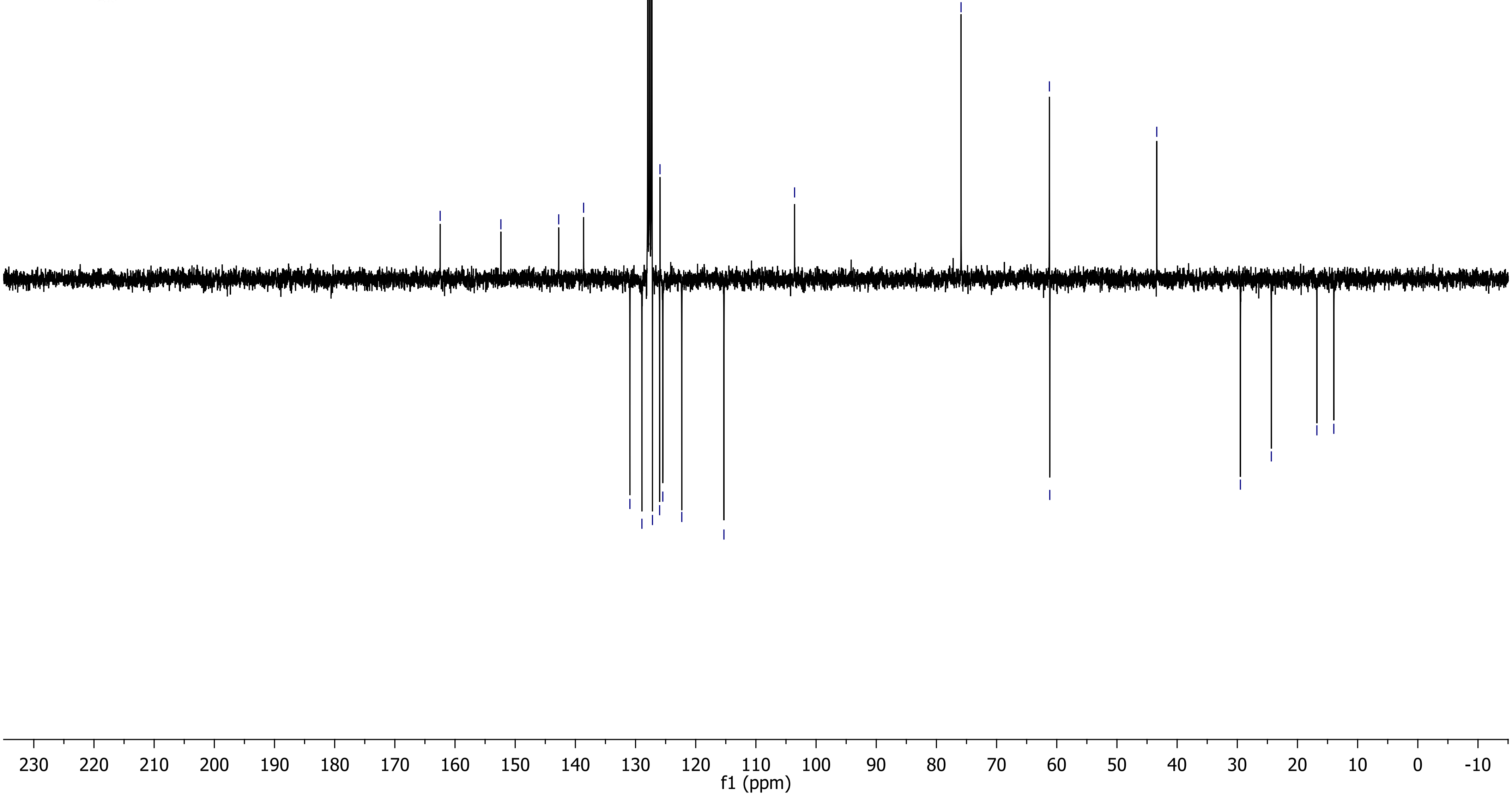

^{1}H NMR (300 MHz, C_6D_6)

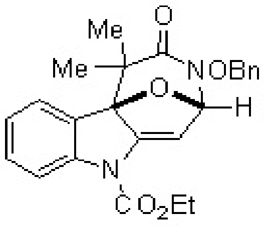

10

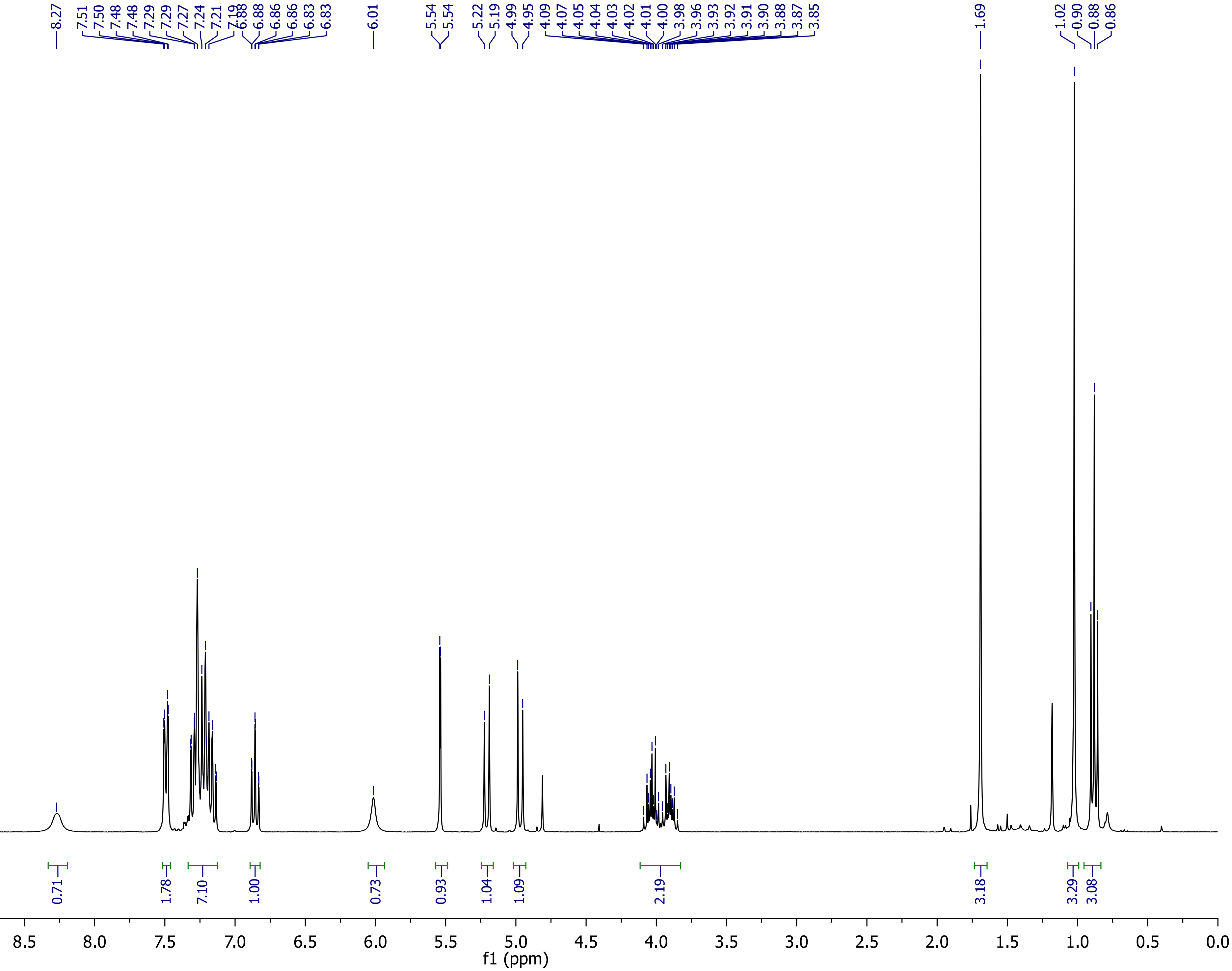

^{13}C NMR (75 MHz, C_6D_6)

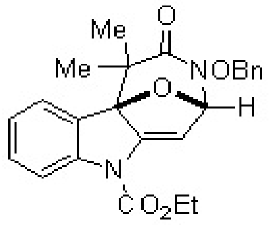

10

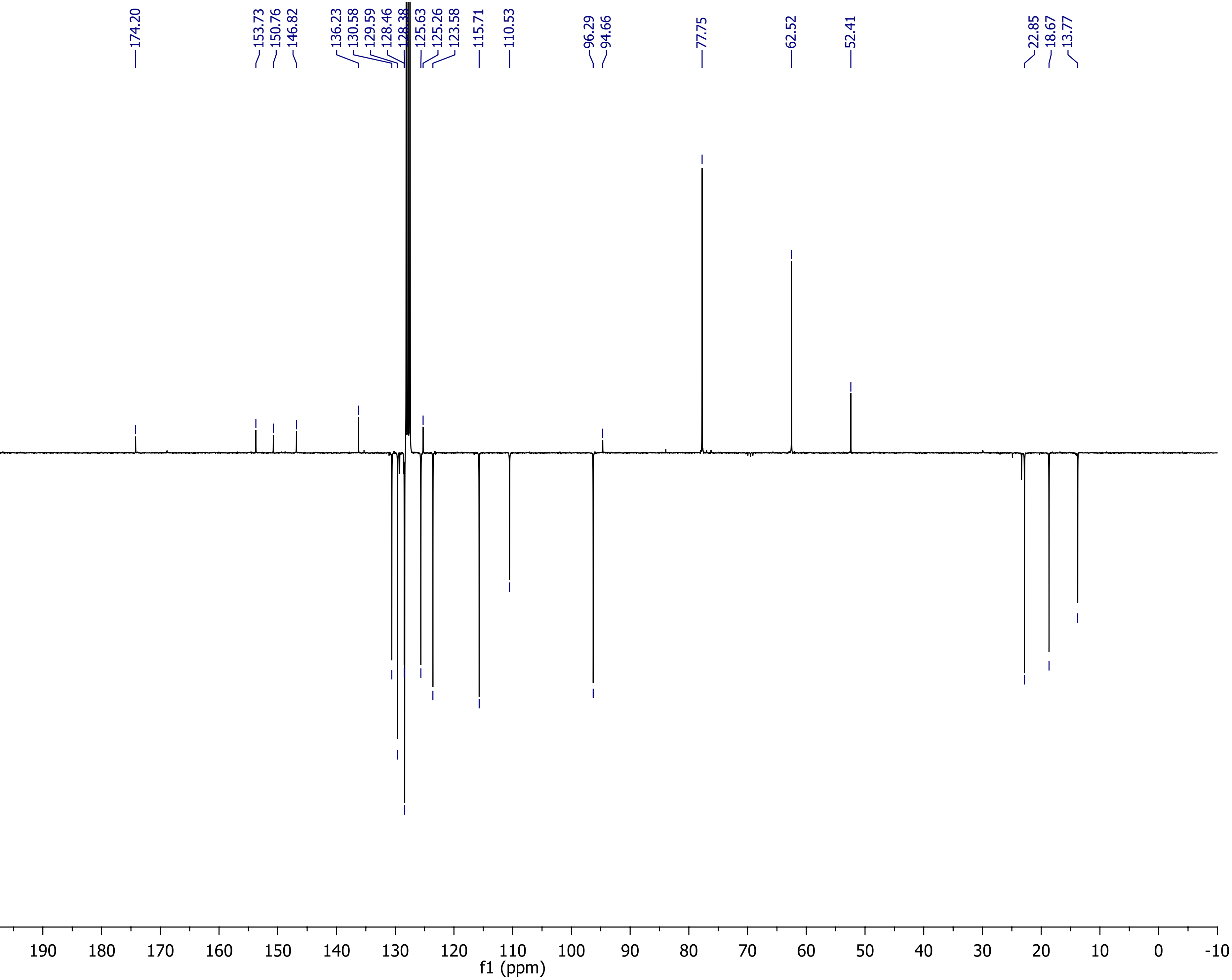

^{1}H NMR (300 MHz, C_6D_6)

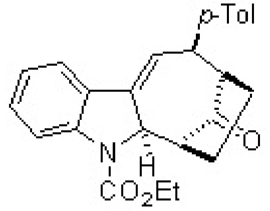

12

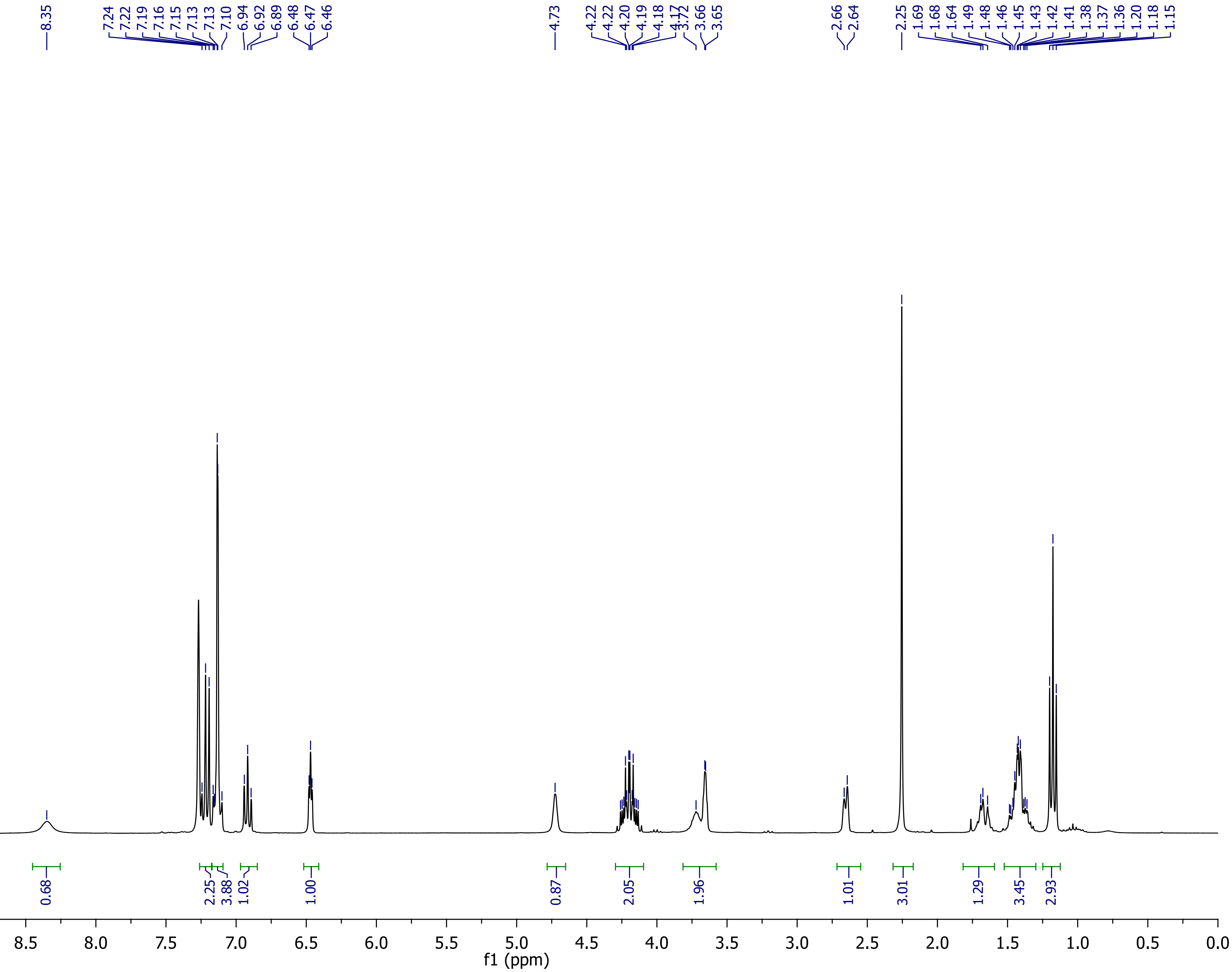

^{13}C NMR (75 MHz, C_6D_6)

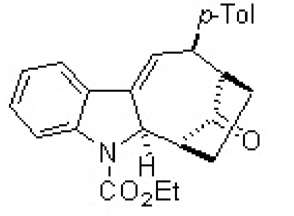

12

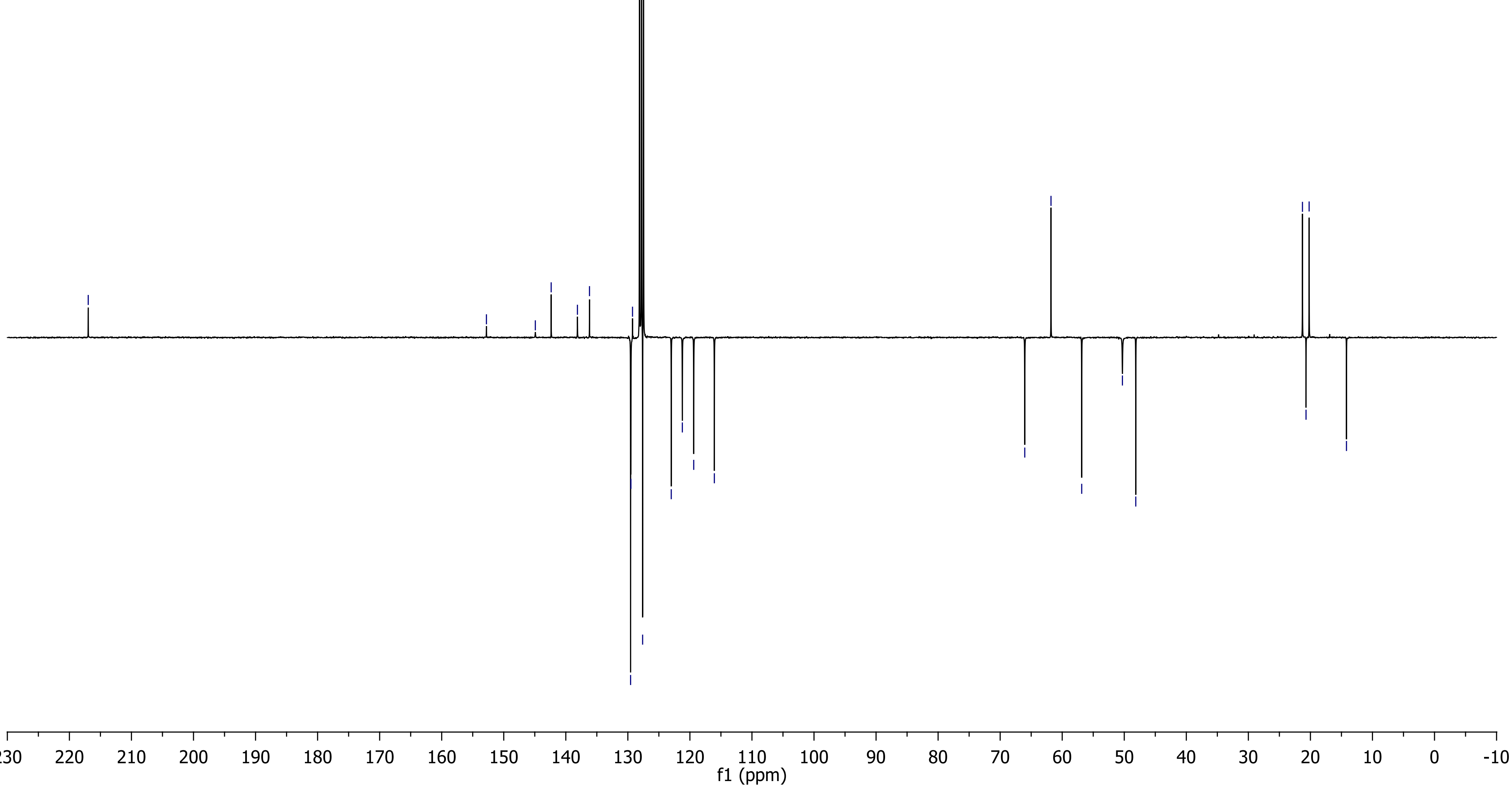

^1H NMR (500 MHz, C_6D_6)

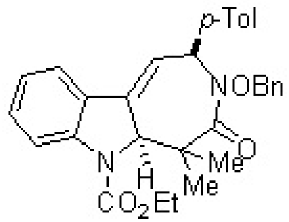

13

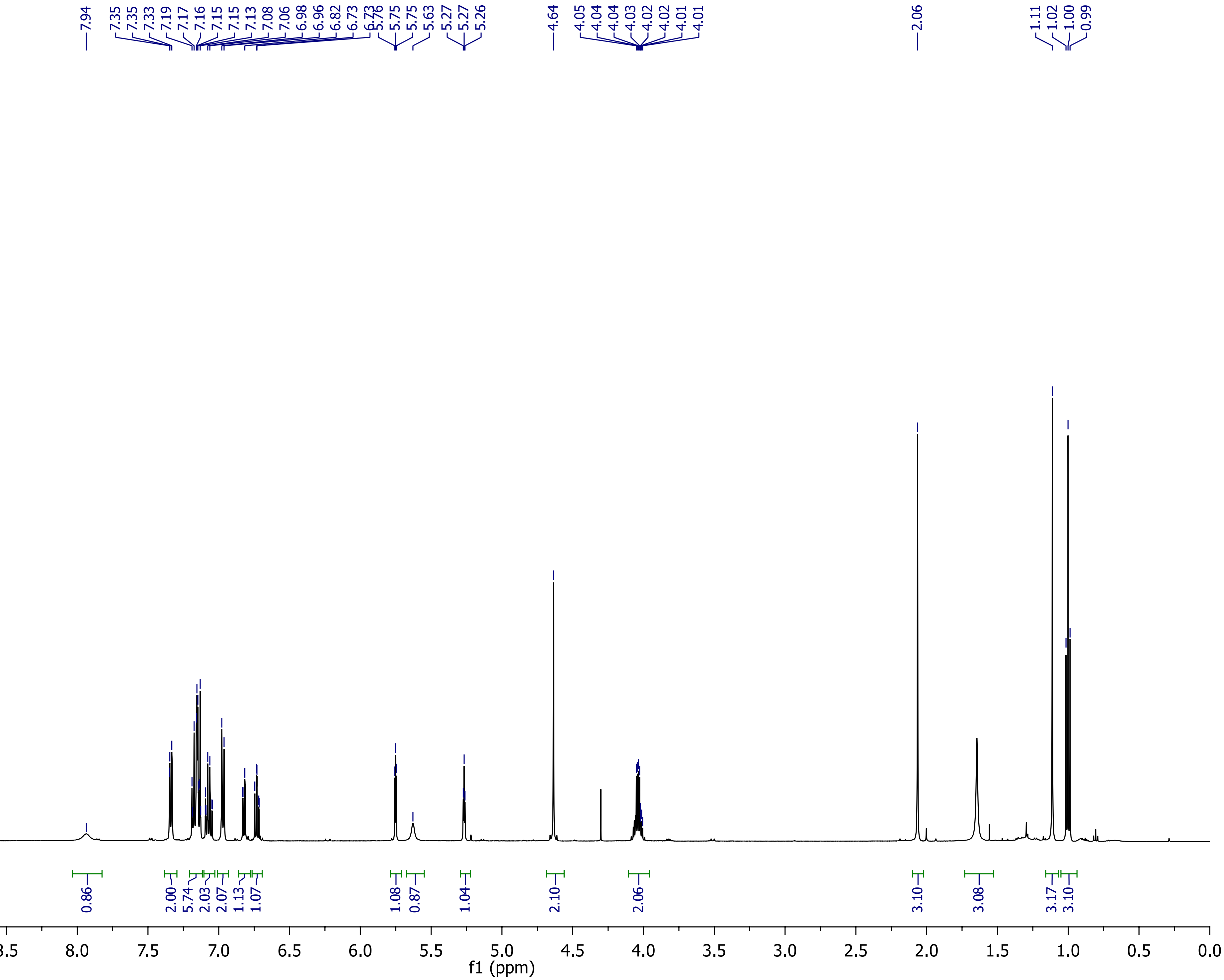

^{13}C NMR (126 MHz, C_6D_6)

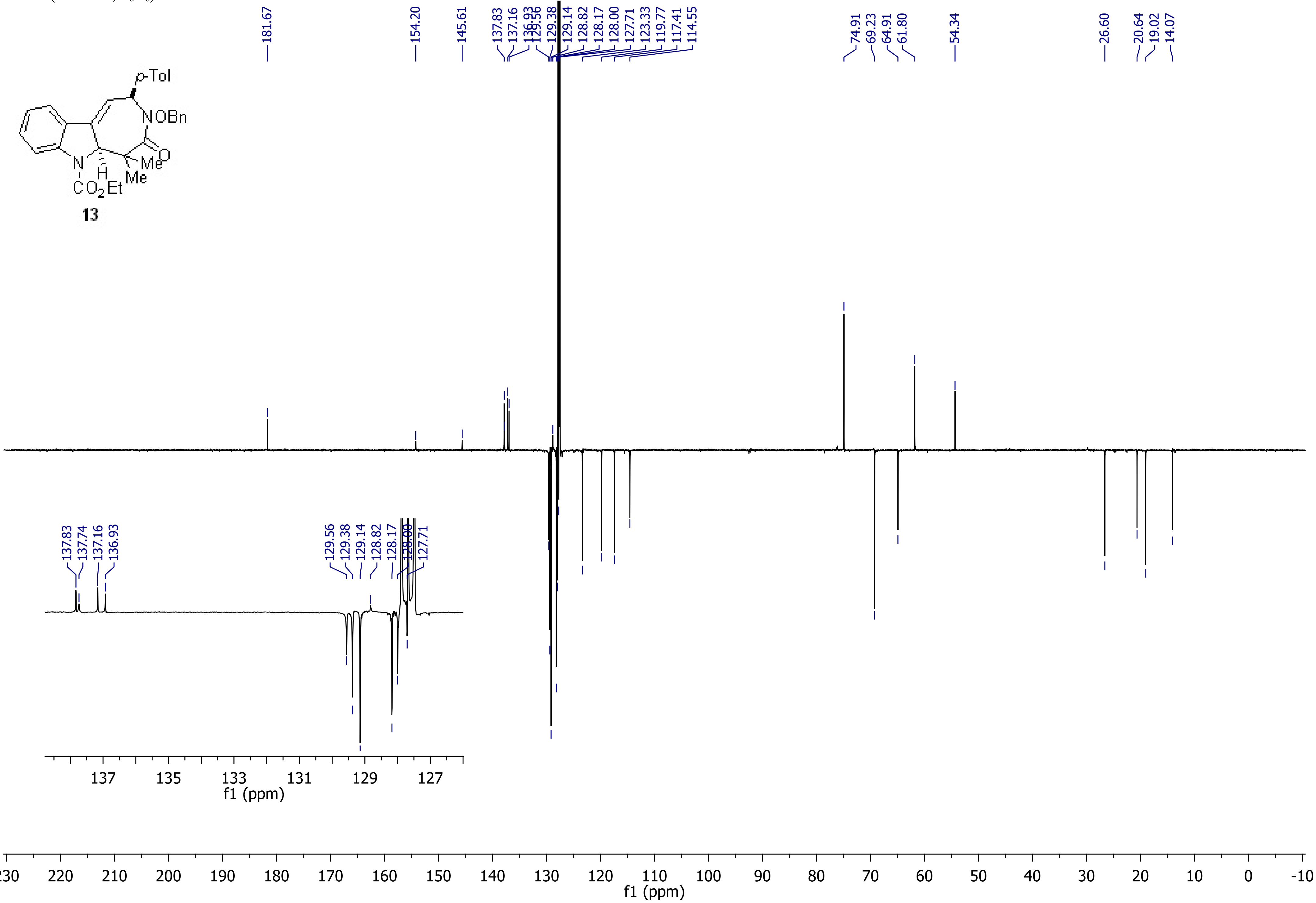

^{1}H NMR (300 MHz, $CDCl_3$)

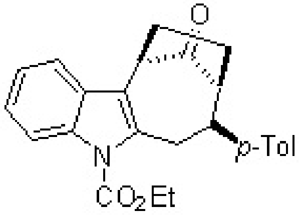

14

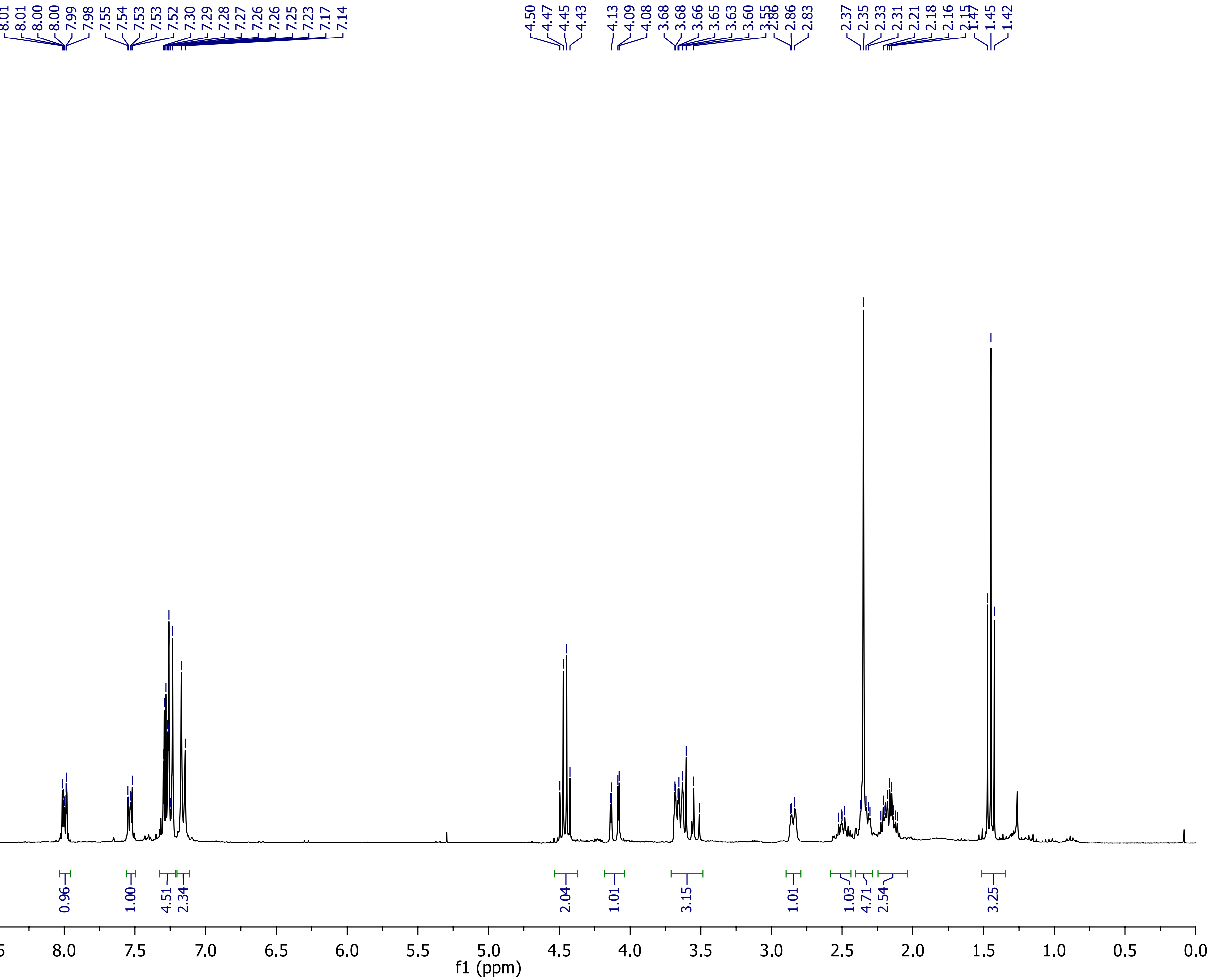

^{13}C NMR (75 MHz, $CDCl_3$)

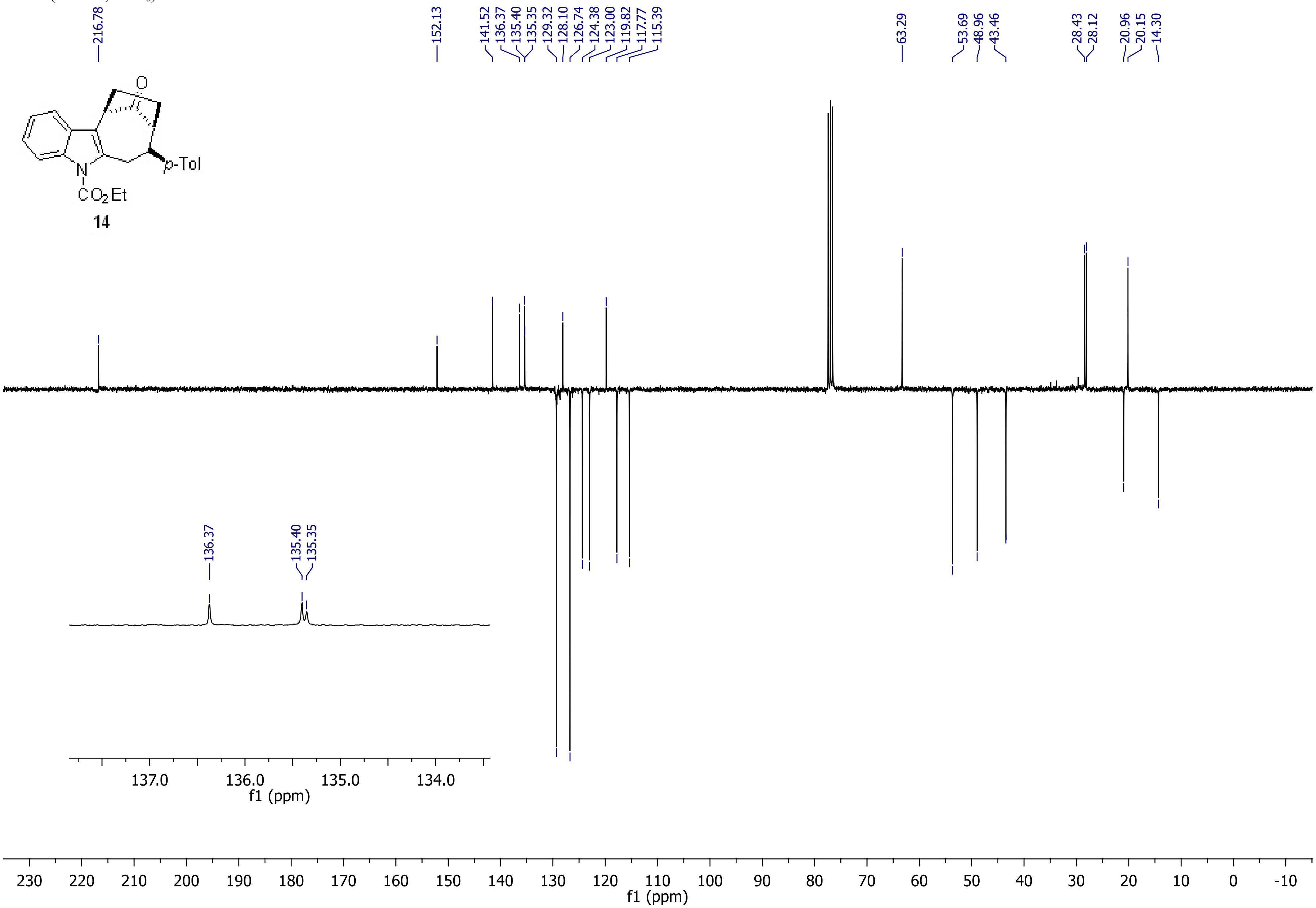

^{1}H NMR (300 MHz, acetone)

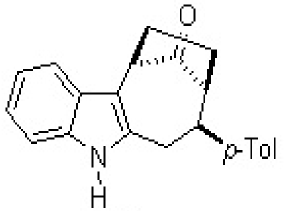

15

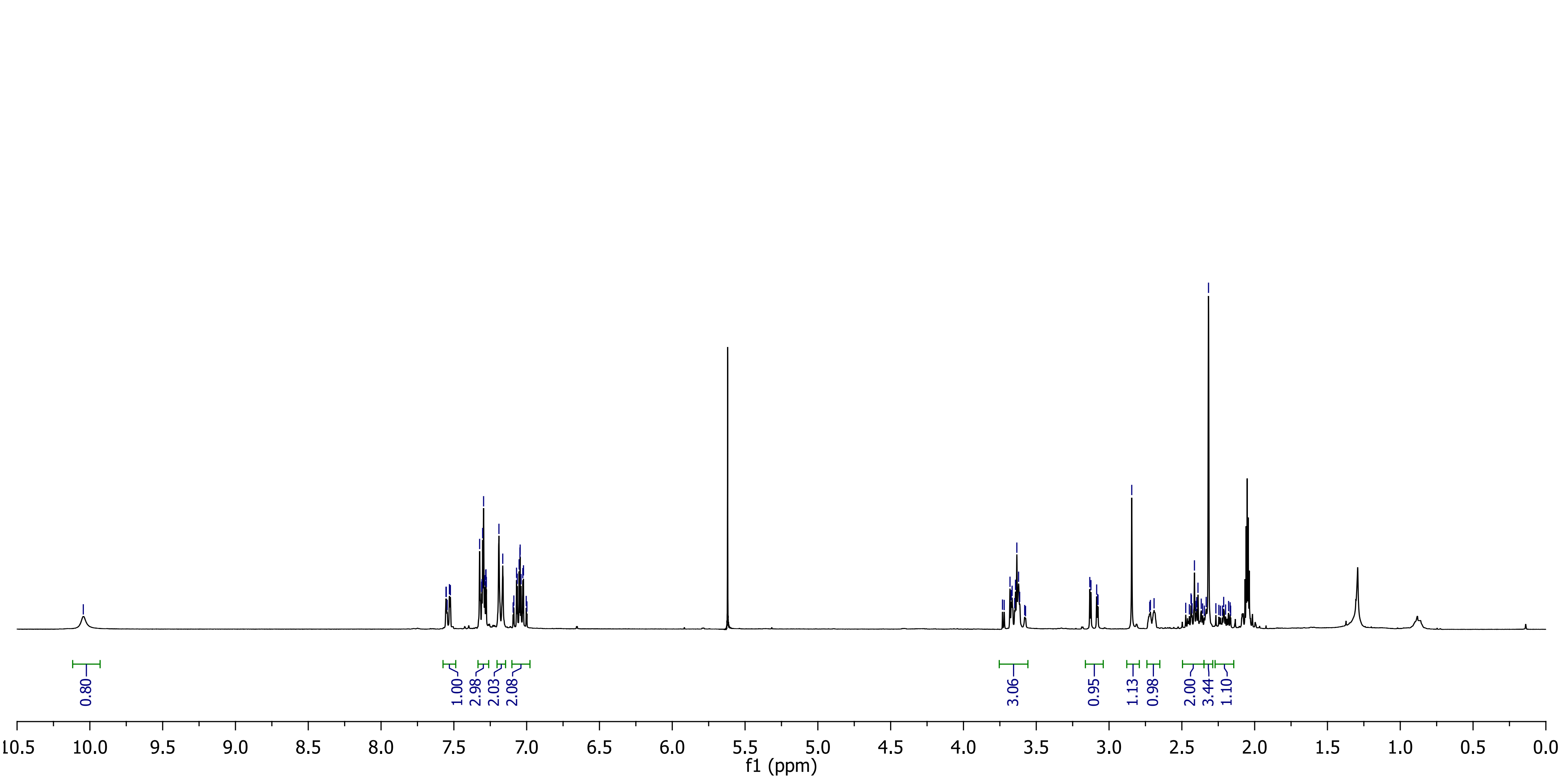

^{13}C NMR (75 MHz, acetone)

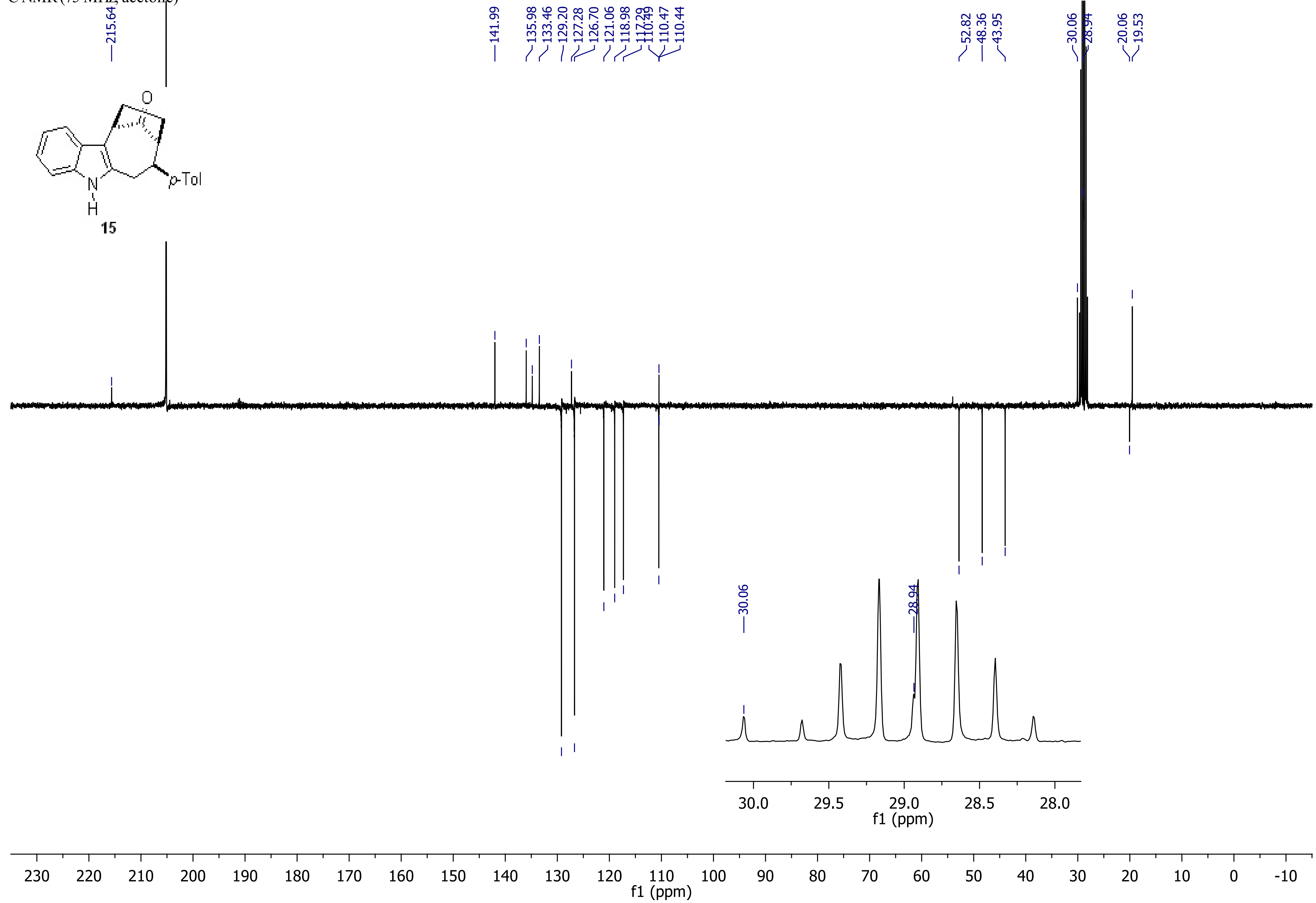

15

^{1}H NMR (300 MHz, C_6D_6)

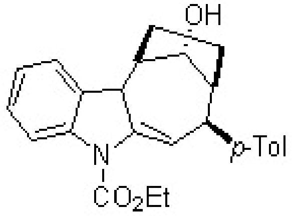

16

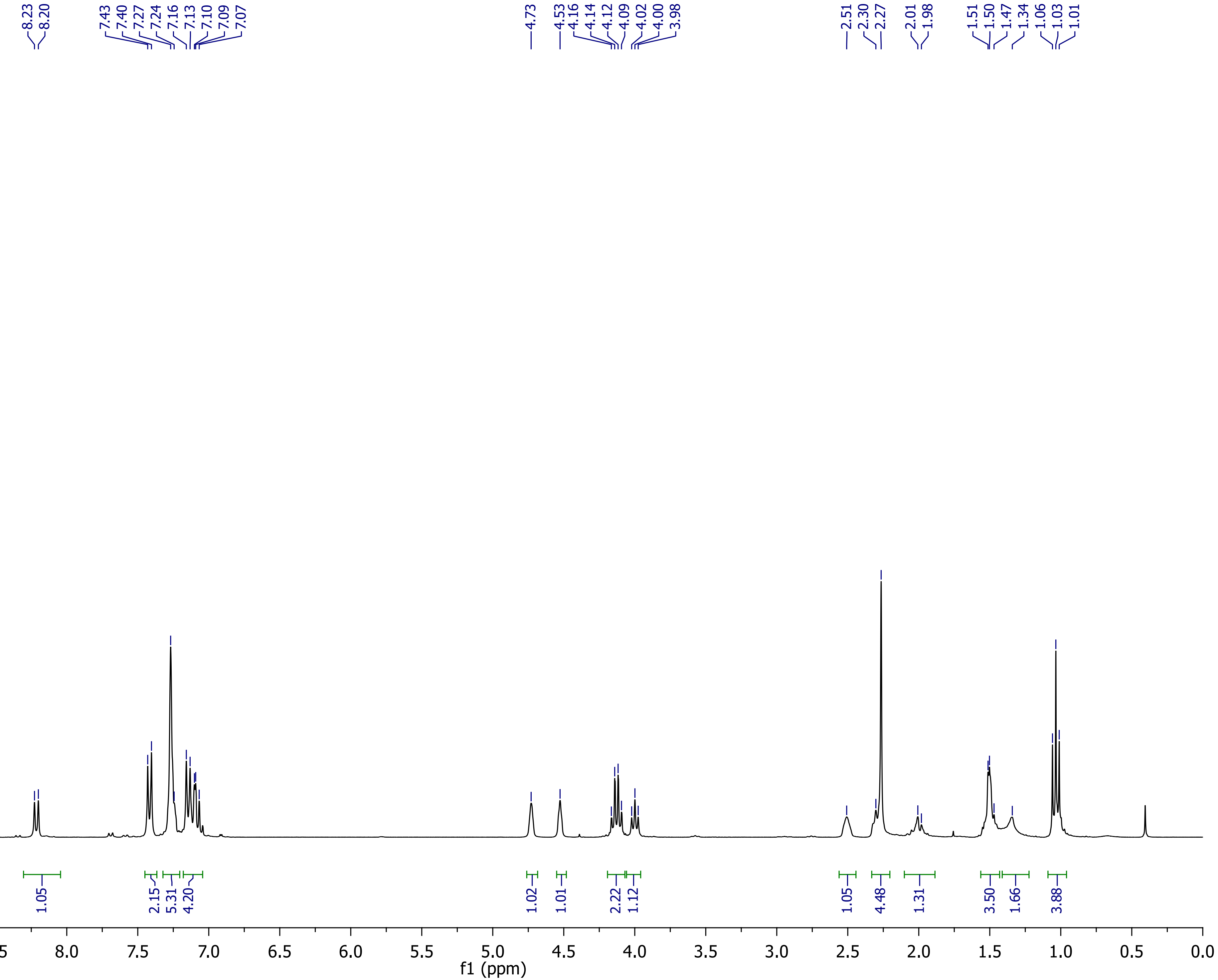

^{13}C NMR (75 MHz, C_6D_6)

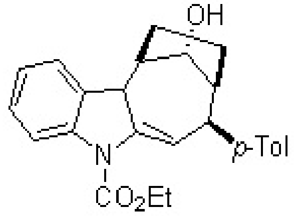

16

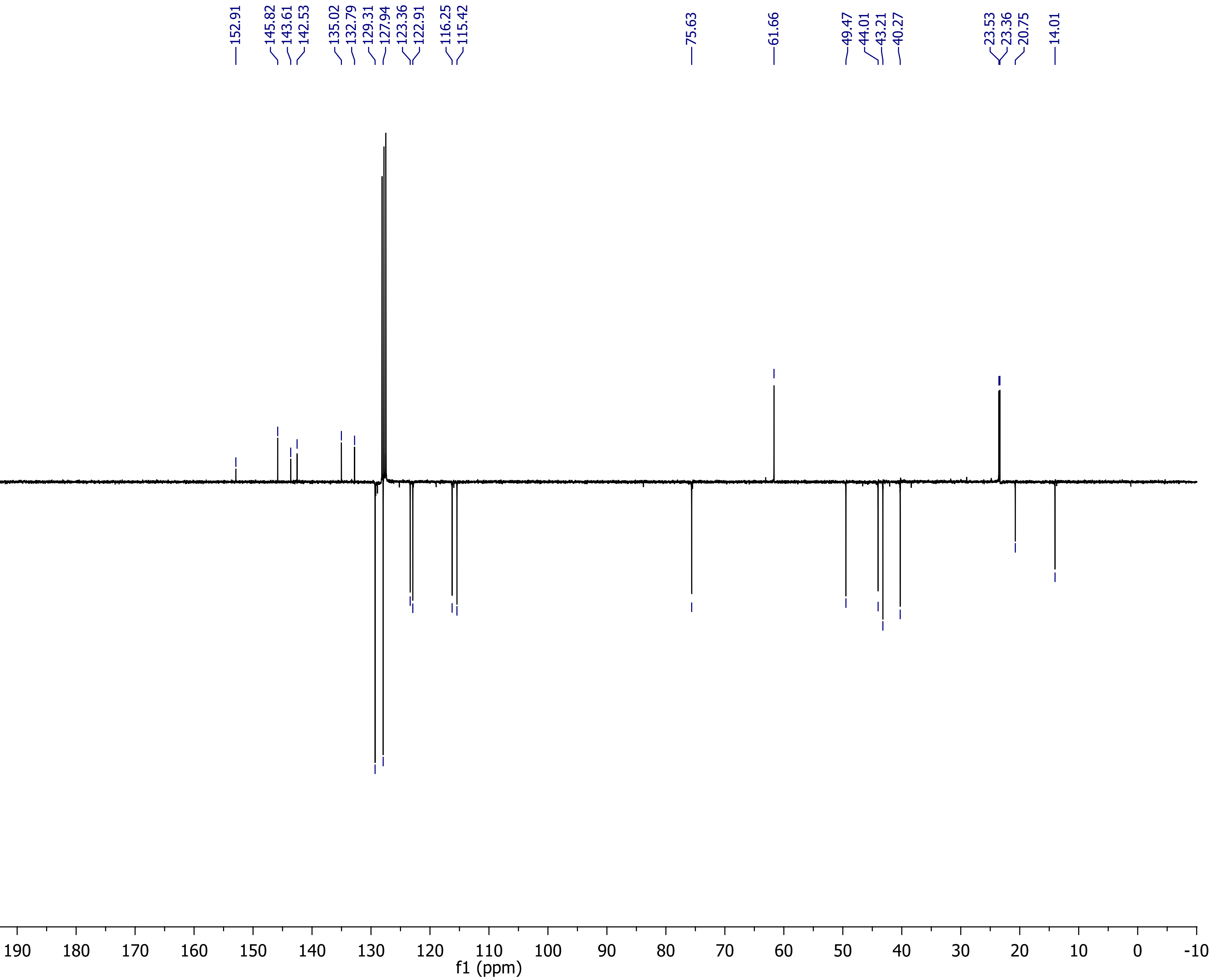

^{1}H NMR (500 MHz, $CDCl_3$)

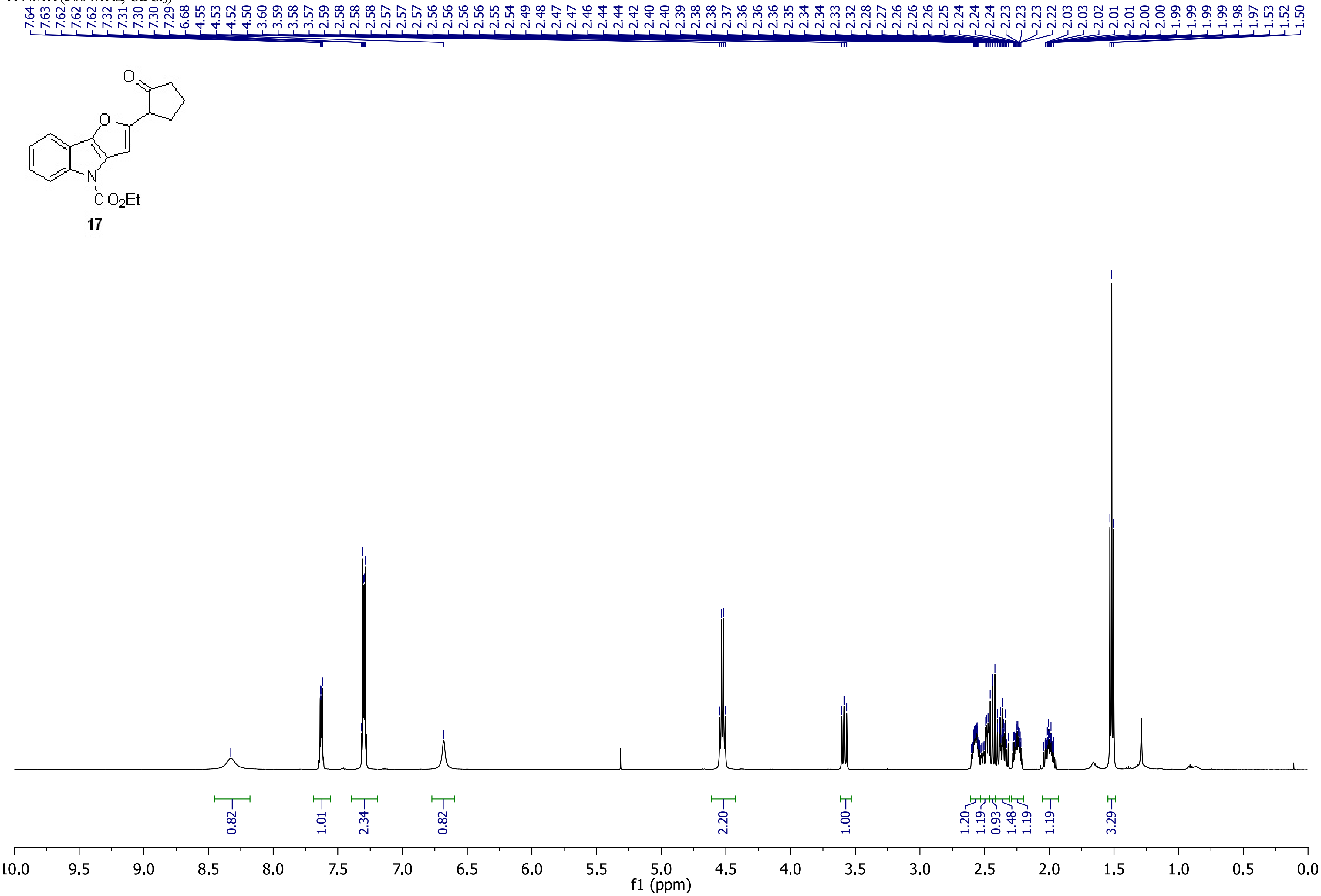

^{13}C NMR (151 MHz, $CDCl_3$)

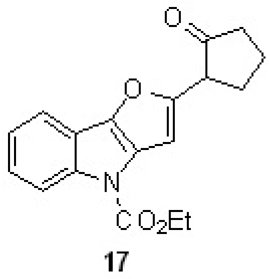

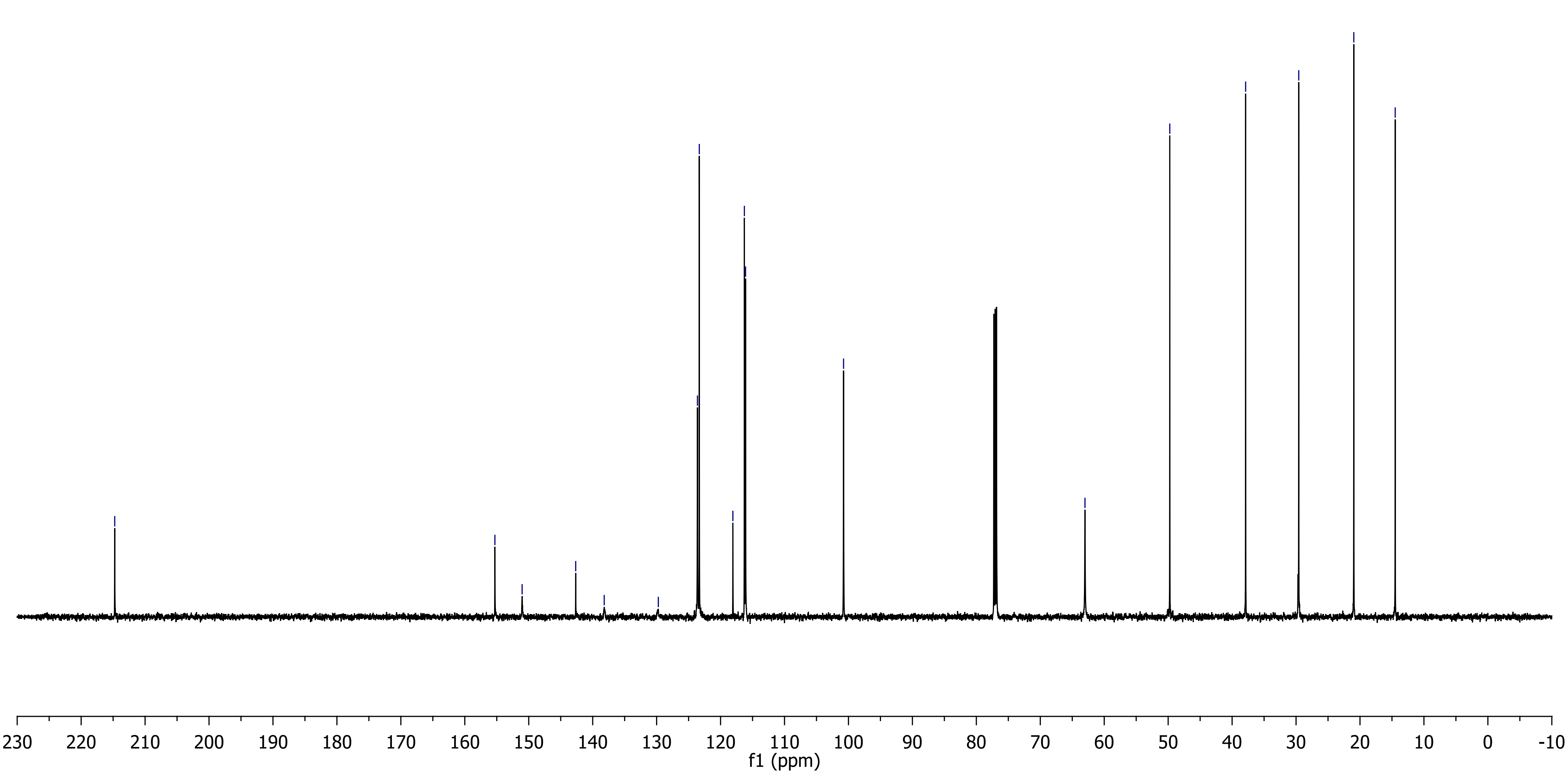

Supplement: Supplementary file 2 — jo9b03117_si_002.pdf [file jo9b03117_si_002.pdf]
